# Supplementary material for: Exploring the challenges and opportunities of multisectoral nutrition programme in Ethiopia: A qualitative study on combating undernutrition during pregnancy
Source: PLoS One. 2025 Jul 3;20(7):e0311336. doi: 10.1371/journal.pone.0311336 (PMC12225801; doi:10.1371/journal.pone.0311336)
Supplement: S1 Data — (PDF) [file pone.0311336.s002.pdf]

**Interview IX: With 05")**

Thank you.

I joine 05 in August 2019. I work here for 4 years. Rural area actually we work in SBCC activity and uuuuuh awareness creation activity. and In the urban erea we work **in school program** and in rural area mostly we started uuuuah with nutrition sensitive agriculture. Uuuuh Primarily giving training , TOT training to woreda, agricultural extension workers, woreda DS, the health extention workers and also selected health office nutrition persons. We provided **TOT** training for them in nutrition sensitive agriculture. Then we cascade that training to uhhhhh daily extension farmers at rural area that will there commonly based on dairy. So we provide the TOT training to woreda government partners then they cascade that training to the kebele dairy farmers. The kebele dairy farmers will take the training at kebelele level or at village level. After that training they uuuuuh they have their own structures but we uuuuuh our project is bridge building uuuuuuh. Building rural income and daily business grows bridge in Ethiopia so that project are dairy farmers that dairy farmers will be grouped in dairy farmers extention groups or DFG. Uuuuh that we will we select that. At the uuuuuh training as kebele we provided to the lead farmers. That the lead dairy farmers that uuuhhh there is a group which include about 25 members. For that member there is a lead. That lead person takes this training and that person will cascade the training in their group. In that approach we conducted at rural area focussing on **uhhh SBCC and awareness creation** activity. After that we have uhhhhhh cooking administration activities at rural area. That cooking administration activities in uuuuh 2 catagories: uuuh **for pregnant** and lactating woman and also for their children the uuuuuh children under 5. For that uuuh group we uuuh **conduct demonstration** focussing on **diet diversification.** Uuuuuh **That is the main** activity that we have in rural areas. Uuuuh regarding **school milk** program in urban area we have a school milk program. School milk program uuuuuh firstly what we do is assess the school. Assessment focusses on the school compound. And the school compound. And a school compound uuuuh the cantina area, the wash facility and the uuuuu **school feeding** Program. That assessment took place after the assessment we identify schools which are appropriate for the **school milk** program. Then we conduct a schoolmilk program **sensitization** workshop. **Sensitization** workshop is basically announcing at school milk program and talking about the school milk program. But at the global level at **national level what we have at ground** and what is the SNV Approach. After that discussion We **will screen** the students who have selection we will sign agreement with schools. Aaaaaahnd after that we will start the **excution** of the schoolmilk program. Basically, the school milk program is uuuuuuuh in cost share **Approach** That means uuuuuh 50% grant from 05 and 50% is **from/parent or volunteers group** or other partners. Based on that so far we have more than 20 schools targeted in the school milk program and about 400 children consume milk in their school twice a week. And the cost share approach is not constant. For example we started 6 months uuuuh or one semester cost share approach. For 4 months 50/50 %. For the next 2 months 75% from **parent and 25%** from 05. For the last month SNV will **leave** the cost share to the families. After 75% we **leave** which is what we will be taken by the parents **or parents leave model.**

In that approach we have drink school milk programs. I think uuuuuuh this is the highlights of the activity. I will continue basically based on your uuuh based on your questions.

Interviewer: thank you very much really. Uuuuh For nice explanation about you providing your introduction as well as the basic activities your office does with respect to nutritional activities.

Thank you very much.

We have 3 important area that we will want to ask you. The first one is challenges.

So challenges in this program.

The first point is what I want to ask you is. Problem. What the nutritional problem looks like in the area where you conduct.

So will you tell me what the nutritional problem in this district and earea where you work looks like?

Interviewee:Okey uh kuch. Actually it is Uuuuuuuuuuh known that there is a high prevelence of stunting as a country. When you come to sidama region also there is the stunting prevalence is almost similar to the national base. So the main challenge or the main problem in our consumers or in our daily **beneficiaries** Or in our clients is what theeeee the diet diversification. Basically they focus on two in sidama almost on 2 types of food groups. And this is Staples or false banana. And the dairy product mostly they use is the skimmed milk. They use mostly the skimmed milk. The consumption of uuuuuuh vegetables, fruits is very low.

Nana: sorry for the skimmed milk? It mean that the uuuuh product..?

**Interviewee: in Amharic አሬራ ማለት አይደለም (Arera malet aydelem).**

**Interviewer: You can use Amharic some times.** the product already cheese. Re-re-re

I2: Jeerrry

I1: Arera: the milk

what I told you last time in Aregash Lodge. they remove some content of the milk like cheese (this is fat) and the water is used for drinking. (Skimmed milk)

I2: ooooh Okee

Interviewee: so that uuuuuuh that **diet diversification is very low**. Theee consumption of fruit is very low, the vegetables very low. The awareness of the community especially the woman's. The knowledge and skills need **to improve** for nutrition practice. They have poor uuuuh experience. The experience is very low. Even without uuuuuh child food preparation and child food preparation also their experience is very low. Based on that we focused on **diet diversification And diet diversification Activity.**

The main challenge or the main issue **bottlenecks or schortages** of the communities is the **diet diversification,** Awareness, skills and the knowledge towards . Skills and knowledge towards **improve nutrition practice.**

Interviewer 2: yeah, so it means that the nutritional problem especially in the pregnant and lactating women is very common.

Interviewee: yeaha. it is very common. That uhhh

Interviewer: because of the especially you say that **diet diversification,** Problem Awareness problem, is the leading cause.

Interviewee:

Ja ehh that is the **main** problem. Actually we work with the government partners so also the health extention workers. They also uuuuh **that information** for us. Uuuuh and based on that we conduct our intervention focussing on that erea. The other thing is what uuuuuuuh currently I'm not sure but the feeding practice they **give priority** to the males and the household **heads** they give **priority** mostly uuuuh nutritious food will be provided to household **heads**

Interviewer: husband?

Interviewee: aaah husband for their husbands so they give uuuuuh priority to husbands rather than childrens. And also they can not consume very, not much during the pregnancy **during lactation in our food.**

Interviewer 1: what was the last thing you said?

Interviewee: yeah?

Interviewer 1: what was the last thing you said?

Interviewee: okay the last thing that the feeding practice uuuuh the feeding practice actually on the household level the women give priority to the household heads or for their husbands other

than the children and therefor they not consume for themselves. But they the give priority for their husband. That is thuhhhh **gap** what we observed during our intervention.

Interviewer: yehaa! excellent. So how you think that the problem t**his trend** is improving or getting worse? How it looks like?

Interviewee: currently it is uuuuh some change is coming. We are also observing. Uuuh also we invite the husbands to the training. Especially in our cooking **demonstration** training the male and the female will take a common training and their habits will change improving some improvement is coming. Not as that **much worriest.**

Interviewer: so the practice as well problem in improving?

Interviewee: yeah

Interviewer: because of your intervention.

Interviewee: yeah

Interviewer: thank you very much but uuhhh have you heard about multi sectoral nutrition program?

Interviewee: yeauhhhhhh previously I worked also in mutlisectorial nutrition program meuhhhh during the uuhhh before I joined this uhhhhh I **were well aware** about multi sectoral but after coming this uhhh 05 we work with uhhhh lifestock office, health office and agricultural office mostly we focus on these three. Even if we are uuhhh nutrition sensitive agriculture **dialogue** among the three sectors. Yeah but we not include all the multi sectoral nutrition program partners. There is a lot of multi sectoral nutrition program partners but we focus on three health office, lifstock, and agricultural office but ja I know multi sector:

Interviewer 2: yes thank you. So uhhh as a partner 05 has some **part involvement** in this program? Or not? This multi sectoral nutrition **program is there.** so as a multi sectoral program this partner as a partner your 05 program has involvement Or not?

Interviewee: uuhja we involved we have uhhhh members in orriginal multisectoral platform. There is a platform that is now active or not. I don’t know.

Nana: **What is the name of the platform?**

Interviewee: the name of the platform is what uuuuh **some scaling up nutrition.** civil society civil society. Ethiopia civil society is scaling up nutrition platform **ተብሎ የተቋቋመ ነበረ ባይዘ ወይ.. (teblo Yetekokame nebere by the way)**

Interviewer 2: ja platform

Interviewee: it means that there is a platform

Interviewer 1: hmmm

Interviewee: that uhhhh is organized by uuuuh world **vision leading** with regional health office Ethiopia civil society **collision** On scaling up nutrition. On Things like that. Due to the structure uuuh splitting.

Interviewer 2: region splitting.

Interviewee: region splitting into two. After that that level we are not active that much but we have the attended and we also have the plan and also a meeting **scheduled** with all the sectors all the **partners submit** their nutrition activity. That nutrition activity merged together. And we'll have the follow up and also the **review session** something that that like that.

Interviewer 1: I don't understand something. Regions got split?

Interviewer 2: it means that before some years ago sidama was not separate region

Interviewer 1: oh yesss

Interviewer: it was southern nations nationalities and peoples regional state together but later on sidama separate and make it it own region. And then there is another region. That is what the platform was or so is it did that something different from multi sectoral issue?

Interviewee: uuuuh not different. It Will basically was based on multisectoral

Interviewer: based on but it is separate different activities.

Interviewee: yeah

Interviewer: so maybe even if it is not exactly what multisectoral nutrition program but you told me that with agriculture with health and with uuuh?

Interviewee: lifestock

Interviewer: livestock you have some multisectoral aspect of activities. What is your particular **and picilar** Activities that you do because of this? Is there something different that you already introduced as in the beginning. What you do in the rural area what you do in urban earea? So when you work with this partner sector with health with livestock and with agriculture, is there some targeted activities in your office?

Interviewee: yeah uhhh when we work with healt office weee, we train health extention workers we provide training to health extention workers towards nutrition sensitive agricultural intervention. And also diet diversification and food preparation we provide training to the health extension workers. Uuuuuoohhh also for the livestock and agriculture we provide similar training for DS and uhhh woreda extension agriculture extension workers.

Interviewer 2: DS stands for?

Interviewee: development **agents.** that is the kebele agriculture **extension.** Yeah like extension workers.

Interviewer: okey

Interviewee: they **serv**e at kebele levels. So that we provide for them with this training and they cascade in the community in the village and also for their client also**.**

Interviewer: thank you very much. interviewee, thank you very much. But let me understand the **may be** Uhhh I already understand the role and responsibility of your office and in respect of the multisectoral nutrition activities. So uhhh maybe can you tell me the common challenge you are facing now uuuuw here now you conduct these activities? with respect to the ownership issue of the program or with respect to the commitment in respect to community attention government political. Can you tell me the challenges that you commonly face.

Interviewee:

Yeah regarding the uhhh government sector. The theeee commitment challenge and olso theeeee problem taking theeeee activity as their own activity or ownership ownership they consider the activity as other partners activity or some organizational activity. They consider Like that. There is a lack of ownership also lack of commitment anduhhh uhhhh even if there is uhhhhh gap in uuuuh including the nutrition activity at there own **annual or sector** annual plans. That is also the main gap. So theee from the government side the commitment is gap of ownership and also theuhhh uh nutrition sensitive agriculture uhhh plan including uhhh nutrition activity in the sectorial plan that is the gap and when you go to the community the awareness gap, skill gap, knowledge gap to all this uhhh to wards diet diversification gap, improving nutrition practice. The common gap is that. Uuuuuh and uhhhhhh regarding the **higher** official or political leaders the uhhhh mainly the time shortage and the workload and also there asl they are too busy. And uhh political commitment for that matter they have no chance to here about this uuuh nutrition activities. Even if they attend a nutrition workshop they will not follow, they will not monitor it based on that that uuuhhhh the challenge the problem the partners the government partners.

Interviewer: yes thank you very much. But what about other challenges like uhhh there are specific activities **in your own plan?** And you have plan with the community? With the government? You **have strategic plan** maybe a strategic plan or nutrition but when you operate specific activities what that common challenge I don't know how you. You might have some…

Interviewer 1: can the door maybe be closed?

Interviewee: heyy, heyy, heyy, calling his friend to close the door (soda mix language).

Yess you can! Close it, close it

heyy: okay haha

Interviewer: So theee would you tell me that specific activities related challenge that you are maybe. So you told us very general point from the government side from the community side so on but what are the specific challenges you have when you conduct your daily activities based on your plan?

Interviewee:

I think it is uuuh very difficult to uuuuh to identify the problems because we uhh have our plan and we have **budgets** If we invite the partners for training they will come and they will attend. After that what is the regarding that activity it was a problem doing that assignment and and ands uhh in **doing that assignment in depth. That lacks.** That maybe we provide training for frontline workers. After that they have to conduct at **grassroot levels.** But ath the implementation **level, their gap.** And they lose it or they miss it that activity when we cascade from kebele to village level there is a gap that is a implementation gap or the implementation problem.

Interviewer 1: isn’t it also important that we focus on like not only the challenges of the implementation of the activities itself but the challenges of the implementation of the activities while your collaberatimg with the other offices. Like don you do the s. Like is there overlap or do you do the same things twice which is like not efficient so there would be more work. Or is there a good collaboration and you uh fill each other’s uh yeah you build on each other or how is the collaboration between the offices while you implement activities that are multisectoral with the other offices that are included like health.

Interviewee: yeah okay uhhh when we implement the activity we uhhh we **integrate** with the sectors. but there is overlap of activity among the partners. Not with the government organizations but among the partners the activity overlap. For example the activity which we provide the activity which SNV implement and then on the other side also **world vision** or uhhh

**Interviewer: with 06**

Interviewee: 06 also implement that activity. There is a overlap of activity But we work with the government we will work in collaboration and integration. We share our plan to them and we integrate with their plans.

Interviewee : yess ah excellent. I think you told us very nice point like implementation problem like the community does not well understand.

Interviewee: hmm.

Interviewee: even those who took a training they have a supervision **…… 27:45** when they practice they face the challenge **….27:48** problem. Activity overlap is a problem but you told us that **… 27:54** of collaboration is not a problem. Okee. What about the structure? Do you have a clear structure that promotes nitritional problem improvement? What does it look like?

Interviewee: yes actually we use the government partners. as region here is nutritional advisor. At woreda level one project officer, project officer maybe not for one woreda but for two woreda one project officer. That project officer will uuuh primarily uhhhh activity of the project officer is what the daily activity That means the dailyextention activity. As additional assignment we keep the assignment for that uhh officer to follow the activity. But the **main implementers are** the government for us. We have uhhh at regional level there is nutrition person. At woreda level there is no nutrition person but the whole project representative are there. They have their uhhhhh primary activities that will be focused on dairy extention activity. Because of that we will work with government partners and we will uuuh give the the assignment to the government partners. Ja.

Interviewee: Yesss thank you very much. Uh maybe the one thing more I want to understand is what is the challenge from the community you faced. Not only those who health extention workers or **IGA** Something. But what is the challenge from community side you're facing? Because you aimed to improve the lives of community but still I think there might be some challenge. What is that?

Interviewer 1: like is there any resistance from the community?

Interviewee: So regarding the community the maybe the challenges uhhhh income or economic status. To access this program we focus on six food groups and that uhhh there is the six food groups that make staples also the animal source foods uuuuh fruits vegetables hmmm the ones with the cereals and also the uhhhh **fats and oils** . We focus on these six food groups so to access all this they have the economic challenge also the accessibility of food items. Since they are at rural areas. For example they lack **iodized sat,** They lack fruits in every corners of market. They lack that so the community challenge for the community accessing food is economic status that is the challenge of the community. Maybe I you understand.

Interviewer: yes exactly. Yes that is a very nice point. Uuuuhm i don't know what the **broad activity of** 05 but what I raised here is your program in relation to the budget. Maybe this nutrition program had to be the one uh many activities of 05

Interviewee: yeaha yeaha, yeha, yeha.

Interviewer: But what is a challenge in relation to the budget? Particularly nutritional program and I am very glad to hear that your focus is on pregnant women and lactating women to improve their life. And tell me some challenges.

Interviewee: yeah regarding the project we have our project is bridge. There is a lot of projects that we are working on bridge project that means building rural income iincome and dairy buisoness growth in Ethiopia the project is that.

Interviewer: this Bridge is a abbreviation?

Interviewee: abbreviation.

Interviewer: what it stands for B?

Interviewee: building Rural Income and dairy business growth in ethiopia. Building Rural Income through inclusive Dairy Business Growth in Ethiopia. Building Rural Income through inclusive Dairy Business growth in Ethiopia)

Interviewer: okay.

Interviewer 2: D is for dairy?

Interviewee: dairy yeah.

Interviewer 2: ah okay

Interviewee: Dairy business growth in Ethiopia

Interviewer 1 and 2: okay.

Interviewee: so bridge is that. Bridge has about four uhhhh goals. The first one is uhhh increasing production productivity of dairy. The second is what thuhhhh market dairy market value chain. The third is nutrition that is improving the nutritious food consumption the third one is this. The fourth is thuhhh improving the **enabling** environment. From that we are at uuuh specif or at objective uhhhh three improving nutritious **consumption**

Interviewer: hmmm that is a point what does is relate budget challenge in relation to nutrition?

Interviewee: uhhhin relation to nutrition we compare with the other outcomes theee budgets **allocation** to nutrition is relatively low. That means uhhhhh the first outcome take the thuhhhhh t the highest one the first outcome take the highest one the second one is lack but **specifically** when we plan we focus based on our budget the budget that will be assigned for nutrition so we prepare our plan based on that the given budget. So our our baseline is what we have decided amounts of **birr** or budget. We plan based on that activity.

Interviewer 2: and where from do you get your budget? Like government or only self uh charity budgets or?

Interviewer 1: yes she wants the source of budget.

Interviewee: yes our donor is SNV which is a Nederland embassy.

Interviewer1: Dutch Embassy?.

Interviewee: yes Dutch embassy. So ’the bridge is also for 5 year project. We’re now at at final year or At the end year.

Interviewer: This year?

Interviewee: yeah so our budget source is dutch embassy.

Interviewer: yes, what about the challenge and relation to the resources human recourses other recourse in addition to budget.

Interviewee: yeahhh I don’t think **ኢንግድህ፡ ማለት ከሰዉ ሃይል ጋር ተያይዞ የነርሱ አሰራራቸዉ ብዙ ጊዜ በጥቅት ሰዉ ስራ ማሰራት ይፈልጋሉ፡ የራሳቸዉ አካሄድ ነዉ። (Engidih malet kesewu haile gar teyayzo yenersu aserarachew bizu gize betikit sewu sira maserat yifelgalu yerasachew akahed new). (I think in regards to human resource, their trend is they want to conduct work with small human resources. This is their principles**

Interviewer: you will understand because of the thuhhhh amount

Interviewee: yeah regarding te scope of the project the scope there is a limitation for example we have uhhhh work more than 10 woredas at sidama region. Even we now we scale up to the south. In the south we have working with **wolita** uhhhh one of the zones of the south region. So when you look the scope of the project there is a limitation in **human power.** Do that is theuhhh uhhh thuhhhh the the the plans of the project a project will be based on that if their assumption is working uh with a limited number of person uhh that is uh that is focused on based on their uhhhh proposal uh their plan i mean that it is focus on their plans but there is a shortage we have no nutrition representatives at woreda level or at **zone** Level. That is a challenge in regarding as as in regarding our project.

Interviewer: yea

Interviewee: soooo uh so this is all about I have.

Interviewer: maybe the one point is how the uhhh professionals are capacitated in this area. With their knowledge and how they are skilled up in training opportunity workshop**?? 37:44** opportunity **anything/ may be.**

Interviewee: for the internal stuff or for the project staff?

Interviewer: yes for the particularly for those work on nutrition program how they are updated or **capacitated?,**

Interviewee: uuuuuuhhhh so far uhhhhf different online training will be prepared for us for example hmmmmm hmm in 2020 June we have uhhh the nutrition advisors at national level this project provided online training from wageningen university. Andddd

Interviewer: what university?

Interviewee: wageningen university.

Interviewer: that is in netherland?

Interviewee: yes netherland. They have the online program In Dairy health and nutrition area. So they provided online training. Most of the time they give online training online training regarding that also the year once there is annual workshop project at a workshop like that. But uhhh not that much the professional training is not that much.

Interviewer: not common?

Interviewee: yeaha

Interviewer: so where there is some consultant workshops?

Interviewee: yeah we have a lot of consultant workshops with the partners uhhhhh we work also withWUR university Wageningen uhhh research team there is uhhhh at national level we have uhhh WUR Wageningen University Research team they have different rearches in area of nutrition **on** dairy and animal nutrition. So we also we attend in different national uhhh workshops like the uhhh **ISAP** conferences. **Maybe last year** Uhhh This year I think it will be prepared at Hawassa University **Ethiopia animal science animal science society members** conference.

Interviewer: yeah

Interviewee: different types of workshop.

Interviewer: workshops. Yes already you said about the uhhh platform that is uhh another nutrition related platform in addition to the multisectoral nutrition program and I understand that you are a responsible person to coordinate the nutrition program.

Interviewee: yeah

Interviewer: and uhh your commitment I think is very nice **maybe** uhhh you have operational and a strategic plan?

Interviewee: we have operational

Plans. Uhhhh the plan B is **annual base**

Interviewer 2: annual base?

Interviewee: annual base. We have operational plans. And the Strategic plan will be from the project **managers side**

Interviewer: Interviewer from a program coordinator you have no strategic plan?

Interviewee: yeah

Interviewer.: yess maybe in general I wanted to hear from you one general concept that what are the opportunities that you can mention? Uhhh There are opportunities with respect to structure, opportunities from the communities opportunities from culture. Different aspects which helps this nutrition problem to be solved and this nutrition problem can be achieved if we use this opportunity. What from you experience could you tell me about this?

Interviewee: yeah from the beginning if we take from the government side uhhh there is uhhh very high commitment from the government side toooo to reduce malnutrition to reduce stunting. A lot of policies, a lot of strategies and programs designed .That is the uhhh opportunity from the government side. If we take there is **national nutrition plan** One after one uhhh the government continued to the uhh after that there is uuuuh national nutrition there is

food and nutrition policies also degigned form the government side nutrition sensitive agriculture strategy. A lot of policies and strategies are there but that policy and strategy is not implemented accordingly or in in in designed manner in designed manner or in there based on their projects**.** Or based on their **GOD(???)** it is not implemented but thuuuuu commitment from the Government is good. Uuuuw what another opportunity in regarding here is sidama it is very green and also the culture is very good to reduce malnutrition. That means in sidama most of the household consume dairy products but not the whole milk. They use mostly the skimmed milk. There is a potential in the products potential fruits potential vegetables now that will not be uhh consumed well and most of the time they focus on **marketing** And **selling** so uhhhh improving their skills and their knowledge towards **diet diversification** And the consumption of dairy products and also consumption of fruits and vegetables. That is a good opportunity the **agroecology** is also a good opportunity of this sidama region also the south region also **agroecology** is good. So uhhhh **from the** community sides that is the good opportunity? And from the partners there is also a lot of uhmmmm nongovernmental organizations which work on nutrition. Mainly uhhh save the children uhhhh work very well also **world vision work** in this area. And so the rural working of sidama regions. So uhh strengthening this multi nutrition program especifically integrating this partners in to one **objective goals** is a good opportunity to reduce malnutrition I think.

Interviewer: yes

Interviewer 2: I have a question. Uhh Then would you also think that implementing even more multisectoral approach would you see that also as an opportunity? To make it wider and include more sectors so that the multisectoral approach would be more..

Interviewee: yes it could uhhhh it is a good opportunity integrating multisectoral into one for example Uhum uhhhh if we take lifestock sector they work on animal production if we take agriculture sector they work on increasing the production and productivity of uuuh cereals, fruits, vegitables, when you go to health sector basically they work on health interventions. If we integrate all this sector together it will have a positive uhhh outcome uuuh to reduce the malnutrition.

So far it is tested but not uhhh **strengthed** So far there is uhhh uhhh startup there is startup uh multisectoral program but not uhhhh not well understand understood by the governments and by the partners so strengthening the multisectoral nutrition program is a good opportunity to reduce malnutrition.

Interviewer: really I take this point as a strategy to improve nutrition program more than the opportunity. Because the best recommendation is strengthening this partner and government integration this is very important. So thank you very much. Maybe you say that this d**ay** uhh the government decided you say on pregnant women and lactating women we focus the nutrition program?

Interviewee: uhhhhhh in government **side?** in health sector there is for example pregnant women **forum** that is uhhhh the health office working with health extension workers they uhhhh only focus on pregnant and lactating ones. They participate them innn uhhh I know that there is

a pregnant and lactating women forum like that they will uhhh they will invite all them together and they will discuss on nutrition activity and their feeding practice like that activity they have. The other is what uuuw women or **የጤና ሰራዊቲ ተብሎም**

Interviewer፡ Health volunters?

Uuuh from the health sector side from the agriculture side there is **no** separate.

Interviewer: yess. Already that the sakota something like programs are focused on the stunting of the children to avoid uhhh that actually initiation for pregnant women forumuhmmm and others that is very nice. Then lastly maybe uhh hmm as a recommendation or as a strategy recommendation what is the best like you already mentioned something as I understand that as a **strategy** but because you have a number of **exposurein** nutritional activities and to avoid this to minimize or **to reduce** This challenge what is the best strategy you **think??** that the multisectoral nutrition program should follow? Already you know there are strategies they follow. There are around 6 strategies they know that this multisectoral nutrition program follows including from the community **lab!** at community level then at district and region and then at national level including **steering commmittee** or something. So from your experience what you recommend the best strategy to work and to include and to **Work with it**

Interviewee: yeahhh uh already said **previously,** The main thing is **what?** Integrating the nutrition activity in each and every multisector annual plans. That means there is a lot off multi sectors which will be involved in nutrition implementation. Uhhh we are only saying three but there is women **youth And child affairs** they have intervention and nutrition activity. They work with women **empowerment** uhhh one of the nutrition uuuh pathway. So integrating the nutrition activity with the multisectoral annual plan that so good thing. The other thing is what uhhhh really institutionalizing this the multi sector nutrition program at the government sector uhhhhhh if you got if you find it stunting is so far increasing not reducing but this year it also is nutritional status data **needed** so so far it is not reducing it is increasing For that matter what a lot of programs are there a lot of **government** sectors working in this area but the **main** Thing is what institutionalizing this nutrition activity in the sectors is a good uhhhh a good way to reduce this nutrition malnutrition problem.

Interviewer 2: nutrition system thinking.

Interviewee: yeah. This is just system **thinking** and also budgeting nutrition activity uhhhhh in government **side** there is no budget allocated for nutrition. At woreda at **zone level** there is no specific budget for nutrition so also budgeting nutrition activity uhhhh this is critical. I think besides that the strengthening the multisectoral nutrition activity or multisector nutrition program uhhhh engaging all uhhh relevant **stakeholders** in nutrition activity and also uhh standing or strengthening them for this implementation is that what I think.

Interviewer: yeah thank you very much. Most I have **done** my questions. Is there any concern you would rise that we didn't raise or anything that you would add?

Interviewee: nothing from my side I think I said all what I have.

Interviewer: thank you. Julie do you have a question

Interviewer 1: uh no

Interviewer: thank you very much uuub we may be back to wordt with you. Based on our findings we will invite you for preliminary findings of our study. Thank you very much.

Interviewer 1: thank you. Amesegnalehu.

Interviewee: buna tetu, coffee?

## Interview 9 – 06

Transcriber: interviewee 1

Interviewee/R1: R1

Interviewer/I1: I1

Observer/I2: I2

**Section 1: Socio-demographic characteristics of the participants**

1. Worada/district/partner/region office name: 06
2. Sex: p02
3. Age: 56
4. Marital status: Married
5. Professions: health professional and public health professional
6. Work experiences: 18-19 years

**Interview**

I1: can we proceed?

R1: yes

I2: Thank you

I1: yes, she is my research partner. She works on this areas and uhh the area that we are doing is multi-sectorial nutrition programs, particular issue on maternal aspect, nutritional problem. And the big point that we want to assess is challenge and opportunity to deliver this program. Special this multisectoral program has some deliver or implementational challenges we suspect. So what are the common challenges, what opportunities and then what is the best strategy that helps to deliver this program. This is the three important that we will ask you one by one. So, still, I would like to appreciate your time because you have very busy schedule and you are still asking you to give me some more time, and finally you are okey to give us very important information. So, let you start with your detail, some introduction part of yourself. About your age, your marital status, your profession, your work experience here and what particular profession you have been working. Let you give us some brief introduction about yourself.

R1: okey!

R1: and uhh concerning, thank you, with regard to my educational background, I am health professional and public health professional. Uhhh and general nurse, graduated from blacklion. And then after I joined Civil service University with BA degree in development management. Then after I joined, I received master of rural development focusing on rural healthcare. And uhh specially area was uhh rural healthcare. Then I joined pharmacology and I graduated with mph, Master of Public Health. Again, my third master's is MBA, business and, Business Administration focused on non-profiting organizations. And uh this all is my educational background and work experience.

I1: thank you very much. Really, this is a very wonderful profile of your personality. I liked your family story, your family success, it's a blessed family. And it's a wonderful experience in this program. We are lucky to get such important issues. So, I, just uhh, saying this I would like to hear from you about nutritional problem, in this particular Sidama region, and in southern region, you can say something but our focus is what the nutritional problem looks like. Cause you know, the nutritional problem, in children, in pregnant women, and lactating women, what this looks like.

R1: okey, thank you. The first one, and the first and the most problem of nutrition, not only in Sidama, in general the country, is the problem of awareness. For instance when you come to children and mothers, the major problem is stunting. But the community do not know what stunting means. And the community or the mothers don't know how stunting will affect their children and how the stunted child became for the coming future and which was the compromised child. Therefore, the first one is the uhh awareness problem of understanding of stunting or the problem of nutrition, which affects their childs their family as well as the generation as whole.
The second problem uhh for nutrition is, nutrition, uhh we can say that you do have different policies, programs, strategies for nutrition. But that was not uhh well-orchestrated with the activity which was managed or led by the politicians. And nutrition is not one of the agenda for the politicians or for higher leaders. Because nutrition, uh there are the attitude towards nutrition, nutrition would be considered as the one sector, or the sector for health only. And the impact of nutrition is not well uh known. Even though if the problem, uhh, some for instance the health professionals or some trained or educated uh people knows what the problem means, but the major problem for that one is that there's no behavioral change to practice what has been known and what the impact for children as well as for the family. For instance, uh, one uh individual or one who have money may not bring or may not think about the nutritious food for his family just rather than just bringing the uhh meat or some other things only.

Therefore, the first one is uhh the understanding problem, the awareness problem, and there's no practice or there's no behavioral change, even on those who knows or even on those who meant to be educated. Yeah.

I1: Yeah, excellent, that's really nice. So, you mean that as the high nutritional

R1: yeah.

I1: high nutritional problem associated with awareness as well as uh the behavioral change problem. That is the reason why it is high.

R1: yeah.

I1: excellent. So…

R1: now again, the nutrition is not agenda uhh

I1: for the …

R1: not for as major agenda, as it impacts the generation.

I1: so, they didn't give big attention for the nutrition.

R1: yeah, no attention at all.

I1: excellent. So, this actual problem of pregnant women. Is it common or is not common, nutritional problem? Is it common in pregnant women or not?

R1: it is very common.

I1: very common.

R1: it is very common, because the uhh it is uhh what matters is the pregnancy. The first 1000 days, that means starting from the day of pregnancy and until the child gets two years. This is the first 1000 days period.

I1: yeah, yes.

R1: this impacts a lot. This decides the future generation of the child.

I1: exactly.

R1: this decides the future generation of the mother to be, an adolescent girl. This decides the problem or the good faith of the nation. Therefore the pregnancy part is very very important matter just to manage nutrition.

I1: exactly. Very great ... of course.

R1: yeah.

I1: so, how is the trend looks like? Is it getting worst or getting better? How it's look like, the problem?

R1: you know uuuh this has to be under some investigation or uh research just to give …

I1: from … observation

R1: to complete. But, as one of the publicate professional in rural part nutrition, it is not well organized or it is not well coordinated. At the problem uh being magnified, the coordination is not well organized. There are different opportunities, the media, just uhh, when you come to Sidama being the central region uh in Ethiopia. There are different opportunities, different NGO's, different education centers, different medias, different mass medias. Just to have different understanding of nutrition. But, as far the problem become bigger, the coordination the solution is not as compared to the problem. Therefore, just uhh, you can observe that the mother uhh understand what nutrition problems, from media or from different health facilities. But the practical part is the problem. One, due to the understanding, the second one is the living conditions. The living conditions also matters. But, uhh, just …, compared the living condition the problem, with the understanding more, the understanding problem, is bigger. One mother can sell the egg from her home and bring uhh biscuit or some other things uh for her kid. This is a problem of understanding. She can sell avocado, and she can pay unnecessary uhh not nutritional value food from the market. And the understanding gap is still there. Not well organized, uhh not well understood by the community level.

I1: yeah, yeah, yeah, exactly. So the trend is still getting lot of worriest, still there.

R1: yeah.

I1: so, one of the project or one of the suggestion that already started is, to solve this problem, is that multi sectorial nutrition program. You heard about this, and you already implemented this program.

R1: yeah.

I1: and your office has also some role in this area. Tell me about it, maybe what is your role, what is your activity, with respect to this multi sectorial malnutrition program.

R1: yeah, okey. Thank you very much. As far as the program is multi-faceted, it needs multi sectorial approach or multi sectorial performance, just to tackle the problem. For this, there are different uh agents that are, there are different stakeholders, there are different policies.

I1: yes, yes.

R1: let's start, start at 2008, the Ethiopian National Nutrition Program was revised, in 2008. For three years, from 2008 up to 2011, the first one was..

I1: that in Ethiopian calender.

R1: in Ethiopian.. no in uh Gregorian calender

I1: oh okey

R1: uhh then after, the uhh national nutrition, the first national nutrition program was revised uhh until 2011, 16. That was the major center for that one multi sectorial coordination. because, there are different sectors, nine sectors during that period, all nine sectors have their own roles, their own responsibilities, their own strategy just to tackle the problem of nutrition. And to tackle the stunting programs, special focusing on the first 1000 days. During that time uh there are uh all nine sectors uh member for multi sectoral formation. Then after, the uh national nutrition II was launched at uh 2020, then during that time also the major call center for uh the national nutrition program was multi sectorial coordination. There are different sectors. There are two committee, the multi sectorial coordination body and multisectorial coordination technical committee. The head of the bureau, or the head of the sector, he's the member for uuuh multisectorial coordination body. And the nutrition focal person or the technical one was the technical committee member, during the first national nutrition program. Again, last trend continued for second national nutrition program, including the nutrition integrating sectors, for instance Save the Children was one of the number for multisectorial coordination body. And Hawassa University was one of the number for this part. And, again, uhh the food and nutrition policy was announced and different policies and different strategies was announced an launched at national level. And that was cascated through the grassroot to the woreda level to the community level. And those multisectorial coordination establishment also went to the community level to the kebele level. For instance, during our ENGINE implementation period, we have established kebele level nutrition multisectoral coordination, at kebele level. Again, during the GTM also we did that one. And currently those all nutrition multisectoral

coordination body, uh is not as expected as the policy said. Because, the first one is the, it was not led by the president, during the first one. Then through the revolution of the first national nutrition program, what we presented from our practical experience, was just that uh the prime minister has uhh to uhh the role to lead the national nutrition program at national level. And at regional level also, the regional president need to lead that one. If the national nutrition program expected to be effective. That was then from the second one. But uh it was not satisfactory. And it was better than the previous one but no..

I1: still there is a problem.

R1: still there is a problem. The major challenge for that one is that, the first one is the understanding barrier . Because they uh the most of them did not understood what the problem, what fact of nutritional value during pregnancy, during childhood food, during the first 1000 days. They understood, they hear from media, but there's no interest for that one. The second problem for multi sectoral coordination is that leaders do not consider as their one of their political agenda. For instance, if you say uh, if you wanted to have uh prone the cadra or the sector phase for uh some other activities. They'll work

I1: they didn't …

R1: they work at night. For instance the nutrition that is from the training. That is one of the major problem. The second one is, the thirds one is the high turn-on of the staff. For the staff we have conducted trainings, on capacity trainings, on the roles and responsibilities on the issues of scorecard. You know the scorecard?

I1: yes, yes, exactly.

R1: it has conducted a training on scorecard just to manage, to know, to value themselves. Uhh every uh sector had their own role, their own responsibilities, that has to be checked using their scorecard. What has then been well, then what has been less, and what was not them. Just shown by yellow, green and red color. Just uhhh we conducted uh different trainings together with such multisectoral organization body numbers and uh Hawassa University. That was noted such became fruitful. And the fourth problem is the issue of budget. The nutrition sector, or the uhh, each sector nutrition focal person was professional, and some uh sectors assigned budget some not. That is the major problem for nutrition aspect. And that is the major problem for multisectoral coordination of the nutrition. Just to be effective as the planning, as the policies and different coordination strategies developed by the national level.

I1: yeah, thank you very much. This is a great and deep knowledge about nutritional issues, specially the multisectorial issues. So, because of the interest of time let me come to the leader, to the major point. To say that, thank you very much. You tell that the great problems from the starting of this nutritional program and the policy and the nation and even to the regional level, but let us come to your office, please. Let you come to your office.

R1: yes

I1: there's some concern about this the multi sectoral nutritional issues. And, if yes, then what that specific activity that you involve and come to good because of this multisectorial nutrition program. Because there's the government, there's the partners, who involved in this multisectoral program. Is there involvement of this partners like Save the Children involvement to the multisectoral program? What are their role, what are their responsibilities in that aspect?

R1: yeah. Just to have played the launchshare of multisectoral progression at regional level. The first one, you have the number of multisectoral coordination. We have conducted capacity training for uh uh technical workers. And we have conducted different revolute things with multisectoral coordination at regional level as well as zonal level. We established Save the Children. We established multisectoral coordination for the rural level, and we reviewed and uh we established at woreda as well as kebele level.

The other point which we have contributed that's just with regard to nutrition. Uh most of uh projects are nutrition focused. For instance, currently, we are uh working on education. Uh on education we are providing supplementary feeding programs for school education, school children. Because, one of the high dropout rates at the school level is the problem of nutrition. Therefore, considering that one, 06 developed proposal and good budget from work bank, from global partnership for education.

R1: yes, we have been there. It is very drought prone area.

I1: it was started in 2003 the drought started there, it is still there. Because there is no river or some other things. And uh, water started from one area, but not yet infected. There were high demands of nutrition there. And were working for just the last two years. And the dropout was highly reduced, the uhh number of schools education was highly increased by 234. And other were working at, we are working with uh uh

LEAP project, it is the empowerment of women. On empowerment of women one of the criteria of that one is just the vulnerable household.

I1: vulnerable, okey.

R1: yeah, vulnerable household, special women. And uh we will select that one from community level through the woreda through the kebele and we'll post the selection criteria everywhere just to know who is vulnerable than the other one. Then after we make the beneficiary, then we talked them that, and we have been conducting different trainings. And the four steps trainings, the ladies training, then after, being your own boss. The first one. Then after, if a woman became being her own boss, we'll provide her sort amount of money and we'll give them microfinance. And most of them are engaged in different business. There are many women who engaged in uh different business in group, as well as individual level. And those, the major problem is that the problem of family, the problem of feeding them that linked to the nutrition. And we are supporting the mother, we are supporting their children, we are supporting their household. The other one also, we just will have one project on employment. Those also have the same trend, and we uh at the end of the day, we engage on business, and they support their family and that's something that will be supported by their nutrition. And again, our other project is on child protection. Also, that one is focused on IDP, the major problem of IDP, especially we are working that. The IDPs came from other area, and just are uh hosted in host community, uh IDP means Internally Displaced Person.

I1: yeah, internally displaced …

R1: they are hosted in host community. And they don't have uh home, they don't have what to eat, therefore we are providing them. We'll provide 60900 Ethiopian Birr for per household. We are supporting them to have their uh daily food, their family. This all are working good, we are implementing that at community level.

I1: oh, great. I was wondering to hear about uh the women issues uh in 06. I understood 06 only works on the children, and then to they heal and even been reading on the post that

R1: yeah

I1: respect for the women and the girl, that is posted on. And I'm very happy to hear. Would you say something more about the women could become boss of themselves.

R1: yeah

I1: what does it mean? Maybe that piece, I wanted to hear more.

R1: yeah, that means we have four steps

I1: to empower women

R1: to empower women. Then, the last one was 'women, be your own boss'.

I1: what does it mean?

R1: 'be your own boss' means the particular women who was selected by the project and who have received different trainings from the project, then she can, decide she can make herself as well capacitated, as well empowered woman. Then we'll link that woman, just uh we provide her sort of money. Then they'll save their money with uh microfinance. Then after, they can have access, uh from the start we'll have different ample experience in that one. For instance, in one group around nine women

I1: in one group

R1: in one group. That can be individuals, that can be a group that can be three, it can be four or five. Those five women or nine, uhh they'll get money from 06. This is a private donor. Those donations goes to women, then they can for instance, many of them, many group are engaged in minimal paid, in a café, a soil production, and meta work, and wood work, and hair dresser, and animal, small sheep, shepherd. And then after, they deposit their money and microfinance, and have money. More than what we expected, something till they have deposited around 300,000 Ethiopian birr. Just from the scratch. That made them by just being their own boss, they have a kid, they empower themselves not just themselves. They empower their house, their home. And then after, what we are taken then are all those woman groups, or individuals who became their own boss can integrate and can make community. Then the community will be changed and become self-sufficient.

I1: that is why a very, very nice program. The woman will be self-reliant, they can manage herself, their family and she will be boss for life.

R1: yeah.

I1: wow great. The other point when you conduct these activities, these projects to the women, to the children, about nutritional self-reliance, what are the common challenge that you face?

R1: *hhhh…*

I1: Tell me, thinking from the participant review meeting, when you give training to capacitate, when you give money to empower, a number of activities you have conducted. What are the common challenges that you, you can raise, please.

R1: yeah, uh you know uh the major problem is understanding.

I1: awareness.

R1: yeah, awareness. That's the major problem. Had it been known as if everyone is poor, had it been recognized as if they can work as individual?

I1: is understanding the problem at community or politician or the health workers?

R1: yeah.

I1: which one?

R1: the first one is, if the leader made good decision, the community can change themselves. Due to different or restricting activities the leaders may not focus on this nutrition issues. The second one is the high term of turn-on of government staff. For example, who provide the training for one sector. After uh a couple of months, when we went there we not find that one. The one who trained for uh different activities. The high turn on of the staff, the trained staff. High change of the staff, it is a major problem. The other problem is a budget issue. The budget shortage, you can find that different activities many more

demanding. Community, but the budget which we have that limited. And uh the coordination issue is the major problem.

I1: is the coordination with the government sector?

R1: yeah uh just from the staff we do have a government, and this is all coordination. That has to be strengthened. We'll have at regional level strong, that uhh should go to uhh the grassroot level, to donor, to woreda, to community level. As it, it strong at regional level, the changes become loser and loser.

I1: when it goes down.

R1: when it goes down, yeah. That's a major problem. The coordination, the coordination uh not only with partners, the coordination of sectors, towards one point, has to be strengthened, that is not assigned strong enough. As the problem is very high, the thing goes the action has to be as well as the problem. That, there's discrepancy over there. And uh I think this is all.

I1: this are the challenges. The awareness challenge, financial challenge, coordination challenge

R1: yeah, the high turn on number of the staff.

I1: staff. yeah, excellent, thank you very much. Maybe uhh we have raised about the challenges and not more this but uhhh tell me maybe the opportunities, the uhh that is very important to achieve this program. To minimize nutritional problem, one very important strategies, multisectoral collaboration and there's challenges, but there is also opportunities that we to use. If we use that opportunity, we can deliver. So, what about that potential opportunity you can bring.

R1: you know, the first and the most opportunity is here being at the central level. For instance, if you come to Sidama, the Sidama is integrated in the center of the country. The weather condition, the peace issue, whatever you can say, this is a good opportunity for Sidama. Particularly, if your research is for Sidama

I1: yes of course

R1: it is a central region. There are different cash crops? Compared to the other one. There are different fruits, vegetables and different fruit types for nutrition. But, the other opportunity is that just uh uh the media. You can get different Medias which can propagate the advantage of nutrition. The opportunity of nutrition in different areas, be it uh mass media different opportunity and the coverage is very high. The other uh the third great opportunity, there are different partners who are working with the region. Be it uh development partner, humanitarian, local partner, or international, there are many international UN agencies and local partners. Those are the major opportunity for the region. If coordinated well, if the multisectoral coordination becomes strong the opportunity is very high, uh even more than the other regions. The peacefulness of the region is one of the attractive opportunity for the partners. The other thing is that there are different educator individuals. Uh the uh people are uh more of educated and uh just uh you can tell properity to the other one. You can say they supplies, who knows what is good and what is bad.

I1: yeah.

R1: all these opportunities will lead us to the good multisectoral coordination, to good impact of nutrition, if we utilize these nutrition uh opportunities well.

I1: yeah, that's very nice. One point maybe what uh with regard to the culture of society of communities, is there some that is very helpful to this project. The uhh I heard from the some PSI workers and we have interviewed one and they say people conduct some open defication there is somewhat proverbss which say uhh

*የሚሰራ ኢያለ፤ የሚያይ ያፍራል* (
'Yemisera iyale Yemiyay yafiral'

That is something when someone deficate on the field, so the one who is doing, instead of the one who doing, the one who watch felt shame on that expected. So, this is some problems. Is there some culture which helpful for to improve, to implement this project? Multisectoral aspect, with respect to community culture.

R1: yeah. I just to look into this the cul, there's no culture in this multisectoral coordination, rather when you come to the nutrition, not the culture, there's the trained for tradition. Just uh the you can say tradition, the tradition is more relevant for this particular activity. Just uh I did my MPH on the first 1000

days and the impact of stunting, during the pregnancy and during the lactating period. One of my questions was uh whether there is uh traditional issues which hinder the mother not to eat. And the uh, tradition which the child not to have sucking the breast. Uhh it is that most of the community currently knows the advantage of uhh, what it is called, the cholesterol. Previously cholesterol was not faced by the child, but uh currently that one is very improved. And uh the other thing that the mother didn't allowed to have butter food in the community because that is the tradition. Because that the fetus and the mother will get fatter and fatter and the mother couldn't able to give birth normal. But that was not the case now. The tradition is improving, thanks to this religions and education, and mass media. And uh there's no tradition, there's no culture which hinders the multisectorial coordination. Rather that is a good opportunity to the grassroot, to the community level. They gather together, they have their own uh contribution, and uh they do some things that uh favors the community. And if we took that one as an example, the multisectoral coordination linked with that one become a great opportunity.

I1: yeah.

R1: rather than the hindering of the tradition.

I1: yes, thank you very much. Maybe my last question

R1: okey

I1: *short laugh* is uh about uh you know about the strategy towards the multisectoral, from the national to the regional to district as well as to the kebeles, there are strategies. Including like Community lab different activities, about steering, steering and technical committee, there are different strategies. And uh, can you tell me some important strategies to include more, and to avoid, what is your best strategy, your recommendation? To achieve this project, or to achieve this multisectoral nutrition program.

R1: You know, the best one is that you do have very marvelous strategy towards multi-sectorial coordination. But, that has to be workable. That has to be implemented in practice. That has to be go down to the community level. We do have uh smart food and nutrition policy, which included multi-sectorial coordination. We do have national nutrition program, we do have Seqota declaration. For instance, uh that one is a major one to make it multi-sectorial. For instance for Seqota declaration, there are different sectors, who are engaged at committee member that has to be practical, the major issues that we do have many more strategies. But, that strategy has to be unpacked and engaged into action

community level. Not only at regional level, here is that only the structure. Where the community is where uh the more people are at community woreda as well as community level. It has to be exercised well. That has to be monitored well. That has to be supervised well. Whether it has been going on what has been declared at regional level. Or uh whether the gap. If there's any gap, the capacity training has to be linked to strengthen the multi-sectorial coordination project, or uh activities with the country announced.

I1: yeah, thank you very much. Anything to say lastly, not that I'm right yet, but you wanted to say anything.

R1: first of all, thank you, just uhh for having you, and just uh this is one of the major problem. I have tried uh many uh, I1 knows that uh I have been trying to have my PhD on this area, stunting reduction related to multi-sectorial coordination. Due to the problem, due to the examiner, without my knowledge I was stopped just on the presentation day of my you know. Therefore, I'm very happy for having you on this topic and this has to be very great opportunity to bring what multi-sectorial, what the gap is, what opportunity is, how to implement this one, just make in practical the strategy of national nutrition program as well as policy. Therefore, this will be a good springboard for the community, just to have uhh reduction on stunting reduction and to save lives for many generations.

I1: thank you very much.

R1: thank you very much.

I2: thank you.

I1: do you have anything to say?

I2: uhh no I only have one question out of curiosity if that's okey. With like the empowerment intervention, with the microfinance, and uhh you know what I mean? That you give money and then the women become their own boss, did it have any disadvantages? Like were there any challenges in that intervention?

R1: yeaahh, uh so far, no challenges for that one. The problem is that uh insufficience of funding. Just to have started with very small. Then sure that one and uh community seen improvement of uh those

vulnerable household. Then that one became high, then donor gave us more money. And we started again with vulnerable women. Now, it is very demanding. The only problem is the budget issue, no challenges so far. The community, the woreda people, the regional people, the women and children are very …. on that one. And so far, no challenges for that one.

I2: that's good to hear.

R1: yeah. Thank you.

I1: yeah, thank you. Thank you very much. (All transcripts chronological order from gender office, Pos. 672-810)

## Interview IX: IDI with '08

I: Thank you very much.

R1: You are welcome.

I: Ah, great.

R1: And another thing, I can come to challenge, the challenge, directly to the challenge.

I: Yes, of course. Directly to the challenge. This is introduction part actually.

R1: Okay.

I: Regarding maybe, we will be happy if you start with the problem of nutrition. How it looks like in the context of your area where you work. So, the points that you don't touch then he will

add. And then, let me also give to him to introduce himself, directly before proceeding to the challenge maybe.

R1: Okay.

R2: My, my, my name is R2 and I am …(3:30)….

I: How long you work in this?

R2: My, still in this program, almost one year…

I: One year.

R2: …but I have twenty-three year experience in health sector.

I: Health sector.

R2: And I am married and I have four children.

I: Oh, excellent, four children. Thank you very much. I will come back. So would you tell us the nutritional problem in the regional estates. How is this, under children and under pregnant women, and the women, how it looks like. Let you start with. Because one challenge is the prevalence, high prevalence of nutrition, literature review understand that. Because you target to

minimize that problem, so, how looks like this problem and this your district and your region. And your work area, please?

R1: Very nice, it is a nice question. I like it so much. And as my position indicates, it is nutrition sensitive WASH. So, before, I was working in Southern Nations, Nationalities, and People's Regional state. There, as a WASH technical person there. That means only when we say there in the under the water bureau, water, drinking water supply and latrine construction was a major works that we did there. Drinking water supply in the region and latrine for the institutional, institutional. That means health and school latrines. And the hygiene part was not that much focused and also the nutrition part was not that much focused. And when I came to here and enrolled here as a nutrition sensitive WASH program manager, I was seriously looking at the problems that there is wider between the sectors. The multi-sectorial idea is here, I faced here. Multi-sectorial means, many sectors, health, water, agriculture, livestock, women and social affairs, and education. Jointly, they do on nutrition.

I: Yes.

R1: But before, you know, the main problem is the people in these sectors do not do. Even though the WASH there, at least four sectors join, before. When I see there, water, health, health and education and finance is the fourth sector only. And here also, only the institutional latrine is focused. And other communal latrine was also not focused.

I: That is the ignored part.

R1: The ignored part. So, when I come to here, I see the at the glance or the thing that we make magnifies the problem of nutrition. Magnified and the problem is, third sector specific, planning. In every direction. That problem is challenging us now. The sectors want to do their works

separately, they want. They, we try to come together and jointly do, but the problem that they know, their status quo is the main problem in this field. But the nutrition addresses all the sectors. If it is only twenty percent is addressed by specific that means, nutrition specific means health sector. Health sector that twenty percent, problems are solved. The rest, the other eighty percent is addressed, by then nutrition sensitive part, which is another sectors, as well as the health, so the problem is this one.

I: So pillar problem is, there is no collaboration.

R1: No collaboration. No, integration is very challenging. Integration, collaboration, coordination, is very challenging.

I: Why is this challenging? Why, why is this?

R1: The reason, the reason is that sector specific their status quo, sector specific or working separately is what they know, very well. And the system does not make them integrity to do on nutrition. The system does not oblige them to come together.

I: So, there is no system which bind together.

R1: Yeah.

I: For them. That was one challenge

R1: One challenge.

I: What else? What else?

R1: The other challenge at community level, expectation from the government. Expecting better thing or donation or everything. Rather than doing by themselves, waiting for the government or donors, somebody who do things for them. This is ambitious expectations.

I: From the community.

R1: From the com-, community, yeah, expect. They wait so, rather. We cannot address the nutrition problem by government or donors. But the community, if they do by themselves, if we do the social and behavioral change communication very well, very well, then the community can do things by their own, their own trying. So, that is a second problem on community level.

I: What else? You told me two major problems.

R1: Yes, to major problems.

I: Two major challenges. One is shortage of integration and the second one is about high expectation from the community.

R1: Yes, high expectation.

I: So they expect from the government, from the program, to supply them more things. Then they themselves try to self-reliant, they have high expectations. What else, what is a challenge?

R1: Another challenge, another challenge, a third one, not mentioned so far. The third one is financial support, for the budget shortage, shortage of budget and shortage of logistics, to do in this program. Shortage in logistics like cars, and to do supportive supervision to the woredas. And woredas themselves, to the kebeles, they need at least motorcycle in the local supportive

supervision type. At kebele level, of course, you know, there are the system of government there, at kebele level. There is a health post, educate system school and farmer trainers center there. And there are experts at kebele level, so that mean, close follow up. So, the woredas need motorcycle, so they don't have that and for us motorcycle is not recommendable because it is wider to go to the woredas. So, a vehicle, you know, a car is missing. So that is the problem. So, the budget, the shortage of budget is together with budget in the logistics the problem, so.

I: Thank you very much. Let me bring. Do you have any problems that you want to add, in addition to this two or three focus area that he already raised, like technical problem, any awareness problem, attitude problem, of those peoples, staffs, community, and other things. If you want to add more, please.

R2: Another problem is, lack of multi-sectorial nutrition structure in the sector is a main problem. Now, the vocals assigned in each sectors, they see as extra job. And shows weak commitment because this activities not mentioned in their job description. This is extra jobs assigned for them. And no permanent structure in sectors, this is one of the challenge not mentioned. Another challenge is competing issue or priority issue for political leaders. Competing issue. They prioritize another political issues and do not prioritize this for the nutrition issue. Political leadership. Another problem is a shortage of input for farmers, like improved seeds, fertilizers, they cannot get easily. We see at community level, level. And even if they get seeds, the seeds is very costy. For example, if you see one kg tomato or cabbage, one kg is above fourteen thousand birr. Improved seed.

I: Fourteen thousand?

R2: Fourteen thousand.

I: One kilogram?

R2: One kilogram. This is also another issue or problem for the farmers. And another issue is traditional farming. Traditional farming. They are not productive, community farmers. And also the community level, land management is another problem. Land management. They plant quite a pitish tree around their garden and this causes less product for the farmers.

I: This also affects the fertility of gvr.... Land

R2: Ye-, ye-, yes. Also, the, another issue is drought, drought is another problem. Climate change, for example like Borricha woreda and Bilaate woreda, frequently they affected by climate change. And then another challenge is weak coordination. he mentioned weak coordination, vertical coordination is a poor between the sectors. The multi-sectorial woreda communicate with a zone or regional level. The communication is poor. Horizontal communication between also the sectors is poor, this is all, I can add this.

I: Thank you very much. You raised very important concepts. One point additionally I would like to see to hear from you is what are the attitude of those sector leaders on this program, like in the district. I know that at a mean the woreda the chair of that particular issues and is there really communicated? Even not only the staff of this specific sector, what about the central coordinators as well as the chairperson on that?

R2: I don’t think it is not strong. Most of the time they delegate depute aids, delegate … (?) TOR they delegate to our mention or said that the chaired by woreda administrator.

I: Yes.

R2: But, but most of the time the reality is known. They delegate, depute aids everywhere. If you see in regional centre, the TOR say, they leads the president office leads …(17:28)…

I: This multisectoral should be lead by?

R2: By regional president. The reality is not that. They delegate, the depute aids the same still in the woreda level.

I: Yeah. Any additional informal challenges you would like to add? Like raise regard to the string committee, technical committee, the different sector coordinator. Any challenge more? Maybe you first. About the plan, about reporting, performance, challenge?

R2: Already the discussion is on and off. On and off. … (18:14) … And they added further problems, but proposal solutions but do not follow as. Do not prepare the action plan as still the end. They on and off.

I: Thank you very much. I would like to ask you one question maybe. With regard to, sometimes the people consider as this the 08 or multi-sectorial non-governmental program. And they expect more payment, some additional supplies, like that. What is true on this ground? So the Seqota Declaration is the governmental activities or it's the non-governmental organization activities. Because sometimes we hear from the people, this is not the program of the government. Such, such rumors are there. What's the fact?

R1: Of course, as you said, and I also tried to mention in the part of problems. Raising from community, they know there are so many donors and the programs like one WASH program, one …(19:40)… program and Unicef or other NGO programs. So many NGO programs. And the people, people know that. The governmental programs are also for example the water, water supply, the schemes are constructed and then it will be hand over to the community. So, in the government, even though it is the government commitment, they know that the government sometimes do things for the community. So, they know, they wait for such a donation. That is

the main problem, then, but we were trying so don't expect that the organization itself but the government community and we were, are trying our best but people, you know the behavioral change does not come overnight. But we are trying.

I: You are trying, yes. Thank you very much. Let me ask you any opportunity that you can raise me, not yet consumed in the community, with respect to the culture of the community, with government structure, some very important opportunities to achieve the vision of this program, but not yet consumed or that you are using. The opportunities. You can think about the opportunities with respect to the culture of the community, religion, or the local context, or political structures, something. You can raise us, on opportunity. You know what that means, opportunity, actually.

R1: Yeah, I know that.

I: Let you say something.

R1: Thank you so much. Before, I mentioned that there is the government structure in the lower level, which is kebele level. That is the best opportunity, the existence of the structure there. There is agricultural experts are there, so that is the best opportunity, agricultural and health extension workers, and school principals at the community level. You know the community by themselves should bring the change that is the best way of bringing the change of, the change in the nutrition problem. So, because expecting donation, and governmental support does not bring the, which is the good change so. That is the way of the strategy.

I: So the existing government structure…

R1: The structure.

I: In communities is one opportunity.

R1: One opportunity.

I: Like health extension worker, agricultural extension, school, principals

R1: Yeah, livestock, experts.

I: What else please?

R1: Another, another opportunity is the fo-, the key, the focus of government. Federal government. The food and nutrition strategy before three years in twenty nineteen, in twenty nineteen, it brings the focus of the structure up to the lower level, up to the kebele level. So, but another follow-up, another strengthening even though it requires, another strengthening. To the getting of searcher, food and nutrition strategy, and guideline, and also the innovation, innovation point to start there in the 08, existense of PDU and costed woreda level.

I: yea.. it is program delivery unit.

R1: Yes, program delivery unit. … phone is ringing .. off or give it to me …

I: good thing

R1: so, and also the community lab and innovation, that means community by themselves. Solving their problems, identifying by their own. The problems and then giving solution by themselves in the community lab.

I: Do you think that the community lab is the one thing you establish, yes?.

R1: Established during the expa-, innovation period of, period of the 08 n in Amhara and Tigray region, community lab. And there is also another data revolution, that is capturing data is also another innovation. So this system, now even we are trying to expand in UNICEF, unified information system.

I: Unified Nutrition information system.

R1: Yes, nutrition information system is opportunity. These six innovation points in 08 ,are opportunities, and another governmental policies like Yelemat trufat. ehh which …diversified

I: yelemat trufat ? 'LIMAT TIRUFAT?'

R1: What does it mean?

R1: yelemat, means there, in one dish there are diversified food. Should be provided in one dish or in one meal. This is the policy of government and that supports the 08 or the nutrition program. So this is new policy. So and the other, other opportunity is like the World Bank, getting attention of the World Bank. To invest in nutrition. Before, you know, there is one presentation the depute president of Ethiopia and health minister of health, together they participated one conference to tell and present about information declaration of Seqota and there they said the World bank, the leader of the World Bank there, we invested on infrastructure. So many billions on infrastructure and there was no change, that much It is required. Hereafter, if

we invest on nutrition in human development on nutrition, we will bring the change. The leaders said. We will bring the change and we will invest in six African countries. In six African countries, hereafter. Even you know, in Amhara region, World Bank started support the 08

I: Oh.

R1: So, it is getting attention of World Bank leaders. Because it is the productivity, the economic development, if you avoid stunting, it will come by default. So, they give you attention of these leaders is the best opportunity.

I: Thank you R2, thank you very much. Would you, I've some more points maybe. Opportunity. Because you already raised about the structure, existing structure of this program, existing structure of the government. Is very one of the opportunity, the owners issue. Attention of the government, attention of the World Bank is very important opportunity. So you, attempt to achieve this programs. Could you say something maybe that you think is opportunity maybe with respect to the community and culture, or others things that you like to add maybe.

R2: I only two points can add. One thousand plus public movement strongly working in the health sector is a good opportunity.

I: What is that, one thousand plus?

R2: From conception up to

I: Yes.

R2: Two years. Up to two years.

I: About the pregnant women and the first two birth days of the child.

R2: Child.

I: ’ish?’.

R2: Yes. To stop stunting, when they stunting in twenty twenty-two Ethiopian calendar and twenty thirty in Gregorian calendar. The government working strongly on pregnant-women’s care, feeding and other health care, you know, all the health care. And change feeding, breast feeding, and complementary feeding. This working is a good opportunity. So, another point is the costed woreda based plan. Costed woreda based plan. The plan prepared in the grassroots level, in the woreda technique committee, sitting in one area and prepare the costed woreda based plan. Some food availability issues there, food accessibility and they plan, food utilization also and cooperating in plan and the resource mapping at woreda level, From government, from community contribution, from partners and their search. And map the resource and they implement it and evaluate quarterly as a woreda level. This is a, also good opportunity.

I: Thank you very much. Woreda, the, the, the cost woreda based planning?

R2: Costed woreda based planning.

I: Costed woreda based, what does it mean, please? I want to well understand it. I, maybe I know that something about that, from the concept, what does it mean?

R2: They plan jointly.

I: They plan jointly…

R2: Yes. They, as you know, the nutrition is a multi-sectorial issue.

I: Exactly.

R2: Not a single sector issue.

I: Exactly.

R2: Therefore, the agri-sector, the agricultural sector, livestock sector, health sector, women and youth and social sector, and education sector, WASH sector, and the other disaster prevention sectors, all jointly sit at one and list the activities which contributes to reduce stunting or…

I: Nutritional…

R2: Nutritional problems. They list for example, nutrition days groups like others vegetables, so many activities mentioned under the agricultural sector. The livestock sector also, they animal animal rearing, animal feeding, animal care, like these activities all mentioned. And then, education sector also, student hygiene, also student feeding. They list all the activities according their sectors and jointly, and they search map the resource. What we have they say, what

community can contribute? What the partners can contribute for this issue? For this issue, they said, and search the resource. Map the resource,

I: Yes.

R2: And jointly plan…

I: Plan.

R2: Jointly evaluate,

I: And jointly evaluate last report is a challenge.

R2: Ye-, ye-, yes. If you see, agri- agri-sectors and health sectors jointly visit household, household, and counsel the household members, so that.

I: Thank you very much. I have the last issue maybe. You have the already known strategy to achieve this program, Seqota Declaration program, the maternal nutritional problem. you have strategy nationally to woreda region and to the district level even to the other region. Because you have passed this road to at least one year expanse of movement, what, what best strategy is your recommend to improve this area? If the Seqota or the multisectoral program improves this strategy, or includes this part of the strategy, then that we will achieve more. You have some to say about the strategy?

R1: Yeah. You know, our mentioned problems, or the challenges, if you identify the problems, then you come to the solutions…

I: Exact.

R1: …in research. Identification of the problem and the data, as mentioned the logistics, the cars and the shortage of budget, and like are the problems getting more budget is a solution.

I: That is one strategy.

R1: That is one strategy. More budget, more financial.

I: S how to, how to get maybe. Improving the budget status of this program is one solution, then how to improve?

R1: How to improve.

I: In your opinion. This community participation to include, donor searching for more donors, or including government, or activating more the government to allocate more budget. Which strategy?

R1: You mentioned good things. Trying or pushing the government to allocate more budget and also finding as my colleague he said. The name of the costed woreda based planning is also. It shows multi sources of the budgeting. Costed means, community themselves allocate by themselves, so expectation or motivating or promoting the community to focus on nutrition issue

because stunting is the problem of the community. So SBCC work. More SBCC work requires there.

I: SBCC, is it that…

R1: Social and behavioral change communication.

I: Yes. Social and behavioral change communication. So working on that is also a recommended strategy.

R1: Yeah. More recommend. They allocate at kebele and community level. Then the government, pushing government, to allocate and donors to also to allocate more attention and give more money. And the strategy by itself of course brings solution And the innovations, identified innovations needs more focus more on that and brings solution.

I: Excellent. Thank you very much.

R1: Thank you so much.

I: Any additional point with this, with respect maybe to the best strategy, best recommend way to improve this problem.

R2: Multi-sectorial coordination should be strengthened.

I: How it can be strengthened?

R2: It should be led by the president at regional level and should be led by the woreda administrator at woreda level in the sense of delegating. The depute, the deputes are not decision makers for the budget. Should be strengthened and another is structure, permanent structure. Multi-sectorial permanent structure should be established in each sector instead of delegating focal persons. The Another point is multi-sectorial M and E guide should be guide M and E guide should be established.

I: This is about monitoring and evaluation.

R2: Yes, yes. Monitoring and evaluation from data recording and reporting procedure starting data recording and reporting procedure. You know the nutrition, specific indicators, you know. The indicators have standardized data recording and reporting procedures. Whereas nutrition sensitive indicators has no, have no recording and reporting procedures. And even reporting flows clear, clear reporting flow. It needs clear M and E guideline and evaluating, data verifying procedures should be established. You measure the change by M and E system. weak M and E I think and we are recommending for federal level to prepare these all.

I: Excellent. Thank you very much. If you have the last point you would like to raise, that we didn’t touch here because of the time, you can raise maybe. Any point that you would like to say. No?

R1: We touched all of them.

I: Thank you very much.

O: Thank you. ‘Ejigi argen inamesegenalen!’

**Interview IX: 07**

Key:

I: Interviewer 1

D1: respondent 1

D2: Respondent 2

R2: interviewer 2

Transcriptie interview 9

**D1**: For the past five years uhm in case there are three projects that may **…** sentences). Silence.

**I**::For some disturbances

**R1**: : yeah that's nice

**I::** Yes

Amharic

**D1**: : His name is respondent 1,

**R1**: : jaah hahaha, that would be nice

**I::** Good Morning,... she interviewing, thank you very much. I1:,

**I::** Yes, she is my research partner, she works uhh research with me, the topic of this research is about multi-sectorial nutrition program, partivularly with malternal issues and she wants WASH as contribution. So, actually the purpose is of this interview is to understand the challenges and opportunities about mali-sectoral nutrional program in Ethiopia for this government and governmental actions. What they are? what the challenges, what the opportunities and what the best strategy they recommend for nutriotnal problem of the country for the children and the

women so this is our aim of the topic and we are happy if you start introducing you, the name maybe…(rure?) age, marital status, profession position and we will translate…..You can express…

**D1**: : okay, thank you very much for giving this chance to be part of your research and interview with me and I am WASH business development associate manager and I'm working for PSI and in PSI there are 3 main wash projects. The first one is ..(GTN , *that is a a …nutrition part in the interventional woredas*, transform wash and unicef wash. So, thank you very much.

**D2**: : i'm glad to be part of this research … I'm working for 07, especially for detailed program for almost 6 + last years for wash program and uhm the position is wash executive advisor and working before the transform wash and doing the GTM program and almost cover all SNNP areas by wash program or by wash. thank you very much

I:: excellent thank you very much, thank you for introducing yourself your work experience and maybe your position here and may be can you tell me your profession associated to this … to your work and how start this position, marital status as well?

**D1:** : Okay, I'm married, still waiting for children to come, i already started to order hahaha ..laughing, and my age is 41, last May 16, I celebrated my 41 years. And. My study is previously, i was graduated from Hawassa university, department of plant science, that's agriculture, . But I studied my MSc in agre-business and valid chain management.

**I.:** Ohh, Thank you very much.

**D2**: : My background is, I ammarried, I have two daughters, one of my daughters graduated from business and economics faculty from Hawassa university, marketing. So my background also marketing. May be I am influencing her.kkkk... They laugh together.

**D1**: ,2 & I:: Laughter

**I::** thank you very much, so let me start the first question that is about nutritional problem in the area where you work. now this PSI, Particularly the wash focus area that is now, how nutritional problem looks like?, can you tell me?. May be tell me additional problem of the women and of the children and position to this. In general, what looks like?

**D1**: : Thank you very much, 07 ethiopia especially those three have conducted activities in 33 woredas. In Sidama and SNNP areas. the situation is different in different woredas. For example in Sidama, the nutritional problem, in fact it needs research support to say but according to my observation, it is better in Sidama compared to others. Because the nutritional status of the region is better as compared to other, but in Hadiya especially In some woredas the environmental condition is very harsh and agricultural productivity is very low and the ecomic background is that people are very low. So therefore, we observe some children withsome nutritional problem. Otherwise in other areas it is better. For instance, in South-West region, still the agricultural environmental condition is very good and the nutritional status is better. But, even if the overall condition of nutritional status is very good in Sidama but we observed that some people in different area and, there is some households have nutritional problems yeah that is why we are intervening wash activities here. Because the contradiction is the people with nutritional problem are subjected to different diseases, especially diseases related to sanitation and hygiene. the difficulty also, people do not have the capacity to purchase wash products to improve their households that is why we intervene there.

**D2: /I**:?: They are unable to buy that products? Excellent.

**D1:** : yes, that product and

**I**: You intervene in this areas?

**D1**,: Yeah.

**I:** Excellent.

**D2**: : May be to add on this,

**I::** May be when you add on this point, tell me, how it looks like the trends. previously, how it was. How it is going on, is it getting worry or improving associated to WASH

**D2**: : okay, even theGTM product projects we started to do, that detailed activity,

**I:** what is the GTM?

**D2**: Growth Monitoring activity nutritional activity uh it was concertium project but uhm head of the projects was save the children

**I::** Safe the children

**D2**: : Almost all the nutritional activity covered 06. They are supporting on wash component. Even the projects goal is to reduce stunting by 20%. When we come to our activity on WASH businesses, previously, it was totally difficult to influence or to buy the improved latrine. Because nobody can prioritize for the latrine status, toilet status. After our engagement, step by step increased. Still, we didn't achieve the target on activities woredas , as compared as with transform WASH woredas, because the topography of the area , the distance from here to that area, is too difficult but it is still progressing

**I::** so, there is good process. So from the worst area coming to get better. Thank you very much

**D1:** : Btw, the detail project growth through-nutrition a project is initiation of Obama.cross-nutrition is a project, It is initiated by obama during his presidency and it is implemented by four main organisations: Save the children, who is the lead. by the majority of this data related to nutrition is founding save because they have the agricultural part.

**I::** so save the leading the project?

**D1:** : yes they lead the organisation. Next one is world vision, world vision is engaged in constructing the water points, water ways and the like and water activities. The other is care, Care also working for people that is related to agriculture,with high nutrient food. Seedlings and seedss nutritious food and make households to grow that serialss or crops to improve their nutritional status. 08 handle the WASH part, so this was the combination of four organisation.

**I::** thank you very much, have you heard about multisectoral nutrition program and what is your envolvement there. did the government have such an initiation on multisectoral nutrition issue and have you heard about this issue and if yes how is your employment in that area.

**D1:** : as far as wash is concerned, if there is any nutritional meeting or multisectoral platform, we are invited there, to be part of that platform condition. i remember i participated more that 4 or 5 meetings that was conducted to create multisectoral nutritional conditioning area. as far as

nutrition is concerned, this is one part that affect , people with poor nutritonal status. Therefore, people in south especially before sidama is separated in a region together with SNNPR, we participated on many multisectoral platform that creates good environment, I mean nutritional condition for households. For example, water office, agricultural office, water, women and children are the part of this multisectoral platform. But, the strongest platform is found in wash, wash creates especially thanks to transform wash project, we created strong multisectoral platform for wash but we didnt included so far for agriculture as nutrition but the government of ethiopia and the government of SNNPR have a policy to create multisectoral platform in relation to wash and nutrition.

**I::** so you already heard about mult-sectoral and already involved in these activities. Are there any particular activities in this project that is activities related to multisectoral nutrition and wash if you get my point.

**D1:** : yes i get your point, but

**I::** the activities in relation to multisectoral aspect of nutrition

**D1:** : yes, btw this as i mention before we had three projects directly related to wash but one year ago

**I::** What were the 3 projects

**D1**: : transform WASH, GTN, unicef wash. But two of them are already phased out. The activities that directly relate to this, nutrition is related to that two projects.

**I::** GTN?

**D1:** : GTN and unicef WASH

**I::** They are phased out?

**D1:** : yes faced out, december 2022 so after that time we don’t have any activities related to nutrition but now no nutritional activities

*three people talking at the same time (16:26)*

**I::** but you do WASH activities to improve nutritional problems in specific areas

**D1:** : yes

**I::** that's nice, thank you very much

**R1**: : Is there a reason why the programs stopped, didn't it work or what is exactly the reason why two of them stopped

**D1**: : because of funding problem

**R1**: : it is not because it didnt work?

**D1**: : no only problem of funding, .. was interested to provide us money for transform wash but not GTM. btw the gtn activities cross nutrition is a multisectoral approahc that provide solution for households with low nutrition.

**I::** excellent, so can you tell me the specific role and responsibilities during this GTN particular this office What are the specific activities in relation to role in activities specified by type of GTN for this purpose

**D1**: : our activities are responsible for conducting all activites that is related to wash. We create demand for wash products and we provide supplements of wash products for our community. this is our responsibilities. Do you want other NGO's activities?

**I::** no you mentioned two important points. One, creating awareness and demand will be include second one, supplying their demands based on the demands you supply

**D1**: : We train business partners who are involved in creating demand and supportinting providing supply for this WASH products. Job opportunity for the community especially for the low income households. So, in this way the three things are integrated and provide WASH products for the community. we are not the one who provide the support for products for the household directly but we train enterprises, we train local people to create demand and to provide the supply for that

**I::** so indirectly you ar also helping the people to make income and job opportunities

**D1**: : yes

**D2:** : to add on this, the basic concept to engage in WASH, water sanitation and hygiene was nutrition only nutrition not if people get balance diet without wash, there is not Hing. if they go to toilet, without wash or sanitation, totally no effect. So because of that it is related wash business, wash acitivites with nutrition. As he mentioned earlier, we establish sustainable activities for all business partners in the community. Even if the project already phased out, the business continue.

**I**:: thank you very much, that is very wonderful. May be can you tell me how you coordinate these activities? Because to coordinate these activities there is some structure best. Some system you have. May be coordinate the step here. How you actualise these activities. How you coordinate, how you run this activities

**D1**: : for the sanitation activities we do have business model that we deliver our products to the community. That has its own structure and business model that works for each products. At 07 office, we do have associate manager who totally manages the project and we have associate, no business adviser and at local level we have facilitators. so our three are integrated to provide our services and products. And from the community we do have field agents the one who knock door to door to create demand and become bridge between our business partner and the household. In this way we create, we deliver our service and product. In relation to the government, we work with, at the top, health office. Woreda, Zone and regional health offices are the one that is directly related to WASH activities. And water office is also our partner. Also our partner is women and children because nutrition is highly related to children and women's and we conducted our activities integrated with this sector WASH i mean women and children affairs. The other, can i forget, TVET we worked with TVET or job creation. Technical part is totally supported by TVET. if there is no TVET in the woreda, we work with job creation. The mandet and responsibilities are at the national are almost similar with TVET. in the absence of TVET, the job creation are the technical part of this wash program and services.

**I::** That's very nice, let me ask you one important question maybe. i wonder if you tell me the challenge of these activities because that is our question. We want to understand the challenge

working with the sector working with the government working with community and customers. There are challenges to achieve wash activities, why don't you tell us that challenges please.

**D2:** : I'm proud of this GTN and transform WASH program because we influence not only community but also ethiopian government. Our structure is market based sanitation before this two projects, there was different activities done by different NGO's with free distributed sanitation products just like that. But our projects comes, every person can invest for his toilet. so, the ethiopian government also engage for this program. We are established market based sanitation system in ethiopia. so the challenge was nobody can prioritize to invest on his improved latrine. Day after day we influenced them. Finally, even.. the challenge. The challenge is that even if he has a nice mobile and invested a lot but not for his toilet, that did not change. But currently changed.

**I::** So the problem is the attitude of people

**D2:** : definitely

**I::** They don't give attention to the latrine and instead of giving materials ..

**D1:** : btw you ask us very huge question, you can answer in multiple ways, it is very big question. Let me put it this way. The challenges are to be mentioned in three main areas: demand, supply & environment otherwise the challenge from the environment is the challenge related to the community. In the community, btw toilets are not a big priority, the big priority is for example, in every house there is a mobile but there is no toilet. One mobile may cost more than 2000 or 3000. One mobile but everyone in the household has a mobile phone. In every household there maybe is one motorbycicle but there is not toilet. So in the community, toilet is not purchased priority. Sanitation is not concerned as other issues like agriculture and other. You see the big challenge.

..... some sounds from ouside

**I::** So this is the community challenge

**D1:** : This is the challenge from the community, the other challenge from the community is that even if this is true, there is the seasonality of the income in the community. Their income is

concentrated in some months of the year. in the other parts they don't have income. so, this affect our activity, the seasonality of the income. The leading style of our community is For example if they have enough money doing harvest or if they sold products they use that money for other activities and don't have even food to eat in the other season. So this is the big challenge for the community,

**I:** the problem of saving?

**D1:** Yeah, the saving and lifestyle is very affecting the community. Even in the community there is gap in awareness. some people not purchase the product because the awareness is very low. It is not works for all community but some observed did not build their toilet because the awareness is very low, they consider it as unimportant and is not concern for that. They don't know why our children is sick, why diarrhea is occured and also why their children are died. They didn'tconsider because they do have improved toilet

**I::** So there is the problem of awareness.

**D1:** The awareness in total. So awareness level in the community is zero. Even if there are many NGO active, governmental state workers there, the awareness is very low. The other challenge from the community price of latrine is problem especially when we start the project 6 years ago, one toilet costs may be 500. now it is 2000. Simple toilet! .. The price affects, one factor that affect the community not to purchase the product is cost of the product

**I::** cost of sanitation product?

**D1:** : yes, the cost of raw material become very expensive. So the other challenge that affects the community is that previously the demand creation by the government used product based approach.

**I**: What does this mean?

**D1:** they tell them about the benefits of the product but we tell them the problems of not having the product. Previously, this product is very high (points at water bottle) it has non-sense to the community but the problem of not having improved latrine was the best way to create demand in the community. Now that the challenge was that. I cover the demand. the supply. The very big

challenge previously was the enabling environment side challenge that the government didn't support sanitational activities so far or previously. For example, there is no, there is huge loan for agriculture but there is no loan for wash. There is main support for agriculture in water but no support for hygiene and sanitation. There enabling environment for wash was very poor. But now, napoleon mentioned, we have high influence to the government. the government put as strategy, market and sanitation because of the intervention of transform wash. we contributed 100% for the development of that in malnutrition.

**I::** wow that is very nice, thank you very much maybe to illustrate more can you say something about the open defecation issue, its status because one of your aim or plan is to avoid the **problem in the future, what this look like**

**D1:** : when i was a student in 1994 at the freshman university studetn during that time the OD **condition in SNNPR and Sidama, the same region was ---%. Do you remember?**

**I:** 94%?

**D`:** % of open defecation. No open defecation. That was report. No! They achieved there. Open defication free kebeles. 94% of kebeles in south because sidama became region recently. were free of that. But it returned. Because it didn't supported by improved latrine. during that time the government followsCL teaches community level hygiene approach and all the community provided with local materials and it failed down and this coverage came back to 7%.

**I::** my God

**D1**: : because it is not supported by modern toilet but now still the government of ethiopia target especially Ministry of health is making kebeles models, creating model kebeles throughout in ethiopia. In Amharic 'Tsidu mender' , Tsidu Ethiopia (clean village, Clean Ethiopia) Target for the government, for the minister of health up to 2030 is to creating tsidu ethiopia.

**I:** 2030?

**D1:** Yea, to create, therefore, it means directly creating open defecation free kebeles. That means if everybody use toilet, ethiopia becomes 'tsidu' (clean). so now the government link this activity with market based sanitation and starts to get attention from the government. The government

one month ago the minister of health, vice minister was in one of the districts in sidama region) to visit our work. He really appreciate our work and he promised to link open defecation or creating or open field defication or tsidu and the goal for 2030 with our market based activities he promised, and now it is the status to creating open defecation free kebeles is main target of ethiopia by creating model kebeles and so on in ethiopias . But it is reported that currently OD kebeles are very low. But literally, it is clear that there is OD kebeles.

I:: Yeah, Thank you! what it looks like the commitment, the commitment of the government and partners, you told me about the commitment of the community is problemized area. but what about the commitment of the government and those who work on wash area. what commitment looks like

D1: : Yeaha, I respond this question in two ways, there are two areas. One area is where transform wash is implemented in other areas there are woredas without transform wash implementation areas. Because we have experience with government in all woredas. 'We support government to train or to support market based sanitaiton related activities. Therefore in south we have six woredas, in sidama we have four woredas. In south we had nine woredas. in these nine woredas , the commitment to the government is very high because there is multi sectoral platform for market based sanitation and they totally committed to work on wash actiivties. they have experts who train on this market based sanitation. there are government technical expert who train on this technical aspect of market based sanitation. all aspects, the technical, deman creation, the environment and so on. Therefore, they critically monitor and evaluate their activities. They plan to cover 100 % now they are working to create blanket coverages for all of woredas. In transform wash woredas, in this total 15 woredas. For in sidima and 9 in SNNP, there is very nice enabling environment there. the indiciation for the enabling environment is mentioned by for example they provide land for interprices to manifacture their product. they provide land and they have sheep for interprices to conduct in sanitation providing activities all the kebeles and woreda officials conducted in creative demand in the community so this is the enabling environment that creates conducive environment for the wash community. But in other woredas I doubt! in non-transform woredas and zones, so it is very difficult to say there good enabling environment. In fact this is because they don't have enough knowledge to support the

community. We identified this gap and we support the regional government to provide this market based sanitation trainings and skills for other zones.

I:: thank you very much, the challenge is not actually good enabling environment but progressing commitment. and even they have land they monitor they evaluate so that is very nice commitment. They have plan. So you told me there are two types of WASH transforming woredas and wash transforming woredas…

D1: : to add on that important for you to have this information. In transform wash woredas, woredas have strategy plans for wash, because of the support provided by the WASH. they have strategy plan, they sign MOU) memorundium of understanding to implement these sanitation activities

I:: with whom?

D1: : the five main sectors, water, health, women and children affairs, TVET, and microfinance. they sign *MOU* to conduct market-based sanitation in total wash, sanitation and hygiene. The other issue is that may be included is woredas from the strategic plan they prepare their own plan from wuruda to kebele levels to do sanitational activities from the whole woredas all activities include sanitation and hygiene in numbers in kebele levels. So, this is the transform wash activities to create enabling environment.

I:: excellent thank you very much. you told me government is now committed and promised you to support and link specific sectors to achieve activities especially for the plan tsidu in Ethiopia 2030. excellent thank you very much. May be particularly to hear about the budget issue related to this program. Could you say something about the program relation to budget about its adequacy shortage in activities because one of the challenges and what is the challenge in this perspective.

D1: : on your previous question you were asking me the challenge and that is why it is a very big question. if we classify the challenge of aspect of budget. So you are asking me the challenge of wash sector in ethiopia that means. You start from the government and national, so it is very challenging. The government of Ethiopia allocate the budget for all activities, they put in blanket. For example they allocate one million or two million but when it is reaches woreda, or

kebeles, there is no budget for WASH. no budget. This is a very big challenge . Even the government when they

I:: sorry, you mean that officially and woreda level, there is no budget to wash

D1: : even if there is budget, they give little amount of money for wash, if there is a budget. At the beginning it is difficult to get budget for wash. but we are working for this activity for the past five years. the government funded money for transform wash, is conceptium project and is implemented by four organisations. 05, 07 directly work in creating enabling environment, plan international work for demand creation through health extenstion workers, and IRC international research center for wash conduct a research. So 05 directly support the government to create enabling environment and with conceptium, we support one wash national program. The budget that is accessed for wash is from onewash. Do you know one wash?

I: yeaha, I heard about One wash.

D1: yeaha, we support onewash to allocate enough budget for sanitation and hygiene activities. So we were successfull in allocating budget for transform wash woredas in transform wash woreda, we clearly see the government starts to allocate budget for wash. For example two maintain water points, to construct water points, , to allocate budget for promotion is already on board. but for the non-T WASH i'm not sure. Because I was not there.

I:: so you mean that from the one wash they already have the budget and is no problem with budget. But from the government side they allocate the budget particularly in the district there is no budget, even if it is allocated, it is very low. that is another challenge

R1: : may i ask something, onewash is mostly about children and women, the babies right

D1: : which one?

R1: : one wash, that is mostly focussed on

D1: : no, women and children affairs

R1: : yes, is that the reason why they give money to that, they think that is more important or why do you think they give more money to one wash and not to wash

D1: : in fact one wash is collecting budget not from the government only, they search from another option, donors, others to collect budget. That is why it is called onewash. All the budget, all the activities and all things are in one place.

I:: can you say why the budget allocated to the area, woreda and district is very inadequate, very unsure. Can you tell me some reason maybe

D1: : it is very challenging, to know it, btw in brazil some 3 or 4 years ago there was national, no, international wash summit conducted in brazil and there the government of ethiopia accepted that they didn't focus on wash on budget issue. They know, sometimes

I:: I think the problem of understanding

D1: : yes understanding attitudes, they didn't know the impact of wash

I:: and nutrition and different..

D1: : Last time we did, some 15 days ago we had meeting with the ministry of health in relation to allocating budget in loan for businesses and taxi excemption on important product. They didn't know how much Ethiopia it costed for not having improved latrine for example the coverage now, the recent research, 9% only improved latrine in Ethiopia. Improved latrine in Ethiopia 9%. In fact previously it was 17% but the standard was revised, the standard was saying the improved is changed. So it is totally removed the percentage and it reached 9. so if we put tax excemption on products, it improves the percentage of the 9% into 17%. May be if I'm mistaking in the number but, inducing tax on latrine products, only improve increases the number so last time i will understand, they did not understand

I:: so the one you say is that the tax reduction for the product of sanitation it will improve people's demand and and it ultimately improves Coverage. So, is there problem with resources like human resources who work on this area and government sector, this sector?

D1: : Yes, but the very big challenge in the government sector is Turnover. as compared to experts in majorityof at least there is one expert but the turn over is very high. you train someone for one or two years about wash and they leave to other place

I:: They leave the profession or the work

D1: : They leave the direction area to become political leader

I:: is there any problem why to work that area why they leave?

D1: : as compared to any sector, WASH is very good in pay salary for the government, good salary price is in wash. That is why, when they are in *kebele,* they want to go to *Wareda* because the salary is very high. if they are in *Wareda* they want to work in *zone,* because the payment is still high. They move from one area to another

I:: because of the payment

D1: : because of the payment but it doesn't mean there are no shortage of work experts but the very big challenge is Turnover.

I:: maybe you say that sometimes you train these people so these people can work in that area but they leave, but how, for example do you have any number of how many trainings they took, the workshop from that area, capacity these people that work in that area. Is that common in that area. Otherwise maybe there is a link to that place. So how they capacitate the people who work in that area

D1: : it is also an advantage for them to leave as a opportunity. Last time we conducted market sanitation status assesment and from the data we understand the majority trained, experts already they, but we plan to provide refresh training for the new one

I:: So you mean that there is opportunity for training, workshop and any consultative workshop training is common for staff workers in that area.

D1: : in wash area, is in majority common. It is an opportunity for

I:: so there is no problem of training, no problem of workshop for staff, because maybe there is .. one of the human resources you recruit the people, you maintain the people in that area so one of the means to maintain the people working in that area is providing training opportunities workshops so you think there is no problem in that aspect

D1: : It works but these market based sanitation and special focus on wash has started some five or six years ago it is traditional way increasing coverage for wash and for free previously. So this

one is new but approach gives very good opportunity for staff, otherwise it is true that to maintain the people you have to train but specific to wash it creates opportunities for staff to leave, to the opposite.

I:: What about, about the consultant workshop for the partner government. Was there consultant workshop means partner with government sector in this program

D1: : yes, two things are to be mentioned here in rurla areas when we are working with government and local partners we have linkage creation and review meeting there all partners sit together and discuss about the status identify challenges and put action points to for the way forward. this is one way for to discuss on it. Apart from this the 05) one of our concertium partner, 05 always have biannual consultative meeting with the government private sector partners and selected government officials. and we conduct consultative meeting. We prayed for the government on the implemitation of wash, if there is new ideas, new agenda's we concert them. so it it is regular, it is regularly for the past five or six years we conduct this consultative review meetings.

I:: well, i heard the term 05

D1: : yeaha, it is the abroad government

I:: I read it

D1: : ;05 is master NGO on value chain especially agricultural value chain. Because Netherland is known for agriculture

R1: : yes we are

Interviewee: even if they are small country, they produce more than Ethiopia. agricultural good country and they are known in master value chain. Especially dairy and animal and even crop value chain. They work with us on WASH. You see one of our .. concertium member

I:: So it is the 05 who organise consultant workshop for you.

D1: : yes and environmental governent part is covered by 05

I:: 05, oh yeah

D1: : They work on the government enabling environment for example. That is why we influence the government of Ethiopia to change implementation and let me show you one activitiy that is common in 05 to create enabling environment for the value chain. BTW the combination of these four organization is depends on their specific capacity. 05 is known for creating enabling environment, plan international is known for creating demand, PSI known for social marketing. that is why they create. Oh i miss the, Anyway continue your question

D2: : to say about 07, about population

D1: : 07,

I:: 07. 07 is known for?

D1 & D2: social marketing

R1: : So that is the combining of the three things,

D1: : yes, IRC is also known for research, this one is core elements of enabling environment. Therefore 05 works on these issues with regard to WASH sector. So for the past five years we have been working on policy and strategy. That is why the government of Ethiopia changed its policy.

I:: ahh

D1: : so we have to focus on market based sanitation. We convinced them . because for example the World vision conducted this sanitation and hygiene for the past 27/30 years that only provide not more than 1000 products for this 27 years. but we in five year, we sold out more than 29000 products, that means we covered 29000 households.

I:: wooo

D1: : yes because of the policy, there is no free provision of sanitation in Ethiopia

I:: And demand should be

D1: : yes, the other issue is institutional arrangement. That is multi sectoral platform is designed because of these activities, who would be responsible for these activities. The business model has

two types, one the private sector, the other one the government and the government there is the business model that is designed to implement sanitational activities. the responsibilities of omo-micro businesses the responsibilities of health is mentioned clearly the role and responsibilities and the other issue is financing. the very big challenge in wash was financing. There is no any organization in Ethiopia microfinance nutrition who provides loans for businesses. Now all microbusinesses start to provide loan. So this is a financing issue, and the other one is ensuring the availability of products. cost effective implementation, monitoring and evaluation. 05 trained the government partner to conduct the study, they trained on this issue. that is why they become efficient in providing training for environment and standardization and research. standardization, that is why she is interested

I:: yeah, we will go to them, maybe you will give us contact number of 05 people

D1: : no problem

I:: because it is very agricultural aspect that we ask, one of our partner, julie who works on the agricultural part, we will move with her. Thank you very much. So I'm going to right after my questions and then you already have seen to create awareness in the community. What are the mechanisms you follow to create awareness in the community and demand maybe, i want to hear

D1: : this is our commercial strategy, but it mix simple for me to create awareness. And in demand creating we are different approach to system. The first one is SBCC (social , behaviour change and communication. the government has its own strategy and SBCC guideline. and by using that we use health extension workers to create demand for WASH and sanitation and hygiene products. the other issue door to door promotion by manufacturer, the one who produces the product, and sales agents, the commercial bit

I:: and make door-to-door

D1: : they move door-to-door and create awareness for the community. This one is the very best for majority of products sold out or .. by the communities because of this. door to door, we knock door to door and we sold the products and we completed households to create. The other one is group promotion, on social gathering, in social gathering especially government meeting, very

I:: very easy to aware

D1: : we adress many people at one time and women development targets by using women development targets. and in new aspect, by using WASHco. Water sanitation hygiene committes.

I:: commissions?

D1: : commities. for example in ethiopia if one water point is created or the government digg the hole at the same time theyestablish association or committee. the group who manage water point. So we use that committee to create awareness. The other issue is master nutritional activaiton on market based. on market based in promilance we .. the product in the abilities of sanitation. so we are this many people. like the promotion of bill, we use every gathing aspect. TV, radio, in person

I:: ja

D1: : door to door,

I:: these same mechanisms

D1: : the other issue is using social structure specially religion. preachers, community leaders we use this,

I:: okay thank you

D1: : in this way we

I:: you create awareness in the community, that is very good thank you. Do you have any more question, if you want you can ask me (looks at R1: ). Than, yes.

D1: : btw, anytime we are ready to give responds. even when you go back to home

I:: yes thank you very much, thank you. already you said about the strategy and operational plan of your office. to address this aspect you have this and how the communities are committed to support this plan of this program. you say there are many programs with regard to the communities. sometimes i wanted to ask you opportunity, i wanted to raise the culture of this, this association, this type of culture we can say is opportunity to improve this program. other challenge side

D1: : quality side

I:: not only quality side, there is point of the culture that promotes this program. there something like that

D1: : we are conducting research on the Ethiopian sanitation and hygiene status. It is called deep dive sanitation market assessment in Ethiopia. so, it deep dives

I:: deep?

D1: : deep, deep dive. So it deep dives in btw it was conducted in on line.

I:: deep dive?

D1: : deep dive, ive, sanitation and market assessment. it was it was conducted all use of ethiopia. anyway we have many opportunities committees that support their sanitation status. For example in tigray, in tigray, you have to wash your hands before and after urine. because this mich, you know mich?

I:: yes mich

D1: : the community said, if you are not washing your hand, you are affected by ‘mich’. So this is a very good opportunity to

I:: that is their culture

D1: : their culture, in tigray

R1: : Could you maybe explain for me what MiCH is?

I:: mich is type of disease

R1: : ahh

I:: which makes, swelling or abrasion in skin like some allergy

R1: : They are affraid to get it.

I:: if you didn’t wash your hand, you get mich haha

*everyone laughs*

I:: that is a very good opportunity, in that culture in that area, in that agriculture, similar something not exact mich but there opportunity that you can use

Interviewee: there are some proverbs

I:: proverbs, can you tell me that please

D1: : do you know sidema

I:: yes, i do

D1: : in sidama there is a saying, if someone open defecate, the one who see that person will shy. there is one program in sidema

I:: tell me about that please

D1: : that tells

I:: in sidama?

D2: : Iii

D1: : I wrote it in my notebook

I:: i can translate, you can tell me, i can translate

D1: : can i make it in amharic?

I:: yes make it in amharic

D1: : yes, ………………….. (amharic)

D2: : …………….. (amharic

I:: ohwww

D1: : … (laughter)

I:: I don’t know in sidemic actually

D1: : btw, it is the same as in hadia, in hadia also this program

I:: yes

D1: : if someone open defecate, the one who saw it will shy.

I:: and is it instead of the one who work, the activity, but the one who observe will be shy.

D1: : so it will make him shy not to open defecate in the proverb

I:: thank you very much, it is a very wonderful saying . thank you. I think, do you have some promising work structure that you think is opportunity for the government, partner, even within yourself. We have some promising structure, even if it is some program, some product, some promising structure.

D1: : yeah

I:: that helps to sustain and improve this project

D1: : yeah,

I:: because the system, you help rather than activities different

D1: : this multisectoral platform in WASH creates promising structure in the government of Ethiopia, for example in sidema we worked in three woreda's . first in one we worked in for woreda's, now the government, the religious people of sidema, scaled up this activity to 30 woreda's. so this is promising, with our intervention they scaled it up. And additional, 13 woreda's this is one promosing activity. In we were conducted that activities in 15 kabele, one five, the woreda scaled it up in 27, in kabeles, this is promising. It is very nice impact on the government structure, for example we train local kabele leaders, .. managers, on market based sanitation and they .. in main kabele, with their own budget. With their own technical capabilities. So with this it is totally created and conducted activities

I:: yes, excellent. And uh, the last area to ask you is the recommended strategy, recommended strategy to improve this implementation of wash aspect. to improve nutritional problem and to health problem of the people. already the government as well as the partners strategy already.

Based on your contextual experience of your work area, do you think there is a recommended strategy that will hold maybe

D1: : yeah i recommended the government, i recommend every NGO, every private sector on market based approach

I:: market based approach

D1: : market based approach, is sustainable by itself

I:: how it is structured, this market based approach. say something more, because you recommend this strategy to improve, to improve what, sanitation program. what did you think

D1: : market based approach is sustainable by itself, for example, there is no need to go in any government in any NGO to purchase in this project. market provide this, product for ommunity. So it is possible to create, to bring product to the community. here everything the market function is responsible for the governement. so in this way, it becomes sustainable. in Hawassa there is water points or water structure, everybody gets water. so tap. So there is a system created by the government

I:: to access the product?

D1: : yes, the government provide water for you, through this tap. when it reach here, the responsibility of the household is from this point to the houses. but from this point to the source. from government. this is government-market. if the part is broken, the household goes to the market and buys product to maintain

D2: : yes fix

D1: : this is great, the market is functional and government support the market to provide products or systems for the household. now in transform wash woreda, we create market form sanitation products

I:: market based approach

D1: : market based, local supply product, more than 18 types of sanitation types to the community. so but it is only implemented in few woreda’s . the government has to scale up in all woreda’s this approach. that is my recommendation. it became market asked

I:: for example, let us take one about sanitation instrument/product, take some toilet sink

D1: : we have sauto products, sauto part,

I:: sauto?

D1: : sauto, sectoral that means

D2: : I think that health is encouraging the private sector to own this business as Abrham said before, is the water supply. the government bring the

D1: : to the compound

D2: : and after that

I:: the household will manage

D2: : the same thing if the encouraging the private business partners, it becomes central to them.

D1: : if you want market to function, you have to support private sector. BTW, very big agenda, big idea, in market based sanitation is to improve the role of private sector to work on WASHl the government of ethiopia tries for 100 years to improve the sanitation but still nothing happened. based on market sanitation that is by improving, by encouraging the private sector in the market than the kebele come up. btw within this 5 or 6 years it become 9%. otherwise it is not less than 5% previously

D2: : I have such kind of products,

D1: : yes here (points at phone D2: )

D2: : it is automatic, if uhh

I:: what is it

D2: : sato toilet

D1: : sato toilet, safe toilet

D2: : safe toilet

I:: safe toilet

D2: : this is automatic

I:: yes

D2: : if the uhh

D1: : do you know the toilet in airplane

D2: : airplane yes

I:: airplane?

D1: : airplane yes

I:: yes I know

D1: : do you remember, did you visit

R1: : yeah it does like (make sound), ahhh

D2: : when you related, it immediately

D1: : it has ..

R1: : but isn’t it like full at one point, because it doesn’t wash away right, isn’t it full at some point

D1: : we managed to work on local context, with minimum water it breaked out but one that is in airplane is functioning high press.

R1: : yes

D1: : here is not

D2: : yeah it is balancing, so we do have, product also for elders

D1: : women

D2: : pregnant women

R1: : ahhh yes

D2: : like seat toilet

R1: : ah yes, they can really sit

I:: how it is cost, is it very expensive in the context of

D1: : no , previously it is 500 birr, now we come 1000

I:: so is it possible

R1: : i wanted to ask, if that is possible, what is the reason that the prices increased two times. because you said 500 to 1000 and one product was even 500 to 2000

Interviewee: just inflation

R1: : only inflation

D1: : yes inflation

D2: : things from outside, we have this aim plastic one

R1: : ah

D1: : to avoid this, the cover is function by the leg, there is no contact with hand

Itnerviewee 2: this one, yes yes, when you open it

I:: you open by leg, no need of touch

D2: : we do have different products also

D1: : because you are public health commisionar, you know the problem of contacting feces

I:: yes yes, but the one point you recommended this market based approach and why, that is the point

D1: : yeah

I:: there must be reason

D1: : there are many reason

I:: tell one important

D1: : i have one, any project should be market based, because market based avoids diseases .. if market works, no one pays free stuff, free provision. yeah? if the next reason, if somebody buys product with money, it has ownership, this product is mine he said. and he gives attention and use. if you provide product for free the household may not use it at all.

I:: this the sense of ownership

D1: : ownership yes,

D2: : we build that

Iterviewee 1: it happen when they invest their own money

D2: : for instance if you look at malaria net, it is freely, they cover the way toilet or something

I:: as a first, they used actually exact, that is good

D2: : one point. ownership

D1: : the other, i prefer or recommend in market based sanitation. the profit, the profit earned from the sector, takes the sector to the .. the profit sector helps private sector on it. if there is profit,

I:: promotion of product

D1: : they promote, they involved and they stay in the sector, market based, market means work for profit, if there is profit, it is sustainable

I:: sustainable yes

D2: : even if it is margin area we are here around 9 %, of the total population of ethiopia, if everybody improves their latrine

I:: sanitation increased,

D2: : yeah

I:: now i’m almost done with my questions, you can express any concern you have this progrma you want to say. to us as well as to the program

D1: : you told us the research is conducted, when is it finished?

I:: hopefully after a month, we will try to sum up and we will call you maybe to, for somee get together on some area on finding

D1: : so you will bring project

I:: maybe, we hope

all laugher

D1: ”: focus on market based approach, especially even for stunted people, low nutritional area’s in fact the gtm approach works on that on the high nutritious groups for the community it was for free but it is possible to make market based. they give them high product they sold it, they became rich and there is no more stunting for children because economy

I:: it improves nutrition

D1: : yes nutrition, so if you bring project, please make it market based

D2: : it may help you or not, i engaged in global alliance for nutrition, did you hear that project or that program?

I:: sorry it is what?

D2: : global alliance for improved nutrition

I:: global alliance

D2: : yeah, we worked as 07 on market side

I:: okay

D2: : Its main objective was, including breastfeeding after 6 months, they do additional supplementary food. that is even it was, area contextual, the combination 70 to 20 10

I:: yeaha,

D2: : 70 serial to 20 legumen 0 to 10 sugar . So, uhhh there are sustain or not, i dont know actually, we worked in Shebedino (leku), Aleta chuko, shone, geta in gurage. we support them.

D 1: it is better to give the phone number of local, safe the children

I: we communicated to areas and now they appointed us in the afternoon

D1: for the nutrition part, you have that fully information

I: thank you very much
