# Supplementary material for: Exploring the challenges and opportunities of multisectoral nutrition programme in Ethiopia: A qualitative study on combating undernutrition during pregnancy
Source: PLoS One. 2025 Jul 3;20(7):e0311336. doi: 10.1371/journal.pone.0311336 (PMC12225801; doi:10.1371/journal.pone.0311336)
Supplement: S2 Data — (PDF) [file pone.0311336.s003.pdf]

FGD 1

**Section 1: socio-demographics characteristics**							
**Discussant**	**Woreda**	**Sex**	**Age**	**Marital status**	**Profession**	**Position**	**Work experience**
D1	03	P02	25	Married	BSc in plant science	N02	8 years
D2	03	P01	32	Married	Public health	M01	10 years
D3	01	P02	38	Married	mph	N02	11 years
D4	04	P02	30	Single	Social work	M01	4 years
D5	04	P02	35	Married	nutritionist	N02	11 years
D6	02	P02	32	Single	Hydraulic engineer	M01	12 years
D7	01	P02	34	Married	mph	M01	14 years

I: Tell me about the nutritional problem in your region/district/locality?

D3: in our woreda there is huge food insecurity due to the current climate change. The woreda itself is a drought prone woreda and the agricultural production dropped due to the current drought.

D2: the community is dependent/depends on rain for agricultural production because of water unavailability. In addition, there is ongoing conflict at five kebeles neighboring Oromia region which resulted displacement and the farm in these kebeles completely stopped Which exacerbate the food insecurity in the woreda.

D7: Boricha woreda is a known drought prone woreda and there is an existing household level food shortage. Especially pregnant and lactating mothers and under five children are suffering, there is high number of vulnerable groups following at OTP, SC and MAM treatment center.

D1: in addition to the climatic change there is an ongoing conflict which resulted an internal displacement of a high number of families that they unable to do their farm. Moreover, there is

a huge gap in agricultural supply from the government side like fertilizer, hybrid maize seed, and haricot bean which exacerbates the problem.

D6: due to the drought that resulted from the climate change the harvest from the farm decreased resulting in food insecurity. The number of populations is increasing but the harvest from the farm is decreasing particularly with in the past two years.

D5: the agricultural production hugely dropped which resulted household level food insecurity. Shortage of rain due to the climatic change the production dropped since the farmers are rain dependent. In addition, they cannot practice irrigation due to unavailability of water source. The unseasonal torrential rain also resulted a flooding that damages the cultivation.

(I). Have you heard about the multi-sectorial nutrition program? If yes, would you say something about its policy, principles, and implementation?

D4: yes, we screen under two year's children, pregnant and lactating women for malnutrition. We also supplement vitamin A for children, provide Plumpy'Nut for SAM and "fafa" for MAM. For pregnant mother we give Iron Folate supplementation. In addition we give new improved breed cattle for the household to increase milk production and consumption for vulnerable. We work to strengthen the linkage between the households and the local market for improved market price for the households.

D3: yes, it's about specific nutrition sensation, malnutrition like stunting, deworming for pregnant and under five children, Iron folate for pregnant mothers. The national nutrition policy work for preventing stunting among under five children. The nutritional counseling strategy include increment of crop production at household level, increasing household consumption of fruits, vegetables and cereals, empowering women and decreasing dependency on husband income by income generating activities, introduce trading practice among mothers. Moreover, eliminating stunting by 2030. By conducting monitoring and evaluation activities we identify acute and chronic malnutrition cases. The activities coordinated by string committee which is led by woreda administration and the technique committee (multi-sectorial focal), they meet once in a month, the technical committee will be led by woreda health office head.

D7: the multi-sectorial nutrition program, each sector has its own specific task, for example the health office will identify the severe and moderate malnutrition cases and they will be admitted to SC/OTP and MAM treatment. Creating awareness regarding nutrition at HH level, consumption of diversified food for pregnant and lactating mothers and children. The malnutrition cases identified from the community will be treated at the hospital, health center or health post based on the severity of the case. For identification of stunting growth monitoring

will done every week. Counseling, deworming, and vitamin A supplementation will be done for pregnant and lactating mother and children accordingly. Additionally, the program works on underlying cases of malnutrition; personal hygiene, environmental sanitation, safe water supply, open defecation free, increasing health service utilization and improving access to diversified food.

(I). How does this problem trend look from past to the present?

D2: the nutritional problem is increasing in alarming rate. Climatic change, conflict and shortage of hybrid maize seed and unavailability of water source are the main cause for the increment.

D1: the trend is increasing because the young farmers are not working on their farm, the unseasonal rain resulting flood, shortage of agricultural supply, and migration from rural to the urban areas.

D5: the trend is increasing due to the drought that resulted from the climate change. For example, there is a shortage of rain since 2013 which resulted increased food insecurity. The population is increasing resulting over population on already dense area.

D6: the inflation on the market is also one of the reasons for increased nutrition problem trend. The farmers did not afford to buy fertilizer and seed due to the inflation.

D3: it is increasing. Year after year the population is increasing, decreased agricultural production, resulting shortage of food which makes the vulnerable groups to beg on the streets. The community is dependent on rain. The woreda is known for maize production but the community did not have drought resistant crop like “enset” false banana. In addition, the inflation made the agricultural supplies unaffordable to the farmers which resulted further reduction in harvest.

(I). What is the nutrition related activities of your office?

D5: For example, we conduct screening, do growth monitoring, promote diversified food consumption among pregnant and lactating mothers and children, we work on behavioral change on dietary practice, we work on improving women’s economic condition, teach students on diversified food consumptions so as they teach their parents, promote appropriate dietary practice like consumption of iodized salt through different local medias. Additionally, we do deworm biannually, and targeted micronutrient supplementation, ease access to safe water supply, establishing and supporting nutritional student clubs to reach the community. Supporting the farmers by providing improved breeds dairy cattle, poultry increased access to

farmers. Increasing production and consumption of meat, milk and eggs. Creating awareness on consumption of eggs, and provide health education on diversified food consumption.

D1: mainly creating awareness for multi-sectorial nutrition program by identifying beneficiary households, under five children, pregnant and lactating women, growth monitoring, and providing health education on diversified food consumption. The program provide goat for beneficiaries in order to increase access to milk.

D7: the program is working together for common goal. The main goal of the program is creating awareness at community level through HEWs, food cooking demonstration like porridge? at health post for pregnant and lactating women, strengthening and capacity building behavioral change activities, supplementation of Vitamin A, deworming, Iron folate, ease access to safe water supply and monitoring and evaluation the progress.

(I). What are the roles and responsibilities of your office to achieve the Implementation of multi-sectorial of nutrition?

D2: our role includes providing health education on multi-sectorial nutrition program that is vitamin A, deworming, Iron folate supplementation for pregnant and lactating women and children. Screening for malnutrition and identifying severe and moderate malnutrition cases for SC/OTP treatment and provision of fafa for moderate cases.

D6: our main role is increasing irrigation practice at kebeles around lake Hawassa. Increasing technology utilization; solar pump for their irrigation, use fast harvest crop seeds. Improving dietary practice specially consumption of diversified food. Creating awareness to improve nutritional status of pregnant and lactating mothers in addition to their under-five children.

(I). Tell me the ownership of the program

D3: multi-sectorial program member sectors has their own job description. All sectors are integrated and collaboratively working with the help of woreda coordinator of the program. The technical committee of the program is led by the woreda health office head, the report compiled and send by the head of the health office. Generally, all stakeholders are ownership for the program since they are working for common goal. The whole program in the woreda is led by the honorable woreda administration.

D5: woreda health office head will lead the technical team and string committee led by woreda administrator. All sectors jointly do the planning, implementing, monitoring and evaluating, supervising, so all sectors are the owners of the program.

(I). Tell me the challenges of your office in relation to collaboration with other offices Can **you tell me detail about the procedure of program such as; about the annual plan, half year plan, quarterly plan, achievement report, meeting schedule, monitoring and evaluation related commitment.**

D7: The challenges we are facing includes budget shortage, transportation access, decreased motivation due to lack of incentives, and price inflation of supplies which resulted training programs to be cancelled. Shortage of computers resulting poor data management, only paper-based data handling. Water source unavailability in the area is also a challenge to improve agriculture since we only rely on rainfall holding the program to bring significant change. The leadership of the programs is not planning and sharing tasks on time. Shortage of agricultural supplies shortage is one of the main challenges we are facing. These challenges are holding us to improve the nutritional condition of the mother and children.

D3: the focal persons from the sectors are busy with other duties is one of the challenges. Low political commitment especially at finance office for facilitating payments. Poor awareness at community regarding the multi-sectorial nutrition program.

D5: lack of commitment at finance office. There is shortage of budget to achieve the program goal, eliminating stunting in 2030 and improving maternal health. Moreover, shortage of vehicles to conduct kebeles level field work. Lack of digitalization in data management. Community level poor commitment due to lack of awareness.

(I). What challenges are there for the community to achieve a multi-sectorial nutrition program?

D3: lack of awareness regarding multi-sectorial nutrition program, resistance to behavioral change, and could not afford consumption of diversified food.

D5: poor knowledge on the program, poor financial condition, poor commitment at household level, and poor consumption of egg even if available and food taboos.

(I). Tell me how the structure of this program is organized?

D2: each stakeholder office has focal person for multi-sectorial nutrition program. Each working integrated with partners moreover led by woreda administration made the program to be structured and well organized.

D3: presence of focal person in each office contributed to the organization. Presence of single plan, and common goal, the sectors are collaborated and integrated.

D7: multi-sectorial nutrition program works for common goal. The program has two working committees; string and technical committee. The program has collaborated household supervision. All the program activities led by political party or woreda administrator. They usually give working principles and guidance.

(I). Say something about the program in relation to the budget?

D5: we got the budget portion from the federal government but we did not receive the regional government portion. To make stunting zero at 2030, the budget shortage will make it difficult to achieve this goal.

D3: financial problem is making difficult to run the program activities smoothly. Shortage of budget for purchasing computers for data management.

D2: due to shortage of budget we selected only two kebeles with six households from each.

D1: we only got small amount of budget from federal government. We are not getting budget from the regional government. Therefore, it is difficult to conduct activities to improve nutritional and health condition of the pregnant and lactating mothers including children. In addition the economic inflation in the country are also making it difficult.

D7: due to shortage of budget we are not able to make supportive supervision at health centers and health posts. We are only working with 12 households in two kebeles due the financial problem facing the program.

(I). HR and other resource issues in your office?

D4: we did not have problem in terms of trained personnel. However, due to shortage of incentives, the staff motivation has decreased. Additionally, there is a knowledge gap due to lack of training.

D5: we did not have human resource problem but we have capacity building issue due to the shortage of budget for training.

Opportunities for this office/partner in implementing a multi-sectorial nutrition program

(I). How did the professionals who work on a multi-sectorial nutrition program capacitate?

D7: we are using the limited budget to capacitate the focal to provide updated training. Limiting other burden of job for focal, by availing logistics from partners, rewarding and sharing idea regarding the multi-sectorial nutrition program. Data handling by already existing ICT.

D3: by holding periodic discussion on multi-sectorial program. By giving specific tasks for each sector, by providing updated training on improving maternal and child nutrition and health,

by participating community in discussion, by conducting supportive supervision, availing logistics for pregnant and lactating mother forum, and conducting mentoring and coaching by focals.

(I). Have you had the consultant workshop on this program?

D6: All multi-sectorial nutrition program sectors conducted workshop by participating woreda administrations, party head, kebeles administration, health extension workers, and kebeles agriculture officer. We identify poor screening by HEWs as limitation, lack of nutrition counseling from agricultural office side, and unavailability of strong multi-sectorial activity at school level are also an obstacle.

D3: during our workshop we discussed about updated training, improving women's economic status, the importance and practice of first thousand days in nutrition.

(I). How did you involve the community to create awareness?

D7: by providing health education, community mobilization including kebeles chair person, health development army, religious and community leaders, by conducting pregnant and lactating mothers forum, conducting food cooking demonstration at the health post.

D5: teaching the community to bring social behavioral change. By participating the farmers in experience sharing from in farmers trainings centers, community mobilization, by using community influential individuals like religious leaders and elderlies. Moreover, by using broadcasting medias like Sidama radio and SMN television.

(I). What are the nutrition related programs other than multi-sectorial of your office?

D7: strengthening expanded program of immunization (for preventable disease), Vitamin A supplementation, deworming and provision of Iron folate for pregnant and lactating mothers and children are the other programs that work to improve maternal and child nutrition. Additionally, increasing health seeking behavior, creating awareness. By promoting utilization of community-based health insurance, ease affording and accessing health services.

D3: supporting pregnant and lactating women in economic empowering activities, promoting appropriate dietary practice, providing daily support and care to street children, strengthening HIV-TB linkage.

D6: providing improved traditional stoves to prevent indoor pollution particularly to pregnant and lactating mothers. By providing solar energy source for light to prevent indoor pollution.

D1: availing and increasing access to cattle food, improving hygiene and sanitation, education on harm of open defecation practice.

(I). Is there designated responsible body to coordinate the program?

D4: all focal persons are responsible for coordinating the program in their respective sectors. And they are highly committed to the program. The focals are specially focused on building knowledge.

D2: the string committee coordinate the program which made all the members to be responsible and committed to the program.

(I). What are strategic and operational plans of your office in multi-sectorial nutrition program?

D6: during outbreak, malnutrition screening, and during campaigns like deworming, Vitamin A supplementation and vaccination campaigns, community level forums, and strengthening feeding programs are our operational plans. The strategic plan of the program is making the prevalence stunting zero.

D7: working on behavioral change activities are our operational plan. Identifying severely and moderately malnourished mothers and children, growth monitoring program, data quality activities, are also the operational plans. As my colleague said before the strategic plan is to eliminate stunting from our country in 2030. Program monitoring and evaluation is also included in our operational plan.

(I). How are communities committed to support the activity plan of this program?

D4: by participating and increasing ownership of the program, the community is committed to all the nutrition related activities.

(I). Tell the presence of promising work structure of this program.

D3: the program structure is promising because it is integrated, coordinated and deep routed in all sector offices.

D1: since the program is led by the higher officials and with focal person in all sector offices it is well organized. The program will easily achieve its goal if the budget shortage problem solved.

D6: since the program is working on building economy, behavioral change, dietary habit change at community level it is promising.

(I). How is the political support of this program?

D: the program is led by the party head and the woreda administration so the support from the political side is huge.

(I). What do you think on the recommended strategy to improve the implementation of multi-sectorial nutrition program in your district/region?

D5: increasing budget, availing transportation mechanism, data digitalization, decreasing work and load from focal persons are my recommendation points.

D7: improving finance, improving integration and collaboration, improving availability of hybrid maize seed and fertilizer. Improving mechanized agricultural practice, and making internet accessible for updated information.

(I). How do you think these strategies can improve the multi-sectorial nutrition program?

D3: increasing budget for the program, hiring accountant for only multi-sectorial program activities. Digitalizing data management. Strengthening integration and collaboration, daily monitoring and evaluation.

D1: availing adequate budget from regional and federal government on time including from partners. Strengthening the support from partners; providing logistics, supplies to conduct field work.

FGD 2: FGD with sector office heads

**Section 1: socio-demographics characteristics**							
**Discussant**	**Woreda**	**Sex**	**Age**	**Marital status**	**Profession**	**Position**	**Work experience**
D1	01	Male	32	Married	Agricultural Economics	W01	13 years
D2	04	F	43	Married	Cooperative Field	Wc01	17 years
D3	01	M	35	Married	Plant science	A01	15 years
D4	02	M	30	Single	Health Profession	H01	9 years
D5	03	M	30	Married	Horti-cultural Science	A01	7 years
D6	01	F	-	Married	Business Administration	wc01	15 years

D7	03	F	28	Married	Gender Equality	Wc01	13 years

Multi-sectorial nutrition program implementation related challenges in this office/partner

Facilitator:

Our aim is to discuss the challenges of multi-sectorial nutritional programs in the region.

You are from different districts, so I would like to assess the multi-sectorial nutrition problems in your districts.

Participant D1, please say some things.

Participant from D1: Okay! Thank you! According to our district, it is known that this was supported by the federal and regional governments on the issue of food shortages. It is a major issue because there was a lack of rain, it did not rain during ploughing time, and it rained intermittently. There were safety net programs and other organizations that supported households victimized by food shortages in the past. But for the last year, this support was interrupted, and for this reason, many households faced food shortages, as we realized during the Kebeles supporting programs. A large number of populations were purchasing their food needs from the market, and a large segment of the population was migrating to the urban areas, where some of them became beggars. This is not only in Boricha District but also in other districts of the region. When we ask why they became beggars, they tell us that there was no food. A person who led his family by ploughing his farm in the past became a beggar, and for this, in my opinion, the biggest imposing factor is one interruption of rain, an interrupted supply of inputs for agricultural production like fertilizers, and selected seeds. The lack of availability of selected seeds is not only a current problem but also unclear for the next season. Generally, I conclude that the biggest problem in society, other than other problems, is food shortage.

Facilitator: Let me give a chance to B GROUP.

Participant from BG: Thank you! The big problem that led to nutritional problems was not availing selected seeds and other inputs on time. Some input is not enough for all farmers. In addition to this, scientifically, if support from specific stakeholders is not given appropriately, they will not become more productive. The most vulnerable segments of the population who faced shortages of food were children, pregnant mothers, and elderly.

The other reason that led to nutrition problem was drought. In order to decrease the impact of this problem, scientific support for vulnerable groups and stakeholders is mandatory. Which means all stakeholders play their role, which means all members of the multi-sectorial nutritional program, agricultural offices by availing necessary input like fertilizers, selected seeds, water; mineral offices by availing water; health works on the quality of food; others, accordingly, work on nutrition in order to deduce the impact on the population.

Facilitator: let me give a chance to D4.

Participant from D4: Nutritional problems can be categorized into two categories: one is the unavailability of food, the second one is the appropriate use of food.

The reason for unavailability of food was interruption of rain since farmers depend on rain for cultivation of crops. Owning a small farm is also another problem which exposes them to nutritional problems. But, a nutrition problem differs from one kebele to another within a district. Households in the same kebeles have large farms and a hardworking habit; they earn more products, but farmers with small farms are exposed to this problem. Generally, we can conclude that nutritional problems are due to a shortage of food and a missed use of available food, which means taking market high nutritional value foods in order to buy lower nutritional value foods even to have money for other purposes than feeding on them.

Facilitator: let me give a chance to D6.

Participant from D6: Thank you! In case of our district, nutritional problem is common which is known at federal level and supported for last many years. From the 14 kebeles of the district, 13 were included in the safety net program in order to get support. The reason for this was drought due to the lack of rain. Currently, those organizations that were supported in the past are not working, and this worsens the problem. For this reason, people are begging on the

streets. The other reasons that increase the burden of nutritional problems are increased population and owning a small farm. For your surprise, one father-owned farm is divided among four children; each child is born with four children, and then the farm is divided among them. Look how much it becomes too small. That is why people are going to be hungry even after working hard; therefore, working on Family planning also plays a great role in reducing this problem.

Another direction that helps to handle this problem is increasing productivity by using different mechanisms, like availing selected seeds on time, using modernized technology, and with the help of the agricultural office.

Facilitator: You are explaining well. Your idea shows that there were supply problems with selected seeds. However, there is a program called the multi-sectorial nutritional program, or Sekota Declaration. How is it working and what are the challenges?

Participant from D5: In Bilate Zuria district, it is known for drought-prone districts like Boricha, Loka Abaya, and Hawassa Zuria, which have no natural streams. Our district is separated from district called Boricha which is known by drought even globally. For the last three years farmers faced couple of problems due to damage of crops by shortage of rain. The increment in population is one issue that leads to nutritional problems. The other one is that the number of working hands decreased while the nonproductive population increased. For example, in one household, there are 10 people, of whom only two work; the rest are dormant and feed on the products of mother and father. My recommendation for this problem is cultivating crops that resist climate change and availing selected seeds on time in the region; currently, we are getting selected seeds from the Amhara and Oromia regions, which makes us unable to see on time.

Working on water sources other than direct rain, like irrigation and the health sector, must work on family planning utilization.

View of society on Sekota declaration is good, that is we are teaching the society in order to prevent stunting among children in collaboration with them. All sectors that are members of this program are working on food diversity in order to close the gap of stunting.

Thank you!

Facilitator: - let me give chance for those who not participated. D7

Participant D7: I am from the Bilate Zuria district, where the nutritional problem affects approximately 95% of the population. There are 19 kebeles in the district, all of which are part of the aiding program. The factors that enforce nutritional problems have two reasons: one is natural, which includes climate change, no natural streams, or running water.

The second one is a manmade problem, which is the war between Oromia tribes and Sidama. There are four kebeles in which children, mothers, and the whole member households are out of their residence, which makes them more vulnerable to nutritional problems.

After Sekota declaration launching society got information for about things in hand which have nutritional value. We are teaching a woman who has a hen not to sell eggs but to use it, but it needs more strengthening. However, Sekota Declaration faces financial management challenges in order to run the program.

Facilitator: You are telling me in detail, but our discussion must focus on nutritional problems.

Let me give D2 a chance.

ParticipantD2: In case of our district, there is big nutritional problem, the reason is geographic location of land; in which one is flat, the other is mountainous which exposes to degradation of soil during heavy rain season. During the sunny season, crops burned due to stones in the soil. The other one is that for the last year, populations in the district faced drought and became hungry. The habit of cultivating only one type of crop, such as maize or haricot bean, is another problem that will expose you to drought effects. Total cultivation of drought-resistant crops is needed.

Facilitator: I think it is clear to you if any exceptional idea should be raised.

Participant D4: I thank you!

The other challenge is a lack of awareness about using available food products. The coordination problem among multi-sectorial nutrition programs, including the government official's refusal to allocate budgets and low follow-up, is also a setback.

Facilitator for D5

Participant D5: Planting eucalyptus is another problem in our district, which exacerbates the drought effect on the farm. Currently, most farmers are planting eucalyptus, which absorbs underground water that is used by crops, and its pollen has a burning effect on crops. So, this needs underlining with a red color, which increases nutritional problems.

Facilitator for D7

Participant D7: When we see the trend of nutritional problems from past to present, in the past, using food lacked knowledge on its importance and relied on a single type of food; however, there were more foods in the past even if they were not used in the same type as in the present. Currently, people know about the quality of food, but they face a shortage of food due to an increased population and small farms.

Facilitator: have you ever heard about a multi-sectorial nutrition program? If your response is yes, what is its policy, system, and guiding principles?

Let me give D2 a chance.

Participant D2: Multi-sectorial nutritional program policy focuses on preventing under-two-year children from mental retardation and stunting, and it is on the governmental agenda.

Participant D4: Yes, I heard it. Currently, I am working within it, which includes different sectors, mainly six sectors, and is aimed at preventing stunting among less than two-year-old children. It works in order to achieve zero stunting in 2020 E.C.

This Sekota declaration is working in order to prohibit stunting among children by providing necessary food on time, providing safe water, and overcoming taboos that hinder nutritional activity.

Facilitator: Is there anyone else who likes to add more? To D1.

Participant D1 A multi-sectorial nutrition program is aimed at preventing stunting starting in pregnancy and includes the collaboration of a number of stakeholders in order to tackle the nutritional problem.

The policy of the Sekota Declaration is the commitment of the government to prevent stunting at the route level. This includes identifying exposed individuals and supporting them. Later on, identified individuals were also empowered, and the neighbors learned from each other.

Facilitator: What is the trend of nutritional problems from the past to the present?

Participant D3: In the past, food utilization was not based on knowledge of its importance; people fed on one type of food throughout the year. This is due to a lack of awareness about a balanced diet, even though food products are available. However, there was no problem with the availability of food because there were enough false bananas, animal products, and others. In the past, there was no problem with quantity, but despite knowledge of the quality of food, there is a shortage of food at present. This is due to an increased population, small farms, and lower productivity. Currently, we are realizing that there is a nutrition problem.

Facilitator: for D4

What were the nutrition problems in the past and present?

Participant D4: In the past, there was food and a smaller population, but balancing diet has its problems. At present, society has knowledge about diversifying food; however, there is a shortage of food.

Facilitator: What are the challenges of owning the multi-sectorial nutrition program? What are the job descriptions in your specific sector related to the multi-sectorial nutrition program? Plan? And Aim?

ParticipantD4: I will discuss what sectors perform in this program and the challenges they face. Health sector behavioral change issues related to feeding habits - how many and which type of food - are answered by the health sector. The agricultural office works on the production of foods by planting different plants in their garden. The Water and Mineral Office works on the provision of clear and safe water, which helps the population not be exposed to different health problems and enables them to be more productive. Whereas women and child affairs works on taboo tradition, which makes women and children malnourished. For example, in some areas, pregnant women do not eat cucumber because they believe that it increases the size of the fetus. They also work on early marriage, which is associated with immature delivery of children and leads to stunting.

Facilitator to D7

Participant D7: Women and Child Affairs works on awareness creation for pregnant mothers and their household members in order to feed on a variety of foods. They also work on empowering households in order to secure themselves with food by working with women to develop a culture of saving.

Facilitator: Let me give a chance to D5

Participant D5: The challenges to the ownership of this program are that the finance office of the district is a big problem, and the head of the sector has low commitment. We are working in order to bring behavioral change among the society in order to enable them to feed on a variety of food, even starting with the cultivation of different food crops.

Facilitator: On job distribution, could you say the same things related to your sector?

Participant D6: As a women and child affairs representative, our responsibility is to create awareness for women. Because women experienced different challenges in the past, like depending on their husbands for social and economic affairs and the culture of prioritizing fathers during feeding, these must be left from them, and we are struggling to come out of such harmful practices. Ideas raised earlier suggest that unbalanced diet eating habits are due to a lack of awareness or to the attitudes of women. Human beings form in 1000 days; therefore, in order to take measures against stunting, pregnant mothers must feed first. It will help them have normal growth throughout their lives. We have a structure up to the kebele level that works on the rights of children and mothers.

We are also working on empowering women by developing saving habits. Evaluation and follow-up are done continuously.

Facilitator: D1

Participant D1: I am from the water and mining office, and clear and safe water is mandatory for pregnant mothers as well as for others. Water is needed for washing, rearing animals, and agriculture.

But, we faced a shortage of budget in order to fulfil requests for materials to implement the program.

Facilitator: D2

Participant D2: Our job description focuses on 1000 days of life; this is the window-closing point for stunting. We provide training on empowering women. We had done awareness creation for Kebeles administrators and health extension workers in order to include pregnant mothers in the safety net program.

Facilitator: who is responsible for this program?

Participant D4: It is expected that all sectors own the responsibility, but in the real world, the district administrator is responsible for controlling all activities of the program. The district Sekota declaration coordinator follows the activities performed by each member office, including financial utilization.

Facilitator: let me give chance to D7.

Participant D7: as his colleague said, the general Distract administrator controls every activity that is performed in six sectors, whereas all heads of member sectors are responsible for the program. In each office, there is a head of office, and the focal person assigned to perform activities related to the program also shares responsibility.

Facilitator to D2

Participant D2: District administrator leads all six member sectors, and the health office manages the activities. The head of office arranges the activities performed by focal persons from each sector.

Facilitator: What are the challenges for coordination among member sectors? Is it during the annual plan, biannual plan, quarterly plan meeting, reporting, or monitoring and evaluation?

Participant D3: The problem for coordination among sectors is material and logistical shortages for meetings. Including financial problems for transportation and per diem costs, bide is not arranged on time. That makes us always incompatible with the district finance office.

The other problem is that allocated budgets are not released on time from the region; this extends activities performed in September to December.

Generally, it is not a coordination problem among the six member offices; rather, the finance office is our problem.

Facilitator to D4

Participant D4: We are working in collaboration with sectors, but sometimes during meetings and monitoring and evaluation time, there were problems among the majority of sectors. Another challenge is that the finance office's allocated budgets are used for other purposes and are not arranged on time for requested activities.

Participant D5: The problem is at the time of monitoring and evaluation; if work is not evaluated on time, it may not be common performance, which reduces cumulative achievement. The other challenge is not releasing the allocated budget on time.

Facilitator: What are the challenges among communities that set back the multi-sectorial nutrition program?

Let me give D1 a chance.

Participant D1: expectation of population, which is related to dependency on aid and selling supported items back; for example, last year we provided goats but sold them back.

Facilitator: let me give chance to D6

Participants D6: The community has a problem with attitudes; this is due to the fact that society learns bad attitudes from aid and does not believe that they can help themselves but rather expects ready-made food. They also think that if I have resources, I will be out of support; this is their thought, which is an obstacle to implementing the program. Generally, it is better to work on the attitudes of the population.

Facilitator: let me give chance to D4

Participant D4: For example, if we provide hens for a mother to feed on eggs, they will sell both eggs and even hens, sometimes being too laggard to accept what they think.

Facilitator: How did you report your performance?

Participant D4: Reports collected from the community are sent to each sector, then to the district administrator, region, and federal level. We use DHS2 to report reports; reports could be monthly, quarterly, biannual, or annual.

Facilitator for D3

Participants D3: We are reporting what we have done. We take reports from kebeles focal person, then compile district-level reports for higher district administrators, regional administrators, and federal administrators.

Facilitator: Could you say the same things related to the program budget? The way administration and its adequacy

To D1.

Participant D1: the program budget is very small. In the case of our water and mining offices, the price of materials is increasing on a daily basis, making it very difficult to perform activities with a scarce budget. The way of utilization is distributing budget in a compensatory way in order to handle problems faced. For example, I bought some materials and couldn't find transport budget materials there. This is why I say it is scarce.

Facilitator for D4

Participant D4: At the very beginning of budget allocation aimed at the number of activities performed in the district, the source of budget is not only from the government; it may also be from supporters and the community, which also affects the budgeting system. The long budget releasing system, which starts from the federal to district level plus allocations from regions and districts, has its own problems. The regional government is expected to allocate a budget equal to the federal government's allocation. The district is also expected to allocate a certain percent of the budget allocated by the region. But, currently, we are using only the budget allocated by the federal government of Ethiopia. That is why it is too small. So, a scarcity of budget hinders our activity.

Facilitator for D6

Participant D6: thank you! For the question, is it adequate? It is not. We are using it as "yeast." It lacks not only adequacy but also equality. I said this: if the work is done on mothers and

children, there must be an acceptable level. For example, in our district, the allocated budget to women's and children's affairs is 130,000 birr. How could this be enough for trainings at the lower level in all districts for awareness creation for mothers, elderly, and health extension workers? That is why I said it is not fair. For your surprise, this does not pay back transportation costs for participants during training.

Facilitator: Thank you, for D5.

Participant D5: The budget is very small. In the case of our agricultural office, there are 38 activities expected to be achieved, but the budget does not enable us to perform two of these. For example, in order to buy a tin of seeds for vegetables, the price had increased in the past. Despite this, the budget was reduced. For example, the budget that was allocated for Bilate Zuria district agricultural office is 328,000 ETB, which is a very small budget to accomplish 38 activities. When you see the transportation cost in order to see one kebele, which costs 200 ETB, it is expensive.

Facilitator to D2

Participant D2: Budgeting has a wide range of problems. For example, a mother who comes to take training needs entertainment, but the budget is only allocated for transport costs. No budget allocation for entertainment and other office materials

Facilitator for D3

Participant D3: Budget is very small. For example, a large number of people faced nutritional problems, but we only selected 13 due to a shortage of budget. The program has a good idea, but its budget is too small.

Facilitator: could you say the same things related to human resource issues in this program?

Participant D2: human resources is needed at the lower level, but they are not there. We try to use the women's development army structure, but it needs a focal person in order to harvest good achievements.

Facilitator: let me give a chance to D3.

Participant D3: In the case of the agricultural office, there is no human resource shortage; as mentioned earlier, we have kebeles focal person at kebele level.

Facilitator to D6

Participant D6: Thank you! For others, there are focal points at kebele level, for example, for agricultural offices, Health offices, health extension workers, and Education offices with school principals, but in the case of water and mining offices and Women and child affairs offices, there are no focal points at kebele level.

Facilitator for D7

Participant D7: women and child affairs office has a large population to serve. Not only this, but we also work for equality side by side. For example, I am delegated to accomplish this activity.

Facilitator: How could we make human resources function actively?

Participants D4: Thank you! In order to cascade this multi-sectorial nutrition program, we provide training to different stakeholders, arrange workshops, facilitate transport allowances, prepare posters, and make the working area attractive.

Facilitator for D1

Participants D1: The first thing that initiates the work force is training, but it is not provided due to budget shortages. Even the budget that was allocated for training in the case of the water office targets only beneficiaries.

Facilitator

Participant D5: it plays a crucial role in order to have competitive officer training. However, the budget is still too small. Equipping the working area with attractive materials and using sophisticated materials like tablets for reporting and allocating transportation allowance.

Facilitator for D6

Participant D6: training and refreshment training are needed to have a competitive work force; workshops and experience sharing are needed. Arranging technology-based reporting mechanisms like installing an internet connection.

Facilitator: Is a discussion workshop held for this program? If yes, who attended it?

Chance to D3.

Participant D3: Yes, I attended the discussion workshop, which was arranged by the regional government. The participants were district administrators, coordinators of the Sekota declaration, and focal person from each district. Then they trained Kebeles Das and beneficiaries.

Facilitator for D4

Participant D4

Thank you! During the program launch of the Sekota Declaration, the federal government cascaded workshops, which were done at both the regional and district levels.

Facilitator: Who were participants in the workshop? To D4.

Participant D4: The participants were sector heads of district, focal persons from sectors, and food system council chairpersons who participated in the district-level workshop.

Facilitator: How are you working to create awareness in the community about this program?

Participant D6: In order to create awareness in the community, we discussed it at the district level first, then cascaded to the kebele level.

Facilitator: Let me give a chance to D5.

Participant D5: whom we call the chairperson of the kebeles, religious elderly, and women’s league to the district center, received training from the 19 kebeles of Bilate Zuria district. And this was cascaded to lower levels in formal and informal ways.

Facilitator for D7

Participant D7: women and child affairs office, we gave training on 1000 days of life and empowering women; we gave training for elderly, women's development army leaders, and others. Thank you!

Facilitator: thank you**!** Out of these multi-sectorial nutrition programs, is there another program working on nutritional programs? What was its aim and plan?

Participant D3: There is a safety net program that works on nutrition. This works on indigents in order to overcome the problems. The other one works on availing selected seeds, which is a government program that works on nutrition.

Facilitator: let me give a chance to D5.

Participant D5: water and mining office, in collaboration with the enterprise office, created working opportunities for non-employed youths through the association of mining groups and created work opportunities.

Facilitator for D7

Participant D7: Out of this multi-sectorial nutrition program, there is Save the Children, which is a school feeding program. They also work on empowering women through saving. There is also an NGO that helps 32 vulnerable children and orphans. There is also the safety net program, which works on indigents and poor households; we conduct identification with other stakeholders.

Facilitator: To whom is the responsibility given for this multi-sectorial nutrition program? If yes, what is his or her commitment?

Let me give a chance for D4.

Participant D4: responsible bodies are the heads of member sectors, nutrition focal points from each sector, coordinators of Sekota declarations, district administrators, and generally the district food and food system council is responsible for the program.

Facilitator: Let me give a chance to D3.

Participant D3: Yes. It was given at different levels: federal level, regional level, and district level. At the district level, each head of office is responsible, focal person in sector, and kebeles focal persons are responsible.

Facilitator, what is your long-term and short-term plan for the multi-sectorial nutrition program plan in your office?

Participant D3: There are different types of planning, depending on the activities performed.

Facilitator D2

Participant D2: short-term planning involves maintaining leaking pipes, hand pipes, and line pipes.

Long-term planning includes wide-ranging water distribution to a large population.

Facilitator for D4

Participant D4: Thank you! The operational plan is most often related to the budget for the year. The other one focuses on indicators for each sector for the next five-year plan.

Facilitator: how does the community commitment and involvement in order to support this program look like?

Chance to D6.

Participant D6: community looks like their leader; they accept what is good for themselves.

Facilitator for D7

Participant D7: It is good; this program initiates the community to be involved in it. The other thing is that this program supports poor households.

Facilitator: What is the political support for this program?

Participant D3: political leaders are supporting this program, and we realize that from the region to the kebele level, there is great support.

Participant D4: it has medium political support but needs continued support, follow-up, and the allocation of enough budgets.

Facilitator: at the end, give your recommendation on how to change the challenge of this multi-sectorial nutrition program.

Participant D4: in order to change the challenges of the program, it is better to establish it as a sector. Allocating a sufficient budget.

Facilitator to D1

Participant D1: Releasing the budget on time, finance mismanagement must be solved.

Facilitator: I finished my questions. Any additional ideas?

Participant D7: Budgets are released into other accounts rather than the government account. In order to make a successful program, it needs continuous monitoring and evaluation. Necessary materials and logistics, like motorbikes, must be provided.

Participant D2: release of the budgets has its own time.

Facilitator: Thank you very much for your time.

FGD 3: FGD with sector offices heads round two

**Section 1: socio-demographics characteristics**							
**Discussant**	**Woreda**	**Sex**	**Age**	**Marital status**	**Profession**	**Position**	**Work experience**
D1	01	P02	42	Married	Economics in agriculture	H01	13 years
D2	04	P01	52	Married	Business Administration	H01	17 years
D3	01	P01	354	Married	Plant science	A01	15 years
D4	02	P02	40	Single	Health Profession	A01	9 years
D5	03	M	35	Married	Horti-cultural Science	H01	7 years

D6	01	F	47	Married	Cooperative Field	Wc01	15 years
D7	03	F	38	Married	Gender Equality	Wc01	13 years
D8	04	M	48	Married	Environmental health	W01	15 years

Facilitator: F **D: Discussants**

F: What is the nutrition related problems looks like in your districts?

D5: our district is known for the nutritional problems, especially children and pregnant women.

F: why do you think? What are the challenges?

D5: Climate problems are leading factors. This district is dry area

F: who else?

D7: Delays in providing essential seeds and inputs, coupled with insufficient quantities for farmers, hindered productivity. Lack of targeted scientific support exacerbated vulnerabilities for children, pregnant women, and the elderly, especially during droughts. To mitigate nutritional issues, a coordinated multi-sectoral approach is essential, requiring stakeholders like agricultural, mineral, and health offices to collaboratively provide fertilizers, seeds, water, and ensure food quality.

F: D8, please say something on this question

D5: our community are suffering not only nutrition problems but also water shortage which leads Nutritional problems to be the sever in this area. In addition, the reason for unavailability of food was interruption of rain since farmers depend on rain for cultivation of crops. Owning a small farm is also another problem which exposes them to nutritional problems. But, a nutrition problem differs from one kebele to another within a district. Households in the same kebeles have large farms and a hardworking habit; they earn more products, but farmers with small farms are exposed to this problem. Generally, we can conclude that nutritional problems are due to a shortage of food and a missed use of available food, which means taking market

high nutritional value foods in order to buy lower nutritional value foods even to have money for other purposes than feeding on them.

F: let me give a chance to D1.

D1: Persistent droughts and failed rains have led to nutritional crises in the district, previously supported by federal and safety net programs. Organizations that once aided these efforts are now inactive, worsening food scarcity. Population growth, especially small-scale farms passed down generations, exacerbates the issue. Family planning is crucial to alleviate the burden.

F: thank you!

D5: our district is prone to drought, like 01, 02, and 04, which lack natural streams. 01, a neighboring district, is globally known for its drought. In the past three years, farmers have faced crop failures due to insufficient rainfall. Population growth has led to nutritional issues, with fewer working hands supporting more dependents. For example, in a household of 10, only two work, while the rest rely on them.

Thank you!

F: - let me give chance for those who not participated. D7

D7: About 80% of the population faces nutritional issues. The district has 19 kebeles, all part of aid programs. The causes of these problems are both natural, like climate change and lack of water sources, and manmade, such as conflicts between the Oromia and Sidama tribes. Four kebeles have been displaced due to conflict, increasing their vulnerability.

Since the launch of the 08, the community has learned more about locally available nutritious foods. For example, women are encouraged to use eggs from their hens instead of selling them, but this effort needs more support. However, financial management challenges hinder the effective implementation of the 08.

F: Let me give D3 a chance.

D3: The district's sunny season crops were damaged by soil stones, while last year's drought led to hunger. Monoculture farming, cultivating only crops like maize or beans, increases drought vulnerability, necessitating the adoption of drought-resistant crops. The situation in the district is dire, with agriculture facing two significant threats. First, the physical damage from

stones in the soil during planting and growth stages has led to reduced crop yields. This issue requires immediate attention, such as soil tillage or stone removal, to prevent further losses.

D4: The other challenge is a lack of awareness about using available food products. The coordination problem among multi-sectorial nutrition programs, including the government official's refusal to allocate budgets and low follow-up, is also a setback.

D5: agriculture is worsening drought effects in our district. Many farmers grow it, but it absorbs underground water needed by crops, and its pollen harms them. This issue increases nutritional problems and needs urgent attention.

F: Whoelse?

D7: When we see the trend of nutritional problems from past to present, in the past, using food lacked knowledge on its importance and relied on a single type of food; however, there were more foods in the past even if they were not used in the same type as in the present. Currently, people know about the quality of food, but they face a shortage of food due to an increased population and small farms.

F: have you ever heard about a multi-sectorial nutrition program? If your response is yes, what is its policy, system, and guiding principles?

Let me give D2 a chance.

D2: Multi-sectorial nutritional program policy focuses on preventing under-two-year children from mental retardation and stunting, and it is on the governmental agenda.

D4: Yes, I heard it. Currently, I am working within it, which includes different sectors, mainly six sectors, and is aimed at preventing stunting among less than two-year-old children. It works in order to achieve zero stunting in 2020 E.C.

This 08 is working in order to prohibit stunting among children by providing necessary food on time, providing safe water, and overcoming taboos that hinder nutritional activity.

F: Is there anyone else who likes to add more? To D1.

D1 A multi-sectorial nutrition program is aimed at preventing stunting starting in pregnancy and includes the collaboration of a number of stakeholders in order to tackle the nutritional problem.

The policy of the 08 is the commitment of the government to prevent stunting at the route level. This includes identifying exposed individuals and supporting them. Later on, identified individuals were also empowered, and the neighbors learned from each other.

F: What is the trend of nutritional problems from the past to the present?

D3: In the past, food utilization was not based on knowledge of its importance; people fed on one type of food throughout the year. This is due to a lack of awareness about a balanced diet, even though food products are available. However, there was no problem with the availability of food because there were enough false bananas, animal products, and others. In the past, there was no problem with quantity, but despite knowledge of the quality of food, there is a shortage of food at present. This is due to an increased population, small farms, and lower productivity. Currently, we are realizing that there is a nutrition problem.

F: for D4

What were the nutrition problems in the past and present?

D4: In the past, there was food and a smaller population, but balancing diet has its problems. At present, society has knowledge about diversifying food; however, there is a shortage of food.

F: What are the challenges of owning the multi-sectorial nutrition program? What are the job descriptions in your specific sector related to the multi-sectorial nutrition program? Plan? And Aim?

D4: I will discuss what sectors perform in this program and the challenges they face. Health sector behavioral change issues related to feeding habits - how many and which type of food - are answered by the health sector. The agricultural office works on the production of foods by planting different plants in their garden. The Water and Mineral Office works on the provision of clear and safe water, which helps the population not be exposed to different health problems and enables them to be more productive. Whereas women and child affairs works on taboo tradition, which makes women and children malnourished. For example, in some areas,

pregnant women do not eat cucumber because they believe that it increases the size of the fetus. They also work on early marriage, which is associated with immature delivery of children and leads to stunting.

D7: Women and Child Affairs works on awareness creation for pregnant mothers and their household members in order to feed on a variety of foods. They also work on empowering households in order to secure themselves with food by working with women to develop a culture of saving.

F: Let me give a chance to D5

D5: The challenges to the ownership of this program are that the finance office of the district is a big problem, and the head of the sector has low commitment. We are working in order to bring behavioral change among the society in order to enable them to feed on a variety of food, even starting with the cultivation of different food crops.

F: On job distribution, could you say the same things related to your sector?

D6: As a women and child affairs representative, our responsibility is to create awareness for women. Because women experienced different challenges in the past, like depending on their husbands for social and economic affairs and the culture of prioritizing fathers during feeding, these must be left from them, and we are struggling to come out of such harmful practices. Ideas raised earlier suggest that unbalanced diet eating habits are due to a lack of awareness or to the attitudes of women. Human beings form in 1000 days; therefore, in order to take measures against stunting, pregnant mothers must feed first. It will help them have normal growth throughout their lives. We have a structure up to the kebele level that works on the rights of children and mothers.

We are also working on empowering women by developing saving habits. Evaluation and follow-up are done continuously.

D1: I am from the water and mining office, and clear and safe water is mandatory for pregnant mothers as well as for others. Water is needed for washing, rearing animals, and agriculture.

But, we faced a shortage of budget in order to fulfil requests for materials to implement the program.

D2: Our job description focuses on 1000 days of life; this is the window-closing point for stunting. We provide training on empowering women. We had done awareness creation for Kebeles administrators and health extension workers in order to include pregnant mothers in the safety net program.

F: who is responsible for this program?

D4: It is expected that all sectors own the responsibility, but in the real world, the district administrator is responsible for controlling all activities of the program. The district Sekota declaration coordinator follows the activities performed by each member office, including financial utilization.

F: let me give chance to D7.

D7: as his colleague said, the general Distract administrator controls every activity that is performed in six sectors, whereas all heads of member sectors are responsible for the program. In each office, there is a head of office, and the focal person assigned to perform activities related to the program also shares responsibility.

D2: District administrator leads all six member sectors, and the health office manages the activities. The head of office arranges the activities performed by focal persons from each sector.

F: What are the challenges for coordination among member sectors? Is it during the annual plan, biannual plan, quarterly plan meeting, reporting, or monitoring and evaluation?

D3: The problem for coordination among sectors is material and logistical shortages for meetings. Including financial problems for transportation and per diem costs, bide is not arranged on time. That makes us always incompatible with the district finance office.

The other problem is that allocated budgets are not released on time from the region; this extends activities performed in September to December.

Generally, it is not a coordination problem among the six member offices; rather, the finance office is our problem.

F: let me give chance to D4

D4: We are working in collaboration with sectors, but sometimes during meetings and monitoring and evaluation time, there were problems among the majority of sectors. Another challenge is that the finance office's allocated budgets are used for other purposes and are not arranged on time for requested activities.

D5: The problem is at the time of monitoring and evaluation; if work is not evaluated on time, it may not be common performance, which reduces cumulative achievement. The other challenge is not releasing the allocated budget on time.

F: What are the challenges among communities that set back the multi-sectorial nutrition program?

Let me give to D1 a chance.

D1: expectation of population, which is related to dependency on aid and selling supported items back; for example, last year we provided goats but sold them back.

F: let me give chance to D6

D6: The community has a problem with attitudes; this is due to the fact that society learns bad attitudes from aid and does not believe that they can help themselves but rather expects ready-made food. They also think that if I have resources, I will be out of support; this is their thought, which is an obstacle to implementing the program. Generally, it is better to work on the attitudes of the population.

F: let me give chance to D4

D4: For example, if we provide hens for a mother to feed on eggs, they will sell both eggs and even hens, sometimes being too laggard to accept what they think.

F: How did you report your performance?

D4: Reports collected from the community are sent to each sector, then to the district administrator, region, and federal level. We use DHS2 to report reports; reports could be monthly, quarterly, biannual, or annual.

D3: We are reporting what we have done. We take reports from kebeles focal person, then compile district-level reports for higher district administrators, regional administrators, and federal administrators.

F: Could you say the same things related to the program budget? The way administration and its adequacy

To D1.

D1: the program budget is very small. In the case of our water and mining offices, the price of materials is increasing on a daily basis, making it very difficult to perform activities with a scarce budget. The way of utilization is distributing budget in a compensatory way in order to handle problems faced. For example, I bought some materials and couldn't find transport budget materials there. This is why I say it is scarce.

F: D4

D4: At the very beginning of budget allocation aimed at the number of activities performed in the district, the source of budget is not only from the government; it may also be from supporters and the community, which also affects the budgeting system. The long budget releasing system, which starts from the federal to district level plus allocations from regions and districts, has its own problems. The regional government is expected to allocate a budget equal to the federal government's allocation. The district is also expected to allocate a certain percent of the budget allocated by the region. But, currently, we are using only the budget allocated by the federal government of Ethiopia. That is why it is too small. So, a scarcity of budget hinders our activity.

D6: thank you! For the question, is it adequate? It is not. We are using it as "yeast." It lacks not only adequacy but also equality. I said this: if the work is done on mothers and children, there must be an acceptable level. For example, in our district, the allocated budget to women's and children's affairs is 130,000 birr. How could this be enough for trainings at the lower level in all districts for awareness creation for mothers, elderly, and health extension workers? That is why I said it is not fair. For your surprise, this does not pay back transportation costs for participants during training.

D5: The budget is very small. In the case of our agricultural office, there are 38 activities expected to be achieved, but the budget does not enable us to perform two of these. For example,

in order to buy a tin of seeds for vegetables, the price had increased in the past. Despite this, the budget was reduced. For example, the budget that was allocated for Bilate Zuria district agricultural office is 328,000 ETB, which is a very small budget to accomplish 38 activities. When you see the transportation cost in order to see one kebele, which costs 200 ETB, it is expensive.

D2: Budgeting has a wide range of problems. For example, a mother who comes to take training needs entertainment, but the budget is only allocated for transport costs. No budget allocation for entertainment and other office materials

D3: Budget is very small. For example, a large number of people faced nutritional problems, but we only selected 13 due to a shortage of budget. The program has a good idea, but its budget is too small.

F: could you say the same things related to human resource issues in this program?

D2: human resources is needed at the lower level, but they are not there. We try to use the women's development army structure, but it needs a focal person in order to harvest good achievements.

D3: In the case of the agricultural office, there is no human resource shortage; as mentioned earlier, we have kebeles focal person at kebele level.

D6: Thank you! For others, there are focal points at kebele level, for example, for agricultural offices, Health offices, health extension workers, and Education offices with school principals, but in the case of water and mining offices and Women and child affairs offices, there are no focal points at kebele level.

D7: wc01has a large population to serve. Not only this, but we also work for equality side by side. For example, I am delegated to accomplish this activity.

F: How could we make human resources function actively?

D4: Thank you! In order to cascade this multi-sectorial nutrition program, we provide training to different stakeholders, arrange workshops, facilitate transport allowances, prepare posters, and make the working area attractive.

D1: The first thing that initiates the work force is training, but it is not provided due to budget shortages. Even the budget that was allocated for training in the case of the water office targets only beneficiaries.

D5: it plays a crucial role in order to have competitive officer training. However, the budget is still too small. Equipping the working area with attractive materials and using sophisticated materials like tablets for reporting and allocating transportation allowance.

D6: training and refreshment training are needed to have a competitive work force; workshops and experience sharing are needed. Arranging technology-based reporting mechanisms like installing an internet connection.

F: Is a discussion workshop held for this program? If yes, who attended it?

D3: Yes, I attended the discussion workshop, which was arranged by the regional government. The participants were district administrators, coordinators of the 08, and focal person from each district. Then they trained Kebeles Das and beneficiaries.

D4: Thank you! During the program launch of the 08, the federal government cascaded workshops, which were done at both the regional and district levels.

F: Who were participants in the workshop? To D4.

D4: The participants were sector heads of district, focal persons from sectors, and food system council chairpersons who participated in the district-level workshop.

F: How are you working to create awareness in the community about this program?

D6: In order to create awareness in the community, we discussed it at the district level first, then cascaded to the kebele level.

F: Let me give a chance to D5.

D5: whom we call the chairperson of the kebeles, religious elderly, and women’s league to the district center, received training from the 19 kebeles of 03. And this was cascaded to lower levels in formal and informal ways.

D7: women and child affairs office, we gave training on 1000 days of life and empowering women; we gave training for elderly, women's development army leaders, and others. Thank you!

F: thank you**!** Out of these multi-sectorial nutrition programs, is there another program working on nutritional programs? What was its aim and plan?

D3: There is a safety net program that works on nutrition. This works on indigents in order to overcome the problems. The other one works on availing selected seeds, which is a government program that works on nutrition.

D5: water and mining office, in collaboration with the enterprise office, created working opportunities for non-employed youths through the association of mining groups and created work opportunities.

D7: Out of this multi-sectorial nutrition program, there is 06, which is a school feeding program. They also work on empowering women through saving. There is also an NGO that helps 32 vulnerable children and orphans. There is also the safety net program, which works on indigents and poor households; we conduct identification with other stakeholders.

F: Who is responsible for this multi-sectorial nutrition program? If yes, what is his or her commitment?

D5: The heads of member sectors, nutrition focal points from each sector, coordinators of 08, district administrators, and generally the district food and food system council is responsible for the program.

F: D3 say something regarding this.

D3: Training was provided at various levels — federal, regional, and district. At the district level, office heads, sector focal persons, and kebele focal persons are responsible.

F, Tell me long-term and short-term plan for the multi-sectorial nutrition program plan in your office?

D2: short-term planning involves maintaining leaking pipes, hand pipes, and line pipes. Long-term planning includes wide-ranging water distribution to a large population.

D4: Thank you! The operational plan is most often related to the budget for the year. The other one focuses on indicators for each sector for the next five-year plan.

F: how does the community commitment and involvement in order to support this program look like?

D6: community looks like their leader; they accept what is good for themselves.

D7: It is good; this program initiates the community to be involved in it. The other thing is that this program supports poor households.

F: What is the political support for this program?

D3: political leaders are supporting this program, and we realize that from the region to the kebele level, there is great support.

D4: it has medium political support but needs continued support, follow-up, and the allocation of enough budgets.

F: Thank you, all!

FGD 4

Interviewer: I R: Respondents

FGD 4: Discussion with coordinators of NGO who are working on nutrition

**Section 1: socio-demographics characteristics**							
**Discussant**	**NGO name**	**Sex**	**Age**	**Marital status**	**Profession**	**Position**	**Work experiences**
R1	07	P02	45	Married	Marketing	C01	3 years
R2	05	P02	50	Married	Public health	C01	10 years
R3	06	P02	38	Married	Public health	C01	11 years
R4	08	P02	40	Single	Nutritionist	C01	2 years

R5	07	P02	43	Married	environmental health	Wa01	11 years
R6	06	P02	35	married	Public Health	O01	12 years

I: How you explain the nutritional problem in your work catchment area?

R3: Our work includes seven districts in Sidama region and each district has big nutrition problem, and food shortage. Pregnant women and children are severely affected

I: Can you say more, please?

R2: We work on children as well as now a day we are working to empower the women. We organize different types of women in group and provide them money as starter. Then they work together, share the profit together. There are some model groups.

I: Do this groups of women include pregnant women?

R5: Yes, for example from old groups of women who become the model in Boricha woreda more than half are pregnant women. They were undernourished at the beginning but now they have everything. We are happy for this. Some of the group members have become pregnant after they joined the group.

I: anyone else who would like to add?

R6: I am officer to organize such groups of women to empower themselves. I am sorry to say, many pregnant women are not equally served by the multisectoral nutrition program in different districts of the Sidama region. Even there are variation from districts to districts. The program officers for the multisectoral nutrition program are friends of mine and they told me that they give priority to the children than pregnant women.

(I). Have you heard about the multi-sectorial nutrition program?

R4: yes, we heard but not working in that program. The regional government program, not our program. We sometimes invited to attend the conference or some types of workshops. Indirectly we are the member of multisectoral nutrition program of the region.

R3: yes, multi-sectoral nutrition program assigns specific tasks to each sector. The health office identifies and treats severe and moderate malnutrition cases through SC/OTP and MAM treatment. It raises awareness about nutrition, promotes diversified diets for pregnant and lactating mothers and children, and treats malnutrition cases at hospitals, health centers, or health posts based on severity. Stunting is monitored weekly, and counseling, deworming, and

vitamin A supplementation are provided. The program also addresses underlying issues like hygiene, sanitation, safe water, open defecation, health service access, and food diversity.

R5: Yes, it is all about working on undernutrition.

I: say more about it

R5: As you know, in Ethiopia '08 which works on stunting, wasting and undernutrition on lives in the first 1000 days of lives. It is multisectoral nutrition program. Our office is working on the societal problem, one of which is underlining causes of undernutrition. We work commonly water and sanitation problem of the community.

I: Anyone else?

R2: yes, we heard about it

(I). How does this problem trend look from past to the present?

R2: the nutritional problem is increasing from time to time. Previously, there were no such malnourished pregnant women as that of the children but now in rural area people have high food insecurity.

I: R1 I think you have something to say on this.

R1: Yes, the trend is rising because young farmers are not working on their farms, unseasonal rains are causing floods, there is a shortage of agricultural supplies, and people are migrating from rural to urban areas.

I: who else?

R5: For example, there is a food insecurity since 2013 which resulted increased undernutrition. The population number is increasing from time to time and densely settled in the area.

R6: the underlining problems of undernutrition is sanitation problem and water shortage in some districts in Sidama region. For this, the problem the trend is increasing from time to time.

R3: it is increasing. Due to food insecurity, or food shortage the trend of undernutrition is increasing from time to time

(I). What is the nutrition related activities of your office?

R5: Our office has both short- and long-term plan on the sanitation and water related activities in the four districts of the Sidama and southern Ethiopia regions of Ethiopia.

R2: 05 purely work on nutrition in collaboration with agricultural and health sector office.

R4: The program works collaboratively toward a common goal of raising community awareness through HEWs, conducting food cooking demonstrations like porridge preparation at health

posts for pregnant and lactating women, and strengthening behavioral change activities. It also focuses on providing Vitamin A supplementation, deworming, and iron-folate, improving access to safe water, and monitoring and evaluating progress.

(I). What are the roles and responsibilities of your office to achieve the Implementation of multi-sectorial of nutrition?

R2: We do have clear role in this regard. We discharge our routine activities. However, as multisectoral nutrition program, we have no written role and responsibilities but our work in directly related to the nutrition program in the region and we are working on the nutrition of the children and the pregnant and lactating women in the region

R6: we are working to improve nutritional status of pregnant and lactating mothers in addition to their under-five children.

(I). Tell me the ownership of the multisectoral nutrition program

R3: In the woreda, all sectors are seamlessly integrated, collaboratively operating under the guidance of the program's woreda coordinator. The technical committee, spearheaded by the head of the woreda health office, diligently compiles and dispatches reports. Ownership and commitment to the program are shared amongst all stakeholders, as they collectively strive towards a unified objective. Ultimately, the woreda administration, led by an esteemed figure, oversees the entire program, ensuring its smooth operation and success.

R5: In the woreda, the health office head energetically steers the technical committee, with the woreda administrator at the helm. Collaboratively, all sectors zealously engage in comprehensive planning, execution, supervision, and appraisal activities.

(I). Tell me the challenges of your office in relation to collaboration with other offices.

R6: Faced with budget constraints, limited transportation, low motivation without incentives, rising supply costs leading to training cancellations, computer scarcity causing inadequate data management, and unreliable rainfall affecting agriculture, our programs struggle. Delayed and poorly planned leadership exacerbates these issues, particularly the critical shortage of agricultural supplies, all of which hinder efforts to enhance maternal and child nutrition.

R3: One significant challenge is that key individuals from various sectors are preoccupied with other responsibilities. There is also a notable lack of political commitment, particularly within the finance office, which hampers the facilitation of necessary payments. Additionally, there is a deficiency of awareness at the community level about the multi-sectorial nutrition program.

R5: There is a lack of commitment, insufficient budget to achieve the program's goals of eliminating stunting by 2030 and improving maternal health. Additionally, there is a shortage of vehicles for fieldwork at the kebele level and a lack of digitalized data management. At the community level, poor commitment due to limited awareness further hinders progress.

(I). What challenges are there for the community to achieve a multi-sectorial nutrition program?

R3: poor focus on the pregnant women

R5: poor awareness and integration with governmental organizations.

(I). Tell me how the structure of this program is organized?

R2: there is no clear structure. I mean, they work in the regional office. If needed they contact us to attend the meeting. There is no clear structure.

R3: absence of one plan together to address nutrition related problems.

R4: The multi-sectoral nutrition program operates with a shared goal and has two main committees: the steering committee and the technical committee. It conducts collaborative household supervision, and all activities are overseen by the political party or woreda administrator, who provide guidance and working principles.

Opportunities for this office/partner in implementing a multi-sectorial nutrition program

(I). Have you had the consultant workshop on this program?

R6: Multi-sectorial nutrition programs held workshops with local administrations, officials, and health workers. Poor HEW screening, insufficient agricultural counseling, and weak school-level multi-sectoral efforts were noted as barriers.

R3: yes, we had consultant workshop and the following issues raised as there are important opportunities to improve the implementation of multisectoral nutrition program. These opportunities are not yet properly utilized. They include availability of churches and mosques, community centers like edir and ekubs.

(I). How did you involve the community to create awareness?

R4: so far, we involve the community in different ways, for example, invitation in food demonstration program

R1: Educate communities for behavioral change through farmer experiences in training centers, mobilization with influential locals, and broadcasting via Sidama radio and SMN TV.

(I). Tell the presence of promising work structure of this program.

R3: the program structure is promising because it is integrated, coordinated and deep routed in all sector offices.

R1: involvement of higher officials itself is the opportunity

R6: Involving the many sectors

(I). What do you think on the recommended strategy to improve the implementation of multi-sectorial nutrition program in your district/region?

R5: Having the improved budget allocation, working together both NGO and government organization to minimize the insufficiency of the budget

R3: improving integration and collaboration

(I). How do you think these strategies can improve the multi-sectorial nutrition?

program?

R3: no doubt where there is the integration of these organization work activities and budget, it will be improved obviously.

I: Thank you very much for your critical feedback and time!!
