# Supplementary material for: Exploring the challenges and opportunities of multisectoral nutrition programme in Ethiopia: A qualitative study on combating undernutrition during pregnancy
Source: PLoS One. 2025 Jul 3;20(7):e0311336. doi: 10.1371/journal.pone.0311336 (PMC12225801; doi:10.1371/journal.pone.0311336)
Supplement: S3 Data — (PDF) [file pone.0311336.s004.pdf]

Interview I: with 02's A01

I1: Interviewer, Observant; O R: Respondent

I: You are selected deliberately since you are multi-sectorial nutrition program workers who work as a leader in the agricultural sector. The information that you provide will be used for this research purpose only and will be placed in a closed cabinet. Questionnaires may take not more than 30 minutes and for the questions that you may not want to respond to, you can skip them and if you have any issues that may lead you to interrupt; you can interrupt and restart it. But we considered that the information that you provided will be valuable and useful.

Do you agree?

R: Yes, No problem

I: Thank you. So, we discussed our informed consent like during multi-sectorial implementation, challenge, opportunity, and some recommendation. We discussed that he is and you can continue!!

I: … ahh!! I am going to start my main questions. Would you tell me about yourself, mentioning the region and woreda too?

R: I am A01 and natural resource office.

I: - What is your age?

R: - I am 39 years old.

I: - Marital status?

R: - Married.

I: - Your profession?

R: - Plant science

I: Position?

R:-A01

I: Work experiences?

R: Do you mean that in this position or all work experience?

I: All experience.

R: A total work experience of 18 years.

What about in this position experience?

As office head for 0ne year and vice head for 7 seven years.

I:-Would you tell us about multi-sectorial nutritional challenges?

R: - 02 are one woredas from Sidama region which is susceptible to nutritional problems and our community cannot get balanced diet even if challenged to have staple food. Especially, for the last two years due to environmental change, the nutritional problem becomes worsened and in addition to this many children and mothers affected by other problems related to nutritional problems. To avert this problem region support our woredas by providing meal (alm-migib) but it is not sufficient to solve the problems.

I: Probe; is this problem uniformly distributed? Or a special place/kebele/village?

R: Problem severity is not uniformly distributed but the problem is throughout woredas kebele. The most affected area is a town by this problem. We have two kinds of places: Areas above Stone affected due to air condition or environmental change, community severely affected and areas below Stone less affected(this place is a cash-crop area produces khat, coffee…etc) but this is not for all households and we cannot say all household can get enough amount of money.

I - Have you heard about multi-sectorial nutrition programs, working on nutritional system programs?

R:-Yes, not only heard but also participate in it. I participated in meetings at Woredas and regional levels on the multi-sectorial nutritional program. At this, the Woredas we are working on this issue attentively since we are string committee members and with different sectors review our activities based on the share that we have on this issue.

I:- What kind of policy system for multi-selector nutritional programs do you have?

R:-Related to this we know the 07, concerned sectors agreed and signed for their activities at the national, regional and woreda levels. At this time we discussed especially related to stunting, care provided within 1000 days on behalf of health sectors. On behalf of agriculture, we work on

foods/meals required for pregnant women and after delivery til 1000 days. Water and energy offices also focus on clean water supply, and animal and fish offices focus on balanced diet provision.

I:-What are the activities of women and children's offices?

R: - Screening those affected groups.What are the problem-level trends of the Multi-sectorial nutrition program?

R:- Two Years back in 02 woreda nutritional problem was not severe, and this woreda agricultural system is known as ‘Badhesa’ (based on natural rain), cereals were destroyed due to air condition changes within the past two years and nutritional problem become severe. As 02 leaders, performed a nutritional need assessment at the woreda level, and the finding was 58,000 people need food support in March (rain based harvesting/farming time), and 28000 people need food support at ‘Goire’ when we see generally food support need to be increased in last two years when we compare with before two years. This year the air condition is so good and we expect that the condition become good and we will see together.

R: - According to your offices, what is your aim and plan related to nutritional program system?

R: - Our aim according to our office and woreda is to secure all households for their food needs, and their plan is to develop productivity and maintenance of a stable workforce.

R:- Your office's responsibility on the Multi-sectorial nutrition program is as you said providing a balanced diet for the community. Would you tell me the leaders for the Multi-sectorial nutrition program system?

R:- For this multi-sectorial nutrition program main leader is the String committee leader who is the woreda administrator. The health sector receives a report from different sectors and facilitates review programs with other sector reports and this is led by Woreda administrator.

R:- What are the challenges for your office to work with other sectors on this program? Like planning, review meeting, and supportive supervision time….

R:- Still no problem identified while we are working/reviewing our activities. Our communication (different sectors) is only during report review time. As an office, they perform their activities by

their staff alone and they meet at the time of the review meeting and they have no problems/challenges.

Q:- For the community what are the pitfalls to attaining a multi-sectorial nutrition program?

R:- At the lower level we selected very poor people (people who can not get a balanced diet) for this program, and there is a land shortage to produce a variety of commodities(like carrots, papaya, red potato, and money shortage to stay doing their farmland.

Q:-What about reporting? Is it all sectors collected to report to woreda administrators? Or report to the region? or how is the system?

R:- Woreda adminstrator review the activities and all sectors have their focal person and report to Woreda health office. Woreda health office report to the regional health bureau nutrition focal person. Woreda agricultural office also provides reports to the regional agricultural focal person.

Q:- What is the roles and responsibility of the focal person?

R:- The roles and responsibility of focal person is serves as bridge/communication between sectors.

Q:- What do you say about program budget?

R:- It is complicated, budget allocated from federal and region but is not conducive to implementation. B/s they have many activities tittles and has little amount of money which is not conducive. For purchasing also not conducive. Others issues budget disbursement is not on-time or it is disbursed at the end of budget year and not proper for the implementation specially water bore-hall drilling. There is time to prepare water hole and it is better to allocate budget at the beginning of budget year. Other issues is money allocated for administrative issues is not adequate. For farming activities budget not allocated for fertilizer. Budget at this time managed according to action plan developed buy federal/region provided.

Q:- What about HR adequacy?

R:- We have focal person on the human resources and we are working on this issues as part of sector plan. We have six professional specifically for this program. We have budget shortage but no human resource shortage.

Q:- How can we equip human resources on this issues?

R:- Previously I said, we have many human resources but training and orientation should be given and no training, workshops at all level of sectors.

Q:- who is prepare workshops for mult-nutritional program? Who was participant?

R: Last year Federal collaborated with region prepare mult-sectorial nutrition program workshop and different sector leaders participated.

Q:-What are the nutrition related programs at your office other than multi-sectorial nutrition program?

R:- Saftnet program since our woreda is benefit of SafetyNet.

Q:-What is aim of SafetyNet?

R:- The SafeNet program can be classified into two categories:- some get benefit by working and others benefited without working they may be disabled and can't perform job. The main of this program is also to secure their food need. Sometimes government was provided aid/support but now it is interrupted.

Q:- Is there responsible body nominated to coordinate the saft-net program? How is the commitment?

R:- Yes we are designated to for this program and we are committed to address the to the community. Also we are working on to increases productivity and stable work force.

Q:-What are strategic and operational plans of your office in multi-sectorial

nutrition program

R: Operational plan to secure balanced diet for all selected villages by increasing productivity.

Strategic plan even if the this program phase-out, sustainability of food security by expanding to all woreda kebeles by incorporating mult-sectorial nutrition program within our plan

Q:-.How do you think the community commitment to support this program?

R: Community members express their commitment to support this program.

Q:-How is the political support?

R:- Government support this program and woredsa administrator is string committee leader and support the program.

Q:-What do you recommend federal, region and woreda to improve the program?

R: - To scale-up to all woreda kebeles/community, and to all woreda at regional level. This program should consider all woredas and not only SafeNet woreda. Budget issues should be solved specially for capacity building. Budget should be flexible and should be disbursed timely.

Q:- What you can add for this program

R:- It is better scale-up the program to other woredas and including non-SafeNet woredas.

Interview II: with 02 district, ANF

Interviewer (I) and Respondent (R)

Interviewer (I): You are selected to participate in this study intentionally since you are working on the multi-sectorial implementation of nutrition. We consider the information that you provide useful and valuable for this research. Questionnaires will take 30 to 45 minutes and your participation will only be based on your willingness. You have the right to choose not to take part in this study and you have the right to stop at any time. This information was used only for this research purpose and no one accessed it except the principal researcher for the research objectives. Collected data will be placed in a closed cabinet.
Do you agree to participate in this study?

Respondent (R): Yes I agree

I: He said yes I agree and you can proceed.

R; YES
I:- How old are you?
R:- 35 Years old
I:- Marital status
R:- Married
I:- Profession
R:- Extension Communication professional
I:- Position
R:-ANF
I- Work experience
R: - 15 years of work experience.
I: - Tell me about nutritional problems in your woreda.
R: - Due to drought and air condition change, nutrition problems happened.
I:- Would you tell me nutritional problems at the level of kebeles/villages?
R:- Our woredas contained 22 villages and last year we worked specifically on 4 villages. Especially, on under 2 years children, pregnant women and lactating women. This year we are working on two villages we are working.
I: - Have you heard about the multi-sectorial nutrition program?
R: - Yes, we are working on these activities with the women and child affairs office, health offices as a lead office, water office, animal and fish office, and finance office work on stunting reduction.
I:- How this problem trend looks from the past to the present?
R: - Nutritional problem is not uniform throughout woredas kebele. Due to air conditions changing especially for those farmers who live low socio-economic levels, are most affected. Now we are started on capacity building/creating awareness on the reduction of stunting. Previously, communities were not aware of stunting and now there is an improvement. Due to knowledge gaps to prevent stunting, it occurred but all things are

at hand to prevent. But now this problem is solved by creating awareness by proving training.
I: Would you make it more clear that how it was and how it is happening now?
R: Previously, there was high awareness problem. But currently there is better awareness. Accordingly there is improvement.
I:- What are the activities of your office on nutrition?
R:- since our office is agricultural office, our focus is providing food.
I: What are the aims of your activity plan?
R: would you make me clearer?
I: There is sector role in multisectorial program?
R: Different sectors has different aims. For instance our office aimed at farming, and different fruits and ultimately to provide food. Livestock focus on Animal and fishery office works on providing egg, milk, meat, etc. general food has six components. cereals, legume plants, etc by agriculture. Proteinase food provision is led by livestock. When we do this we need water which is facilitated by water office, knowledge creation by education offices, protecting the health of children related to health offices, and women and children offices focus on women and children issues.
I:- How is the ownership of this multi-sectorial nutrition program? Who is the coordinator of the program?
R: - Rural women and children desk. Program coordinated by me on behalf of 08 which is under the agriculture office.
I: - What are the challenges of your office regarding collaboration with other Offices?
R: - Logistic shortage and this program not separately leaded, and time not provided daily focus on this program. In addition there is time shortage. Because we are working agricultural work as my duty or job description, I have no nutrition focused allocated time. I work jointly.
I: - Is there any problem related to the annual plan, half-year plan, quarterly plan, or meeting schedule?
R:-At the woreda level, we have a string committee that includes a woreda administrator, and sector administrators and we are a technical team. Working together with them, they are political personnel and a work burden so, we can’t move forward as we plan.
We reviewed our report with health offices and we have no problem related to this.
I: - What are the community problems to attaining a multi-sectorial Nutrition program?
R: - community dependence on professionals. Preparedness for community problems and no problem related to acceptance.
I: - How is the structure of a multi-sectorial program structured?
R: - Every office at the woreda level is in line with its structure, from region to the woreda. For instance we are agricultural office at the woreda level to the agricultural bureau of the region, health officials of woreda to the health bureau of the region and the like. Water office also similarly. But the main owner or led by health office. We are supporting body to them. This line structure is up to the kebele/village level.
I: - Say something about the program about the budget

R: - Budget breakdown at a higher level and this needs correction since budget/activities breakdown should be by implementers at a lower level. The allocated budget is also not enough, and the time to perform some activities is not feasible like water bore-hall drilling which is appropriate in summer time but they request to drill in winter time. This is not proper. Budget disbursement is not timely. It should be (planning and budget breakdown from bottom to up),
I: - What looks like HR?
R: - Human resources are not uniformly distributed within kebeles and that much is not sufficient. Kebele/village workers are not capable. There is shortage of human resources in different villages.
I: - How can we capacitate the professionals who work on a multi-sectorial nutrition program?
R: - There should be program-specific professionals and coordinators. (Professional should only focus on multi-sectorial specific)
I; what are opportunities for training, and workshops?
R: - there were opportunities and the region also provides training but it is not satisfactory.
I: - Have you had the consultant workshop on this program?
R: - YES; at the time of the beginning of the year, participants were HEW, kebele admn, education director, women and children office and prepared by the health office.
I: - How do you involve the community to create awareness?
R:-By providing training. Everything is at hand within the community and they can prevent stunting if they get awareness.
I: - Is there a responsible body to coordinate the multi-sectorial nutrition program?
R:- Main program leader within is woreda administrator, there are also six sector offices leader, and under each office one focal person, when we come to kebele level there are health development armies and health extension workers who have responsibilities in this program.
I: - What are the other nutrition programs in your office rather than the multi-sectorial nutrition program?
R: - Yes, they aim to increase coverage of food security of farmers/community.
Q: - What are the strategic and operational plans of your office in multi-sectorial Nutrition program?
R:-Yes, a strategic plan is to increase secure farmers' food security; the operational plan is taken from strategic.
Q:- What are promising work structures for multi-sectorial nutrition programs?
R:-Promising is this agricultural activity and as an agricultural office, we have three directorates working on it.
Q:- How the community is committed to supporting the multi-sectorial nutrition program activities?
R: - Community members work on food production, and if they can’t farm they can’t feed their families even if they face challenges.
I:- What is the political support for this multi-sectorial program?
R:- Yes there is political support. There are different ‘gimbar’ ‘key mesino gimbar, badhesa gimbar’ ‘Mehere gimbar’ Mesino gimbar for this community mobilization at a lower level by political leaders.

I:- What do you recommend the strategy to improve the multi-sectorial nutrition program implementation at the woreda/region level?
R:-My recommendation for the region is timely disbursement of budget, proper amount budget allocation for the activities and plan should be from the lower level (not only sending from the higher but it should be context considered manner. For woredas sectors, my recommendation is to provide special attention.
I: Do you have sector specific recommendation or strategy?
R: For instance, health offices screen malnutrition timely, as agriculture offices better focus nutrition specific food and animal and fish offices work in those screened communities. Water offices should identify places for water bore-hall drilling timely and provide safe water. As an education office, they should work on awareness creation. Women and Child affair office should work with health offices to screen malnutrition.
I: - What strategies do you recommend to improve the multi-sectorial nutrition Program?
R: - First-level planning and review plan timely.
I: - do you have any additional point to add?
R:-For this program specific attention should be given and its separate office.

I: I would like to thank you for your answer and time. I give time to her if she has some more questions. Julie do you have additional question.
Julie: no

Both I and Julie: Thank you!

Interview III: with 01 District A01

Interviewer: I Participant: P, Observant: O

Interviewer: Good afternoon?

Participant: Praise to the Lord! I am fine.

Interviewer: Praise to God!

You were selected because your sector is one of the multi-sectorial nutrition programme member offices. The confidentiality of the collected data is respected; it is used only for this research. Questions may take 30 minutes; if you are not interested, you can interrupt at any level of question; however, your answer is very important for the study.

Do you agree to respond?

Participant: Yes

Interviewer: To O, he agrees, so we can proceed.

Thank you, Participant: For the opening of the question, tell us about your name, your position, and the district where you are working:

Participant:- Thank you! I am currently working at the 01 District A01 as a team leader and delegate to the head of office.

Interviewer: Tell us your age.

Participant: 32

Interviewer:-for about your marital status:

Participant: I am married.

Interviewer: What is your profession?

Participant: A natural resource management professional

Interviewer: Your job description

Participant: As I told you earlier, the natural resource management working process team leader

Interviewer: - How many years have you worked in this position?

Participant: For 15 years.

Interviewer: Could you tell us about nutritional problems in your district?

Participant: - Nutrition problems in our district could be categorized into two categories:

1. One problem is a shortage of food.
2. The second one is the quality of the food, which means the unavailability of a balanced diet in the district is another nutritional problem. This means the feeding habits of the food that is available in the area do not consider a balanced diet.

Interviewer: - You said that there are two types of nutritional problems in the district: shortage of food and feeding habits of an unbalanced diet. Could you add more about nutritional problems at the kebele level and in the distinguished area?

Participant: - Our district was a large district earlier, which was divided into three districts; currently, there are 14 kebeles, of which 13 were rural and one was urban. These 13 kebeles have similar ecology and weather conditions. This region has a similar nutritional problem. As I said early, in all kebeles of the district, there is both a shortage of food and the unavailability of a balanced diet. Generally, it is difficult to differentiate nutritional problems in specific kebeles. Even though there were both long-term and short-term Crop cultivations, the nutritional problem is similar throughout the 13 kebeles of the district. However, in the case of urban kebeles, even if there was a shortage of food, they tried to have a more balanced diet than rural kebeles because most of the educated population lives in urban areas.

Interviewer: - Do you ever hear about different sectors working on the nutritional system? Which means a multi-sectoral nutritional programme?

Participant: - A01 is one of the sectors that works on nutritional programmes. When we see other sectors working on this programme, like fish and animal resource offices, which also work on this programme, which is aimed at producing animal products like rooster, sheep, poultry, and others,

The district H01 also works on nutritional programme follow-up; all A01, H01, and Fish and animal resource offices work on nutritional programmes directly or indirectly. Particularly, the health office works on the quality of food.

Interviewer: - How do these sectors implement the policy, principles, and system of nutritional programmes?

Participant: Well, these sectors that work on multi-sectoral nutritional programmes have their own roles; when we see the system of implementation, which means the responsibility of availing food in both quantity and quality, it is agricultural office.

The health office also works on how society feeds foods; they emphasize feeding habits in quality, like a balanced diet or not, which means diet is for health, for repairing the body, in order to get energy, or for disease prevention. Follow-up is a health sector responsibility.

The Fish and Animal Resource Office works on availing animal products, which is very important for child development. This sector works collaboratively with other sectors too.

When we see about policy, I doubt to explain it. As mentioned above, these three sectors on availing food by both agriculture and the Fish and Animal Resource Office and the Health Office work on the quality of food based on its importance, like disease prevention, repair, health, or other purposes. These sectors also provide training for community-specific or collectively for the work force that participates in the nutritional programmes**.**

Interviewer: Well, you are explaining in a good way; you said earlier that it is good for children and pregnant mothers. Please tell us about previous nutritional problems as compared to our current status**.**

1. **Participant:** Here we can see in two ways: Currently, the availability of food is better; this is due to increased production. The children and pregnant mothers also get a better diet when compared to the past. This is because of the increased awareness in society of a

balanced diet. For your surprise in the household, the head of household struggles to feed his children as much as he can afford. This is better than the past feeding habit.

Whereas the availability of foods is noticeable, there is a shortage; this is due to inflation. However, the government is working to handle this problem by inviting non-governmental organizations that work on nutrition and implementing strategies to increase productivity. Generally, we can say:

1. There was increased availability of food when compared to the past; however, there is still a shortage of food due to different reasons, like increased population and inflation throughout the nation.

Interviewer: - Good! Could you tell us what your office's actions are on the nutritional programme?

Participant: - In this office, we mainly work on availing food. It is known that at the national or district level, agriculture is one sector that works on the availability of food. We are working to increase the availability of food by using different strategies like mobilizing communities, preventing soil degradation, preventing climate change through planting trees, and increasing production. Another mechanism was initiating the planting of edible fruits.

Commonly, in this district, Maize, Haricot beans, and false bananas are planted throughout the district. The Agricultural Office owned the production of these crops. Generally, this office works on providing food for the community, starting with selecting seeds for sowing and fertilizers.

Interviewer: - You explained in detail; could you tell us about the aim and Plan of the nutritional programme in your office?

Participant: The Aim is to empower society with food security.

At the beginning of the year, we begin by planning, which includes winter and summer.

During the summer season, it focuses mainly on Maize sowing, which includes availing selected seeds and fertilizers, and is followed by Haricot bean production.

In the case of the winter season, there is also its own plan, which is mainly focused on winter. Haricot bean sowing and Planting potatoes are major planning tasks performed in our office. During these activities, strategies are implemented in order to increase productivity, like preventing soil degradation, using irrigation, availing fertilizers, and selecting seeds.

Planting vegetables is also another task performed in this season. Planning includes farmers' water harvesting systems and preparing for the sunny season. These are very important preconditions in order to provide a balanced diet for children and pregnant mothers.

Interviewer: Who is the owner of the multi-sectoral nutritional programme?

Participant: The A01 takes the greatest dividend in order to fulfil the nutritional needs of society. In this sector, the others also play their roles, either directly or indirectly.

Interviewer: Who will direct the programme? What was the job description?

Participant: This food availability programme?

Interviewer: Yes, what is his or her job description?

Participant: Issues related to availing food this office. The plan prepared by agricultural office will be presented to all sectors of string committee and implemented in collaboration with other offices. The major work of the agricultural office is working with farmers in order to increase production.

Interviewer: - What are the challenges of working with other sectors in the programme?

Participant: When we were working with the other sectors, there was a problem, which was not an integration problem but a logistical problem we faced. Despite this problem, we were discussing and solving it. So, there is not that much of a problem working with different sectors.

Interviewer: - As you know, sectors under this programme must avail themselves during annual, Bi-annual, and quarterly planning, monitoring and evaluation, and meeting attendance. If any problems, please mention them.

Participant: Well, I tried to tell it, sometimes there was a problem with monitoring; this was due to a shortage of logistics and vehicles due to the distribution of resources that were owned by one district in the past to three districts recently. As you know, our work is outreach, which includes working with farmers; therefore, transportation is a big challenge for us. However, we were using different strategies to solve this problem, and we are trying to overcome it.

Interviewer: - What are the community challenges to implementing a multi-sectoral nutrition programme**?**

Participant: In the case of our district, even though there was awareness of the nutrition programme, there was a distribution of aid due to climate change. This increases dependency on aid in a segment of the population rather than hard work. There was also a problem with technology adoption.

Generally, we can say that there was a problem with awareness to some extent. There were no challenges other than the problems mentioned above to implement a multi-sectoral nutrition programme.

Interviewer: Did the community reject the idea presented to them?

Participant: No, as I tried to mention earlier, the district faces recurrent drought, which leads them to accept government Ideas to increase production.

Interviewer: - Could you tell us about the preparedness of the community?

Participant: related work, which includes community; they are ready to work except in some areas where there is dependency; they are asking for inputs to the work related to increasing production.

Generally, we can conclude that there is no problem with preparedness except the one mentioned above.

Interviewer: - Please, could you tell us how multi-sectoral nutrition programmes are managed and reported?

Participant: Each sector will prepare its own report based on the activities performed and evaluated at the sector level, and then a review meeting will be held to evaluate performance in one center with other stakeholders. Reports will be presented to its office, then the string committee, the region, and other stakeholders sequentially.

Interviewer: - Who do the agricultural offices report their reports to: the health office, the administrator of the district, or all sectors? I need more explanation.

Participant: - First of all, as mentioned earlier, all sectors will be integrated to make one job, then reports will be prepared in each sector and presented to all stakeholders in one center. A performance evaluation will be conducted, and feedback will be given to each sector based on their performance. After evaluation at the string committee level, each sector reports to the region based on their line of reporting.

Interviewer: - Could you say something about this programme in accordance with the budget?

Participant: - What do you mean by this programme?

Interviewer: Multi-sectoral nutrition programme in relation to the budget for each sector to conduct nutrition programmes.

Participant: Generally, budget is allocated according to duties given; in cases of utilization, it is based on the need for each sector. In our office, budget was allocated in order to perform issues performed by this sector.

Interviewer: Is the budget enough or not? Why? And how do money and asset administration look?

Participant: I could not say budget is enough; as mentioned earlier, for monitoring and evaluation, it needs budget. The supports provide funds when they have them, but most of the time we face a shortage of budget to accomplish even planned activities.

Finance administration issues depend on the strategy of the district to manage finances. A big problem is budget shortages, which force them to use specifically allocated budgets for nutritional programmes instead of other programmes.

Interviewer: - Could you say something about Human resources in order to implement a multi-sectoral nutrition programme?

Participant: There was no problem with human resources, but a shortage of Budget.

Interviewer: - How can the workforce on the multi-sectoral nutrition programme be improved?

Participant: - A good opportunity is the integration of professionals from different sectors, which enables them to share experiences. In addition to these, In order to capacitate the work force, we provided training and shared experience with other stakeholders.

Interviewer: Is there a chance of training and other inputs to increase work force performance?

Participant: The availability of training is not given due to a shortage of budget.

Interviewer: - Did a discussion workshop for this programme take place?

Participant: - Even if I am delegated, I couldn’t say a lot, but I know different meetings are held and the head of office attends meetings. I think it was held.

Interviewer: It was you who said it, you heard it: who attended the workshop?

Participant: This includes all sectors, health extension workers, kebeles agricultural officers, kebeles administrators, and sector heads at the district level.

Interviewer: - who arranged the work shop?

Participant: It was expected to be arranged by the agricultural office; however, due to a shortage of budget, it was arranged by begging different non-governmental organizations.

Interviewer: Do you mean the NGO was arranging a work shop?

Participant: No, I mean, NGOs were helping to conduct meetings by supporting budgets for the review meetings.

Interviewer: - What activities are done to create awareness in the community?

Participants: - This was done by using different mechanisms, such as development army structures at the kebeles level, different meetings, SAFTNET programmes, and even churches, to create awareness about nutritional programmes.

Interviewer: Is Either another programme working on nutrition in your office other than the multi-sectoral nutrition programme?

Participant: There was in the past, but I know some times in the form of Aid.

Interviewer: - What was their aim and plan?

Participant: Their plan is similar to this programme, which focuses on the provision of an improved food supply, and they have productive programmes. It was aimed at empowering the community to assure food security.

Interviewer: Well, anybody who is given responsibility to establish this programme?

Participant: The district takes this responsibility; next to this, our office also takes responsibility.

Interviewer: - What is their commitment to performing this?

Participant: - This could be measured based on their aim and plan, which focus on strategies to enable communities to assure food security. During the evaluation of their aim, we recognized that they are committed and motivated.

Interviewer: What is your short-term (operational) and long-term (strategic) plan related to the multi-sectoral nutrition programme?

Participant: The Operational plan is to create a community with no problems related to food and a head of household able to avail food to his or her parents.

The Strategic plan is to empower the community to secure nutritional security and increase in

Interviewer: What does it look like in terms of the commitment of society to implement this programme?

Participant: Drought is common in this area; therefore, the community is always ready to respond as needed, except for the problems mentioned earlier. Even if they are ready, there is a shortage of inputs for agricultural needs like fertilizer and selected seeds.

Interviewer: - Is there any issue that gives you ambition for this programme?

Participant: - Community readiness, sowing of seeds, and digging their farm on time are reasons to believe it could be fruitful.

Interviewer: - Could you tell us about the political support for this programme?

Participant: - There is a lot of support for achieving good results on this programme. It was witnessed by the Kebeles supporters of district cabinets' work on this in order to achieve food security. Higher administrators in the district were also following and evaluating this programme.

Interviewer: - What is your recommendation in order to change the strategy of multi-sectoral nutritional programme challenges at the district, regional, and national levels?

- **Participant:** Current integration is very important to have a good result.
- Disagreement-solving mechanisms must be established and solved on time.

- Strengthening integration among sectors,
- Working in collaboration with other programmes and supporting each other both financially and materially by presenting an aim and plan in order to have better achievement**.**
- **Interviewer: -** You discussed well; could you please add more recommendations in relation to different sectors?
- **Participant:** In different sectors, which are performing well but may have some weak sides, each must work collaboratively and strengthen their integration.
- Each sector must work hard at the sectorial level, and when combined, the result will be attractive.
- **Interviewer: If you have any other ideas, please**
- **Participants:** Integration is a must; for example, in our district, water is a big problem, and the water and irrigation offices must support us to achieve our plan.
- **Interviewer: "I** finished my question. Do you have any questions, Julie?
- **O: -** How could we specifically improve child and maternal nutrition?
- Tell us how to prevent stunting and improve the nutritional status of children and pregnant mothers.
- **Participants: -** Stunting occurs due to a lack of food and a balanced diet, so making them have a balanced diet improves their nutritional status. This could be gained by using simple mechanisms, for example, making houses have hens, cows, roosting sheep, and goats planting vegetables. In the case of water and mining, the office supports them to provide safe and clean water to the community. The support of stakeholders to provide enough food for the community at one point, from farm to mouth, may improve the nutritional status of children and pregnant mothers**.**
- **Interviewer: -** Thank you very much for your time.
- **Participant:** No matter
- **O; "**Amesegnalewu"

Interview IV: with 01's ANF
I: Interviewer **R: Respondent**

I: For this, you are selected deliberately since you are multi-sectorial nutrition program workers who work as a leader in the agricultural sector. The information that you provide will be used for this research purpose only and will be placed in a closed cabinet. Questionnaires may take not more than 30 minutes and for the questions that you may not want to respond to, you can skip them and if you have any issues that may lead you to interrupt; you can interrupt and restart it. But, we considered that the information that you provided will be valuable and useful.
Do you agree?

R: Yes,

I: He said you can proceed.

I: can you tell me about yours including your region and woreda?

R: I work in 01 Woreda in Sidama Region.

I: How old are you?

R: I am 32 years old

I: Marital status

R: Married

I: Profession

R: BSc in plant science,

I: Position

R: ANF

I: Work experience

R: 15 years

I: Tell me about the nutritional problem in your region, district, or locality.

Probe: Ask for the nature of the nutritional problem in the local context.

R: In our district, there are nutritional problems for two reasons: firstly, due to the scarcity or shortage of rainy fall. This means that our district depends only on rain-based farming due to a lack of water sources, both ground and underground, in order to cascade irrigational purposes, and indeed, our district is a low-land area that increases vulnerability to drought and suffers from nutritional problems in our district. Secondly, due to the increment of high population growth in the district, the decrement of farming land in addition to being low-land leads to deep-rooted nutritional problems in the woreda. Because of this, children under five, pregnant women, as well as lactating mothers mostly suffer from nutritional problems mainly due to a lack of knowledge and food insecurity.

I: Have you heard about the multi-sectorial nutrition programme?

Probe: If yes, would you say something about its policy, principles, and implementation?

R: Yes, I heard about the multi-sectorial nutritional programme in our woreda, which is collaborated by the steering and technical committees regardless of sector. Multi-sectoral, there is a nutritional programme that targets the prevention of stunting for those who have under five children and are suffering from nutritional problems, including pregnant and lactating women. As part of our agricultural duties, we avail vegetables, fruits, hybrid maize seeds, and fertilizers at the household level in order to tackle nutritional problems.

I: How does this problem trend look from the past to the present?

R: In the past, there was a knowledge gap about how to harvest and cascade farms technologically. But currently, we are empowering, creating awareness, and giving nutritional-based education to the community.

I: What are the nutrition-related activities of your office?

Probe: Would you say more about each nutrition-related activity in your office? List of activities? Its aim? Any plan?

R: Our office responsibility is creating integration and collaborating with safety net programmes in order to tackle nutritional programmes and also supporting non-governmental organizations like World Vision, especially pregnant and lactating women, as well as those suffering scarcity of economic resources at the household level. We also support public work issues that lead to nutritional problems regarding disasters like flooding and worms in cereals. These challenges are aimed at by our office in order to decrease nutritional problems. We are working on incrementing the economy at the house level in order to boost socio-economic status by buying hens and educating consumers about the consumption of eggs and their nutritional importance for pregnant, lactating mothers, and under-two-year-old children rather than selling them on the market and encouraging farmers to assign as a model for other

communities. Generally, our aim and plan are to tackle nutritional problems at the household level.

I: What are the roles and responsibilities of your office to achieve the implementation of multi-sectorial nutrition?

R: All stakeholder groups in a multi-sectorial nutritional programme have their own aims, which were collaborated on and integrated by our office and bonded by both the string and technical committees. We should choose those very poor communities and create coordination among all stakeholder groups procuring, distributing, and using nutritional supplies through an integrated logistics management system that may improve implementation regardless of sector, even at the household level. All work should be rewarded, evaluated, and monitored by time-based schedules in front of higher-level officials. To sum up, farming, harvesting, and availing food are our sector's responsibilities at the household level to tackle nutritional programmes.

I: Tell me the ownership of the programme.

Probe: Ask in detail about the coordinator of the programme and this body's responsibility.

R: All multi-sectorial nutritional programmes aimed at sectorial duties and

Multi-disciplinary professionals, led by Wenda, administer, make decisions, and overcome nutritional problems at the household level. By improving nutritional status at the community level. Integrating and collaborating to tackle nutritional problems without assigning individual tasks to generalize this issue, all multi-sectors for this programme are owned regardless of the different multi-sectorial aims for working on a single improvement of the nutritional programme at the house level.

I: Tell me the challenges of your office in relation to collaboration with other offices.

Tell me detail about the procedures of the programme, such as the annual plan, half-year plan, quarterly plan, achievement report, meeting schedule, monitoring, and evaluation-related commitments.

R: Our challenge is a shortage of economy and not achieving a strategic and operational plan at the district level due to a shortage of finance systems, which are the main challenges at the woreda level.

I: What challenges are there for the community to achieve a multi-sectorial nutrition programme?

The commitment of the community and for any resistance from the community.

R: In the community, there is a behavioral change problem to seek and the acceptance of the importance of a multi-sectorial nutritional programme. There have been challenges in adapting the vitality of the nutritional programme, and the community needs support from any aid organizations without any work.

I: Tell me how the structure of this programme is organized.

R: The Multi-sectorial nutrition programme has its own string committee, which has its own plan based on the sectors and aimed at the kebeles and collaborates with multi-sectorial nutritional programmes accordingly. Each sector prepares the plan and has it evaluated by the woreda-level multi-sectorial nutritional programme focal and in front of the woreda administration. Monthly reports are prepared under the low-to-high levels of responsibilities of the multi-sectorial programme accordingly.

I: Say something about the programme in relation to the budget.

R: The Multi-sectorial nutritional programme targets many aspects at the household level, but due to a scarcity of budget, we couldn’t improve the programme because of the targeted budget on a single agenda. To sum up, there is a huge scarcity of budget at the woreda and regional levels. And all multi-sectorial nutritional programme financial systems are governed by the plans of the Ministry of Finance of Ethiopia accordingly, without interruptions.

I: HR and other resource issues in your office?

R: Multi-sectorial nutritional programmes have enough skilled human resources; rather than a scarcity of budget, we have skilled manpower in our sector and a skilled administration body.

I: How did the professionals who work on a multi-sectorial nutrition programme capacitate?

R: Update the programme through training, deep-rooted capacity building, and awareness for the community and ownership of the programmes, and all stakeholders should be updated on time to build capacity for a multi-sectorial nutritional programme and assigning sectors and based on focal person responsibility to capacitate in order to access nutritional improvement at the community level based on knowledge-based given tasks at the woreda level.

I: Have you had the consultant workshop on this programme?

R: In order to change those weak trends at district level, workshops are not enough, even if we had twice the attendance at work shops in the Aleta Wendo and Yirgalem cities, respectively. We all, focals and worada administering bodies, participated in the work shops that were arranged by sidama regional states, but not enough due to a shortage of budget through the year.

I: How do you involve the community to create awareness?

R: We involved the community after creating awareness by using local language through the radio by their kebele, religion, and neighbor leaders and creating awareness and showing

demonstrations through the local languages by flask discs. And about how to feed a variety of food, especially to pregnant women, two children, and lactating women in the community.

I: What are the nutrition-related programmes other than multi-sectorial in your office?

R: As I discussed, before we avail hybrid maize seed and fertilizer for protecting both man-made and natural disasters like flooding and soil erosion and improving and boosting nutritional and economic status at the household level,

I: Is there a designated responsible body to coordinate the programme? If yes, how is she or he committed?

R: Our office has responsibility for availing and augmenting the food-based support issues accordingly at the workplace level and in the community as part of our daily work activities to tackle nutritional problems at the household level, and our office has committed to doing so at any time based on the given plan.

I: What are the strategic and operational plans of your office in the multi-sectorial nutrition programme?

R: As our office, we have a monthly, quarterly, quarter and annual plan after proposing the strategic plan in order to accomplish it in the coming five years. According to our office, we plan to cultivate short-term farms like maize, harcot beans, and cabbages, which are planned as operational but, like false bananas and coffee, are planned as strategic, creating different potential bottlenecks to achieve developmental goals for agriculture at the district level.

I: How is the community committed to supporting the activity plan of this programme?

R: For availing agricultural logistics, including hybrid maize seeds, fertilizers, and supporting nutritional programmes, the community is very committed to providing support in any aspect for the demonstration and improvement of the multi-sectorial nutritional programme.

I: Tell us about the presence of a promising work structure in this programme.

R: A Multi-sectorial nutritional programme is launched; the community is ready to practise such programmes and knows the importance of integration and collaboration in multi-sectorial nutritional programmes rather than giving to a single sector.

I: How is the political support for this programme?

R: In our district, political support is initiated and supported by the woreda administration body in order to accomplish the duties of multi-sectorial nutritional programmes.

I: What do you think of the recommended strategy to improve the implementation of a multi-sectorial nutrition programme in your district or region?

In my view, there are three recommendations: first, strengthening political encouragement and professional achievement, increasing budgets, and training in different aspects.

I: How do you think these strategies can improve the multi-sectorial nutrition programme?

R: Creating awareness for the community about the importance of a multi-sectorial nutritional programme, increasing the training and annual budget, and sustaining the nutritional programme at the household level.

Interview V: with 03's A01

Interviewer: - Good morning?

Participant: - good morning.

Interviewer: - You were selected because you are the head of an office that works on nutrition. The confidentiality of your response is respected and used for only this purpose. We expect your response to be attractive and helpful; the interview may take from 30-45 minutes if you are not good at responding to questions. You can interrupt in the middle of questions, but it is very important to us.

Is it voluntary to respond to questions?

Participant: - Yes

Interviewer: Your work place?

Participant: My work place is the 03.

Interviewer: Age?

Participant:- I am 35 years old

Interviewer: Marital status?

Participant: I am married.

Interviewer: What is your profession?

Participant: agricultural profession

Interviewer: - How long have you worked?

Participant: I worked 12 years as an officer and as head for five years, for a total of 17 years.

Interviewer: "Good, could you tell us about nutritional problems in your area and district?

Participant: Good, there is a nutritional problem in the district; most of the time, mothers and children are exposed to this problem more. There are 17 kebeles (rural and two urban), in which 175,000 people live. As I said earlier, in this population, the majority of children and mothers are exposed to this problem.

Interviewer: - Good, you said that the most exposed groups are mothers and children for nutritional problems; please would you tell me the problem in relation to the villages?

Participant: good**,** I said before, there are 19 kebeles. I can distinguish the kebeles that faced more problems: Hanja Goro, Sadama Chala, Hogowo Bongicha, Shelo Balela, and shelo ilaancho and others. In each kebele, when I say mothers, pregnant mothers and children who live in this area were more unprotected.

Interviewer: - Have you ever heard about multi-sectorial nutritional programs?

Participant: - Yes,

Interviewer: - If you heard, what are its policies and principles, and how will they be implemented?

Participant: - Well, there are a number of discussions held among the agricultural office, women's and children's affairs, health office, and other offices. Sometimes the discussion focuses on the food system; particularly, health office workers report that mothers are not feeding their children even the food that they have in hand. For example, one mother who has hens in her household sells the eggs, which are equipped with protein, and buys less nutritionally valued food. We were teaching them not to do so and to feed themselves and their children with whatever they had on

hand. These participants from different sectors raised during the meeting that the problem is a shortage of food and that they are not using available food accordingly.

Interviewer: Well, how was this put into action?

Participant: The way of implementation is done by different stakeholders; for example, starting from the agricultural office, agricultural agents at the kebeles level were teaching about the way of implementation of the nutrition programme; they also taught about the policy and the aim of the programme at each kebeles level.

Interviewer: - good, could you tell us the trend of nutritional problems in the past and present?

Participant: There is a big nutritional problem that is increasing from the past to the present. There is a reason for this: the conflict between the tribes started in 2011 E.C., and for this reason, four kebeles migrated from their living area. The other four kebeles were semi-migrated, which makes the farmers not cultivate timely on their farm.

Another problem is the absence of rain. For example, the month of March arrives, but there was no rain last year, causing the people to face a nutritional crisis.

Interviewer: What work is related to nutritional problems in your office?

Participant: In my office, in order to overcome this problem, we are working on false banana planting. Even though it takes a long time, false bananas cover the nutritional problem for a long time, based on our past experience. Next, we are teaching the farmers to grow maize, haricot beans, and potatoes by using selected seeds and different fertilizers. By doing these, we are working to increase productivity. In order to solve the current nutritional problem, we are creating awareness to plant potatoes and saw haricot beans, which may reach maturity within three months.

Interviewer: - You tell us in detail, but what is the aim and plan of your office on nutritional problems?

Participant: The goal is to increase product and productivity in order to prevent a food shortage in society**.** It is also aimed at making society secure its nutritional needs and providing food for the market.

Our plan is to empower society in relation to food security. This means sawing selected seeds, increasing the product and productivity. Generally, we planned to see a productive society.

Interviewer: What is your office's responsibility in a multi-sectoral nutritional programme?

Participant: In collaboration with other stakeholders, the office worker plays a great role in productivity. The structure is extended up to the farms of farmers at kebeles level, and it’s aimed at securing the food needs of society by providing professional support.

Interviewer: Who is the owner of a multi-sectoral nutritional programme?

Participant: The owner of this programme is mainly a health office; next is an agricultural and natural resource office. These two sectors have a lot of responsibility. I said that because agricultural and natural resource offices first work on food availability, if it is beyond this, the next step is owned by the health office in order to fight against the outcome of nutritional problems.

Interviewer: Who will manage this multi-sectoral nutritional programme and what is his or her job description?

Participant: in a programme managed by the health office, the job description is to demonstrate nutritional diversification. However, if it is for sawing, responsibility lies with the agricultural and Natural resource offices.

Interviewer: - What are the challenges while your office works with other offices in this multi-sectoral nutritional programme**?**

Participant: The challenge of collaboration with other offices is work overload; sometimes stakeholders are not available during meeting time. Unavailability of logistics like vehicles.

Interviewer: - could tell us more about challenges during planning, quarterly, biannual, and annual meetings, monitoring and evaluation, and meetings?

Participant: As said earlier, there was a problem during follow-up and support; during planning, the heads of some offices complained that they had no time.

Interviewer: What were the challenges for the community to implement a multi-sectoral nutrition programme?

Participant: There is not that much problem because society takes what we tell them; however, sometimes it seems that they are busy and do not attend meetings during awareness creation.

Interviewer: - Could you tell us how this programme was managed and how they reported their work?

Participant: There is a plan and reporting mechanism; during the meeting, there is a report presentation and it is reported to the higher and diagonal hierarchy.

Interviewer: - You said that the ownership belongs to the health office; for whom do you report, the administrator of the district, the health office, the region, or whom? Please elaborate more.

Participant: After discussion, the health office reported their line to the region.

Interviewer: From the health office to the regional health bureau, how about the agricultural office?

Participant: The health office is responsible for managing the programme; therefore, the agricultural office reports to the health office.

Interviewer:- Could you say some things related to the programme budget?

Participant: I am not aware of the amount of the budget; therefore, I do not know about the budget and could not give a response.

Interviewer: - Could you say the same thing about human resources for this programme?

Participant: - What do you mean?

Interviewer: I mean, the work force is recruited by the government, who assigned it for this programme; is it enough or not**?**

Participant: Well, there is a shortage of human resources. It is difficult to distribute in the 19 kebeles of the district.

Interviewer: - As I mentioned early, we are discussing a lot of challenges; now we proceed to opportunities. How do we make the multi-sectoral nutritional programme more productive and competent?

Participant: We make them competent by providing training and refreshment and availing some logistics for Kebeles support, which is not that much.

Interviewer: - Could you say more about the training opportunity, work shop, and sector work on this programme?

Participant: There are no chances.

Interviewer: Is is discussion workshop held? If held, who participated and who arranged it?

Participant: No one is requested to participate in the discussion workshop. No workshop was held at the district level.

Interviewer: anyone else participated in it?

Participant: I have no Information.

Interviewer: - How are you working to create awareness for the community**?**

Participant: We are working on awareness creation for the community; the administrator of the district manages the meeting, and after the discussion, each participant takes the responsibility to create awareness for each supporting kebele; for example, there were 12 mothers from two kebeles who got support this year. This means providing training for mothers, attending meetings at the

kebeles level and other conferences, and creating awareness through work done by health extension workers and officers from the agricultural and natural resources offices.

Interviewer: Does any other project work on nutritional programmes other than multi-sectoral nutritional programmes in your office?

Participant: There is no other programme. However, we are working to eliminate hunger in people unless they have a disability, which is our motto in order to increase productivity.

Interviewer: - Could you add more about this point? For example, I know the SAFTNET programme, aim, and plan.

Participant: As you said, there are SAFTNET beneficiaries on a monthly basis, and we are encouraging the beneficiaries to save some of their benefits through awareness creation. Because this SAFTNET programme is not continuing, it may be interrupted, unfortunately. Therefore, some households buy sheep and goats based on ecology. We are also teaching them not to depend on the aid. For the other aids, there was a selection criteria for each household, which was aimed at the head of the household who was not able to feed his family.

Interviewer: What is the aim and plan of these programmes working on the nutrition programme other than the multi-sectoral nutrition programme?

Participant: The plan of these programmes is to prevent human deaths due to food shortages, and their aim is to empower households to have sustainable food security. Generally, their aim is to see productive households**.**

Interviewer: For whom is the responsibility given for this multi-sectoral nutrition programme? If it is given, what is his responsibility?

Participant: The responsibility is given for different work processes; for this multi-sectoral nutrition programme, the higher responsible person is the administrator of the district.

Interviewer: - You are telling me, well, what is the commitment of the district administrator to this programme?

Participant: The district administrator is working day and night on this programme in order to help poor people. For example, during the wintertime wheat sawing around the River, he played a great role in empowering society for their food needs**.**

Interviewer: - Could you say the same things about the operational and strategic plan of your office in this multi-sectoral nutrition programme?

Participant: We have a ten- and five-year strategic plan, and our operational plan includes the rain season plan for five seeds: maize, haricot bean, potatoes, sugar potato, and false banana planting. For example, for false banana planting, digging the old one and planting the young one, in the case of maize sawing, digging, and providing fertilizers, and so on.

Interviewer: - Could you say the same things about the commitment of the community to support a multi-sectoral nutrition programme**?**

Participant: The commitment of the community is high beyond the problem that I raised earlier. This is because they see products at ground level; for example, with the help of the Sekota Declaration, we bought goats and hens and provided seeds of carrot, cabbage, and red root, which is why they are energetic to help the programme.

Interviewer: Could say the same things about the conditions that give ambition to these multi-sectoral nutrition programmes?

Participant: According to our office support at the kebeles level, while sawing seeds, it has its own way unless it could not be productive; therefore, professionals from sectors and the availability of selected seeds, fertilizers, and garden vegetables are enforcing factors of this programme.

Interviewer: What was the political support for this multi-sectoral nutritional programme?

Participant: There is a lot of political support for this multi-sectoral nutritional programme; during monitoring and evaluation and follow-up, they were working on solving problems related to this programme.

Interviewer: - What are your recommendations for the improvement of this multi-sectoral nutrition programme strategy?

Participant:-

- Regular supportive supervision of regional government since we are working for the community.
- Logistic support, special vehicles we are divided from Boricha district, and we have a shortage of vehicles.

Interviewer: What are your recommendations for sectors that are working in multi-sectoral nutrition programmes? If any

- The education office must follow up on school dropouts; if it is a nutritional problem, we could work to solve that problem.
- The water mining office must work on schools and households by availing clean and safe water.
- Health offices assign professionals to assess the nutritional status of pregnant mothers and children.
- Mothers also support each other by accepting packages arranged by the animal and fish resource office and feeding on animal products

Interviewer: - We said that nutritional problems affect pregnant mothers, children, and lactating mothers. What do you think about improving strategies in multi-sectoral nutrition programmes?

Participant: It needs a new strategy in order to improve the programme; for example, in the past, there was a follow-up problem. For this reason, increasing follow-up is one strategy. During meetings, active participation is encouraged.

Interviewer: - I am going to finish my question. Do you have any additional ideas?

Participant: I have no other idea.

Interviewer: - I have finished my questions. Do you have any questions?

Julie: I think you asked about pregnant mothers and children's nutrition.

Participant: - In our office, we are not working that much, but health office and health extension workers are working well. During antenatal care, health professionals identify the nutrition problem and attach it to the food aid system. We are supporting them.

Interviewer: - Thank you for your time!

observant: - "galateema"

Participant, thank you!

Interview VI: with 03's ANF

Interviewer (I) and Respondents (R)

I: Good morning?

R: Good morning. Welcome! Did you come safely? I: yes

I: You were chosen because you are a staff member on this programme, and your willingness to participate in the programme determines whether you participate or not. The surveys will take 30-45 minutes to complete, and you have the right to stop at any time. We consider the information that you provide valuable and useful for this research purpose and for the community. Information collected is used for this research purpose only, and data collected from you will not be accessed by others and will be put in a closed cabinet. Do you agree to participate? Participant…

R: Yes, I agree.

I: He agreed to participate, Julie: okay!

I: - Tell us your Woreda/region/region office name

R: - 03s ANF.

I:-Age

R:-31

I:-Marital status

R:-married

I:-Professions

R:-Horticultural science degree

I: - Position:-

R:-ANF

I: - Work experiences

R:-7 years (5 officer service and 2 position service year)

I:-Tell us the nutritional problem in your woreda?

R:- Although the onion, potatoes, "boloke," and sweet potatoes are the main foods consumed by our community, or Bilate zuria woreda people, not all households have access to these foods. Food scarcity as a result of climate change is common in this woreda. Food shortages were a major problem owing to climate change, particularly last year. Approximately 80% of people currently experience food shortages. Even though the government is supplying the lunch through World Vision and the amount of inset is decreasing because to climate change, dietary issues still exist in Bilate Zuria woreda.

I: - Tell us about nutritional problems at the level of kebeles/villages.

R: - Due to climate change, food is scarce in our villages. To purchase food, they sold their livestock. Our population struggles with poor nutrition. To raise awareness of these problems and the food shortage in villages, we are engaging with health experts. Food shortages for expectant and nursing mothers are also common, as is stunting. Food shortages also frequently cause children to miss developmental milestones; therefore we are attempting to raise awareness of the

importance of consuming a variety of foods from their environment. The previous time, with the aid of the Seqota program in the kebeles, Seqota declaration supervisors, the region by various sectors worked on the screening of malnourished pregnant women, and breastfeeding mothers of under 1 year old children performed and supported. Agricultural fruits were offered, including lambs, carrots, tomatoes, potatoes, and "the Protein-Quality Maiz" programme. We thought that as the kids ate from the better nutrition system, their developmental milestones would increase. We are educating the population about the importance of varied food intake because there are gaps in the availability of a balanced diet there. The lack of a balanced diet has an impact on our community and will limit or harm children's mental development, but if we receive support, this issue will be resolved. For instance, Mr. Tadesse's family's nutritional issues were resolved by delivering all packages, including goat, hen, "key-sir," carrots, and this Seqota declaration program's ongoing activities.

I:- Have you heard about the multi-sectorial nutrition program?

R:- Yes, I not only heard but also worked with them on nutrition.

Probe: About its policy, principles, and implementation?

R:- As a multi-sectoral nutrition program, we are working on preventing stunting in collaboration with the Seqota Declaration. To prevent stunting all sectors working on behalf of their concerned duties. For example, the agricultural office, water office, health office, education office, and women and children office, work together, and the main aim is to make stunting zero in children. To do this, not one sector but all sectors must collaborate. Activities performed mobilize the community, create awareness of stunting, and can prevent stunting at the kebele, woreda region, and national levels. As the water office works on the provision of clean water, the agriculture office provides fruits, vegetables, grains, and tubers, like potatoes, tomatoes, and protein-containing products. In collaboration with the Seqota declaration, screening malnourished pregnant women and feeding children for one thousand days to prevent stunting, otherwise, stunting can’t be prevented. Seqota declaration trains us on how and when to transplant, and we are working on our activities by our plan.

I: what about implementation?

R:-Implementation is good since the 08 programme from the region supports and follows this programme implementation. We are also working at an individual beneficiary level within Kebele. We see visible changes/improvements in awareness within the community through the 08 programme on nutrition. When we compare people who benefited from the SafeNet programme have no improvement but from the 08 programme, beneficiaries there are improvements. My request for the 08 is it is better to add the budget to include additional beneficiaries.

I:- Nutritional challenges in the past and present? What trends look like?

R:-Previuosly nutrition problem was severe due to awareness problem but now we are creating awareness on how they can get balanced diet, productive farm/yield increment method and there are improvement.

I: What are nutrition related activities in your office/agricultural office?

R:- As an agricultural office, to secure food for the community and use their product effectively. To make beneficiary farms throughout the year by using irrigation like cabbage, 'key-sir, carrots, and sweet potatoes, and working on 'Protien wise maize' but a shortage of its supply.

I:- What do you say about aim and plan of this agricultural office on nutrition activities?

R:- Our aim is to have food secured community. We have a plan, and our plan is to identify malnourished children from kebele and how to prevent this malnutrition. In addition to that, we planned on productivity (increment of product).

I:-What are the roles and responsibilities of your office to achieve the multi-sectorial of nutrition?

R:- 08 supporting us. Our responsibilities in this program are preparing plans, coordinating programs like the Seqota declaration, and working with our community to improve nutrition problems. Providing proper training, using technology to improve productivity and prevent nutritional problems.

Probe: what are the specific roles and responsibilities of the agricultural office?

R:- Providing agricultural products to farmers, and the first level for nutrition/food provision. The health office works on creating awareness, the water office focuses on clean water supply, the women and children office work on creating awareness, agriculture office focus on balanced diet provision for children and pregnant women.

I:- Who is Ownership of this program?

R:- Agriculture office.

I: - Who is coordinator of the program and his responsibility?

R: - Coordinator is nutritional focal person. Responsibility is creating plan, creating awareness for community.

I: What are the challenges for your office to collaborate with other sectors?

R:- The reporting system which means health office to health bureau, agriculture office to agriculture bureau, and budget allocation to each sector for the same activities may affect the program/challenges collaboration.

I: Challenges related to the annual plan, half year plan, quarterly plan, performance report, meeting relate, and monitoring and evaluation related challenges.

R: - Yes, we have challenges. On presenting the report, we have problems/challenges in reviewing our performance report in cooperation with different sectors. The health office is the main lead sector for this issue and the health office does not coordinate the sectors for reviewing our performance report, due to another workload for the health office, and the leader of the agriculture office. If the nutrition program self-stands for sectors, it can review its program performance properly. We provided a three-month performance report, and six months report, but gaps in meeting to review our performance. The program is not coordinated properly, the budget is not coordinated properly, budget is not at one place and may challenge to coordinate and control properly.

I:- What challenges are there for the community to achieve a multi-sectorial nutrition program?

R:- Poverty is one challenge for the community. For one kebele we identified a problem example at ‘Konsore Hanja kebele’. We provided hen, goat, fruit, and vegetables in Seqota Declaration program but, households have no meal/food at home and they prefer to sell goat and hen to buy meal/food for that time. The community can’t stay for time to get food since the shortage of food is urgent for them and they want ready-made food.

I: Is there any problem related to community resistance?

R:- No resistance from community. We screen malnourished children by technical working group, and identify kebeles mostly vulnerable.

I:- How the structure of this program is organized?

R:- The Seqota declaration structure is well organized and can we say it best program, but the challenge is the financial system because fund disbursement is on governmental bank accounts not for separate bank accounts. So, when we want money for the program, we can’t get it because they pay money for salary and other activities.

I: Tell me the reporting system of the program

R:- Reporting is based on per quarter, Monthly, bi-annually, and yearly. We have focal persons for kebele and compile their performance and reported monthly base till 26. Woreda also compiled reports from kebeles and reviewed them to report to the region. The Woreda coordinator also collects reports from sectors and reports to the Seqota Declaration delivery unit. Routine activity performance is directly reported in line with sectors to region but the Seqota Declaration program is directly reported to the Seqota Declaration delivery unit from Woreda.

I;- tell us about the program the budget, financial administration , budget adequacy..

R:- We have a budget shortage, and a transportation system to support mothers. This time is a technology and need smart phone and tablet to capture data/information but due to budget shortage, we can’t get this material.

I: How are you utilizing/managing this scarce budget?

R:- The budget is allocated mainly for three purposes, first for creating awareness for beneficiaries on how to feed and when to farm, second for procurement of materials, and third for transportation purpose.

I:- Tell us HR in your office.

R:- Human resource is sufficient within their office.

I:- How can we capacitate the professionals on multi-sectorial nutrition programme?

R:- Sufficient budget allocation, supportive supervision, proper training provision, and inclusion of all concerned bodies and providing proper data capturing materials like tablets.

I: Is there opportunities for training and workshop?

R:-Yes, at kebele level there is opportunities for training, and workshop planned for future.

I:-Have you had the consultant workshop on this programme?

R:- Planned.

I:- How you involve the community to create awareness?

R:- Six participants participated/awareness was created from each kebele. Those aware community members create awareness at Church, and also we created ‘Community lab innovation’ within the community.

I:- What are the nutrition related programmes other than multi-sectorial of your office?

R:- World vision programme Probe: Its aim? R:- Provide daily food to prevent nutritional problem.

I: Is there designated responsible body to coordinate the programme?

R:- On behalf of agricultural nutrition program and well committed, but no single person designated.

I:- Do you have strategic and operational plans of your office in multi-sectorial nutrition program?

R:-Yes we have. For example:- as agriculture office short term plan three months like ‘boloke’, potatoe, ‘key-sir’ medium term plan five months, six months, like maize.

I:- What look like the community commitment to support Seqota Declaration activity plan?

R:- Our community commitment is good. Training provided through this program most of the time cascaded.

I:-Tell us the promising work structure of this program

R:- Seqota Declaration program prevent nutritional problem.

I:-How is the political support on multi-sectorial program?

R:-Political support on this program is medium.

I:- What do you recommended strategy to improve the multi-sectorial nutrition program implementation in your woreda/region?

R:- The region should take procurement issues. Reviewed and cleared activities for implementation with timetable, follow up from region, and provided direction while the finance system delayed our request, especially procurement issues. Other printer provisions, at least, bureau, sufficient budget allocation, capacity building training provision since the update is mandatory

I:- Recommendation for other sectors?

R:- Let us work together for all sectors, timely meeting with each other.

I:- What kinds of strategies can improve the multi-sectorial nutrition program?

R:- Seqota Declaration program support with sufficient budget, training for professional and supportive supervision will bring improvement.

I:-I have finished my question and if you have any concerns you may add.

R:- Yes, I have. Here in Kebele, we have many model farmers, and for the Seqota Declaration program those model farmers also it is better to include them in training, because even if they have enough food, they can’t feed a balanced diet due to knowledge gap. So, through this training, we can solve the nutrition problem.

Interview VII: Interview with 04 district A01

Interviewer: I

Translator and transcriber : A

Interview setting : A01

office interview duration ; 1 hour

i: interviewer

r: respondent

The objective of the study is to evaluate the challenge and opportunity of mutisectoal collaboration in Southern Ethiopia, 2015 EC. In addition it is to identify strategies to improve the multi sectoral program. You are selected due to you are the office head of one of the multi sectoral office. The information you give us is confidential. And the information is very helpful for our study. The interview will take around 30 minutes. I f there is any attempt to interrupt an interview you can leave our interview with out any risk.

I : so he has agreed for the interview we can proceed to our questions

Socio demographic data

Working place : 04

Age 42

Marital status: married

Educational background : Bsc in agricultural economics and masters degree in economics

Year of work experience : 23 year

Current position : A01

I: what are nutriotional related program in your district ?

R: because of the implementation of multisectoral program(sekota program) it consists six sectors. They are working a very wide range of work in collaboration. Last year it have assessed

for their achievement and this district is not well known in nutrition related activities before several year . but since the implementation of this program

I : your response is good but my focus is basically to identify what are the challenges of nutrition program in your locality ?

R: education were given from the health and from agricultural office also with coordination of focal persons > the identified challenges are there is habit of consuming only one or two kind of food item even though there is little change now a days the community is highly dependent on a single crops like maize . for example we tried to demonstrate with cereals and fruits but there is no habit of consuming fruits that is one the challenge. Lack of awareness to include nutrient like protein, carbohydrate The other challenge is in adequacy of rainfall to harvest enough amount of crops

I : have you heard about a multi sectoral collaboration program ?

I : if yes what are the principles, core activities and policies of the program ?

R: all the six sectors are performing a good job, there is a focal person for sekota program in our office even though I do not have access to participate in the training as he has informed us they have acquired good knowledge regarding the aim of sekota , discussion with concerning bodies . there is also demonstraton session of nutrition practice to the community, it is huge benefit for the society to create awareness. Last year we have worked among six areas but this year we selected only fourteen people from two kebeles we have been working on them. There is change in awareness among the community

I :very good your responses are in line with my question , you have briefly answered the way of implementation of sekota program. Would you please explain more on how they are working on pregnant and children?

R : well, regarding pregnant women and children there is education session for mothers about 1000 day feeding system, feeding system, benefit of 1000 day. A few days before we have provided training for them on the 1000 day they have raised many questions we tried to brief them well, they get a very good knowledge.

I : what is the current status of nutrition problem as compared to previous years ?

R: there was no nutrition problem in the previous years, the community can feed themselves very well. now adays the resources are somehow limited . and there is a bad habit of selling all the products from farming and also from animal production. They sell butter they did not consume for themselves and feed their children. Though we have reached by education only limited number of people but we expect they will teach their surrounding community and can bring change. And we witnessed there is an improvement in the utilization of their products since we started educating them,

I : what are the activities related with multisectoral nutrition program ?

R : the first one is we have purchased seeds of fruit and vegetables then we give them training how to cultivate in their farm land on the selected kebeles for women. Last year we have planned to implement this in selected four kebeles, as an agriculture sector, our office has planned to distribute seeds of maize which enriched with protein , barely, carrot, cabbage and the like. The fish animal production sectors on its part has also distributed different animal product like chicken.

I : very good, would you please tell me the plan of the this multi sectoral program ?

R : first we planned to bring change in our locality then in the whole district and at county level. To change the nutrition status children and mothers .In addition to the activities of other sectors our sector has also taken its part to enhance the experiences and go forward with shared experiences

I : your answers is very satisfying, what is the responsibility of this office in implementing multisectoral nutrition program ?

R: the responsibility of our office is firstly, we agreed on the aim, training the concerning bodies, cascading it at the lower level a health professionals then we expect final result. Particularly creating awareness and assisting the community to get a high benefit

I : well, who is the responsible person from this office for multi sectoral program?

R : according to our sector we have one focal person for sekota program, and from those six sectors each of them have one focal person. Totally six person overall the responsible sector is

a health office beyond that there is focal person assigned at the woreda level to facilitate the overall activities

I : who is the responsible person overall

R: it is the health office that coordinates the multisectoral program

I: who is the facilitator of the program?

R as I have said a focal person at woreda level will communicate with the region, get information and share to those six sectors.

I : good, what is the responsibility of this assigned person at woreda level ?

R : he assign tasks for the responsible sectors like finance, fish and animal production office, water and sanitation office accordingly to the respective focal persons. He is responsible to summarize all the activities from the sector offices

I: what are the challenges to implement the program for your office

R: there is no that much challenge but we face problem with finance office because all the sector will request for finances for their plan to access their support on time to purchasing a materials . except that there is no challenge with other sector.

I: is there any challenge related with annual, six month and quarter plan or related with monitoring and evaluation ?

R : there is annual and also quarter plan for each sector offices , in our sector office there will be seasonal and urgent task which may result delay in nutritional activity. For example we need to distribute the seeds for the community it was happened in the last week. But we try to came back to the routine activities as soon as we completed such urgent tasks. Regarding other sectors there also may be same scenario due to completing urgent tasks. Otherwise we did not faced a challenge regarding meeting, reporting issues.

I: what are the challenges at community level to implement multisectoral program ?

I : commitment from the community, resistance to implement

R: regarding this program there is no big challenges from the community. Sometimes one of the common challenge is there is complain form some people for not get selected for this program. Due to or selection of neighbor and when they get excluded there are minor problem related with this, and we try to explain them

I: what is the structure of multisectoral program ?

R: the way of structure has planned to meet the standard of this district based on the requirement, plan is cascaded from the region level then meetings are arranged at the district meeting hall with the concerned bodies they evaluate and give comments. Then share task to implement at the community level

I: could you please tell me the reporting system of this program

R: professional will compile report and submit to the focal person. Then it will be evaluated with extension workers and sector health professionals. There will be meeting at the health office with respective focal persons then submit to the woreda level focal person

I: could you please say something regarding budget?

R: there is no increment in the allocated budget from previous year almost the same . it has allocated 408,900 ETB . it is inadequate because from the total budget we spent around 246,000 ETB only to purchase seeds for the community and get shortage or other activities. The financial system is also not convenient for us to directly take action accordingly.

I: how about human and other resource utilization?

R: we prepare proposal for the finance but we do not get answer with in short time, it is highly challenging. Among the focal persons that I have mentioned one is from financial sector he get access for training and he tried to facilitate the financial issue at the lower level. However still there are challenges to solve financial issue problem at the lower level

I: say something about human resource for multisectoral program ?

R: we do not have challenges regarding a human resource, as is said before the problem is to access budget on time to implement the activities, when we plan to assign responsible bodies for those task we did not face any challenges related with human resource

I: very good, could you please tell me about the opportunities of mutisectoral program ?

R: the first opportunities is training the responsible person from each sectors about the program , not only that but also monitoring and evaluating to make sure that the activities activities are in line with the training. Monitoring mechanism of their reporting system is also can be mentioned as an opportunity. There are a wide range of opportunities created and also it needs strong monitoring and evaluation mechanism

I : is there an opportunities for training , workshop at any level either it is at woreda level or at kebele? Please say something about this ?

R : YES, there are opportunities both for training and workshop either at woreda or at the regional level. Last year there was training on this program at a place called “kuyara galalcha” we did many activities. We can say there is wide access for training, but the challenges is on the implementation at the lower or community level

I: is there opportunity for consultative workshop? Is yes who are the participant

R: steering committee from the woreda has participated on this consultative workshop, technical team , focal persons and different responsible person from the woreda had also participated on the workshop.

I: who are members of the steering and technical committee?

R: steering committee include the woreda administrator, consists five woreda administrator and the technical team are focal person form the six sectors

I: who prepared the workshop

R: the workshop was facilitated form the region level in collaboration with the woreda

I: good, what are the activities for create awareness to the community ?

R: we have a good progress on awareness to the community , the health extension works with the kebele leaders and head of sector office in collaboration they get knowledge

I: is there any other nutrition related activities in this office other than multi sectoral program ?

R: there is one program under the extension communication and it has been functioning simultaneously with the sekota program dietary practice , demonstration session for mothers, how to fee their children. There is also safety net program but it is not under this sector

I: Aim and plan of this program ?

R: the aim is to improve the mothers there financial income , previously women were highly dependent on their husband now our plan is to improve their living standard , to get better income , make able to satisfy basic need to their children

I : who is responsible for the facilitation of the program , how about his/ her commitment ?

R: because of the newly launched program in addition to the routine activities , there was some inconveniences at the beginning, but we get familiar to the program there is no such problem. The assigned person is also highly committed to his responsibility. He had been collecting the necessary informations timely and share to the public. We do not have challenges on this dimension

I: what are the strategic and operational plan of this program?

R: the strategic plan is planned from three up to five years . we put in percentage while planning and to evaluate the achievement. Regarding the operational plan we evaluate quarterly and with in six month.

I : how about the commitment of the community to support this program ?

R: the community is highly commuted and supportive for this program. They want to be scaled up and to get access for the training

I: What are the promising actions for the program?

R: there is an increased access to get material support for the community like water pump and other

I: do have any recommendation?

R: I recommend to the sectors to collaborate more, to have meeting frequently and evaluating properly. To facilitate more trainings for women on how to become more productive, dietary habit I : thank you I have finished my question

Interview VIII: Interview with ANF of 04 district

Interviewer: The objective of the study is to assess challenges and opportunities in Multisectoral Nutrition Programs in rural Ethiopia in 2015 E.C. In addition, it is aimed to improve the Multisectoral Nutrition Program by creating/ looking for new nutrition strategies. You are selected as a study participant because you have been working as an expert in rural Multisectoral Nutrition Programs in different sectors. The information that you give is very important for the study. I interview you for 30 minutes. If you are not ok with interview, you can with in between. Do you agree?

Interviewee: Yes, I agree! And you can start the interview.

Interviewer: What is your name?

Interviewee: My name is Y.

Interviewer: What is responsibility?

Interviewee: I'm head of Farming Department under the office of Agriculture and Natural Resource Development in 04 Woreda

Interviewer: What is your age?

Interviewee: 32

Interviewer: What is your marital status?

Interviewer: Married

Interviewer: What is your profession?

Interviewee: I'm agricultural farming professional

Interviewer: How much is your work experience?

Interviewee: 16 years

Interviewer: What are challenges related with food/nutrition in your woreda/locality?

Interviewee: We can say the problems/challenges of food is a very big problem in our locality/woreda. These food problems/challenges are happening due to varies reasons; the first

reasons for food problems is rapid population growth rate. Rapid population growth rate is causing fragmentation of farming lands (farming land has become insufficient each household), cutting forests for farming lands (deforestation), and environmental degradation. Due to these effects, the fertility of soil has become decreasing, the rainfall has become reduced, the climate has become changing, due to climate change unusual worms and diseases are damaging the crops. These all factors add up to result in a great reduction of agricultural food production and alarming food shortage in our locality/woreda.

Interviewer: Well! you have explained the reasons/challenges of food very well. Would you explain challenges/problems in your locality/woreda? In addition, would you tell me food/nutrition problems in pregnant women and children?

Interviewee: As I told you before, todays, even though the size of farming land has become significantly reduced, people farm and plant the crops on time. However, there is shortage of rain, especially since 2007 E.C, the amount of rain has reduced and the climate is totally changed in our locality/woreda. Ethiopia in particular and Africa in general has been facing climate change and reduced rain. Our locality/woreda is semi-arid (Qolla) where climate change and reduced rain affects most. Rain does not come on time, if rain comes it stays for short time, it ceases before crops ripens and consequently crop reduction. Because of this reason the production of vegetables, fruits and cereals has become declining year to year. Children, pregnant and lactating/nursing women do not get enough food as they need. It is obvious that this in turn affects future generation. If pregnant and lactating/nursing women don't have a diversified food or shortage of food, the children will show static or slow growth because they get nutrients from their mothers. Therefore, there is a great problem regarding nutrition of children, pregnant and lactating/nursing women in our locality/woreda. Population size is another challenge in our locality/woreda. People of our locality do not move or migrate to other places to reside there. Therefore, it is a big challenge in our locality.

Interviewer: Have you ever heard about different sectors work in collaboration on nutrition program?

Interviewee: Yes, I heard and I can explain what I know.

Interviewee: If you heard, would you briefly explain about policies, strategies, and implementation and how activities of nutrition program are going in your locality?

Interviewee: If we talk about the nutrition program, Seqota Declaration since 2014 G.C has been implementing in our woreda/district. Seven sectors have been working jointly in our woreda/district to achieve goals of the Seqota Declaration. From FDRE Ministry of Health to our woreda along the different governmental hierarchy, different sectors are jointly working in collaboration. The joint work of the sectors identified the gaps in children, pregnant women and nursing mothers. Since the Seqota Declaration, special emphasis is given to improve nutrition of the children, pregnant women and nursing mothers. Seqota Declaration aimed to eradicate child malnutrition nationally by 2030 G.C. In consistent with national objective different sectors of our woreda are working in collaboration to eradicate child malnutrition by 2030 G.C. We are aiming to make child malnutrition zero by 2030 G.C in our locality. One sector cannot achieve this. It needs a multisectoral collaboration. Our all sectors working in conjugation to eradicate th child malnutrition by 2030 G.C. The sectors are fulfilling their responsibilities. When doing these activities, our woreda administration and sectors fully considered it as direction of the government program and hence given special attention. Our ultimate goal is to create better future generations that are very creative rather than expecting something created from the other, to create generations that find solution for the problem by themselves. Producing creative generation with wide understanding is very important for the development of the country in general. The second is a future generation should have normal and healthy body growth that is a very important for the productivity of individuals themselves and for the development of the nation also. And our ultimate goal is to create generation that are talented and competent nationally and internationally. For now, I can explain this much and if you want more I can explain.

Interviewer: Very good! Of course, you cannot explain all, if you understand the questions I ask and explain accordingly, it is ok. What looks like food challenges from the past to present?

Interviewee: In the past it was better. Because, soil was very fertile and there was the better crops production. As I told you before, the population size was small before, the number of populations has become increasing, the family size has become increasing. However, the size of farming lands remains the same. The land is not giving crops in amount it was previously giving due to different environmental and man-made factors like climate change, crop worms and diseases, reduced soil

fertility, shortage of rains, etc affecting crops and hence the crop production has become decreased. Our farmers are not obtaining enough improved seedlings and fertilizers because their price is beyond their income and they cannot afford. Previously they were used to purchase fertilizer with 500/600 birr. Currently, the same amount of fertilizers costs over 2200 birr. This price is beyond they can afford. They do not cultivate all of their farming land because they are bother of fertilizer cost. They cannot afford for the all the packages that farming professionals tell to do. This also reduces the crop production. The awareness of our people on food/nutrition in previous and in currently is verry different, currently our people have better awareness. Before animal farming was very better, now the cattle have become reduced because reduced grazing land due to population size growth. Although currently our people have better awareness regarding nutrition, they sell better products that they produced and use poor quality products for themselves. Generally, past food production better than the recent.

Interviewer: It is very nice! As I told you the objective of the study, from you, we obtain information about challenges, opportunities and recommendations on nutrition program. Now I ask you about problems on belongingness of the nutrition program. What does your office work regarding food/nutrition?

Interviewee: This office has been working on food/nutrition according to Seqota Declaration. We are working in conjugation with seven sectors. We are working with CEFTNET to provide food for hunger, very poor and during emergency. We collect information about the status of the people and transfer that information to the responsible authority and food and other aids will be provided to those who wants special food aids. Seqota Declaration focuses on nutrition of child (<2 years), pregnant and nursing mothers. The activities that our offices is undergoing are as I mentioned.

Interviewer: You nicely presented very briefly! What is aim/plan that your office is working on food related challenges?

Interviewee: The first aim of our office based on Seqota Declaration, identifying individuals or families that have food problems. The second aim of our office is creating awareness with in our community how to produce crops on their farm in the way they produce sufficient food from their garden. We advise people to plant grow variety type of crops to have diversified food from their own garden without purchasing food. We train our people how to plant and grow the crops, produce quality crops free of disease, and about the packages that they should fulfill to make plants

contain desired nutrients. While doing this tell our people that different inputs are needed like water and we tell them how to obtain water. We also tell them about soil treatments to produce crop containing desired nutrients, and tell them all processes from planting up to harvesting. Our first target is enabling our people sustainably produce quality crops from year to year. The second ensure food self-reliance of our people from their garden, by themselves. This solves food problems our people. It is expected from us to empower our people through awareness creation to improve their nutrition and health of their children. This enables to have better and healthier families that improve their income. It is expected from us to enable people to improve the crop production, to produce diversified food (based on nutrients) from their own garden and to live better life. We are training people to plants crops that have diversified nutrients contents in their garden, like protein, fat, carbohydrates and vitamins. This enables family to have children with good brain development and good mental understanding and creativity.

Interviewer: to achieve multi-sectorial collaboration, what is responsibility and efforts of your office?

Interviewee: As I briefly explained before, my office is working in collaboration with other sectors based on 'Secota' Deceleration.

Interviewer: To implement multi-sectorial collaboration on nutrition program, what is responsibility of your office?

Interviewee: My office provides different inputs for agriculture like fertilizers, improved seeds, water provision, farming and harvesting techniques, trainings on crop preservation, crop processing, etc. If there are no these inputs, crop will be reduced. We follow all step on process of farming from planting to consumption. Another role of our office is sharing, exchanging, and reach at consensus on ideas among the sectors. The works within sectors is highly interrelated and interdependent, hence need conjugation and collaboration. For example, inputs for our office for agricultural development is supplied by another office. Other office supply technique of food processing, cooking, hygiene, etc. Our primary work is enhancing farm production and farmers collect all food they need from their own garden.

Interviewer: A multi-sector-al nutritional program belongs to whom?

Interviewee: In our woreda health offices lead multisectoral nutritional program, other sectors share responsibilities. In office took multisectoral nutritional program as one of the major activities. Other sectors also do the same. All sectors share the responsibilities. But woreda health office lead the program.

Interviewer: Well! Coordinates program? What is his responsibility?

Interviewee: The coordinator is experienced. He is a leader of the technical group of the program. He is leading the program. He is a coordinator since 2014 G.C.

Interviewer: How much is his determination toward the program implementation?

Interviewee: In my opinion and perspective he is good, very good in motivating staffs, identifying the weaknesses work on it, and dissemination of information both face to face and via calling. For me, he is determined toward the program implementation.

Interviewer: Very good! You are telling me good information. What are challenges that your office faces in multi-sectorial nutrition implementation? Relate your explanation with, yearly, half-yearly, quarterly, final achievement report, evaluating and motoring of the program implementation. Would briefly tell me challenges of sector working together in collaboration?

Interviewee: There is no other problem, delayed finance process is major problem preventing achievement of activities on planed time. Some inputs are purchased and brought timely. Since 2014 G.C, I'm together. In both last year and this year delay due to financial process is major challenge. Due to this challenge, we could not finish our activities on planed time. It is very rare facing challenges from other sectors during the program implementation. Another challenge is budget of the program is always not released at the beginning of the budget year; it is always released in mid-year of the budget. It is very difficult to run earlier activities of the year. Our farming work is seasonal, activities of the farming depend on the season. We prepare annual plan based on the season. For example, during dry season, we plan for irrigation, if planned money for irrigation is not released on time, there will be cultivation in dry season. If money is released on time, all activities accomplished according to its time table.

Interviewer: Good! Is there no challenges in joint session of sectors, evaluating and monitoring programs?

Interviewee: There are problems. Sometimes we plan for meeting and the appointment are cancelled out. The chairperson of sting committee is woreda chief administrator and chairperson of technical committee is head person of health sector. Before I came this office, there was another head, I was not here since the beginning of the program. Since I came, postponing the meeting is a challenge. But one who is a coordinator is always contacts me and he properly follows, evaluates, monitors and gives direction. Since I came this of monitoring and evaluation is very good.

Interviewer: Up to now it is ok! Continue explaining the questions as you did before. During the implementation of multi-sectorial nutrition program, what are challenges from community? Is there unacceptance of the project from the community?

Interviewee: Till now we are working with people that have different problems. They listen and accept, but they have low knowledge and awareness. Due to this, they may not properly practice. Last year we have identified this problem and this year we are working with experts to improve their knowledge, awareness and practice. This problem is major problem and not solved yet and it need a lot of work. There is no problem from the community other than this challenge. We usually meet with a people and understand level of their awareness based on the ideas they rise. We farming professionals considered that low level of their awareness indicates our poor performance to raise awareness of the community. I don't see other challenges from the community. If we all professionals with other sectorial stakeholders implement properly, we can raise awareness of the community.

Interviewer: Would you explain how coordinated? For example, how activities are reported?

Interviewee: The report of this year begins from the April and plan is prepared. In the next three months plan will be implemented and people will be beneficiary. This program has a budget. This budget is breakdown for the accomplishment of each activity. When report prepared, its beginning is from the low-level managers (from farming professionals). There are report formats that are filled by the farming professionals. They report each activity monthly what is done and what is not done. The monthly reports from each kebele show what was the plan? And what was achievement? Then in woreda level, we collect the reports from each kebele and we give to technical committee. Then technical committee transfer report to the next level governmental hierarchy. If achievement is low in particular kebele, we assess factors, identify the gaps and challenges work on to raise.

Interviewer: Well! Before you raised challenges related with finance. I want to rise one question. How finance of the project is governed? Is budget is sufficient or not? You can raise more.

Interviewee: Good! When we compare nutrition problems of our woreda with an allocated budget, the budget is too less to address all the problems. We have 25 kebeles in our woreda. We cannot address all nutrition problems of all kebeles. There are selected kebeles based on priority. Some people in this kebele have become beneficiary of the multi-sectorial nutrition program. We help these people not to support for their life long, but to work to improve themselves to become self-sufficient, then we shift help to others. We do not want to make people rely on food support, but to become self-sufficient, to improve their income and motivate to lead healthy life. While supporting, we motivate people to look for another way to generate income and to produce food. I carefully compare multi-sectorial nutrition program with another programs. Unlike other program, multi-sectorial nutrition program details budget for each activity, and you cannot use the budget of one activity for another activity. If particular activity is not performed due lack of material or other preconditions, its budget is discussed by the technical committee, then technical committee transfer to string committee for the decision. Otherwise, if you use another purpose or activity, you we will be held accountable. Therefore, responsible body of each sector work carefully regarding the budget of the program. In this program activities are on ground within people, activities and budget are directly involves beneficiary. The budget utilization is very careful. Also, the activities are containing of details of to whom the budget is allocated, why is the budget allocated, and how is the budget utilized. I can say confidently that the staffs are very careful and have good attitude toward the budget, they are well minded that to whom this budget is allocated. Last year we successfully provided allocated budget to the people, but there may be some gaps among the staffs in the manner of the budget utilization. Detail budget breakdown directly address the people and it should be continued. Therefore, in this way of budgeting, the program will be a fruitful and can be achieved what aimed for.

Interviewer: It is very good! Would you explain about the human resource management?

Interviewee: Regarding human resource we see human resource as whole as woreda, we focus not only on staffs running the program, but we widen involve all farming professional staffs in a whole woreda. We give direction, training, and consideration for the all staffs in the woreda. Last year, our special support of the multi-sectorial nutrition program was implemented in four kebeles;

this year, multi-sectorial nutrition program has been implementing in two kebeles. But awareness raising and farming intervention involves all 25 kebeles in the woreda by mobilizing farming professionals. The program budget considers to trains professionals only those two kebeles prioritized for food support, but we our office looks for finance sources to train farming professionals of the rest kebeles. Moreover, our farming professionals focus creating awareness on child, pregnant and nursing mother nutrition teaching how to obtain diversified food from their garden by themselves. Human resources in all kebeles of the woreda are equipped with trainings and farming professionals are regularly carrying out activities in accordance with a given direction to raise awareness and improve farm productivity in the woreda.

Interviewer: It is very good that you said we would work on all the kebeles. Now, I ask you about opportunities to implement multi-sectorial collaboration on nutrition program. How to equip the personnel that work in multi-sectorial collaboration on nutrition program?

Interviewee: First, we work to induce objective and purpose of the program among our professionals. In our woreda, we have a total of 21,756 farmers. I represent all these farmers, I make them beneficiary through my professions, I give my skill, idea, advices, supports, training, etc. I motivate staffs that these problems are due to our people lack awareness and if we change awareness of the people will be changed. First, we change mindset of the staffs and we enhance their skill through several trainings. Before we work on the people first, we work on the understanding of our farming professionals. We make them to understand that we have obligation to face problems of our people and improve life of the current and next generations. Then we make them fully engage to work in the people to achieve objectives.

Interviewer: I'm asking you to talk more about the opportunities rather than explaining about the performed activities.

Interviewee: The first opportunity is availability of training both in our office and in woreda as well. Health sector and regional government are also providing trainings. To evaluate and monitor implementation of the program, region government arranges special meeting and call us to attend to have a common understanding.

Interviewer: For this program was there any consultancy workshop done?

Interviewee: A consultancy workshops were done.

Interviewer: Could you remember who had participated the consultancy workshop done?

Interviewee: The participants of the consultancy workshop were woreda and regional technical committee and string committee. The workshop was given in Yirgalem and Shabadino(Laku).

Interviewer: Who was organizer?

Interviewee: Organized by the Sidama region.

Interviewer: Before you were telling how to improve understanding of the people. Would you how it was done?

Interviewee: To create awareness in the people, different sectors are working in collaboration. The primary responsibility of our office is creating awareness how our people can improve crop production, how should they plant, how should they handle the farm, and how should they plant diversified crops on their own garden. We create awareness about the importance of the diversifications of the crop in their garden. We educate our people all the process beginning from planting crops to crop harvesting. We do these for our people because they are our brothers, mothers, fathers, children, etc. During the awareness creation, we show them practically on their garden rather than awareness creation through discussion. Practically, we show them how to diversify plants, how to handle the crops, how to farm in group, how to market the product, and how should women participate in farming. Regarding water provision, our office jointly work in collaboration with water development office to provide water, we create awareness how and when to utilize water, and how to conserve water. We educate about techniques of water conservation for dry season to enhance crop production in dry season. Moreover, we teach them how to withstand climate change. Another is we are giving training on the skill gaps of our farming professionals. We educate them to create better, health and productive children.

Interviewer: What works are done by your office on nutrition other than multi-sectorial collaboration program?

Interviewee: The other activity our office work on nutrition is coordination CEFTNET program. This program provides food and other support during emergency disaster whether natural or man-made, help very poor and hunger people. This program fills the gap of food shortage due to crop damage due to climate change, flooding, drought, etc. This program also supports with food some

prioritized poor families having large family members who have no farm lands. This program also provide food supports to orphans, very old peoples with no social support, weak people who cannot work and people with special needs. Moreover, for those who can work but poor, CEFTNET also provides public job opportunity such as cleaning roads, digging canal for irrigation, etc. by the regular monthly payments. During the natural or man-made emergency, we identify and list the people in urgent food need and we transfer report to the higher responsible bodies and higher bodies cross check and provide the support. CEFTNET program is also involving in replacement of crops damaged by the climate change, improved seeds, fertilizer, and farming training provision.

Interviewer: It is very nice! For the questions I ask, raise brief ideas that you think important. Are there responsibilities or oaths given to multi-sectorial nutrition program?

Interviewee: As I explained before, budgets and activities are shared among sectors and responsibilities are fulfilled according to shared activities. If one sector cannot accomplish its share of activities and responsibilities, that sector will be held accountable. For the example, in our woreda, health sector is responsible for leading, coordinating and reporting the multi-sectorial nutrition program activities. If there is gap in these responsibilities, health sector will be held accountable. Regarding budget utilization in each activity across the sector, sector which does not utilize budget properly for particular activity will be held accountable. For example, budgets for agricultural activities, the office of agriculture is accountable.

Interviewer: What is your long and short-term plans of your office on food program? Long-term plan is plan for one year, three years, or five years. Short-term plan is plan for less than one year.

Interviewee: Our short-term plan is to create crop diversification in gardens of all kebeles, to identify and select crops which our climates suitable most, to create awareness in our people, to timely provide inputs to farming to maximize crop production and beneficence of our people, and to enable people to produce sufficient and diversified food from their own garden. Our long-term plan is all people of woreda to become self-sufficient in food production, to produce surplus food, to provide quality surplus crop production to market, to enhance the income of the people, and to create happy, wealthier and healthier community.

Interviewer: ok! Would you explain shortly about the peoples’ responsibility, readiness, and determination to support this program?

Interviewee: There is big problems in the people especially very low level of awareness. The people are very ready and determined to accept support of the multi-sectorial nutrition program and they want to be beneficiary of the program. The people demand about the services of this program is a lot. The supply of the services of multi-sectorial nutrition program is very low in relative to high demand of the people to the services. To address all this demand, it needs a lot of budgets. We are working step by step increment of budget to address demand of our people. We have learned that most of our people expect or need certain help like awareness creation, technical and professional support, and inputs. We learn that if you give them certain professional help, they will show you work more. They are very descent, cooperative and docile, and want to be beneficiary of the program. They expect a lot from the program.

Interviewer: What is promising (hopeful) of multi-sectorial nutrition program?

Interviewee: There is an excess labor force in our farmers. If this all labor is used wisely and properly it can enhance economy of community. Moreover, if you timely provide sufficient farming inputs for this labor force, the crop production will be more, they become self-sufficient from their garden, betterment of their living standard.

Interviewer: For this multi-sectorial nutrition program what is about political determination, evolvement and support?

Interviewee: Based on my observation, a political determination, evolvement and supports are lot. String committee constitute of political personnel, they evaluate and monitor the activities of all sectors. They evaluate and appreciate strength of the sectors on the program; also, they identify gaps on weakness in certain activities in different sectors and motivate to work to strengthen weakness. The political determination, evolvement and support is invaluable for me.

Interviewer: Very nice! Do you have recommendations improve multi-sectorial nutritional program?

Interviewee: The aim or objectives usually come up to down. For me, it is better to participate professionals of bottom level to set objectives because professionals of bottom level understand real problems on the ground very well than else because they are working with people. I recommend involvement of both higher and bottom level professional to jointly set objectives/aims.

Interviewer: What is your recommendation to the sectors?

Interviewee: Very well! All sector should provide inputs timely, should work to raise awareness of the community to use their land properly to improve their life. Water development office should timely provide water that replace the rain. All sectors should provide inputs timely, activities are interdependent, delayed provision of certain input affects the other. The office of gender and child has responsibility to raise awareness in the rights of children and participation of the women in socio-economic affairs. To achieve goals of Seqota Declaration, sectors should do more efforts. Finance sectors should give priority to urgent and timely activities to enable to run activities depending their given time table. More coordinated and facilitated payment technique are expected from the finance office.

Interviewer: Now I finished my questions. Do you have something to add?

Interviewee: I don’t have other ideas to add. If the evaluation and monitoring continue to top bottom manager, the multi-sectorial nutrition program hopefully achieve its goals. Collaboration of the sectors should continue. The multi-sectorial collaboration and conjugation can change/transform the community, makes work fruitful and helps achieve common goals. Through sectorial collaboration efforts is making activities directly accomplished on the ground. Overall, multi-sectorial nutrition is very good and best method to overcome nutrition problems of our community. I have no more ideas. I thank you for having time together and I look forward for your recommendations and stake in successful implementation of the program.

Interviewer: we thank you for your time!
