# Supplementary material for: Exploring the challenges and opportunities of multisectoral nutrition programme in Ethiopia: A qualitative study on combating undernutrition during pregnancy
Source: PLoS One. 2025 Jul 3;20(7):e0311336. doi: 10.1371/journal.pone.0311336 (PMC12225801; doi:10.1371/journal.pone.0311336)
Supplement: S4 Data — (PDF) [file pone.0311336.s005.pdf]

Transcripts Interviews from H01

Interview I: IDI with 02 district H01

District: 02

Sex: p02

Age: 38 years

Marital status: Married

Professions: Bsc in health officer

Position: H001

Work experience: 6 years' experience on current position

Interviewer: Tell me about the nutritional problem in your region/district/locality?

Respondent: In our district at household level there is food insecurity. This food insecurity causes too many problems. district as you know it is priority hot spot area of nutrition problem. This district has hot climate and there is shortage of rain.

Probe: How do you see the nature of the nutritional problem in the local context?

Respondent: Nutrition problem is seasonal. When there was rain the problem was improved and when there was rain shortage problem aggravated.

Interviewer: How this problem trend looks from past to the present?

Respondent: Nutrition problem still now aggregated through time. In this year rain season is changed.

Interviewer: Have you heard about the multi-sectorial nutrition program?

Respondent: Yes.

Interviewer: If yes, would you say something about its policy, principles, and implementation?

Respondent: Nutrition activities didn't performed only by one sector it needs multi-sectorial collaboration. Near time after sekota declaration implemented concerned sectors collaborated. For example, education sector, health sector, water sector, and women affair. Around seven sectors works in collaboration on nutrition program.

Interviewer: What are the nutrition related activities of your office?

Respondent: h01 sector mainly works on awareness creation. Pregnant women and under five screening and growth monitoring program. Screened peoples linked to agriculture sector for

supplementary feeding. We teach the community how they prepare nutritious food, and how cultivate fortified crop. We provide treatment for sick persons.

Interviewer: What are the roles and responsibilities of your office to achieve the implementation of multi-sectorial of nutrition?

Respondent: Our sector leads this multi-sectorial program. Other sectors work in collaboration with us. Health office coordinates this program.

Interviewer: Tell me the ownership of the program?

Respondent: In current situation the owner of this program is government. Concerned body like multi-sector nutrition program members, community itself, and partners who support this program have responsibility.

Interviewer :detail the coordinator of the program and this body responsibility?

Respondent: Health sectors coordinate multi-sectorial nutrition program. Other sectors participate in this program.

Interviewer: Tell me the challenges of your office in relation to collaboration with other offices?

Respondent: All sectors didn't have equally awareness. Main challenges budget shortage and lack of training. Health sectors have big burden for this program because of nutrition activities main concern for this program. Other challenge is all sectors didn't participate equally.

Interviewer: detail about the procedure of program such as; about the annual plan, half year plan, quarterly plan, achievement report, meeting schedule, monitoring and evaluation related commitment.

Respondent: Program has monitoring and evaluation system. There is inter-sectorial plan, and each sector evaluate and monitor own performance regarding this program. Near time we evaluate nine months nutrition performance, and identified weakness and strengths. Program has meeting schedule but there is gap.

Interviewer: What challenges are there for the community to achieve a multi-sectorial nutrition program?

Respondent: There are challenges. The first challenge is the assume problems didn't solved. Second challenge is work done at community level is not satisfactory. There are a lot of problem but the way under go to solve these problems is not enough. There is interest of conflict.

Interviewer: the commitment of the community, for any resistance from the community?

Respondent: Communities are committed to perform what we inform to them. They are voluntary and cooperative. The other thing community is at risk but the response for community is very small. All kebeles are at risk regarding nutrition problem, but only selected kelebes get support from this program. This is big challenge. Sekota declaration works on household income generation this activity may decrease household food insecurity problem.

Interviewer: Tell me how the structure of this program is organized?

Respondent: Multi-sectorial nutrition program is at federal level there is Vise Prime minister then at region level there is Vise president They leads this program.

Interviewer: the reporting system of the program?

Respondent: Every month report from kebeles received by health centers. Health centers sent complied reports to health office then report sent to region. There is schedule and chain of when reports send and receive. There is DHS2 tool used for reporting system.

Interviewer: Say something about the program in relation to the budget?

Respondent: There is budget allocated by Federal government. Still now there is no additional budget support at woreda level. Allocated budget distributed to each sectors involved in the multi-sector nutrition program.

Interviewer: the financial admin, adequacy or shortage, and others?

Respondent: Allocated budget is not adequate. There is vast problem in the community but allocated budget is not enough to support community fully.

Interviewer: HR and other resource issues in your office?

Respondent: Regarding HR is not enough to support nutrition activities in our health office. In MCH pin point there is many activities for these activities human resource is not adequate to support program. It is difficult to handover all program activities by one person.

Interviewer: How did the professionals who work on a multi-sectorial nutrition program capacitate?

Respondent: As I told you sekota declaration creates opportunities. Regarding capacity building training there is gap. Capacity building training didn’t provided to the health extension workers. Household level training needed for the community. Quarterly review meeting done every six month.

Interviewer: Have you had the consultant workshop on this program?

Respondent: In our district consultant workshop didn’t facilitated.

Interviewer: How you involve the community to create awareness?

Respondent: This nutrition program is mainly government program. Specifically sekota declaration is works at community level. Especially, women development army, community leaders and different stakeholders those community members aware about the program. Different meeting and awareness creation activities done at community level.

Interviewer: What are the nutrition related programs other than multi-sectorial of your office?

Respondent: In this year **find and treat** company was done. We screen children and treat them at community level. We found around 4070 moderately acute malnourished children during community level screening. Those children enrolled to target supplementary feeding program. Around 2000 screened women supplementary feeding was done. **Find and treat** means those children and women with nutrition problem immediately treated at community level. Those malnourished children with complication treated in the health facility. In this year nutrition activity was given due attention and around three companies done.

Interviewer: Is there designated responsible body to coordinate the program?

If yes, how she/he is committed?

Respondent: Yes, there is one focal person for multi-sectorial nutrition program. His responsibilities are works in collaboration with sectors representatives, technical support, provide training, and works in collaboration with community.

Interviewer: What are strategic and operational plans of your office in multi-sectorial nutrition program?

Respondent: There is no strategic plan this considered as gap. All sectors works in collaboration for this program have own separate plan. There is no specific plan for multi-sectorial nutrition, but there is plan for sekota declaration.

Interviewer: How communities are committed to support the activity plan of this program

Respondent: Communities are committed to do for what you told to them. Sometimes there is problem in the community because of they didn't fully understand program aim. They sold income generation sheep which is provided by this program.

Interviewer: Tell the presence of promising work structure of this program?

Respondent: The first thing is working in collaboration. Multi-sectorial involvement creates the opportunity to solve problems easily. This program creates opportunity to work in together.

Interviewer: How is the political support of this program?

Respondent: Support for this program is very good. Administrators are ready to support this program including resource support. They are knowledgeable about the program because of this program lead by them. Nutrition is political issue by itself.

Interviewer: Does regional staff and district administrator support you?

Respondent: Yes, they support this program.

Interviewer: What do you think on the recommended strategy to improve the implementation of multi-sectorial nutrition program in your district/region?

Respondent: I recommend that additional sectors should be participated to this program. And non-governmental organization should participate. Other thing private business man should participate. Other concerned body engaged to this program. In addition, human resource and budget should be increased. Strengthening training and awareness creation to the community. These all about my recommendation.

Interviewer: How do you think these strategies can improve the multi-sectorial nutrition program?

Respondent: For example, when there is enough human resource the quality of service provided will improved. Regarding budget when there is adequate budget community support will increase. When frequent training provided to the health extension workers knowledge gaps filled. These all things improve the quality of services provided, and important to create health community.

Interviewer: Now that I'm almost done with my questions, you can express any concerns you have about this program?

Respondent: Thank you. It is better to have own office for multi-sectorial nutrition program. This improves information utilization and helps to make evidence based decision. I try to address all questions.

Interviewer: Thank you very much for your time and genuine response!

Respondent: Ok, thank you too.

Interview II: IDI with HNF

District: **<u>02</u>**

Sex: Male

Age: 30 years

Marital status: Married

Professions: Bsc in haealth service management

Position: h001

Work experience: 1 year experience on current position

Interviewer: Tell me about the nutritional problem in your region/district/locality?

Respondent: There is big nutrition problem because of this district is hot spot area. Last month **UNICEF** non-governmental organization which screen children nutrition status and give support for those malnourished children.

Probe: How do you see the nature of the nutritional problem in the local context?

Respondent: Our district face nutrition problem challenges for long time due rain shortage. Farmers cultivate seeds but due to rain shortage they are not effective. Even if there is rain many peoples in the community needs nutrition support.

Interviewer: How this problem trend looks from past to the present?

Respondent: Currently due to rainy season there is some improvement. The burden of nutrition problem still continued. Loka Abaya district one among 10 selected districts for sekota declaration in the region. There are seven sectors in the multi-sectorial nutrition program. Agriculture sector support nutrition activity, Water and mineral sector support hygiene and sanitation, education sector teach children about nutrition, and women's affair teach community about saving.

Interviewer: Have you heard about the multi-sectorial nutrition program?

Respondent: Yes. Sekota declaration works on under-two years children. We provide awareness creation training for 27 kebeles community representatives. We take sekota declaration work as routine work. Goats distributed to selected households for feed children to prevent nutrition problem. Multi-sectorial nutrition programs works to decrease stunting.

Interviewer: If yes, would you say something about its policy, principles, and implementation?

Respondent: Main goal is to decrease stunting zero at 2030 G.C. To decrease stunting all multi-sectorial program members work together. Sekota declarations lead by Vise prime minister at

federal level and at regional level lead by president advisor. They work in collaboration to minimize nutrition problem.

Interviewer: What are the nutrition related activities of your office?

Respondent: We teach pregnant women to have additional meal. Family planning service providing for example when family size increase food availability in the household level decreases this leads to nutrition problem, Iron folate supplementation, and Pregnant women forum. We teach 1000 days approach, exclusive breast feeding, complementary feeding, and pregnant women ante natal care.

Interviewer: Would you say more about each nutrition related activity in your office?

List of activities? Its aim? Any plan?

Respondent: Yes, we have plan. First of all we identify number of pregnant women, number of family planning women, and women on ANC (Ante Natal Care). After that we prepare plan. We advise women to start ANC early and to have syphilis test, and nutrition screening.

Interviewer: What are the roles and responsibilities of your office to achieve the implementation of multi-sectorial of nutrition?

Respondent: Our responsibility is to achieve what we plan. My aim in this program is to see health and happy community.

Interviewer: Tell me the ownership of the program?

Respondent: Multi0sectorial program lead by district administrator. There is council which lead by administrator. There are two committee steering and technical committee. Seven sectors have own technical committee they have monthly meeting. Every month district administrator call steering committee for meeting.

Interviewer: detail the coordinator of the program and this body responsibility?

Respondent: health office head is coordinator at district level. In steering committee He is secretarial. His responsibility is compiling reports from sectors.

Interviewer: Tell me the challenges of your office in relation to collaboration with other offices?

Respondent: There is challenge of commitment. Other challenge is vehicle problem to support community at hard to reach area.

Interviewer: detail about the procedure of program such as; about the annual plan, half year plan, quarterly plan, achievement report, meeting schedule, monitoring and evaluation related commitment.

Respondent: Different nutrition support comes through agriculture sector. There is gap on planning and meeting schedule. We have own plan in health office.

Interviewer: What challenges are there for the community to achieve a multi-sectorial nutrition program?

Respondent: At community level there is food insecurity. The main challenge is nutrition problem at community level. Communities accept what we inform.

Interviewer: the commitment of the community, for any resistance from the community?

Respondent: Communities accept what we inform.

Interviewer: Tell me how the structure of this program is organized?

Respondent: Multi-sectorial nutrition program has manual. In the manual everything written what is role and responsibility of each sector.

Interviewer: the reporting system of the program?

Respondent: Reporting system integrated with other activities. Reporting system through DHS2 tool, and our meeting twice in the month.

Interviewer: Say something about the program in relation to the budget?

Respondent: Around 400080 birr allocated by sekota declaration.

Interviewer: the financial admin, adequacy or shortage, and others?

Respondent: Allocated budget and work burden is not equally.

Interviewer: HR and other resource issues in your office?

Respondent: Human resource is not adequate in our office.

Interviewer: How did the professionals who work on a multi-sectorial nutrition program capacitate?

Respondent: Sekota declaration program allocate budget for training. Agriculture extension workers and focal from each sector participated training. There is no capacitating training provided by multi-sectorial nutrition program.

Interviewer: Have you had the consultant workshop on this program?

Respondent: Sekota declaration provided consultative workshop last month. There is no separate consultative workshop provided by multi sectorial program.

Interviewer: How you involve the community to create awareness?

Respondent: Community awareness creation provided when this program launched. Religious leader, community leader, HDA, and health extension workers participated from 22 kebeles. From each kebele around five participants involved. There is nutritional council at kebele level.

Interviewer: What are the nutrition related programs other than multi-sectorial of your office?

Respondent: Women screening, and children above 6 month screened for nutrition status.

Interviewer: Is there designated responsible body to coordinate the program?

If yes, how she/he is committed?

Respondent: At district level there is one coordinator. His responsibility is evaluating performance and identifying gaps.

Interviewer: What are strategic and operational plans of your office in multi-sectorial nutrition program?

Respondent: There is five years strategic plan and operationalized for one year. One year operational plan divided to 12 months. We compare last year performance with current year performance. Woreda transformation agenda incorporate this multi-sectorial plan. In our operational plan around 3955 pregnant women estimate. This plan divided to each month then to weeks.

Interviewer: How communities are committed to support the activity plan of this program

Respondent: Communities are committed to accept what we teach.

Interviewer: Tell the presence of promising work structure of this program?

Respondent: Availability of health extension program, HDA, and health professional are one promising for continuity of this program.

Interviewer: How is the political support of this program?

Respondent: Seven sectors present report to district administrator. District fill identified gaps and whenever there is difficult they report to region. Concerning support I raise there is gap. When we report number of malnourished children to the region they didn’t give as adequate support. Community didn’t get enough support currently.

Interviewer: What do you think on the recommended strategy to improve the implementation of multi-sectorial nutrition program in your district/region?

Respondent: I recommend that it is better to treat malnutrition problem according to health professional ethics. Other thing is avoiding wrong report. Additional all sectors should work in

collaboration. Necessary support should be given to the community. Concerned body should investigate community problem.

Interviewer: How do you think these strategies can improve the multi-sectorial nutrition program?

Respondent: Guideline should be prepared if there is no guideline. Stakeholders should evaluate performance regularly.

Interviewer: Now that I'm almost done with my questions, you can express any concerns you have about this program?

Respondent: My point of view this program is important to solve community problem. Support we provide to the community should be identified problem based. I support community as much as possible I can. It is better to scale up this program to other area.

Interviewer: Thank you very much for your time and genuine response!

Respondent: There is no other thing above this topic it is sensitive topic. Community will be product if nutrition status is good.

Interview III: IDI with 01 district H01

District: 01

Sex: Male

Age: 36 years

Marital status: Married

Professions: Bsc in clinical Pharmacy

Position: H01

Work experience: 3 years' experience on current position

Interviewer: Tell me about the nutritional problem in your region/district/locality?

Respondent: 01 one of hot spot area to nutrition problem. Sometimes there is rain shortage in this area it leads to nutrition problem. There are malnutrition children and mother. Maternal and child health pin point in our office routinely screen children and women found that many

malnourished children and mother. There are 14 rural kebeles and one urban kebele in the 01 district. The nutrition problem not equally distributed in every kebeles.

Probe: How do you see the nature of the nutritional problem in the local context?

Respondent: The nature of nutrition problem has seasonal pattern. The number of malnourished increase in number where there is climate change.

Interviewer: How this problem trend looks from past to the present?

Respondent: It varies each year. There is increment in number nutrition problem in this year when compared to last year. When we screen pregnant and lactating women the number of malnutrition increased to this group. This shows problem trend increasing.

Interviewer: Have you heard about the multi-sectorial nutrition program?

Respondent: Five up to six sectors work in collaboration with us.

Probe: If yes, would you say something about its policy, principles, and implementation?

Respondent: At national level multi-sector nutrition program lead by vise prime minister. Specifically sekota declaration launched starting from national level to selected district in region. Regarding sekota declaration we emphasis to decrease under two years children stunting to zero. Generally we design strategy on nutrition program and planned to cascade to the community.

Interviewer: What are the nutrition related activities of your office?

Respondent: As health office, firstly we screen under-five children nutrition status. Then, we report to those concerned body to give us nutritional support, and provide feeding support to the malnourished. Secondly, we screen pregnant and lactating women. For those moderately malnourished enrolled outpatient feeding program. Severe malnourished treated in the health facilities.

Interviewer:: Would you say more about each nutrition related activity in your office? List of activities? Its aim? Any plan?

Respondent: Yes, we have plan. We plan during woreda base plan every year. In addition, under MCH (maternal and child health) pin point we plan nutrition related activity.

Interviewer: What are the roles and responsibilities of your office to achieve the implementation of multi-sectorial of nutrition?

Respondent: Well, our role in implementation of multi-sectorial nutrition program we plan and evaluate the performance with technical committee which leads by health office head. Finally, we present to steering committee which leads by district administrate head.

Interviewer: Tell me the ownership of the program?

Respondent: District administrator head leads steering committee. He is owner for this program. Health office head is secretarial for this program.

Interviewer: detail the coordinator of the program and this body responsibility?

Respondent: In our district there is one coordinator for sekota declaration. He is responsible for coordinating this program.

Interviewer: Tell me the challenges of your office in relation to collaboration with other offices?

Respondent: There is communication problem between sectors this considered as challenge. There is no other challenges I raise.

Interviewer: detail about the procedure of program such as; About the annual plan, half year plan, quarterly plan, achievement report, meeting schedule, monitoring and evaluation related commitment.

Respondent: We evaluate performance every month and action plan developed for those identified gaps. We have annual plan and operationalize this plan to 12 months. We evaluate reports before send to next level.

Interviewer: What challenges are there for the community to achieve a multi-sectorial nutrition program?

Respondent: To support communities transport access needed due to budget shortage; we face challenges to support community regularly. Other thing is commitment problem.

Interviewer: the commitment of the community, for any resistance from the community?

Respondent: Currently community committed to work what we inform to them. They have interest to the program.

Interviewer: Tell me how the structure of this program is organized?

Respondent: As I told you steering member are sectors heads. They are politician support this program.

Interviewer: the reporting system of the program?

Respondent: First report we gather from each sectors and complied. We send complied report to the region. And they give feedback weak activities to be corrected. At community level there is

steering committee lead by kebele administrator and health extension workers focal to this committee. We evaluate kebele steering committee performance.

Interviewer: Say something about the program in relation to the budget?

Respondent: Federal minister of health allocate budget to this program, but region didn't allocate budget this program. Minister of health send budget with action plan of what activities will done by allocated budget. Agriculture sector buy livestock and Hen to the community. We evaluate by budget utilization performance with finance bureau.

Interviewer: the financial admin, adequacy or shortage, and others?

Respondent: Allocated budget is not adequate, but it is adequate to support few households. In generally it is not adequate to undergo all activities.

Interviewer: HR and other resource issues in your office?

Respondent: In generally health sector human resource is enough we have no problem regarding this. In health sector there are two type of human resource. The first one is health professional. And the second one is supporter staff.

Interviewer: How did the professionals who work on a multi-sectorial nutrition program capacitate?

Respondent: There is no training prepared only for multi-sectorial nutrition program. Only once two day training provided to capacitate this program.

Interviewer: Have you had the consultant workshop on this program?

Respondent: There is no workshop for this program only.

Interviewer: How you involve the community to create awareness?

Respondent: Well, when this program was launched community representative participated. For example religious leaders, health extension workers, school director, elders and other concerned body participated. Awareness created about this program and those participants teach whole community. We teach the community how prepare nutritious food, and when brought sick to health facilities.

Interviewer: What are the nutrition related programs other than multi-sectorial of your office?

Respondent: For example in Agriculture sector **SafetyNet** program support selected malnourished children and households by providing food. This sector receives information from health sector. In addition health sector cascade health education activities to the community by using health extension workers in the community level.

Interviewer: Is there designated responsible body to coordinate the program?

If yes, how she/he is committed?

Respondent: There is focal person and committed for work. Their responsibility is support nutrition related activities in the community level and also in the office.

Interviewer: What are strategic and operational plans of your office in multi-sectorial nutrition program?

Respondent: For multi-sectorial nutrition program we have own plan. We cascade this plan to the community. Currently we work according to our plan.

Interviewer: How communities are committed to support the activity plan of this program Respondent: Well, as I told you without community involvement it is difficulty performs this program activities. When we inform to the community about this program, they didn't ignore our information. They are committed to perform what we told to them.

Interviewer: Tell the presence of promising work structure of this program?

Respondent: At every beginning, we are committed for multi-sectorial program accepted as this program our program. There is no big challenge that hinders continuity of this program. We work to support our community, so program sustainability is not questionable. Our commitment determines continuity of the program because community accepts what we inform to them. If we are strong enough to support program, this program will sustain.

Interviewer: How is the political support of this program?

Respondent: Community leader accept as this program activities are our work. They work in collaboration with us when we screen nutritional status in the community level. From top to bottom we work in collaboration.

Interviewer: Does regional staff and district administrator support you?

Respondent: As I told you for district administrator this program activities are his job to support. Sometimes regional staffs support this program.

Interviewer: What do you think on the recommended strategy to improve the implementation of multi-sectorial nutrition program in your district/region?

Respondent: Still now we are on the work, even if there are challenges. My recommendation is that it is better to solve challenges I raised. For example, transportation vehicle, different trainings to update our work, and supportive supervision. If these things addressed nothing hinder improvement this program. Specifically budget related challenges that obstacle progress of the

program. Not only for health sector, but also for all multi-sector nutrition program members these obstacles challenge them.

Interviewer: How do you think these strategies can improve the multi-sectorial nutrition program?

Respondent: As you know in the rural community the topography of land is good to support without vehicle. Not only vehicle as challenge but also fuel is one challenge. If this challenge solved there is no obstacles hinder our support rural community. If these things full field as I told you the staffs are very committed to support this program.

Interviewer: Now that I'm almost done with my questions, you can express any concerns you have about this program?

Respondent: According to government policy and strategy this program ruin with other program. I recommend that it is better to ruin this program separately. This program should ruin only nutrition and nutrition related activities. For example at district level in our sector this program ruin under MCH pin point. I recommend that this program should have separate office and additional human power should be added to scale up this program. In addition to update involved health professionals and health extension workers regular training is important to strength program. Grass root assessment research is important to address really community problems. All multi-sector nutrition program members should collaborate to do community based research.

Interviewer: Thank you very much for your time and genuine response!

Respondent: Thank you too.

Interview IV: IDI with 01 District H001

Woreda/district/partner/region office name: 01

Sex: Male

Age: 35 years

Marital status: Married

Professions: Nurse

Position: H001

Work experience: 7 years' experience on current position

Interviewer: Tell me about the nutritional problem in your region/district/locality?

Respondent: There is nutrition related problem in this district. Firstly, Even though there is improvement of malnutrition cases through time still now in our district maternal and child malnutrition problem increased in number. Now a day, there is no agriculture product not available for community due to rainy season this aggravates the problem.

Interviewer: How do you see the nature of the nutritional problem in the local context?
Respondent: The nature of nutrition problem in this area is seasonal. Starting March month up to August month the number of malnutrition cases increasing. This indicates as the seasonal pattern of the problem. After August month the cash crop, corn and other product may available for farmer this reduces nutrition problem. In addition, when community didn't store the product in their house this may causes nutrition problem.
Interviewer: How this problem trend looks from past to the present?
Respondent: When we compare to the past trends in the present time there is improvement of nutrition problem. For example there are two types of malnutrition. There first one is severe malnutrition which have seasonal pattern. And the second one is chronic malnutrition which didn't affected by seasonal pattern. In general in our district the nutrition problem shows improvement time to time.
Interviewer: Have you heard about the multi-sectorial nutrition program?
Respondent: Yes, I heard. At district level multi-sectorial nutrition program coordinated by health sector. Those six sectors involved for multi-sectorial nutrition program have contribution to this program. The program has principles designed by minister of health. There are different committees to evaluate the programs performance. For example steering committee lead by district administrator and technical committee lead by health sector head. When we see each sectors role education sector teach about behavior change education, Agriculture sector distribute nutritious seed to the community, Livestock sector provide goat to the households to give goat milk to their children and also provide hen to use egg to their children, and Water sector works on WASH and safe water access. So, multi-sectorial program is very important strategy for nutrition.
Interviewer: What are the nutrition related activities of your office?
Respondent: In this sector nutrition related activities is core activities. Multi-sectorial nutrition program focus on to decrease stunting prevalence zero at 2030. Committee evaluates the performance of each sector routinely. Nutrition related activities are one of our jobs. For example common nutrition related activities under five years children nutrition status screening, deworming for children and women, Vitamin A supplementation, For pregnant women Iron folate supplementation, Severe Acute Malmutrion(SAM) treatment at health facilities, and OTP(Outpatient therapeutic program).

Interviewer: What are the roles and responsibilities of your office to achieve the implementation of multi-sectorial of nutrition?

Respondent: Our sector role?

Interviewer: Yes.

Respondent: Our sector role is evaluating weekly performance of six sectors performance because of health sector head is technical committee head. We go to each kebeles monitor the performance what was done and what was given to selected households.

Interviewer: Tell me the ownership of the program?

Respondent: Multi-sectorial nutrition program lead by district administrator head because of, he is steering committee head. Program owner is district administrator head. Steering committee members are six sectors head. They meet to evaluate the performance of the program and allocate budget to each sector.

Interviewer: detail the coordinator of the program and this body responsibility?

Respondent: Multi-sectorial nutrition program coordinated by health sector. Starting from planning of the program activities health sector monitor program performance up to the end of year. Sekota declaration program have one coordinator in this district and he work with us.

Interviewer: Tell me the challenges of your office in relation to collaboration with other offices?

Respondent: There is a commitment gap on the sectors head. Programs performance needs to be evaluated routinely, but sectors head didn't do this due to politics work load this one is one challenge. Other challenge related to resource. There must be resource to support community at grass root, to check works done, and the other thing is different training needed but this didn't done. Still no we used the budget given by minister of the health. There is budget shortage to accomplish different activities.

Interviewer: detail about the procedure of program such as; About the annual plan, half year plan, quarterly plan, achievement report, meeting schedule, monitoring and evaluation related commitment.

Respondent: Yes, there is annual plan. Sekota declaration has five years strategic plan and operationalized plan for one year. Monthly report sent to region every month. Technical committee have weekly meeting and steering committee have monthly meeting. They have meeting schedule six sectors present report and take minute record. And there is annually review meeting. In review meeting participants were health extension workers, communities and school directors.

Interviewer: What challenges are there for the community to achieve a multi-sectorial nutrition program?

Respondent: At community level there is resource shortage to support community. For example to buy livestock for community there is no enough budgets. Last year livestock distributed to 25 households, but in this year livestock distributed only for 9 households.

Interviewer: the commitment of the community, for any resistance from the community?

Respondent: Still now community didn't resist the program. There is no commitment problem of the community.

Interviewer: Tell me how the structure of this program is organized?

Respondent: Minister of health designed structure of multi-sectorial nutrition program approved it. The program incorporated in the Health sector transformation plan (HSTP). Multi-sectorial integration is very important not only for nutrition program, but also for other programs. The other thing is district level concerned body is committed to this program.

Interviewer: the reporting system of the program?

Respondent: We report to the region. Every month six sectors send report to our office complied by the coordinator after that report through both hard copy and soft copy. And also online data base used for reporting.

Interviewer: Say something about the program in relation to the budget?

Respondent: For this program according to the guideline budget allocated by minister of health and region matching fund. But, last year budget allocated by minister of health and for this year 1.8 million budget allocated by minister of health to our district. This budget distributed to six sectors. This budget is very small to undergo works.

Interviewer: the financial admin, adequacy or shortage, and others?

Respondent: Each sector administer allocated budget. Overall budget monitor by steering committee head (district administrator head). Both technical committee and steering committee evaluate routinely utilization of budget according to the plan. For example budget allocated for health sector lead by health sector head. But, as I said before allocated budget is not enough. Region and district didn't allocated additional budget for this program.

Interviewer: HR and other resource issues in your office?

Respondent: In this office there are three officers for MCH (Maternal and Child health). Among them one is nutrition focal. He supports health extension program and other activities. There is work burden in MCH. There is human resource (HR) shortage for MCH.

Interviewer: How did the professionals who work on a multi-sectorial nutrition program capacitate?

Respondent: Yes. At regional level training was provided. There is opportunity of training for nutrition activities.

Interviewer: Have you had the consultant workshop on this program?

Respondent: Experience sharing was done in our district.

Interviewer: if yes, probe for details like who were participants?

Respondent: Technical committee and steering committee participated in experience sharing.

Interviewer: How you involve the community to create awareness?

Respondent: Yes, Every year this program launched. Kebele administrator, religious leader, health extension workers, and health development arm (HDA) were participated when this program launched.

Interviewer: What are the nutrition related programs other than multi-sectorial of your office?

Respondent: Under five children and pregnant women nutrition status screening using MUAC. Moderate Acute Malinutrition (MAM) enrolled in target supplementary feeding program and supportive feeding was given for women and children. Severely Acute malnourished children admitted to health facilities and treated. Vitamin A supplementations.

Interviewer: list of activities this program? Its aim? Any plan?

Respondent: It has plan. Reported and evaluated separately. Feedback was given.

Interviewer: Is there designated responsible body to coordinate the program?

If yes, how she/he is committed?

Respondent: Yes, there is designed one person. He is committed for his work.

Interviewer: What are strategic and operational plans of your office in multi-sectorial nutrition program?

Respondent: Yes, there is five years strategic plan. Operationalize plan was planned. We plan to reduce stunting to zero level.

Interviewer: How community are committed to support the activity plan of this program

Respondent: Community accepts what we told to them. They are committed. They brought children's for nutritional screening and maintain children health according to our advice.

Interviewer: Tell the presence of promising work structure of this program

Respondent: At every beginning, the availability of both committees steering and technical committee. They monitor and evaluate works. Other thing budget support.

Interviewer: How is the political support of this program?

Respondent: Regional vice president evaluates reports. As I said before district administrator head strictly support this program. So that, there is political support. District administrator head follows and evaluate the program.

Interviewer: What do you think on the recommended strategy to improve the implementation of multi-sectorial nutrition program in your district/region?

Respondent: Research based works. It is better root cause analysis of the problem and takes action for problem early. The other thing is that multi-sectorial team routinely evaluates what work was done. It is better to participate the community to eliminate stunting. Strengthen multi-sectorial collaboration.

Interviewer: How do you think these strategies can improve the multi-sectorial nutrition program?

Respondent: For example at community level farmers center training is important how they prepare local nutritious reach crop, and treat ill children**.** Scaling up good is trained is important.

Interviewer: Now that I'm almost done with my questions, you can express any concerns you have about this program?

Respondent: Nothing**.**

Interviewer: Thank you very much for your time and genuine response!

Respondent: Thank you too.

03 District

Interview V: IDI with 03 district H01

District: 03

Sex: Male

Age: 32 years

Marital status: Married

Professions: Bsc in Health officer

Position: H01

Work experience: 2 year and 7 months experience on current position

Interviewer: Tell me about the nutritional problem in your region/district/locality?

Respondent: There is big nutritional problem in our district. Majority of affected groups due to this problem are children. Not only children but also other age groups also affected. There are SC (Stabilization center), OTP (Outpatient therapeutic program) and MAM (Moderate Acute Malnutrition) programs. These programs initiated in this district because of hot spot priority area in the region due to nutrition problem.

Interviewer: How do you see the nature of the nutritional problem in the local context?

Respondent: Seasonal problem related with availability of food. People's live rural area more affected by problem because of there is no adequate food. Nutrition problem more prevalent at winter season.

Interviewer: How this problem trend looks from past to the present?

Respondent: Relatively there is some improvement when we compare with past five and six years, but nutrition problem still prevalent. Near time many children enrolled to supportive program. Starting last year many children admitted in the health facility in SC program. Rather to say improved it is better to say severed.

Interviewer: Have you heard about the multi-sectorial nutrition program?

Respondent: Yes. Our district one of among selected districts for sekota declaration. Six sectors work in collaboration for this program. Health sector, agriculture sector, livestock sector, women and children affairs sector, education sector, and water and mineral sector works in collaboration. In addition finance sector.

Interviewer: If yes, would you say something about its policy, principles, and implementation?

Respondent: Main policy is all sectors work in collaboration to decrease under-two year children stunting to zero. All sectors should work to accomplished tasks given to them. For example health sector should work on nutrition education, and growth monitoring services. Other sectors should work on responsibilities assigned.

Interviewer: What are the nutrition related activities of your office?

Respondent: Nutrition related activities under-two year growth monitoring (GMP), under-five nutrition screening by using MUAC (Mid-upper Arm Circumference), Pregnant and lactating women MUAC check, Vitamin A supplementation for children 6 month up to 5 year, deworming by using Albendazole, and pregnant women Iron folate supplimantation.

Interviewer: Would you say more about each nutrition related activity in your office? List of activities? Its aim? Any plan?

Respondent: Yes, we have plan.

Interviewer: What are the roles and responsibilities of your office to achieve the implementation of multi-sectorial of nutrition?

Respondent: In multi-sectorial nutrition program health sector role is coordinator. There are two committee steering and technical committee. Steering committee are sectors heads and lead by district administer. Technical committees are each sectors focal lead by health office head. Technical committee has meeting ever two weeks and their performance evaluated by health office head.

Interviewer: Tell me the ownership of the program?

Respondent: Health sector.

Interviewer: detail the coordinator of the program and this body responsibility?

Respondent: At district level there is only one coordinator. His role follows each sectors and work in collaboration with them.

Interviewer: Tell me the challenges of your office in relation to collaboration with other offices?

Respondent: Challenges are work burden, and each sector give preference for own sector task. Other thing is staffs commitment.

Interviewer: detail about the procedure of program such as; About the annual plan, half year plan, quarterly plan, achievement report, meeting schedule, monitoring and evaluation related commitment.

Respondent: Yes, there is plan. Each sector have plan for multi-sectorial nutrition program. There are indicators to monitor program.

Interviewer: What challenges are there for the community to achieve a multi-sectorial nutrition program?

Respondent: There is no problem at community level. They are committed.

Interviewer: the commitment of the community, for any resistance from the community?

Respondent: They didn't resist the program because of we launched program twice. They are thankful for this program. There are selected households (HHs) for this program. These HHs take support from sekota declaration.

Interviewer: Tell me how the structure of this program is organized?

Respondent: These program leads by district administer head. He leads steering committee. They evaluate overall program performance and meet monthly. Technical committee lead by health office head. Program coordinator gathers information from sectors.

Interviewer: the reporting system of the program?

Respondent: Reporting system from bottom to top. From top feedback given to the district. Health extension workers and agriculture extension workers sent reports from community level to health office and agriculture of respectively.

Interviewer: Say something about the program in relation to the budget?

Respondent: Budget allocated is not adequate. Each sector administer distributed budget.

Interviewer: the financial admin, adequacy or shortage, and others?

Respondent: There is budget shortage.

Interviewer: HR and other resource issues in your office?

Respondent: There is no human resource problem. But we have other resource problem including furniture problem because of our district is newly emerged district.

Interviewer: How did the professionals who work on a multi-sectorial nutrition program capacitate?

Respondent: Multi-sectorial nutrition program training facilitated by region usually. At district level we didn't facilitate training but we organize review meeting to evaluate performance frequently.

Interviewer: Have you had the consultant workshop on this program?

Respondent: There is no consultant workshop facilitated.

Interviewer: How you involve the community to create awareness?

Respondent: Yes, we create awareness. Kebele leaders, health extension workers, and agriculture extension workers were toke training.

Interviewer: What are the nutrition related programs other than multi-sectorial of your office?

Respondent: As I told you before. Children severe acute malnutrition with complication admitted to SC center. In OPT program every week **pnupnut** nutrition support given for children. Children with MAM SUP(Supplimentary Pnupnut) provided. Cooking demonstration was shown to women how to prepare food for under-two year children to reduce stunting.

Interviewer: Is there designated responsible body to coordinate the program?

Respondent: Yes. All sectors have responsibility for this program. Overall we have obligation to evaluate program performance. Especially multi-sectorial-nutrition program assigned coordinator has responsibility with accountability. His given written letter which states what is his responsibility in detail.

Interviewer: Do think she/he is committed?

Respondent: As I told you before there is gap on commitment. To tell truth six sectors have focal and they are active enough. But some have negligence. For example last year we changed nutrition focal person because of his performance was low.

Interviewer: What are strategic and operational plans of your office in multi-sectorial nutrition program?

Respondent: Yes, there is five year strategic plan and one year operational plan. Overall sekota declaration has strategic plan.

Interviewer: How communities are committed to support the activity plan of this program

Respondent: Yes, they are committed. Even if there is some problem on utilization of resource, the communities are committed. Regional supervision team appreciates community commitment.

Interviewer: Tell the presence of promising work structure of this program?

Respondent: One thing is sectors collaboration is good opportunity for continuity of the program.

Interviewer: How is the political support of this program?

Respondent: District administer head support programs. But finance sector utilizes program budget for other activity.

Interviewer: Does regional staff and district administrator support you?

Respondent: Yes. They support this program.

Interviewer: What do you think on the recommended strategy to improve the implementation of multi-sectorial nutrition program in your district/region?

Respondent: My recommendation to this program. It is better to create awareness within sectors about the program. For example, in case one sector head changed due to many reason new comers for this position will be new for this program. So that frequent awareness creation about the program is important. Other thing political support should be give due emphasis. In addition, capacity building for staffs is important. Resource shortage should be solved. Finally, team should be created in each sector only for evaluation and monitoring purpose.

Interviewer: How do you think these strategies can improve the multi-sectorial nutrition program?

Respondent: One recommendation I raised is capacity building. Majority of community didn't aware sekota declaration. To fill this gap frequent capacity building and awareness creation is important. Without political support it is difficult to accomplish tasks. For decision making political support is very crucial.

Interviewer: Now that I'm almost done with my questions, you can express any concerns you have about this program?

Respondent: No other thing I will add. I saw multi-sectorial nutrition program is very important for community. As I told you before frequent capacity building training is necessary for health professionals and health extension workers

Interviewer: Thank you very much for your time and genuine response!

Respondent: Thank you too.

Interview VI : IDI with 03 H001

District: 03

Sex: Male

Age: 34 years

Marital status: Married

Professions: Bsc in Health officer

Position: HNF

Work experience: 1 year month experience on current position

Interviewer: Tell me about the nutritional problem in your region/district/locality?

Respondent: Well, this district has nutrition problem due to rain shortage and frequent conflict. For children with malnutrition pnupnut support provided. Frequently we face nutrition support gaps from partners and government. For one month we didn't receive nutritional support specifically pnupnut from concerned body. Due to frequent conflict in Bilate Zuria there are many malnourished people. This pnupnut shortage is one problem in our district.

Interviewer: How do you see the nature of the nutritional problem in the local context?

Respondent: Winter season nutrition problem increase in numbers.

Interviewer: How this problem trend looks from past to the present?

Respondent: Near time there is some improvement due to health education we provided. Nutrition problem caused not only by scarcity of food, but also due to lack of knowledge how handle available food. There is an improvement of nutrition problem trends.

Interviewer: Have you heard about the multi-sectorial nutrition program?

Respondent: Yes, surely I heard this program. Only two years this program was launched.

Interviewer: If yes, would you say something about its policy, principles, and implementation?

Respondent: When map designed the main aim is multi-sectorial involvement to reach whole community. Program policy designed to help community. Different training facilitated for the community at lower level.

Interviewer: What are the nutrition related activities of your office?

Respondent: We work starting child birth monitor weight for age. We give education on exclusive breast feeding for six month, complimentary feeding after six month, disadvantage of local traditional medicine, pregnant and lactating women additional meal, ANC and PNC services, and pregnant and lactating women nutritional screening. And also immunization service and Vitamin A supplementation. Food preparation demonstration at community level.

Interviewer: Would you say more about each nutrition related activity in your office? List of activities? Its aim? Any plan?

Respondent: Yes, we have plan. Without plan it is difficult do any activity.

Interviewer: What are the roles and responsibilities of your office to achieve the implementation of multi-sectorial of nutrition?

Respondent: Our office role in multi-sectorial nutrition program is coordinating the program. Majority activities of multi-sectorial program focus on nutrition. Nutrition related activities are health office task. We monthly evaluate the performance of this program.

Interviewer: Tell me the ownership of the program?

Respondent: Firstly, district administer head is owner of this program. Then health sector own program.

Interviewer: detail the coordinator of the program and this body responsibility?

Respondent: There is one government assigned coordinator at district level. He plan monthly activity and monitor performance of the program.

Interviewer: Tell me how the structure of this program is organized?

Respondent: As I told you before there is seven sectors involved to this program. Health sector gives education on nutrition, Agriculture sector works on production, Water and mineral sector works on water safety, Finance sector is important to manage budget, and women and children sector also has big responsibility.

Interviewer: the reporting system of the program

Respondent: From community level we receive report monthly, and we send to the region monthly.

Say something about the program in relation to the budget?

Respondent: There is shortage of budget. We face challenges when we prepare training. During supportive supervision we face budget shortage.

Interviewer: the financial admin, adequacy or shortage, and others

Respondent: Each sector administer distributed budget.

Interviewer: HR and other resource issues in your office?

Respondent: We didn’t face challenge regarding human resource. As told you before we have no other adequate resource. And we have no motor cycle to support community. Transportation is very crucial to support rural community. Other thing currently we use paper to send report. It is better to digitalize reporting system. We face computer shortage. Sometime we use phone telegram to send report.

Interviewer: How did the professionals who work on a multi-sectorial nutrition program capacitate?

Respondent: Well, for me one year on this position. I trained only one training about this program. There is gap regarding training. From 19 kebeles 2 kebeles selected for this program. From selected kebeles 12 HHs (Households) enrolled to this program. Only two kebeles representatives toke training. I think this is not adequate. One opportunity is involvement of multiple sectors. All sectors give own training concerning program.

Interviewer: Have you had the consultant workshop on this program?

Respondent: Yes.

Interviewer: if yes, probe for details like who were participants?

Respondent: Seven sectors focal participated.

Interviewer: How you involve the community to create awareness?

Respondent: As I told you before we give awareness creation training for community representatives. Last year 19 kebeles representatives participated when this program launched. For example, religious leader, HAD, and other concerned body.

Interviewer: What are the nutrition related programs other than multi-sectorial of your office?

Respondent: we give education on nutrition, ANC, and nutrition screening. HEWs give PNC service; give education on child nutrition, Vitamin A supplementation, deworming children and pregnant women and GMP (Growth monitoring program).

Interviewer: Is there designated responsible body to coordinate the program?

If yes, how she/he is committed?

Respondent: Our sector toke responsibility to coordinate programs. Nutrition focal given responsibilities to coordinate nutrition activities within this program.

Interviewer: What are strategic and operational plans of your office in multi-sectorial nutrition program?

Respondent: Strategic plan of this program is interesting. I recommended all sectors should be included in the strategic plan. Operational plan derived from strategic plan.

Interviewer: How community are committed to support the activity plan of this program?

Respondent: Well, firstly this multi-sectorial nutrition program creates awareness to the community. Community use knowledge for action what they trained. They are committed.

Interviewer: Tell the presence of promising work structure of this program

Respondent: For sustainability of this program is enrollment of poorest HHs because they handle properly provided support. Other thing is regular training provided to the community by different sectors, and involvement of multiple sectors.

Interviewer: How is the political support of this program ?

Respondent: Political support of this program toke major role. As I told you before community accept things come through administer. District administer takes big role in this program.

Interviewer: What do you think on the recommended strategy to improve the implementation of multi-sectorial nutrition program in your district/region?

Respondent: For this I raise one thing to give support for lower level community transportation is important. It is better to fill gaps on transportation access. Other thing training gaps should be solved. Finally, partners should increase support. We have 19 kebeles but only 2 kebeles selected for this program. I recommend that all kebeles should be included to this program.

Interviewer: How do you think these strategies can improve the multi-sectorial nutrition program?

Respondent: As I rose before to improve this program we have to strength communication with the community. To solve transportation challenges we have to collaborate each other. Secondly, we have to effectively use available resources.

Interviewer: Now that I'm almost done with my questions, you can express any concerns you have about this program.

Respondent: Many things discussed. I raise only that transportation problem should be solved, additional partners to this program should be involved, and tablets support for reporting system should be considered.

Interviewer: Thank you very much for your time and genuine response!

Respondent: Thank you.

04 District

Interview VII: IDI with H01

District office name: 04

Sex: Male

Age: 35 years

Marital status: Married

Professions: Masters in nutrition

Position: H01

Work experience: 1 year experience on current position

Interviewer: Tell me about the nutritional problem in your region/district/locality?

Respondent: There is nutrition problem in our district. Main reason for this problem is farmers cultivate similar seed grain throughout the year and there is shortage of land in this district. Other reason is they didn't have habit of saving seed grain rather they exchange with money it without saving. Due to nutrition problem health risk factors increased. Most of time under-five children affected by nutrition problem during winter season.

Interviewer: How do you see the nature of the nutritional problem in the local context?

Respondent: Nutrition problem persistent in this district. Most of time aggravated at winter season July up to September.

Interviewer: How this problem trend looks from past to the present?

Respondent: In our district nutrition problem increasing in number. When we saw different research did in this district the burden of nutrition problem increased.

Interviewer: Have you heard about the multi-sectorial nutrition program?

Respondent: Yes, I heard.

Interviewer: If yes, would you say something about its policy, principles, and implementation?

Respondent: Six sectors involved in multi-sectorial nutrition program. Health sector, water and mineral sector, women and children sector, agriculture sector, livestock sector and education sector.

Health sector activities in this program mainly focus on nutrition specific performance, nutrition screening, Vitamin A supplementation, behavior change education, and provide supplementary feeding for those malnourished children. Agriculture sector activities in this program is nutrition sensitive seed grain farm, and they teach community to farm different seed grain on similar lands

to increase nutrition content. Education sector activities in this program they teach community about proper using available land. Water and mineral sector focus on safe water distribution. Women and children sector behavior change education to empower women economical equipped, and teach women about they have equally power with her husband during decision.

Interviewer: What are the nutrition related activities of your office?

Respondent: Under-five growth monitoring, nutrition screening, Vitamin supplementation, pregnant women nutrition status screening, and supplementary feeding support for malnourished women and children

Interviewer: Would you say more about each nutrition related activity in your office? List of activities? Its aim? Any plan?

Respondent: Yes, there is plan.

Interviewer: What are the roles and responsibilities of your office to achieve the implementation of multi-sectorial of nutrition?

Respondent: Health sector coordinate multi-sector nutrition program. Health sector evaluate performance of six sectors.

Interviewer: Tell me the ownership of the program?

Respondent: District administer lead this program. In each sector there is committee (focal).

Interviewer: detail the coordinator of the program and this body responsibility?

Respondent: Coordinator for this program is under district administer bureau. His responsibility is monitoring performance of this program. And also she/he presents six sectors performances to the district administer.

Interviewer: Tell me the challenges of your office in relation to collaboration with other offices?

Respondent: Food and nutrition activities are multi-sectorial work. Challenges solved through team work. Team work of this program is good. Main challenges for this program shortage of vehicle. Other challenge is budget shortage.

Interviewer: detail about the procedure of program such as; About the annual plan, half year plan, quarterly plan, achievement report, meeting schedule, monitoring and evaluation related commitment.

Respondent: Plan prepared once in year. Performance evaluate frequently. There is schedule for evaluation. Technical committee have meeting monthly this committee lead by health office head. Every three month district administer call six sector head and focal for meeting.

Interviewer: What challenges are there for the community to achieve a multi-sectorial nutrition program?

Respondent: Communities are ready for change. Our communities have experience of similar seed grain farm. But there is improvement.

Interviewer: the commitment of the community, for any resistance from the community?

Respondent: Still now community accepts this program. We provided different type of education to change community attitude. Now they farm different type of seed grain for one land.

Interviewer: Tell me how the structure of this program is organized?

Respondent: There is district administer, six sectors head and focal from six sectors. This program was given due political considerations. At community level there is kebele leaders, health extension workers, agriculture professionals and education director at community level.

Interviewer: the reporting system of the program?

Respondent: Six sectors combine reports and complied report present to district administer. Then evaluated report sent to the region.

Interviewer: Say something about the program in relation to the budget?

Respondent: There is shortage of budget. Even if inadequate budget community supported by this program. For example, when public pipe constructed in one village whole communities in this village have safe water access.

Interviewer: the financial admin, adequacy or shortage, and others?

Respondent: Budget supported by three bodies. District, region and federal government support this program. Budget administered by each sector head. Evaluate the budget utilization according to the plan.

Interviewer: HR and other resource issues in your office?

Respondent: Human resource is not adequate.

Interviewer: How did the professionals who work on a multi-sectorial nutrition program capacitate?

Respondent: Training provided by region for this program but didn’t enough training to capacitate professionals who work in this program.

Interviewer: Have you had the consultant workshop on this program?

Respondent: Consultative workshop facilitate by region. Last year consultative workshop prepared in the Shebedino district for experience sharing.

Interviewer: if yes, details like who were participants?

Respondent: From each district program focal and district administer participated. And also from region six sectors participated.

Interviewer: How you involve the community to create awareness?

Respondent: For awareness creation Kebele administer, health extension workers, school director, and agriculture extension workers participated when this program launched.

Interviewer: What are the nutrition related programs other than multi-sectorial of your office?

Respondent: Health extension workers at community level screen nutrition status of pregnant women and under-five children, Iron folate supplementation, and education on family planning and ANC service.

Interviewer: Is there designated responsible body to coordinate the program?

If yes, how she/he is committed?

Respondent: Yes, there is responsible body. He is committed for his work. Health office assign nutrition focal person to coordinate multi-sectorial nutrition program with responsibility.

Interviewer: What are strategic and operational plans of your office in multi-sectorial nutrition program?

Respondent: Yes, there is strategic plan. Strategic plan goal is to decrease under-two year children stunting to zero level at 2030 G.C. This plan operationalized for one year plan.

Interviewer: How community are committed to support the activity plan of this program

Respondent: Community supportive for this program. They are committed to work in collaboration with us.

Interviewer: Tell the presence of promising work structure of this program ?

Respondent: Promising structure is availability of committed district administer and region support. Community accept nutrition program as their concern. Community currently farm different seed grain in their farming area.

Interviewer: How is the political support of this program?

Respondent: Political is good in the current situation. Regional administers and district administers give due attention for this program. They consider community problem as their problem.

Interviewer: What do you think on the recommended strategy to improve the implementation of multi-sectorial nutrition program in your district/region?

Respondent: I recommend resource support should be increased. There are many problems at community level, so that vehicle problem should be solved. At office level there is shortage of material to support this program like material used for documentation.

Interviewer: How do you think these strategies can improve the multi-sectorial nutrition program?

Respondent: Community should be checked provided education brings change on knowledge and does they apply what we teach them.

Interviewer: Now that I'm almost done with my questions, you can express any concerns you have about this program?

Respondent: At district level federal government supported budget didn't utilized effectively. The budget utilization problems solved by district administer. Even if there is budget shortage, selected households for this program they receive three goats. This initiative has important effect on the community. Last year constructed public pipe is another good thing for community to have safe water access.

Interviewer: Thank you very much for your time and genuine response!

Respondent: I am happy by this program. Thank you too.

Interview VIII: IDI with 04 district HNF

District office name: 04

Sex: Male

Age: 35 years

Marital status: Married

Professions: Bsc in baoratory technician and Master's degree human nutrition.

Position: HNF

Work experience: 2 years and 6 months experience on current position

Interviewer: Tell me about the nutritional problem in your region/district/locality?

Respondent: In our district due rain shortage nutrition problem prevalent. There are 25 kebeles (23 rural and 2 urban kebeles) in the district. Nutrition problem frequently affect rural kebeles. Now a day we work in collaboration with agriculture sector to mitigate this problem. In Sidama

region 10 districts selected for Sekota declaration. Our district one of among selected districts for this program.

Interviewer: How do you see the nature of the nutritional problem in the local context?

Respondent: Last year 2014 E.C there were shortage of rain and many people affected by nutrition problem. Relatively in this year there is improvement. The nutrition problem depends on availability of rain.

Interviewer: How this problem trend looks from past to the present?

Respondent: There is fluctuation of nutrition problem because of agriculture product in this area depends on availability of rain. In our district nutrition problem increases in number March up to May month.

Interviewer: Have you heard about the multi-sectorial nutrition program?

Respondent: Yes, I heard. Multi-sectorial nutrition program launched in 2014 E.C. This program related with Sekota declaration.

Interviewer: If yes, would you say something about its policy, principles, and implementation?

Respondent: This program strategy is food and nutrition. Six sectors participated in this program. These sectors have monthly meeting to evaluate the work done within month. Finally, after discussion report written in the logbook before sent to region. There is technically committee at kebele level. Kebele administer is head and HEW is secretarial for this committee.

Interviewer: What are the nutrition related activities of your office?

Respondent: Iron folate supplementation, nutrition education in the school, and food demonstration for pregnant women program.

Interviewer: What are the roles and responsibilities of your office to achieve the implementation of multi-sectorial of nutrition?

Respondent: Health sector role behavior change education to the community.

Interviewer: Tell me the ownership of the program?

Respondent: As I told you before health sector takes big responsibility in this program. Because, nutrition, and hygiene and sanitation is important thing for community without health sector involvement it is difficult to achieve programs goal.

Interviewer: detail the coordinator of the program and this body responsibility?

Respondent: Coordinator for this program if health office head. His role in this position monitoring and evaluation of program performance.

Interviewer: Tell me how the structure of this program is organized?

Respondent: Six sectors in this program interlinked. There are technical and Steering committee. Techinical committee lead by health office head and steering committee lead by district administer head. This structure reaches community through kebele administers and HEWs. Reporting system for this program starts from the kebele technical committee. Then, reported to district technical committee. Finally, technical committee presents findings to the steering committee.

Interviewer: Say something about the program in relation to the budget?

Respondent: Multi-sectorial nutrition program is correlated with Sekota declaration. No budget allocated separately for this program, but Sekota declaration allocated budget utilized for this program. Federal government allocated around 2 million Ethiopian birr. Allocated budget distributed for six sectors.

Interviewer: the financial admin, adequacy or shortage, and others

Respondent: It is difficult to say adequate budget. Because of there are work burden. Each sector administer own distributed budget. At district level totally budget run by district finance sector. So that we request finance sector to give us according to action plan developed.

Interviewer: HR and other resource issues in your office?

Respondent: Human resource is adequate in health sector. There are one primary hospital and four health centers. Health professionals and supporter staffs are adequate.

Interviewer: How did the professionals who work on a multi-sectorial nutrition program capacitate?

Respondent: Well, for multi-sectorial nutrition program there is one focal in each sector. Since program launched training provided for those focal. In addition, this year they take training. Community leader, religious leader, health extension workers, and HDA were toke training.

Interviewer: Have you had the consultant workshop on this program?

Respondent: There is no consultative workshop. We planned community lab, and we selected two villages for scaling to model kebeles. We give due attention to reduce stunting to zero level. This community lab used for practical demonstration to show 20 selected HHs practical demonstration.

Interviewer: How you involve the community to create awareness?

Respondent: We provide training for community leaders, voluntary people, religious leader, and HDA. Training provided to create awareness on the program and to reduce stunting.

Interviewer: What are the nutrition related programs other than multi-sectorial of your office?

Respondent: As I told you before we give nutritional support for under-five children and pregnant women. We give education on exclusive breast feeding, when complimentary feeding started and for two years breast feeding continued. We advise them local prepared dietary diversity. Health extension workers work on community based nutrition (CBN), growth monitoring, and counsel women's on nutrition. We have plan based on total population. We have plan starting immunization.

Interviewer: Is there designated responsible body to coordinate the program?

If yes, how she/he is committed?

Respondent: Yes, there is responsible body. He is responsible for all nutrition activities. And also accountable for all performance of this program. He is committed.

Interviewer: What are strategic and operational plans of your office in multi-sectorial nutrition program?

Respondent: Yes, there is strategic plan. Strategic plan sent from federal government. For all activities action plan developed.

Interviewer: How community are committed to support the activity plan of this program

Respondent: When we provided training for community representatives, we informed to share whole community what they trained. In addition Health Development Army (HDA) has responsibility to create awareness to whole community in each village. In each village there are around 25 HDA.

Interviewer: Tell the presence of promising work structure of this program?

Respondent: Availability of trained community representative play important role for continuity of the program.

Interviewer: How is the political support of this program?

Respondent: As owner these program leads by district administer. There is steering and technically committee. Steering committee performances evaluated by district administer head. Administer monitor the programs.

Interviewer: Ask in detail about the procedure of program such as; About the annual plan, half year plan, quarterly plan, achievement report, meeting schedule, monitoring and evaluation related commitment

Respondent: Firstly there is plan. We evaluated 2014 performance. We monitor and evaluate the program in collaboration with other sectors. Then prepare review meeting from each kebele five community representatives participate meeting. All participants gave their idea for the presented report.

Interviewer: What do you think on the recommended strategy to improve the implementation of multi-sectorial nutrition program in your district/region?

Respondent: The first thing nutrition education and cooking demonstration should be strengthening. HEWs should strengthen the performance and commitment. Other thing budget shortage should be solved. Vehicle problem solved to strictly monitor work at community level. Finance sector should be cooperative when we ask money for program activity. They delay money request this have negative impact on the work.

Interviewer: How do you think these strategies can improve the multi-sectorial nutrition program?

Respondent: All thing related with budget for example when we call community for training they expect money for transportation. Budget is mandatory to buy stationary. Other thing for demonstration purpose money is necessary. To reach hard to reach area and to support lower level community budget is important.

Interviewer: Now that I'm almost done with my questions, you can express any concerns you have about this program?

Respondent: From totally 23 kebeles 8 kebeles have malnutrition problem. The topography of these kebele is good for farming and frequently moderate acute malnutrition reported. For those with MAM (Moderate Acute Malnutrition) agriculture give nutritional support. Those with Severe Acute Malnutrition (SAM) treated in outpatient therapeutic program (OTP) in the health post we face nutritional support shortage currently. Policital support should be strengthen for this program.

Interviewer: Thank you very much for your time and genuine response!

Respondent: Thank you too.
