# Supplementary material for: Exploring the challenges and opportunities of multisectoral nutrition programme in Ethiopia: A qualitative study on combating undernutrition during pregnancy
Source: PLoS One. 2025 Jul 3;20(7):e0311336. doi: 10.1371/journal.pone.0311336 (PMC12225801; doi:10.1371/journal.pone.0311336)
Supplement: S5 Data — (PDF) [file pone.0311336.s006.pdf]

Interview 1 - 02 Woreda w01

Woreda/district/partner/region office name: 02
Age,marital status,professions,postion and work experiences
I am a 29-year-old, married, water supply and Environmental Engineer by profession, w02 and I have 6 years work experiences.

Section 2: Multi-sectorial nutrition program implementation related challenges in this office/partner
Theme 1: Challenges in understanding the program

8 (I) Tell me about the nutritional problem in your region/district/locality?
(D) In the our district for the past 3 years continuously there was drought and most of cereals damaged due to climate change and drought. Because of this our community especially those who are under 2 years getting “Menkecher” which means stunting

Even if ,there was nutritional problem around our district, we support by safety net program those who suffering by scarcity food .In the our district most of time nutritional problem rises those driest kebeles among total kebeles especially

Barto ,Rude , kutayou , Argada and Alidabo are risky areas because driest kebeles and those community comparatively, only produce and cultivate cereals group like maize and haricot beans rather than other crops due to lack of rain. Those people who produce and cultivate false banana “Enset” not suffering in nutritional problem in the loka abayya district because ,false banana can resist doughtiness, but those community who produce and cultivate cereals group and pastoralists are suffering from nutritional problems in many aspects. And also there is no ground water even to produce and cultivate irrigation in the our district and currently Awada -Boricha water project supplies drinking water unfortunately its temperature is 40 degree Celsius because of this reason closed drinking water projects and no other water resources both ground and under ground water.

9 (I) Have you heard about the multi-sectorial nutrition program?
(D) Yes, I heard after declaration of “sekota” multi-sectorial nutrition program there were coordination and integration regardless of sector as a steering and technical committee by collaboration of multi-sector there is nutritional program which led by woreda administration and each sector plan activity evaluated in 90 days/(3) months/6 months as well as woreda admiration punishes those who had weak performance

based on its plan. This is for prevention of stunting for those who under two children and suffering from nutritional problem those who pregnant and lactating women in the district.. we collaborate with different office like woreda health ,agriculture, women's, children and youth office working for common goals for prevention of stunting for under five children and supporting pregnant and lactating women's in order to accomplish the targets.

Each sector has its own work plan, for example agricultural office avails hybrid maize seeds, fertilizers and prepares for crop lands. We water and mines sector initiate and access to small-and large scale irrigation systems to tackle nutritional problems those for vulnerable groups as well as increase access to safe water and provide water supply for sewerage services .All multi-sectors leads and evaluated by worada administer in order to make strengthening multi-sector collaboration and integration. Woreda administer evaluates accordingly the plan of each sector.

10(I) How this problem trend looks from past to the present?

(D) Our woreda is low-land area (''kola'') /scarcity of water resources due to this ,the most of years there is nutritional problem because of the climate change. According to GTP2 plan with in 1 k.m interval there is should be avail pond to mitigate nutritional problem accordingly and mostly drought affected kebeles are Abaya gangawa, bukito bura and chiracha behind the loka abaya national park for these areas we are planned to construct water points by help of sidama regional states in order to avail water resources . We can't afford because of inflation of the cost specially for the building materials. Those community who mostly suffer from nutritional problem we support by integration an collaboration NGOS like safety net and calm projects for rearing domestic animals. Generally, nutritional problem is increasing day to day due to climate change.

11 (I) What are the nutrition related activities of your office?

(D) Our office couldn't avail accordingly to GTP2 plan because it needs huge amount resources/needs high capital according to the standards. And also our woreda is low land, which increases vulnerability of risks and for the 3 years continuously lack of rain in our district. But in this year, we have rain may be those faced problems will mitigate by estimation due to seasonal rainfall.

Our sector responsibility is availing water for relation to protect nutritional problem for irrigation purposes and accessing safe water for drinking and also purifying, filtering (by using tulip)and treating the availed water. Not only district level but also we avail dug well and pond in the household level for irrigation in order to protect nutritional problems by planting vegetables, accessing water for their domestic animals. Our office provides education based on environmental sanitation ,hygiene and sewerage purposes. Loka abaya woreda water ,mines office aimed to avail and accessing water sources and

planned to overcome from nutritional problems for those under two years children and pregnant and lactating women.

12(I) What are the roles and responsibilities of your office to achieve the Implementation of multi-sectorial of nutrition?

(D) We encourage the community in order to prepare hand dug wells and ponds in order to defeat food shortage at the household level by integrating agricultural office and we provide tulip and water purifiers because our woreda has no other water sources.

We select very poor community and creating coordination all stake holders procuring, distributing and using nutritional supplies through an integrated logistics management that may help the improvements of implementation regardless of sectors even in the household level.

13(I) Tell me the ownership of the program
(D) According to multi-sectorial nutritional program each has responsibility and all sectors are stakeholders for decision making and to overcome nutritional problems at house hold level rather than individuals and targeting prosperity at the house hold level for improving nutritional status. All multi-sectors work hand in hands by integrating and collaborating to tackle nutritional problems without assigning individual tasks. To summarize, all multi-sectors for this program ownerships regardless of sectors accordingly the program even if from selecting house hold and agricultural office avail hybrid maize seeds and fertilizers.

Thank you very much dear!

14(I)Tell me the challenges of your office in relation to collaboration with other offices.
(D) string committee evaluated based on time based on the plan…

Our challenges are water treatments like ‘’purifiers’’ ‘wuha agar’’due to inflation of costs and logistics for building materials for irrigation purposes. According to our plan we evaluate twice in month and finalize the report in three month report without interruptions of the program by participating worada administer and councils of worada. All multi-sectors have focal person for sekota program which leads by worada health office, this makes effective for integration and collaborations to tackle challenges.

15 (I) What challenges are there for the community to achieve a multi-sectorial

nutrition program?
Probe: ask for the commitment of the community, for any resistance from the community.

(D) During selection those very poor community are main challenges to select mr x but ,currently after we selected the poor people again we raised in front of those community in the assembly point at kebeles level

In the past years, there were challenges in order to select very poor community and including data base management. Currently no challenges arise due to supervisions of stake holders with in time. But, all community are committed to achieve multi-sectorial nutritional program without any resistance.

16 (I) Tell me how the structure of this program is organized?
(D) Sekota **multi-sectorial** nutrition program has its own string committee in the kebeles and collaborated with hygiene and sanitation as well as district level accordingly ,which leads by kebeles chairperson that makes organized achievement from top-down stakeholders and weekly and monthly report under gone from low-to-high levels of responsibilities of multi-sectorial accordingly.

17(I) Say something about the program in relation to the budget?
(D) Under sekota multi-sectorial nutritional program targets many aspects at the house hold level but due to scarcity of budget we couldn't achieve regardless of our plan. Unfortunately, inflation costs for all items increasing time through the time. During declaration of multi-sectorial sekota nutritional program there were no baseline assessment and cost analysis benefit due to this, we suffering for scarcity of budget in order to achieve all commodities for water, sanitation and hygiene purposes including sewerage systems. Generally, there is huge scarcity of budget in the woreda and regional level. So, this may increases vulnerability of nutritional program even at house hold levels including not accessing safe water for drinking and washing purposes for especially, pregnant, lactating mother and those who are under two children. This increases the burden of infectious disease in the community level.

18(I) HR and other resource issues in your office?

(D) We have enough skilled human resource, rather than scarcity of budget we have skilled man power in the our sector and there is always fiscal budget scarcity through the year

19 (I) How did the professionals who work on a multi-sectorial nutrition program capacitate?
(D) Created deep rooted awareness for the community and all stakeholders should be updated on time that makes to build capacity for multi-sectorial nutritional program in the district. Integrating, collaborating and assigning sector based focal person responsibility to make capacitate in order to access nutritional improvement in the community level.

20 (I) Have you had the consultant workshop on this program?
(D) Participating by community sanitation ,health extension workers and leaded and arranged by worada health offices , work shop had gone on including all stakeholders of multi-sectorial offices and partners one wash program and sekota mullti-sectorial nutritional program focal person from each sector in the worada level which leaded by worada administer.

21 (I) How you involve the community to create awareness?

(D) We involved the community after creating awareness by using local language through the radio by their kebele, religion and neighbor leaders and creating awareness how to feed different types of feed for under two children, pregnant and lactating women Even not only sekota multi-sectorial nutrition program but also the prime minister of Ethiopia has launched

" limat tirufat" which helps to secure good nutritional status for all community including under two children, pregnant and lactating women in the community.

22(I) What are the nutrition related programs other than multi-sectorial of your office?
(D) AS I have mentioned , before we avail water which means safe drinking water and protecting environmental sanitation and also availing water for irrigation purposes which helps the community could produce and cultivate without rainy based agriculture. we purify and treat water ,provide health education based on sanitation ,hygiene by assigning focal person in order to keep deep rooted sustained community without nutritional program in the community. Evaluated weekly and supervised by our office based on the plan in order to keep healthy community. we overcome stunting for the under two children from nutritional problem is our plan including to tackle nutritional problem from pregnant and lactating women in the community.

23 (I) s there designated responsible body to coordinate the program?

If yes, how she/he is committed?

(D) we should avail safe water and providing irrigation systems in the community is our daily work activities to tackle nutritional problems in the household level and preventing water based disease by treating water sources and collaborating and integrating with the community.

24(I) What are strategic and operational plans of your office in multi-sectorial nutrition program

(D) As I told you before our strategic plan is to build huge projects like dams regarding to irrigation purposes and availing safe water its may take 3/5 or more 5 years ,but our

operational plan are repairing water pump after burst ,treating water, providing health education and creating various potential bottlenecks to achieve developmental goals.

25(I) How community are committed to support the activity plan of this program

(D) For availing water and supporting nutritional programs, community are very committed to support by any aspects.

26(I) tell the presence of promising work structure of this program

(D) Before 3 years back ,those who pregnant and lactating mother don't awarded how to feed different kinds of food ,But currently , after sekota multi-sectorial nutritional program launched :community are ready and practice such programs and know the importance of integrations and collaborations of multi-sectorial nutritional programs rather than giving for single sector. Generally, all community awarded ,ready and integrated for the multi-sectorial nutritional programs.

27(I) How is the political support of this program:

(D) In our district political support are committed for any aspects and leaded by woreda administer.

28(I) What do you think on the recommended strategy to improve the implementation of multi-sectorial nutrition program in your district/region?
(D) My recommendations are increasing budget , base line assessment and providing cost based analysis for water related logistics supplies in the district in order to improve and implement for the multi-sector nutritional program.

29(I) How do you think these strategies can improve the multi-sectorial nutrition program?

(D) Those prevention of stunting on under five years and pregnant mothers and lactacting should provide education through demonstration how to feed, at what frequency they should feed their cheir children and for them….

In order to improve this challenges should increase annually budget for example in this we got 2500 Ethiopian birr in order to buy water purifiers, ha haaa(lough)

Creating awareness for the community that the importance of multi-sectorial nutritional program, increasing annually budget for only sekota multi-sectorial nutritional program and availing water for irrigation to sustain nutritional program in the house hold level.

Thank you very much I completed here!

Interview II: 02 woreda WNF

Interviewer: I Discussant :D

(I) Woreda/district/partner/region office name: Age ,marital status ,professions, position and work experiences.
(D) 02 Woreda,Sidama National Regional state, firstly my name is B , I am 29 years old, single, Construction Engineer by profession, construction W001 person for multi-sectorial nutritional program water and mines office and I have a 11 year work experiences.

Section 2: Multi-sectorial nutrition program implementation related challenges in this office/partner

Theme 1: Challenges in understanding the program

(I) Tell me about the nutritional problem in your region/district/locality?
(D) Currently we are facing nutritional programs due to shortage of budget and shortage supply like ”Aja” means oats for those malnutritined community due to lack of rainy for the past two consecutive years because our woreda depends on rainy and no other water resources. Before those food in secured community get food items like “Fafa” Aja” from the woreda health office which from non-governmental organizations aids for only pregnant, lactating women and those under two children.

In the our district there are many nutritional problems those for under two children ,pregnant and lactating women because of lack of rain(drought) for the three consecutive years associated with low land areas(‘’kola’’) like danshe gambella,falka bukito argada haro dimitu and argada are mostly affected kebeles in the district. In our district there is no ground water in order to improve nutritional problems by supporting irrigations.

Thank you !

8 (I) Have you heard about the multi-sectorial nutrition program?
(D) yes, at right now ! we work by’’ team sprit and integrated and collaborated by multi-sector nutritional programs(woreda health, agriculture sectors) creating string committee to improve nutritional program deceleration and giving responsibility focal person from each sector but before we are ”Bilachawa” meaning separated by sectors currently we are integrating and collaborating for common goals in order to tackle from nutritional problems.

9(I) How this problem trend looks from past to the present?

(D) In our district about 45% of food supplies are from international aids/ NGOS, due to climate change in our district there were many morbidity and death under 2 years children , pregnant and lactating mother from the nutritional problem and due drought we are struggling from nutritional program in generally ,in our district nutritional problem

is increasing through year to year and lack of balanced diet for pregnant, lactating mother and those under two children because of the drought.

Thank you very much!

10(I) What are the nutrition related activities of your office?
(D) We integrating with woreda agricultural sectors availing community ponds for irrigation purpose and should avail safe drinking water ,irrigation purposes and providing health education regarding to sanitation and treating water within one month duration in order to improve nutritional related activities in the district to tackle the burden of nutritional problems as well as we should evaluate on time and separate our strength and weakens for the our plan.

11(I) What are the roles and responsibilities of your office to achieve the Implementation of multi-sectorial of nutrition?

(D) All multi-sectors nutritional stake holders should work in team spirit and we design the projects and avail water resource ,treating water ,providing health education regarding to sanitation and constructing water wells in the district for irrigation purposes to overcome nutritional problems in order to achievement and implementation in the household level and from health sector establishing the VIP latrine ,also we avail building for treating patients in the health centers and health posts .In this kebele like abaya zuriya we give /'tulip/bishan gar" supported those scarcity of water but for availed water we give ''wuha agar'' and chlorine from sidama regional state and each person should 20 liters per day according to GTP2.

12(I) Tell me the ownership of the program
(D) worada health office is leads the program and all multi-sectors stake holders take responsibility by own their plan and each sector has the focal person to run this multi-sector program to implement in the district and house hold level which coordinated woreda health office and leading by worada admiration bodies. **Woreda health office leads the program and writers for the all sectors. To summarize, all multi-sectors for this program ownership regardless of sectors.**

13(I) Tell me the challenges of your office in relation to collaboration with other

offices.
About the annual plan, half year plan, quarterly plan, achievement report, meeting schedule, monitoring and evaluation related commitment

(D) In our district there is challenge how to plan based on the professionals and coordinating by multi-sectors giving responsibility for each sector based on the their professional tasks. we are evaluating and monitoring term by term based on schedules in order to achieve the program.

14(I) What challenges are there for the community to achieve a multi-sectorial nutrition program?
(D) Not getting integration and collaboration all stake holders in the district level could be our challenge .In our district the community are committed to achieve a multi-sectorial nutritional program and lack of ground and underground water resources. Our community are committed for any integrations.

15(I) Tell me how the structure of this program is organized?
(D) After creating awareness for the community, we select 2 kebeles in order to implement this multi-sectorial nutritional program based on the plan. **multi-sectorial nutrition** program based on its own string committee in the kebeles and collaborated with hygiene and sanitation as well as district level by evaluating and monitoring through the time.

16(I) Say something about the program in relation to the budget?
(D) Under multi-sectorial nutritional program targets many aspects at the house hold level but due to scarcity of budget we couldn't achieve regardless of our plan due to inflation of costs and no cost based analysis done before implementation on multi-sectorial nutritional program. Generally, we are suffering of shortage of budget to implement multi-sectorial nutritional program.

17(I) HR and other resource issues in your office?

(D) We have enough skilled human resource, rather than scarcity of budget we have skilled man power in the our sector and there is always fiscal budget scarcity through the year. We only report on hard copy and rather than soft copy that means scarcity computers. (logistics). Generally, we have shortage of logistic supplies in our office in order to improve multi-sectorial nutritional program.

Thank you very much !

18(I) How did the professionals who work on a multi-sectorial nutrition program Capacitate?
(D) Integration and collaboration different targeted hand-in hands together in order to build capacity to access safe water ,providing health education based on personal hygiene and sanitation and supporting irrigation systems for addressing under two years children, pregnant and lactating women by multi-sectorial nutrition program.

19(I) Have you had the consultant workshop on this program?

(D) Yes, workshop was arranged regional health bureau collaborating multi-sectorial nutritional program stake holders. Participating by community sanitation, health extension workers and partners one wash program and multi-sectorial nutritional

program focal person from each sector in the worada level which leaded by regional prosperity party head.

20(I) How you involve the community to create awareness?

(D) Creating awareness ,participating community , demonstrating the program by using local languages . We stake holders are create awareness on how to feed and how avoid junk foods to be effective on multi-sectorial nutritional program especially addressing under two children and pregnant and lactating women for the selected households.

21(I) What are the nutrition related programs other than multi-sectorial of your office?

(D) we avail water which means safe drinking water and protecting environmental sanitation and also availing water for irrigation purposes which helps the community could produce and cultivate without rainy based agriculture/not being dependable on rainy through the year can produce crops .Evaluated weekly and supervised by our office based on the plan in order to achieve accordingly the plan.

22(I) Is there designated responsible body to coordinate the program?
If yes, how she/he is committed?

(D) Woreda health office coordinates and leads the multi-sectorial nutritional programs and having focal person in order to run multi-sectorial program through transparency and accountability by woreda administer body.

23(I) what are strategic and operational plans of your office in multi-sectorial Nutrition program

(D) Treating, purifying water, providing health education on environmental, personal hygiene by addressing primary school students in order to reach community and maintaining non-functional water pipes.(operational plan)

Our strategic plan is to construct projects like dams regarding to irrigation purposes and availing safe water that may take 3/5 or more 5 years ,but our operational plan are repairing water pump after burst ,treating water, providing health education and creating various potential bottlenecks to achieve developmental goals.

24(I) How community are committed to support the activity plan of this program

(D) For availing water and supporting nutritional programs, communities are very committed to support by any aspects. we call stake holders like religion and cultural leaders to announce ownership of the program and encouraged them through the meetings.

25(I) Tell the presence of promising work structure of this program

(D) In the past years, those who pregnant and lactating mother don't know about how to feed different kinds of food only they feed their children and themselves in traditional ways, But currently, multi-sectorial nutritional program launched :community are ready and practice such programs and know the importance of integrations and collaborations of multi-sectorial nutritional programs rather than giving for single sector. Generally, all community awarded ready and integrated for the multi-sectorial nutritional programs.

27(I) How is the political support of this program:

(D) For any means of our woreda ,which supports multi-sectorial nutritional program .Our district political management support are committed for any aspects led by worada administration or worada prosperity party which shows a good political commitment based on improving multi-sectorial nutritional program and evaluated based on the program and also encouraging and empowering the program by political management body.

28 (I) What do you think on the recommended strategy to improve the implementation of multi-sectorial nutrition program in your district/region?
(D) Making deep rooted collaborations with non-governmental organizations like safe net program by providing domestic animals in order economy building capacity at the house hold level and encouraging saving for the very poor community.

On my ''agenda'' in our woreda, very poor community is not getting support from non-governmental organizations aids because of most of governmental employers are getting support through the illegal way which should be evaluated by higher level officials.My recommendations are increasing budget , base line assessment and providing cost based analysis for water related logistics supplies in the district in order to improve and implement for the multi-sector nutritional program through collaboration and integrating in order to preventing stunting and maternal death.

29(I) How do you think these strategies can improve the multi-sectorial nutrition program?

(D) Collaboration and integration are basic way for multi-sectorial nutritional program improvement. Creating awareness ,participating the community and importance of multi-sectorial nutritional program, increasing annually budget and availing water for irrigation to sustain nutritional program in the house hold level in order to improve child and maternal health .

Thank you very much I completed here.

Interview III: , with 01 woreda W01

(I) Woreda/district/partner/region office name: Age ,marital status ,professions, position and work experiences.
(D) 01 Woreda,Sidama National Regional state , I am 35 years old, married, water supply and Environmental Engineer by profession,   for multi-sectorial nutritional program water and mines office represented person   and I have a 15 year work experiences.

Section 2: Multi-sectorial nutrition program implementation related challenges in this office/partner

Theme 1: Challenges in understanding the program

8 (I) Tell me about the nutritional problem in your region/district/locality?
(D) Among Sidama regional state, our district is known low-land district (‘’kolla’’) this increases climate change and shortage of water thoroughly the year that leads nutritional problem. Those for under two children pregnant and lactating women because of lack of rain (drought) for the three consecutive years and absence of rain water resources in the district for irrigation purposes. .Highly risky kebeles for food insecurity(nutritional program) is known as’’ tereje kebele” are korangoge, aldada dela,hanja chafa,konsore chafa ,fulasa aldada and bonoya chire. There is no “Amareche” means no option because of in our district there is no ground (rivers/spring) and underground water(rivers/ in order to improve nutritional problems by supporting irrigations .We get water from awada-borricha water project and no other points in the district.Our community depends on seasonal rain.To sum up, we always have thoroughly the year nutritional problem in the Boricha district.

9 (I) Have you heard about the multi-sectorial nutrition program?
(D) yes, woreda health ,agriculture, water and mines ,women’s children and youth office ……….(7 different offices) integrated together and collaborated by multi-sector nutritional programs creating string committee which led by policy makers , woreda administration body to improve nutritional program and giving responsibility focal person from each sector. we provide safe water for drinking and sanitation purposes and repairing non-functional water pumps through daily actives regardless of other site water resource ; Awada-boricha water projects(from yirgalem- Awada site) .

10(I) How this problem trend looks from past to the present?

(D) Because of, climate change in our district there were many morbidity and death under 2 years children , pregnant and lactating mother from the nutritional problem. Generally in our district nutritional problem is increasing through the time because of the drought and we haven’t water resources in the district another drawbacks thoroughly years and in the our district there are’’ densely populated’’ even in the sidama national state due to this ,” lup” means increases ‘nutritional problem as trend shows. Before our

woreda depends on non-governmental organizations aids. Most of people are begging on the street due to food shortage in the district including under two years children and pregnant and lactating women .That may worsens partners are not supporting as usually currently in the district.

11(I) What are the nutrition related activities of your office?
(D) Even if as our office strategy has written proposal we have not accesses water resources. we planned in order to get safe water regardless of plan in two water points we got high concentration of fluoride and iron due to this we are suffering from these problems. We couldn't access irrigation purposes due to lack of water resources this may leads dependable on rainy for crop cultivation production. And providing health education regarding to sanitation and treating water within one month duration in order to improve nutritional related activities in the district to tackle the burden of nutritional problems to getting safe drinking water in the district.

12(I) What are the roles and responsibilities of your office to achieve the

Implementation of multi-sectorial of nutrition?

(D) All multi-sectors nutritional stake holders should work in team spirit and we avail water resource with interruptions ,treating water ,providing health education regarding to sanitation and constructing water wells. Sustained safe water in the district to overcome nutritional related health problems in order to achieve and implement good nutritional status in the household level.

13(I) Tell me the ownership of the program
(D) worada health office is leads the program and all multi-sectors stake holders take responsibility by own their plan and each sector has the focal person to run this multi-sector program to implement in the district and house hold level which coordinated woreda health office and leading by worada admiration bodies. Each sector focal person leads the program and string committee assures or accomplishment of the program which led by woreda administration body if any problem in the side of shortage of finances. **To generalizes, all multi-sectors for this program ownership of the program irrespective of sectors.**

14 (I) Tell me the challenges of your office in relation to collaboration with other offices.
(D) In our district there are challenge like motor-bikes for transportation to reach house hold level and scarcity of budget for logistics supplies for construction due to inflation of costs and shortage of finance systems .we are evaluating and monitoring time to time based on schedules in order to achieve the program.

15(I) what challenges are there for the community to achieve a multi-sectorial

Nutrition program?
Probe: ask for the commitment of the community, for any resistance from the Community.

(D) Issues of water is in our district is serious problem. Not getting integration and collaboration all stake holders in the district level and shortage of regional budget could be our challenge .In our district the community are committed to achieve a multi-sectorial nutritional program. To achieve our plan even 50% we need enough budget in order to improve the multi-sectorial nutritional program.

16(I) tell me how the structure of this program is organized?
(D) Firstly, we do assessment in the district and creating awareness for the community, we select 2 kebeles in order to implement this multi-sectorial nutritional program based on the plan. **Multi-sectorial nutrition** program based on its own string committee in the kebeles and collaborated with hygiene and sanitation as well as district level by evaluating and monitoring through the time.

17 Say something about the program in relation to the budget?
Probe: ask for the financial admin, adequacy or shortage, and others

(D) For multi-sectorial nutritional program targets many aspects at the house hold level , we got from federal budget government but due to scarcity of budget we couldn't achieve regardless of our plan due to inflation of costs and no cost based analysis done before implementation on multi-sectorial nutritional program. Generally we are suffering of shortage of budget to implement multi-sectorial nutritional program.

18 (I) HR and other resource issues in your office?

(D) We have focal person based on water based water professional and enough skilled human resource, rather than scarcity of budget we have skilled man power in the sector and there is always fiscal budget scarcity through the year. Lack of trained personnel like'' plumper'' in the district. Generally, we have shortage of logistic supplies in our office in order to improvement.

19(I) How did the professionals who work on a multi-sectorial nutrition program Capacitate?
(D) Multi-professional also increases encouragement and capacitate the program through multi-disciplinary in its focal person from each sectors. So, Integration and collaboration different targeted hand-in hands together in order to build capacity to access safe water ,providing health education based on personal hygiene and sanitation and for addressing under two years children, pregnant and lactating women by multi-sectorial nutrition program.

20(I) have you had the consultant workshop on this program?

(D) Yes, workshop was arranged regional health bureau by collaborating multi-sectorial nutritional program stake holders. Participating by community sanitation, health extension workers and partners one wash program and multi-sectorial nutritional program focal person from each sector in the woreda level which leaded by regional prosperity party head.

21(I) How you involve the community to create awareness?

(D) We select the site firstly, and all stake holders are create awareness on importance of multi-sectors through multi-disciplinary to be effective on multi-sectorial nutritional program especially addressing under two children and pregnant and lactating women for the selected households.

22(I) What are the nutrition related programs other than multi-sectorial of your office?
(D) we avail water which means safe drinking water and protecting environmental sanitation and repairing the pumps after broke the pump as soon as possible. Evaluated weekly and supervised by our office based on the plan in order to achieve accordingly the plan.

Thank you very much!
23(I) Is there designated responsible body to coordinate the program?
If yes, how she/he is committed?

(D) Worada health office coordinates and leads the multi-sectorial nutritional programs and having focal person in order to run multi-sectorial program through transparency and accountability by worada administer body.

24 What are strategic and operational plans of your office in multi-sectorial Nutrition program

(D) Our operational plan are maintaining the water pump after broke, treating water, proper management of sewerage, providing health education and creating various potential bottlenecks to achieve developmental goals. Our strategic plan is to construct projects in order to access availing safe water that may take 3/5 or more 5 years

25 (I) How community are committed to support the activity plan of this program

(D) For availing water and supporting nutritional programs, communities are very committed to support by any aspects. We are participating stake holders like religion and cultural leaders to announce ownership of the program and encouraged them through the meetings or evaluations.

26(I) tell the presence of promising work structure of this program

(D) In the past years, those who pregnant and lactating mother don't know about how to feed different kinds of food only they feed their children and themselves in traditional ways, But currently, we are following our plan" seriously" multi-sectorial nutritional program launched: community are ready and practice such programs and know the importance of integrations and collaborations of multi-sectorial nutritional programs rather than giving for single sector. As a comment this, multi-sectorial food program targets only 10 households from two kebeles in the districts….so should increase other households rather than small groups. Generally, all community awarded ready and integrated for the multi-sectorial nutritional programs.

27(I) how is the political support of this program:

(D) In our district political support are committed for any aspects and leaded by woreda administer or woreda prosperity party which shows a good political commitment based on improving multi-sectorial nutritional program in order to prevent stunting for under two children and saving lives for the pregnant and lactating women.

28 (I) what do you think on the recommended strategy to improve the Implementation of multi-sectorial nutrition program in your district/region?

(D) My recommendations are increasing budget and increasing woreda based plan, base line assessment and providing cost based analysis for water related logistics supplies in the district in order to improve and implement for the multi-sector nutritional program through collaboration and integrating in order to preventing stunting and maternal death.

29(I) how do you think these strategies can improve the multi-sectorial nutrition Program?

(D) Creating awareness and collaboration to the community that the importance of multi-sectorial nutritional program, increasing annually budget and availing water for irrigation to sustain nutritional program in the house hold level improving child and maternal health.

Thank you very much!

Interview IV: with WNF of 01 woreda

(I) Woreda/district/partner/region office name: Age ,marital status ,professions, position and work experiences.
(D) 01 Woreda, Sidama National Regional state , I am a 24 year old, single, water supply and Environmental Engineer by profession, for multi-sectorial nutritional program water and mines office focal person and I have a 5 year work experiences.

Section 2: Multi-sectorial nutrition program implementation related challenges in this office/partner
Theme 1: Challenges in understanding the program

Thank you very much!

8 (I) Tell me about the nutritional problem in your region/district/locality?
(D) Our district has nutritional problem for past 3 years more than previous time. Before most of partners support our district. Once I visited during ''belg' means autumn season(rainy season in Ethiopia) mostly high risky areas due to lack of water resources rather than rain/rain dependable areas are Koran goge ,hanjachafa ,konsore chafa due to high population growth rate there. Currently, nutritional problem worsens through the time because of climate changes in the country and lack of water sources in the district for irrigation purposes. So, those under two children and pregnant and lactating mother suffering from nutritional problem in the district.

9(I) have you heard about the multi-sectorial nutrition program?
(D) Yes, we integrated and collaborated by multi-sector nutritional programs creating string committee to improve nutritional program and giving responsibility focal person from each sector. High risky areas are konsore chafe,Koran goge ,hanja chafa aldada della .We provide the water purifiers/chlorine as treatments what did get from multi-sectorial nutritional program and accessing safe water for drinking and sanitation purposes and repairing non-functional water pumps through daily actives regardless of other site water source .we treat ,purify and maintain the water pumps on time and encouraging how to use irrigations regardless of water resources as well as we work on environmental sanitation and hygiene.

10(I) How this problem trend looks from past to the present?

(D) Because of, climate change in our district there were many morbidity and death under 2 years children, pregnant and lactating mother from the nutritional problem. Generally in our district nutritional problem is increasing through the time because of the drought and we haven't water resources in the district another drawbacks thoroughly years. And in the our district there are densely populated due to this also increases nutritional problem as trend shows. Most of people are begging on the street due to food shortage in the district including under two years children and pregnant and

lactating women .That may worsens partners are not supporting as usually currently in the district. Before the partners support wheat ,plump net ,oil and food items for those very poor community.

Thank you very much!
11(I) What are the nutrition related activities of your office?
(D) Water is life, we planned regarding to access only safe water regardless of plan in two site we got high concentration of fluoride and iron due to this we are suffering from these problems and providing health education regarding to sanitation and treating water within one month duration in order to improve nutritional related activities in the district to tackle the burden of nutritional problems to getting safe drinking water in the district.

12 What are the roles and responsibilities of your office to achieve the Implementation of multi-sectorial of nutrition?

(D) This multi-sectorial nutrition program targeted on under two children for prevention of stunting and pregnant and lactating women. We have power interruptions due to lack of transformers ,,we encourage the community to cultivate vegetables like cabbage….All multi-sectors nutritional stake holders should work in team spirit and we avail water resource ,treating water ,providing health education regarding to sanitation in the primary schools to reach community in simply way. Sustained safe water in the district to overcome nutritional related health problems in order to achieve and implement good nutritional status in the household level.

13(I) Tell me the ownership of the program
(D) worada health office is leads the program and all multi-sectors stake holders take responsibility by own their plan and each sector has the focal person to run this multi-sector program to implement in the district and house hold level which coordinated worada health office and leading by worada admiration bodies**. we treat water in 15 days.**

14 (I) Tell me the challenges of your office in relation to collaboration with other offices.
(D) Scarcity of budget , inconsistency of report ,transportation to reach house hold level and scarcity of budget for logistics supplies for construction due to inflation of costs and we have water treatments/purifiers due to expired . We are evaluating and monitoring time to time based on schedules in order to achieve the program.

15(I) What challenges are there for the community to achieve a multi-sectorial nutrition program?
(D) The community is not alerted, lack of other water resources and lack of budgets . Integrated and collaborated all stake holders in the district level as well as the community. shortage of regional budget could be our challenge ,lack of water

resources. In our district the community are committed to achieve a multi-sectorial nutritional program .

Thank you very much!

16(I) Tell me how the structure of this program is organized?
(D) we select 2 kebeles in order to implement this multi-sectorial nutritional program based on the plan. **multi-sectorial nutrition** program based on its own string committee in the kebeles and collaborated with hygiene and sanitation as well as district level by evaluating and monitoring through the time

17(I) Say something about the program in relation to the budget?
Probe: ask for the financial admin, adequacy or shortage, and others

(D) For multi-sectorial nutritional program targets many aspects at the house hold level but due to scarcity of budget we couldn't achieve regardless of our plan due to inflation of costs and no cost based analysis done before implementation on multi-sectorial nutritional program and lack of transportations. Generally we are suffering of shortage of budget to implement multi-sectorial nutritional program.

18 (I) HR and other resource issues in your office?

(D) We have focal person based on water based water professional and enough skilled human resource, rather than scarcity of budget we have skilled man power in our sector and there is always fiscal budget scarcity through the year. Lack of trained personnel like plumper in the district. Generally, we have shortage of logistic supplies in our office in order to improvement.

19(I) How did the professionals who work on a multi-sectorial nutrition program Capacitate?
(D) Multi-professional also increases encouragement and capacitate the program through multi-disciplinary. So, Integration and collaboration different targeted hand-in hands together in order to build capacity to access safe water ,providing health education based on personal hygiene and sanitation and for addressing under two years children, pregnant and lactating women by multi-sectorial nutrition program.

20 (I) Have you had the consultant workshop on this program?
(D) Yes, workshop was arranged regional health bureau by collaborating multi-sectorial nutritional program stake holders. Participating by community sanitation, health extension workers and partners one wash program and multi-sectorial nutritional program focal person from each sector in the worada level which leaded by regional prosperity party head.

21(I) How you involve the community to create awareness?

(D) We select the site firstly, and all stake holders are create awareness on importance of multi-sectors through multi-disciplinary to be effective on multi-sectorial nutritional program especially addressing under two children and pregnant and lactating women for the selected households.

22(I) What are the nutrition related programs other than multi-sectorial of your office?

(D) we avail water which means safe drinking water and protecting environmental sanitation and repairing the pumps after broke the pump as soon as possible. Evaluated weekly and supervised by our office based on the plan in order to achieve accordingly the plan.

23 (I) Is there designated responsible body to coordinate the program? If yes, how she/he is committed?

(D) Worada health office coordinates and leads the multi-sectorial nutritional programs and having focal person in order to run multi-sectorial program through transparency and accountability by worada administer body.

Thank you dear!
What are strategic and operational plans of your office in multi-sectorial Nutrition program

(D) Our strategic plan is to construct projects in order to access availing irrigation systems and drinking water safe that may take 5 years, but our operational plan are maintaining water pump after broke, treating water, proper management of sewerage, planting vegetables, providing health education and creating various potential bottlenecks to achieve developmental goals.

24(I) How community are committed to support the activity plan of this program

(D) For availing water and supporting nutritional programs, communities are very committed to support by any aspects. We are participating stake holders like religion and cultural leaders to announce ownership of the program and encouraged them through the meetings.

25(I) tell the presence of promising work structure of this program

(D) In the past years, those who pregnant and lactating mother don’t know about how to feed different kinds of food only they feed their children and themselves in traditional ways, But currently, multi-sectorial nutritional program launched: community are ready and practice such programs and know the importance of integrations and collaborations of multi-sectorial nutritional programs rather than giving for single sector. Generally, all community awarded ready and integrated for the multi-sectorial nutritional programs.

26(I) how is the political support of this program:

(D) In our district political support are committed for any aspects and leaded by woreda administer or woreda prosperity party which shows a good political commitment based on improving multi-sectorial nutritional program in order to prevent stunting for under two children and saving lives for the pregnant and lactating women.

28 (I) what do you think on the recommended strategy to improve the Implementation of multi-sectorial nutrition program in your district/region?
(D) My recommendations are increasing budget and increasing worada based plan, base line assessment and providing cost based analysis for water related logistics supplies in the district in order to improve and implement for the multi-sector nutritional program through collaboration and integrating in order to preventing stunting and maternal death.

29(I) how do you think these strategies can improve the multi-sectorial nutrition Program?

(D) Creating alertness and collaborate with the community that the importance of multi-sectorial nutritional program, increasing annually budget and availing water for irrigation to sustain nutritional program in the house hold level improving child and maternal health .

Thank you very much! I completed here with you.

Interview V: with 03 district W01

(I) Woreda/district/partner/region office name: Age,marital status, professions, postion and work experiences.
(D) 03 Woreda,Sidama National Regional state , my name is GG ,I am a 32 year old, married, water supply and Environmental Engineer by profession, W02 and I have 6 years work experiences.

Section 2: Multi-sectorial nutrition program implementation related challenges in this office/partner
Theme 1: Challenges in understanding the program

8 (I) Tell me about the nutritional problem in your region/district/locality?
(D) Totally, we 19 kebeles on the district, among 19 only two town kebeles are not have nutritional program because they depend on trade and lack of water resources. Those under two children and pregnant and lactating mother suffering from nutritional problem in the district. We have lack of water resources due to this we can't provide irrigation systems in the district, obviously, our woreda is low-land (''kola'') is suspected for drought thoroughly the year.

9(I) have you heard about the multi-sectorial nutrition program?
(D) yes, we integrated and collaborated by Sekota multi-sector nutritional programs creating string committee to improve nutritional program and giving responsibility focal person from each sector. we provide safe water for drinking and sanitation purposes and repairing non-functional water pumps through daily actives regardless of other site water source.

10(I) How this problem trend looks from past to the present?

(D) In the district there are densely populated due to this also increases nutritional problem as trend shows. Most of people are begging on the street due to food shortage in the district including under two years children and pregnant and lactating women and conflict between our district and oromia region also worsens the community and partners are not supporting as usually currently in the district …4 kebeles(Shelo ilancho,shelo balela,abore,bonoya bonkicha) and lack mechanized agricultural mechanism.

Thank you very much!

11(I) what are the nutrition related activities of your office?
(D) Availing water in the primary schools, providing health education on environmental sanitation and personal hygiene based the aim and treating water and by using sand filter and also for prevention stunting for the under two children and proving and accessing for pregnant and lactating women in the district based on multi-sectorial

nutritional programs regardless of different sectors in the district and to tackle the burden of nutritional problems. Improving safe drinking by treating and filtering the water sources in the district.

Thank you very much!

12 What are the roles and responsibilities of your office to achieve the Implementation of multi-sectorial of nutrition?

(D) This multi-sectorial nutrition program targeted on under two children for prevention of stunting and pregnant and lactating women. After selected two kebeles, from there again selected 12 households in order to improve nutritional status. All multi-sectors nutritional stake holders should work in team spirit and we avail water resource ,treating water ,providing health education regarding to sanitation in the primary schools to reach community in simply way. Sustained safe water in the district to overcome nutritional related health problems in order to achieve and implement good nutritional status in the household level.

13(I) tell me the ownership of the program
(D) Multi-sectorial nutritional program targeted on protecting stunting for only those eligible under two years children and pregnant and lactating mother ‘’Gondoru woxeti’’ which means given duties for under two children and pregnant and lactating women in the district. worada health office is leads the program and all multi-sectors stake holders take responsibility by own their plan and each sector has the focal person to run this multi-sector program to implement in the district and house hold level which coordinated worada health office and leading by worada admiration bodies**. Generally all stake holders are owners for this program**

14 (I) Tell me the challenges of your office in relation to collaboration with other offices.
(D) Lack of water resources in the district, electric interruptions ,Scarcity of budget , inconsistency of report ,transportation to reach house hold level and scarcity of budget for logistics supplies for construction due to inflation of costs. To tackle this problem we built pond here in the district regardless of building there are high fluoride and iron concentration and is not safe for drinking purposes. We are evaluating and monitoring time to time based on schedules in order to achieve the program.

15 (I) what challenges are there for the community to achieve a multi-sectorial Nutrition program?
Probe: ask for the commitment of the community, for any resistance from the Community.

(D) No challenges in our district and our community are committed to achieve a multi-sectorial nutritional program.

16(I) tell me how the structure of this program is organized?
(D) We did assessment in the district and creating awareness for the community. This program was arranged by each sector focal person and leaded by woreda health office controlled by woreda administer, **multi-sectorial nutrition** program based on its own string committee in the kebeles and collaborated with hygiene and sanitation as well as district level by evaluating and monitoring through the time

Here is reporting systems by using a software like conventional charts as follows: has 3 colors

Green- for good performance, yellow-for moderate with warning and Red- for below performance/in danger after feed all reports.

(17 I) Say something about the program in relation to the budget?
(D) For multi-sectorial nutritional program targets many aspects at the house hold level but due to scarcity of budget we couldn't achieve regardless of our plan due to inflation of costs and no cost based analysis done before implementation on multi-sectorial nutritional program. Generally   we are suffering of shortage of budget to implement multi-sectorial nutritional program.

18 (I) HR and other resource issues in your office?

(D) We have focal person based on water based water professional and enough skilled human resource, rather than scarcity of budget   we have skilled man power in our sector and there is always fiscal budget scarcity through the year.

19(I) How did the professionals who work on a multi-sectorial nutrition program Capacitate?
(D) Multi-professional also increases encouragement and capacitate the program through multi-disciplinary. So, Integration and collaboration different targeted hand-in hands together in order to build capacity to access safe water ,providing health education based on personal hygiene and sanitation and for addressing under two years children, pregnant and lactating women by multi-sectorial nutrition program.

20(I) have you had the consultant workshop on this program?
(D) Yes, workshop was arranged regional health bureau by collaborating multi-sectorial nutritional program stake holders. Participating by community sanitation, health extension workers and partners one wash program and multi-sectorial nutritional program focal person from each sector in the woreda level which leaded by regional prosperity party head.

21(I)   How you involve the community to create awareness?

(D) We select the site firstly, and all stake holders are create awareness on importance of multi-sectors through multi-disciplinary to be effective on multi-sectorial nutritional program especially addressing under two children and pregnant and lactating women for the selected households. We provide health education regarding to hand washing after usage of toilet and before making food on targeting prevention of infectious disease on under two years children and preventing scarcity of nutritional related problems.

22(I) what are the nutrition related programs other than multi-sectorial of your

(D) We avail water which means safe drinking water and protecting environmental sanitation and repairing the pumps after broke the pump as soon as possible. Evaluated weekly and supervised by our office based on the plan in order to achieve accordingly the plan.

23 (I) is there designated responsible body to coordinate the program?
If yes, how she/he is committed?

(D)Yes, Woreda health office coordinates and leads the multi-sectorial nutritional programs and having focal person in order to run multi-sectorial program through transparency and accountability by woreda administer body.

24 What are strategic and operational plans of your office in multi-sectorial nutrition program

(D) Even if ,we have both strategic and operational plan regardless of ,wter resource is out of district(Awada-boricha water project). Our strategic plan is to construct projects in order to access availing safe water that may take 5 years ,but our operational plan are repairing water pump after broke, treating water, proper management of sewerage , providing health education and creating various potential bottlenecks to achieve developmental goals.

25 (I) How community are committed to support the activity plan of this program

(D) For availing water and supporting nutritional programs, communities are very committed to support by any aspects. We are participating stake holders like religion and cultural leaders to announce ownership of the program and encouraged them through the meetings.

26(I) Tell the presence of promising work structure of this program

(D) In the past years, those who pregnant and lactating mother don't know about how to feed different kinds of food only they feed their children and themselves in traditional ways, But currently, multi-sectorial nutritional program launched:community are ready and practice such programs and know the importance of integrations and

collaborations of multi-sectorial nutritional programs rather than giving for single sector. Generally, all community awarded ready and integrated for the multi-sectorial nutritional programs.

27(I) how is the political support of this program:

(D) In our district political support are committed for any aspects and leaded by woreda administer or woreda prosperity party which shows a good political commitment based on improving multi-sectorial nutritional program in order to prevent stunting for under two children and saving lives for the pregnant and lactating women.

28 (I) what do you think on the recommended strategy to improve the Implementation of multi-sectorial nutrition program in your district/region?
(D) My recommendations are increasing budget and increasing worada based plan, base line assessment and providing cost based analysis for water related logistics supplies in the district in order to improve and implement for the multi-sector nutritional program through collaboration and integrating in order to preventing stunting and maternal death.

29(I) how do you think these strategies can improve the multi-sectorial nutrition Program?

(D) Creating alertness and collaborate with the community that the importance of multi-sectorial nutritional program, increasing annually budget and availing water for irrigation to sustain nutritional program in the house hold level improving child and maternal health .

Thank you very much!

Interview VI: with 03 district NF

Section 1: Socio-demographic characteristics of the participants

S,no Items

1.(I) Woreda/district/partner/region office name:

(D) 03

2.(I)Sex:

(D) male

3(.I) Age;

(D) 26

4(.I) Marital status;

(D) Married

5(.I) Professions;

(D) *hydraulic engineer*

6.(I)Position;

(D) W002

7. (I) Work experiences

(D) 5 years

Section 2: Multi-sectorial nutrition program implementation related challenges in this office/partner

Theme 1: Challenges in understanding the program

8 (I) Tell me about the nutritional problem in your region/district/locality?

(D) Only our community produces staple food (maize, enset), which depends on the rain rather than using irrigation in the district. This makes those under two and pregnant and lactating mothers suffer from nutritional problems in the district. We have a lack of water resources due to this; we can’t provide irrigation systems in the district; obviously, our worada is low-land ("kola") and is suspected for drought throughout the year; and for the past 3 years, there has been conflict with our neighbouring Oromia region, which is also another problem in the district because they are displaced from their own land and don’t produce or cultivate crops.

9 (I): Have you heard about the multi-sectorial nutrition programme?

(D) Yes, we integrated and collaborated through multi-sector nutritional programmes, creating a string committee to improve nutritional programmes, and giving responsibility to a focal person from each sector. And we also educate the community on how to use multiple food items, avoiding junk foods in the district. We provide safe water for drinking and sanitation purposes and repair non-functional water pumps through daily activities, regardless of other site water sources.

10(I): How does this problem trend look from the past to the present?

(D) In our district, which is densely populated, this also increases nutritional problems, as the trend shows. Most people are begging on the street due to the food shortage in the district, including under-two-year-old children and pregnant and lactating women.

11 (I): What are the nutrition-related activities of your office?

(D) We provide facilities for irrigation based on technologies, provide water in the primary schools, and provide health education on environmental sanitation and personal hygiene based on the aim. And also for prevention of stunting for the underprivileged children, proving and accessing nutrition for pregnant and lactating women in the district based on multi-sectorial nutritional programmes regardless of different sectors in the district,and tackling the burden of nutritional problems. Improving safe drinking by treating and filtering the water sources in the district.

12: What are the roles and responsibilities of your office to achieve the implementation of multi-sectorial nutrition?

(D) According to the Ministry of Health Plan, in 2030, stunting could be eliminated by collaboration and integration with a multi-sectorial nutrition programme targeted at under-two children for prevention of stunting and pregnant and lactating women. We avail water resources, treat water, and provide health education regarding sanitation in the primary schools to reach the community in a simple way. Sustained safe water in the district is needed to overcome nutritional-related health problems in order to achieve and implement good nutritional status at the household level, which is led by the worada administering bodies.

13 (I): Tell me the ownership of the programme.

(D) A multi-sectorial nutritional programme targeted at protecting stunting for only those eligible under two years of age and pregnant and lactating mothers Each sector has a focal person to run this multi-sector programme to implement at the district and household levels, which is coordinated by the Worada Health Office and led by the Worada Admiration Body. Generally, all stakeholder groups are owners of this programme.

14 (I) Tell me the challenges of your office in relation to collaboration with other offices.

About the annual plan, half-year plan, quarterly plan, achievement report, meeting schedule, monitoring, and evaluation-related commitment

(D) Lack of water resources in the district, electric power interruptions, scarcity of budget, inconsistency of report, transportation challenge to reach household level, and scarcity of budget for logistics supplies for construction due to inflation of costs

To tackle this problem, we built a pond here in the district. Regardless of the building, there are high fluoride and iron concentration, which is not safe for drinking. We are evaluating and monitoring from time to time based on schedules in order to achieve the programme.

15 (I) What challenges are there for the community to achieve a multi-sectorial nutrition program?

Probe: ask for the commitment of the community and for any resistance from the

community.

(D) Lack of awareness at the community level, low land area ("kolla"), and lack of water resources in the district are the main challenges, and regardless, our community is committed to implementing a multi-sectorial nutritional programme.

16 (I): Tell me how the structure of this programme is organized.

(DD) We did assessments in the district and created awareness for the community. This programme was arranged by each sector focal person and led by the worada health office, controlled by worada administer, a multi-sectorial nutrition programme based on its own string committee in the kebeles, and collaborated with hygiene and sanitation as well as district level by evaluating and monitoring through the time.

17(I): Say something about the programme in relation to the budget.

(D) A multi-sectorial nutritional programme targets many aspects at the household level, but due to a scarcity of budget, we couldn't achieve them regardless of our plan due to inflation of costs and no cost-based analysis done before implementation of the multi-sectorial nutritional programme. Generally, we are suffering from a shortage of budget to implement a multi-sectorial nutritional programme.

18 (I) HR and other resource issues in your office?

(D) We have focal persons based on water-based professionals and enough skilled human resources; rather than scarcity of budget, we have skilled manpower in our sector, and there is always fiscal budget scarcity through the year, and we don't have motorbikes in order to reach household level.

19 (I): How did the professionals who work on a multi-sectorial nutrition programme

Capacitate?

(D) Multi-professionalism also increases encouragement and capacitates the programme through multi-disciplinary approaches. So, integration and collaboration of different targeted aims go hand in hand in order to build capacity to access safe water, provide health education based on personal hygiene and sanitation, and address under-two-year-old children, pregnant women, and lactating women through a multi-sectorial nutrition programme.

20 (I) Have you had the consultant workshop on this programme?

(D) Yes, a workshop was arranged by the Warada Health Office with collaborating multi-sectorial nutritional programme stakeholders. Participating by community sanitation, health extension workers, and partners in one wash programme and a multi-sectorial nutritional programme focal person from each sector at the worada level, which is led by the regional prosperity party head.

21 (I): How do you involve the community to create awareness?

(D) We select the site first, and all stakeholder groups create awareness on the importance of multi-sectors through multi-disciplinary approaches to be effective in multi-sectorial nutritional

programmes, especially those addressing under-two children and pregnant and lactating women in the selected households. We provide health education regarding hand washing after usage of the toilet and before making food, targeting the prevention of infectious disease in under-two-year-old children and preventing scarcity of nutritionally related problems. And selected a model village, stabling a community laboratory at the village, and creating and demonstrating it before the community. Health extension workers provide health education to the community regarding how to feed children, wash hands before breast feeding, and wash hands after toilet use by using soap, sand, or ash accordingly.

22(I): What are the nutrition-related programmes other than multi-sectorial of your

office?

(D) We avail water, which means safe drinking water, by making filtrate by using ‘tulip," purifying water at the household, protecting environmental sanitation, and repairing the pumps after they break as soon as possible. Evaluated weekly and supervised by our office based on the plan in order to achieve the plan accordingly. Surprisingly, only pregnant and lactating mothers avail of modern stoves in order to protect indoor pollution from smoking and solar systems at house level.

23 (I): Is there a designated responsible body to coordinate the programme?

If yes, how is she or he committed?

(D) Yes, the Worada Health Office and Worada Nutritional Programme are coordinating and leading the multi-sectorial nutritional programmes and having a focal person in order to run the multi-sectorial programmes through transparency and accountability by the Worada Administration Body.

24 (I) What are the strategic and operational plans of your office in multi-sectorial

nutrition programme

(D) Even if we have both a strategic and operational plan, the water resource is out of district (Awada-Boricha water project). Our strategic plan is to construct projects in order to access safe water, which may take 5 years, but our operational plan includes repairing water pumps after they break, treating water, proper management of sewerage, providing health education, and creating

various potential bottlenecks to achieve developmental goals. During an outbreak, we should be alert and treat the water.

25(I): How is the community committed to supporting the activity plan of this programme?

(D) For accessing water and supporting nutritional programmes, communities are very committed to providing support in any aspect. We are engaging stakeholder groups like religious and cultural leaders to announce ownership of the programme and encourage them through the meetings.

26(I): Tell us about the presence of a promising work structure in this programme.

(D) In the past years, those who were pregnant or lactating didn't know how to feed different kinds of food; they only fed their children and themselves in traditional ways. But currently, multi-sectorial nutritional programmes have been launched. The community is ready to practise such programmes and knows the importance of integration and collaboration in multi-sectorial nutritional programmes rather than giving to a single sector. Generally, all communities are ready and integrated for the multi-sectorial nutritional programmes. Currently, most of the community knows about nutritional importance and has addressed primary schools to reach the community.

27 (I): How is the political support for this programme?

(D) In our district, political support is committed in any aspect and led by the Worada Administration or Worada Prosperity Party, which shows a good political commitment based on improving the multi-sectorial nutritional programme in order to prevent stunting for under-two children and save lives for pregnant and lactating women.

28 (I) what do you think of the recommended strategy to improve the

Implementation of a multi-sectorial nutrition programme in your district or region

(D) My recommendations are increasing budget, increasing a worada-based plan, providing a baseline assessment, and providing cost-based analysis for water-related logistics supplies in the district in order to improve and implement the multi-sector nutritional programme through collaboration and integration in order to prevent stunting and maternal deaths.

29 (I): How do you think these strategies can improve the multi-sectorial nutrition programme?

(D) Creating awareness and collaborating with the community on the importance of a multi-sectorial nutritional programme, increasing the annual budget, and availing water for irrigation to sustain a nutritional programme at the household level, improving child and maternal health.

Interview VII: interview with 04 woreda W01

Key ; I: Interviewer

D: Discussant

Section 1: Socio-demographic characteristics of the participants

S,no Items

1.(I) Worada/district/partner/region office name:

(D) 04

2.(I)Sex:

(D) male

3(.I) Age;

(D) 30

4(.I) Marital status;

(D) Married

5(.I) Professions;

(D) *hydraulic engineer*

6.(I)Position;

(D) W002

7. (I) Work experiences

(D) 5 years

Section 2: Multi-sectorial nutrition program implementation related challenges in this office/partner

Theme 1: Challenges in understanding the program

8 (I) Tell me about the nutritional problem in your region/district/locality?

(D) There is a nutritional problem due to climate change in our district because it is lowland. The communities in our district do not make use of irrigation because there is no water source to do so, and they depend totally on rainwater.

9. Have you heard about the multi-sectorial nutrition programme?

(D): Yes, each sector has its own tasks based on its multidisciplinary professionals and focal persons that deal with the problem led by the woreda administrator.

10 (I): How does this problem trend look from the past to the present?

(D) Because the worada is lowland, it is very vulnerable to problems related to climate change, and the trend of nutritional problems has increased from year to year.

11 (I): What are the nutrition-related activities of your office?

(D) providing safe and clean water to the community and facilitating irrigation activities for kebeles situated beside the lake Hawassa.

Maintaining pipes and constructing water points are some of our activities.

12 (I): What are the roles and responsibilities of your office to achieve the implementation of multi-sectorial nutrition?

(D). constructing water sources, installing and maintaining water pipes in households, and providing health education on sanitation, personal hygiene, and sewerage management to primary school pupils.

13 (I): Tell me the ownership of the programme.

(D) Each sector has its own responsibility, led by the Worada Health Office's multisectoral nutrition programme coordinator, with overall supervision being taken care of by the Worada Administrator.

14 (I): Tell me the challenges of your office in relation to collaboration with other offices.

meeting schedule, monitoring, and evaluation-related commitment

(D) The collaboration is not a problem, but the shortage of budget is the main hindrance for us not to collaborate on the issue with other offices.

15 (I): What challenges are there for the community to achieve a multi-sectorial nutrition programme?

(D) The community is pretty much aware of and committed to the programme; they involve themselves in some activities like water point construction, environmental sanitation, and sewerage management.

16 (I): Tell me how the structure of this programme is organised.

(D) . The presence of a focal person in our office and other sectors makes the organising process and reporting pretty easy; moreover, the office administrator oversees the programme, making it more organised.

17(I): Say something about the programme in relation to the budget.

(D) We fail to construct new water points, and we are also unable to maintain the existing ones because of a shortage of budget.

We were even not able to purify and filtrate water due to budget shortages.

18. (I) HR and other resource issues in your office

(D) We have trained personnel regarding the programme, but we challenged the budget shortages.

19(I) How did the professionals who work on a multi-sectorial nutrition program Capacitate?

(D) Through training and different short-term workshops.

20 (I) Have you had the consultant workshop on this programme?

(D) Yes, there are regular workshops organised by the health office of the woreda in collaboration with different stakeholders, ultimately led by the woreda administrator.

21. How do you involve the community to create awareness?

(D) We undergo preliminary surveillance to select the most vulnerable kebeles in the woreda, where we provide health education for the community on the issue. Education before the community regarding how to feed children, washing hands before breast feeding, and hand washing after toilet use by using soaps, sand, or ash accordingly.

22. 22 (I): What are the nutrition-related programme other than multi-sectorial in your office?

(D) We avail safe drinking water by making filtrate by using 'tulip'' and purifying water at the household, protecting the environmental sanitation programme, and maintaining the pipe water by any means possible. To help mothers, pregnant ones, and lactating ones prevent themselves from indoor pollution, we provide them with modern stoves.

23 (I): Is there a designated responsible body to coordinate the programme? If yes, how is she or he committed?

(D) Yes, the Woreda Health Office and Woreda Nutritional Programme are coordinating and leading the multi-sectorial nutritional programmes and having a focal person in order to run the multi-sectorial programme through transparency and accountability by the Worada Administration Body.

24 (I) what are the strategic and operational plans of your office in the multi-sectorial nutrition programme?

(D) We have both a strategic and operational plan regarding water resources in our district. Our strategic plan is to construct projects in order to avail safe water, which may take several years. Our operational plans include maintaining pipe water, treating water, proper management of sewerage, providing health education, and tackling various bottlenecks to achieve developmental goals.

25(I): How is the community committed to supporting the activity plan of this programme?

(D) For accessing water and supporting nutritional programmes, communities are very committed to providing support in any aspect. We are engaging stakeholder groups like religious and cultural leaders to announce ownership of the programme and encourage them through the meetings.

26(I): Tell the presence of a promising work structure in this programme.

(D) The only option that the community had to feed their children and themselves was the traditional way; however, nowadays, as the multisectoral programme is established, they know the importance of integration and collaboration.

27(I) How is the political support for this programme?

(D) Because the woreda administrator is the lead supervisor of the integration, there is active participation and support from politics.

28 (I) what do you think of the recommended strategy to improve the

Implementation of a multi-sectorial nutrition programme in your district or region

(D) My recommendations for improving and upgrading the budget allocation

29 (I): How do you think these strategies can improve the multi-sectorial nutrition programme?

(D) It has created awareness among the community to sustain maternal health, improve child health, and allocate budgets.

Interview VIII: interview with WNF of 04 woreda

Key ; I: Interviewer

D: Discussant

Section 1: Socio-demographic characteristics of the participants

1.(I) Woreda/district/partner/region office name:

(D) 04

2.(I)Sex:

(D) male

3(.I) Age;

(D) 32

4(.I) Marital status;

(D) single

5(.I) Professions;

(D) *Irrigational engineer*

6.(I)Position;

(D) W002

7. (I) Work experiences

(D) 12 years

Section 2: Multi-sectorial nutrition program implementation related challenges in this office/partner

Theme 1: Challenges in understanding the program

8 (I) Tell me about the nutritional problem in your region/district/locality?

(D) Nutritional problems are of different types in our district; some merely expect from the government while they have a plot of land to work on due to a knowledge gap, while others do not have land to plough.

9. (I)Have you heard about the multi-sectorial nutrition program?

(D): Yes, to prevent stunting among children, there needs to be some activities like providing a balanced diet in the first 1000 days starting from pregnancy and supplying pure drinking water in collaboration with different sectors.

10 (I) How this problem trend looks from past to the present?

It is obvious that as the population increases over the past few years, the trend of the problem also increases.

11(I) What are the nutrition related activities of your office?

(D) In collaboration with nongovernmental organisations and with support from the regional government, we have tried to supply pure water to the community, solar pumps for irrigation to residents around Lake Hawassa, and a dug water pit.

12 (I) What are the roles and responsibilities of your office to achieve the

Implementation of multi-sectorial of nutrition?

(D). In Garbicho Kebele, where fetching water is difficult, we constructed water stands and water harvesting in Karara Kebele, created awareness in the community via schoolchildren in collaboration with the schools, and maintained around 16 water stands that were not functioning properly and stopped working somehow.

13 (I): Tell me the ownership of the programme.

(D) The woreda administrators lead the steering committee, and the head of the health office leads the technical committee. The role of the water bureau is to deal with the part that is related to us, which is water.

14 (I) Tell me the challenges of your office in relation to collaboration with other

offices.

About the annual plan, half-year plan, quarterly plan, achievement report,

meeting schedule, monitoring, and evaluation-related commitment

(D) A shortage of budget, transportation, and bureaucratic impediments in finance offices would've been neat had there been an independent financial unit specific to the programme.

15 (I) What challenges are there for the community to achieve a multi-sectorial

nutrition programme?

(D) The community is very committed to the programme; they involve themselves in some activities like water point construction and environmental sanitation.

16 (I): Tell me how the structure of this programme is organised.

(D) . The presence of a focal person in our office and other sectors makes the organising process and reporting pretty easy; moreover, the office administrator oversees the programme, making it more organised.

17(I): Say something about the programme in relation to the budget.

(D) We fail to construct new water points, and we are also unable to maintain the existing ones because of a shortage of budget.

We were even not able to purify and filtrate water due to budget shortages.

18. (I) HR and other resource issues in your office

(D) We have trained personnel regarding the programme, but we challenged the budget shortages.

19 (I): How did the professionals who work on a multi-sectorial nutrition programme

Capacitate?

(D) through training and different short-term workshops.

20 (I) Have you had the consultant workshop on this programme?

(D) Yes, there are regular workshops organised by the health office of the woreda in collaboration with different stakeholders, ultimately led by the woreda administrator.

21. How do you involve the community to create awareness?

(D) We undergo preliminary surveillance to select the most vulnerable kebeles in the woreda, where we provide health education for the community on the issue. education before the community regarding how to feed children, washing hands before breast feeding, and hand washing after toilet use by using soaps, sand, or ash accordingly.

22. (I): What are the nutrition-related programmes other than multi-sectorial of your office?

Probe: ask for a list of activities for this programme. Its aim? Any plan?

(D) We avail safe drinking water by making filtrate by using ‘tulip’’ and purifying water at the household, protecting the environmental sanitation programme, and maintaining the pipe water by any means possible. To help mothers, pregnant ones, and lactating ones prevent themselves from indoor pollution, we provide them with modern stoves.

23 (I): Is there a designated responsible body to coordinate the programme?

If yes, how is she or he committed?

(D) Yes, the Worada Health Office and Worada Nutritional Programme are coordinating and leading the multi-sectorial nutritional programmes and having a focal person in order to run the multi-sectorial programmes through transparency and accountability by the Worada Administration Body.

24 (I) What are the strategic and operational plans of your office in multi-sectorial

nutrition programme

(D) We have both a strategic and operational plan regarding water resources in our district. Our strategic plan is to construct projects in order to avail safe water, which may take several years. Our operational plans include maintaining pipe water, treating water, proper management of sewerage, providing health education, and tackling various bottlenecks to achieve developmental goals.

25(I): How is the community committed to supporting the activity plan of this programme?

(D) For providing water and assisting nutritional programmes, communities are very committed to supporting them by all means possible. Stakeholders, religious organisations, and cultural leaders should be involved in the ownership of the programme and encouraged to encourage it.

26(I): Tell the presence of a promising work structure in this programme.

(D) The traditional way of feeding has been replaced by a modern balanced diet due to multi-sectoral programmes of involvement and integration.

27(I) How is the political support for this programme?

(D) There is active participation and strong support from the political side, as the politicians are part of the integration.

28 (I) What do you think of the recommended strategy to improve the

Implementation of a multi-sectorial nutrition programme in your district or region

(D) to increase budget allocation and facilitate financial units specific to the programme.

29 (I): How do you think these strategies can improve the multi-sectorial nutrition programme?

(D) by improving maternal and child health through a balanced diet.
