# Supplementary material for: Exploring the challenges and opportunities of multisectoral nutrition programme in Ethiopia: A qualitative study on combating undernutrition during pregnancy
Source: PLoS One. 2025 Jul 3;20(7):e0311336. doi: 10.1371/journal.pone.0311336 (PMC12225801; doi:10.1371/journal.pone.0311336)
Supplement: S6 Data — (PDF) [file pone.0311336.s007.pdf]

Interview 2 – 04 district WCNF

Data Collector: z

Translator, and Transcriber: A

Key; I: Interviewer

D: A

1. (I) Woreda/district/partner/region office name:

(D) 04

2.(I)Sex:

(D) male

3(.I) Age;

(D) 45

4(.I) Marital status;

(D) Married

5(.I) Professions;

(D) cooperative

6. (I) Position;

(D) WCNF

7(I) Work experiences

(D) 17 years

8 (I): Tell me about the nutritional problem in your region, district, or locality.

I: the nature of the nutritional problem in the local context.

(D) There is huge nutritional problem in our woreda. Residents are not able to eat thrice per day with problem leaving women and children more vulnerable to the problem. When we compare to the surrounding woredas like 02 and 01 the problem is much greater here because we rely on maize and haricot bean that are dependent on rain for production but those woredas at least have ENSET that doesn't need rain to produce. For there was no rain last year, the production was very low and the problem was also proportional to it.

9. Have you heard about the multi-sectorial nutrition programme?

I: If yes, would you say something about its policy, principles, and implementation?

(D): Yes, it is a programe that practiced by the collaboration of six offices namely (health office, education office, finance office, women and children office, agriculture office, livestock and production office) to achieve a common goal which is reducing nutritional problem, empowering women, combating harmful traditional practices, balanced diet in the first 1000 days starting from conception to till two years of age for the health and future productivity of the newborn and its mother etc. in the woreda.

10 (I): How does this problem trend look from the past to the present?

Prior to the commencement of this program I never knew about the importance of taking care for 1000 days since conception which is very imperative both for mothers and the child throughout his/her adulthood, but after the program, there comes awareness and we started passing it forward to the community. We also advise them on exclusive breast feeding and complementary feeding after six months.

11 (I): What are the nutrition-related activities of your office?

I: Would you say more about each nutrition-related activity in your office?

List of activities? Its aim? Any plan?

(D) Together with all the sectors involved we provide awareness creation for women to empower themselves, make proper use of what they plant in their backyard for them to have a balanced diet to tackle nutritional problem. Women used to eat after their husbands (their left overs) earlier and we are working on creating awareness to stop this discrimination though there still are remnants of this malpractice among few. Furthermore they were not recognized for working sixteen hours per day including the household chores for the family which nowadays is becoming history.

12 (I) What are the roles and responsibilities of your office to achieve the Implementation of multi-sectorial nutrition

(D). providing training and creating awareness on the importance of nutrition in the first 1000 days, trade, selecting candidates for safety-net programe, harmful traditional practice like female genital mutilation, abduction, early marriage,

13 (I): Tell me the ownership of the programme.

I: detail about the coordinator of the programme and this body's responsibility.

(D) Each sector is committed enough to run their assigned programe, I do not think there is any issue regarding the ownership. It even appears in our dreams and we ponder about it day and night as it is a responsibility to from the government and on top of that we want to see our community freed from the problem that we are working against.

14 (I) Tell me the challenges of your office in relation to collaboration with other offices.

I: detail about the procedure of the programme, such as:

About the annual plan, half-year plan, quarterly plan, achievement report, meeting schedule, monitoring, and evaluation-related commitment

(D) We fail to meet our objective after planning on some issues, for example, when we plan to train women on trade, the time schedule should take fifteen days but never have we ever achieved it because of inadequate budget. We plan to create awareness on issues like saving, credit, ANC, delivery & PNC, screening on breast cancer and cervical cancer which are activities that need long distance travel to the community but because of shortage of budget even for perdiems and logistics we fail to do them according to our plan.

15 (I) What challenges are there for the community to achieve a multi-sectorial nutrition programme?

Probe: ask for the commitment of the community and for any resistance from the community.

(D) The community is pretty much committed and cooperative to what they are told to do.

16 (I): Tell me how the structure of this programme is organized.

I: reporting system of the programme.

(D) .we report to the focal person in the woreda and him to the region and so on.

17(I): Say something about the programme in relation to the budget.

I: the financial administration, adequacy or shortage, and others.

(D) We fail to construct new water points, and we are also unable to maintain the existing ones because of a shortage of budget.

We were even not able to purify and filtrate water due to budget shortages.

18:(I) HR and other resource issues in your office

(D) We have trained personnel regarding the programme, but we challenged the budget shortages.

19 (I): How did the professionals who work on a multi-sectorial nutrition programme Capacitate?

I: opportunities for training, workshops, and others.

(D) To bring about change in the community through awareness creation and we need to be changed in the first place, for this reason we have been receiving some training and workshop that are imperative e to run the programe. For your information, I never used to know that providing good nutrition to the pregnant mother since her conception would benefit the child until I took the training, however after that I became aware about it and started passing the knowledge down to the community that are cooperative enough for the idea making it easy for us to work without problem.

20 (I) have you had the consultant workshop on this programme?

I: If yes, details like who were participants?

(D) If you mean about sekota programe, we have consultant workshop at most thrice per year and at least twice per year.

(I): how do you see the commitment of focals in each sectors?

(D): all the focals in every sectors involve actively in the programe except the focal in agriculture office who has been replaced for lack of commitment.

21.How do you involve the community to create awareness?

(D) We undergo preliminary surveillance to select the most vulnerable kebeles in the woreda, where we provide health education for the community on the issue. education before the community regarding how to feed children, washing hands before breast feeding, and hand washing after toilet use by using soaps, sand, or ash accordingly.

22(I): What are the nutrition-related programmes other than multi-sectorial of your office?

I: a list of activities for this programme. Its aim? Any plan?

(D) We avail safe drinking water by making filtrate by using ‘tulip” and purifying water at the household, protecting the environmental sanitation programme, and maintaining the pipe water by any means possible. To help mothers, pregnant ones, and lactating ones prevent themselves from indoor pollution, we provide them with modern stoves.

23 (I): Is there a designated responsible body to coordinate the programme?

If yes, how is she or he committed?

(D) Yes, the Worada Health Office and Worada Nutritional Programme are coordinating and leading the multi-sectorial nutritional programmes and having a focal person in order to run the multi-sectorial programmes through transparency and accountability by the Worada Administration Body.

24 (I) what are the strategic and operational plans of your office in multi-sectorial nutrition programme

(D) We do not have any plan of our own, it has been a year since the programe has been launched and we are still acting on plans received from regional office.

(I): tell me the different functions of steering and technical committee?

(D): the steering committee which is composed of heads of involving organizations evaluate the work in each sectors while the technical committees do the technical work concerning the programe.

(I): in which committee is the coordinator?

(I): The coordinator works with both committees and participates in when there are meeting of both committees.

25(I): How is the community committed to supporting the activity plan of this programme?

(D) For accessing water and supporting nutritional programmes, communities are very committed to providing support in any aspect. We are engaging stakeholder groups like religious and cultural leaders to announce ownership of the programme and encourage them through the meetings.

26(I): Tell the presence of a promising work structure in this programme.

(D) The only option that the community had to feed their children and themselves was the traditional way; however, nowadays, as the multisectoral programme is established, they know the importance of integration and collaboration.

27(I) How is the political support for this programme?

(D) Though the government is not financially supporting the programe, it is working very good job towards clearing the obstacles against the programe as it has already internalized that the programe is for its own auspicious benefit.

28 (I) What do you think of the recommended strategy to improve the

Implementation of a multi-sectorial nutrition programme in your district or region

I: a general and office/sector-contextualised recommended Strategy?

(D) the aim of the programe is to reduce and ultimately eliminating nutrition related problems like stunting and creating productivity among the coming generation that leads to development of the country, for this reason this nutrition programe which includes working since conception of the mother should include all the areas in our woreda and should be scaled up at country level.

I): what happened to the hens you distributed to the community? Are they still alive?

D): for the climate in our woreda is favorable for hens, they are still alive and at condition. What is expected from us is advising and creating awareness among the community to consume the product of hens at household level instead of taking it out for market.

29 (I): How do you think these strategies can improve multi-sectorial nutrition? program?

(D) It has created awareness among the community to sustain maternal health, improve child health, and allocate budgets.

Transcripts Interviews in WC01

Interview 3 – 02 WC01

Data Collector: A Translator, and Transcriber

Key; I: Interviewer

D: Respondent

I: Woreda/district/partner/region office name?

D: 02

I; sex

D: Female

I: how old are you

D; I am 34 years old

I; what is your profession?

D; I have a degree in business management

I; what is your work position?

D; I am the WC01

I; how many years did you work?

D; I worked in government office for 13 years among which 8 years as manager in different position.

I; what nutritional problem exist in your area?

D; in our woreda, the people usually consume ‘Kocho," and they drink coffee afterwards. However, the most common food that has been consumed is maize. Previously, when agricultural production was good, everything was good and healthy. However, now that agricultural production has fallen, people are at risk, particularly children and mothers. In the past two years, there has been a climate change, and the farmers have yielded nothing from their agriculture, which is leading to a loss of health. As I told you before, the main crops in our area were maize and haricot beans, but the production failed and the farmers got nothing. As you can see, the children are in trouble and are at risk of malnutrition. The mother and children are getting out on the streets to beg. Moreover, the cattle died due to drought caused by a shortage of rain, and the children are not getting milk and butter as before. Due to the shortage of rain in the past two years, there is a shortage of food in the community, and mothers and children are at risk of hunger.

I: How does this problem trend look from the past to the present?

D; The problem increased compared to the previous In the past two years, the problem has significantly increased due to the shortage of rain resulting in poor agricultural production, making it difficult to even survive. Additionally, there is no non-governmental organisation working in our woreda, which makes it even more difficult to support those in high need. Derara, 03, Chuko, and Dara Otilcho, including 04 woreda, are drought-prone areas with high temperatures. Due to the fact that in the past two years the community has not gotten any results from agriculture, it is becoming difficult to fulfil their basic needs.

I; Have you heard about the multi-sectorial nutrition program?

D; yes, I heard about it.

I; would you say something about its policy, principles, and implementation?

D: Yes, it is coordination work. This programme works by coordinating the agriculture office, livestock and fish resource office, health office, and water supply office. In general, it coordinated 13 district offices to work towards one goal. For example, the policy in the agricultural office was to increase agricultural production and productivity. In addition, when lactating women face food security, they provide something to eat. When we see the livestock and fish resource office, they have their own policy regarding improving production. Regarding water, since water is life for all human beings, it is particularly important for children's and mothers' health. The policies of all offices are intertwined since they are working to accomplish the same goal: improving maternal and child health through nutrition.

I; what are the nutrition related activities of your office?

D; The nutrition-related work we are doing is not directly related to food and nutrition. But it makes a great difference indirectly. We work on saving and making wise use of resources; this highly relates to family wellbeing. When a mother saves her money and uses it wisely, her children will not be vulnerable for problem like those who did not save. In sidamu afoo"Ama suuqidhuro qaqqu doogo difulaa". We give them awareness on saving, how to effectively work on their farm, and resolving conflicts in the household. During the ‘meher’ and ‘belg’ agricultural production seasons, we work with the agricultural office. In every development group, we identify which mother works differently and effectively, and we will make her an idol for the rest of the group. When we work with animal and fish resource offices, we work on creating awareness regarding small-scale poultry farms and encourage all mothers to start rearing hens and cocks. This helps to increase the availability of protein-rich food in their households and also generates income through the sale of eggs and meat. Additionally, we provide training on the proper care and management of poultry to ensure healthy growth and productivity. With the district health office, we teach them to feed their child appropriately; the child needs to eat eggs and drink milk in order to prevent stunting ("mekencher"). This is related to the currently initiated programme called "Sekota Declaration." The programme aims to reduce malnutrition and stunting in children under five years old. By educating communities on the care and management of poultry, we hope to provide a sustainable source of nutrition for families and contribute to the success of the Sekota Declaration.

I; in addition to the above, is there anything you want to add?

D; In addition to poultry, we are working on improving garden farming by providing improved seeds for households. In order to improve the family’s nutrition, we give them the seeds of lettuce, carrots, and other vegetables. Children's and mothers’ health is our top priority, and we

believe that a well-balanced diet can help prevent illnesses and diseases. In coordination with the 13 offices, we also provide orientation on proper gardening techniques to ensure that the families are able to maximize their harvest and sustain their own food source. Our main work is coordinating and preventing obstacles.

I; what are the roles and responsibilities of your office to achieve the implementation of multi-sectorial of nutrition?

D; for example, the agricultural office works on general district agricultural production while we focus on the production of women and youth. They made a home-to-home visit and provided improved seeds. The agriculture office works with the male household head, while we coordinate with the female household head. We work with the females to take the improved seeds of maize and haricot beans and plant them in their fields. Additionally, we provide training on proper planting techniques and offer ongoing support to ensure successful harvests. This approach has led to increased crop yields and improved food security for the households we work with.

I; what will improved by doing this?

D; This improved the nutrition of the family with the consumption of a balanced diet , leading to better health outcomes and reduced medical expenses. Additionally, it also positively impacted the children's academic performance and overall well-being.

I; what are the roles and responsibilities of your office to achieve the implementation of multi-sectorial of nutrition?

D; The main responsibility of our office is to create awareness. Through development groups, one to five and one to ten networks in the kebele, we create awareness, which eventually improves the lives of the mother and the whole family. In addition, we teach the mothers to save money and work together in groups to start small businesses that can generate income for their families. This helps to reduce poverty and improve the overall well-being of the community. We teach them they have to generate their own income rather than solely depend on their husbands' income.

I; Tell me the ownership of the program?

D; we are the owner of the program as women and young affairs office.

I; who are the coordinator of the program and this body responsibility?

D; the regional government is the coordinator of the programme. At the district level, all 13 district sector offices will work together. Our office always looks after the work on maternal and child issues; in addition, we file all individual and coordinated works. Furthermore, we regularly conduct monitoring and evaluation activities to ensure the effectiveness of our interventions.

I; what are the challenges of your office in relation to collaboration with other offices?

D; we did not face any challenges working with other offices. There are 13 stakeholders working in the program and we are working smoothly with all the sector offices in the district.

I; when a group person work together it is natural to got a challenge, is there any?

D; I said no because every sector office shared responsibility for this issue. When we work with the health office, they have a strategy on maternal and child health, which increases the effort. In addition, when we work with the education office, we work together since they are working to achieve their plan to register all school-age children and eliminate dropout, which could not be achieved without food security. The child could not learn if he was not fed. So, since we have somewhat shared responsibilities and goals, we are working smoothly. We have no obstacles that make us not want to work together.

I; What about the annual plan, half-year plan, quarterly plan, achievement report, meeting schedule, monitoring, and evaluation-related commitment?

D: Yes, we have an annual, half-year, and quarterly plan and reports in addition to the monthly and weekly schedules. Every sector office is working together, and we did not have a significant challenge in this regard. Since the management is also committed to this programme, we all meet every month to discuss the work.

I; what challenges are there for the community to achieve a multi-sectorial nutrition programme?

D; In the community, there was no obstacle or challenge specific to this nutrition programme; the challenges we are facing are when we work on issues like harmful traditional practices. In this regard, the elderlies and justice offices will engage, in addition to the poor awareness of the community regarding the issue. Generally, there is no community-level challenge when we work on nutrition programmes.

I; Tell me how the structure of this programme is organized.

D: The programme is huge in structure. All the sectors, including the management, are working together. The sector office management immediately stops what they are doing when this programme work arrives. We plan and share the weekly programmes on Monday, and we will receive the weekly reports on Friday.

I; when did the staffs report their work?

D; we receive the weekly report every Friday.

I; what did you do with the report?

D; we will compile the weekly reports and send to the region every month.

I; what about the program in relation to the budget?

D; We get the budget at the district level, which includes the salary of the programme staff. Moreover, when there is extra work, we will get an additional budget. When we have an activity, the finance department will present the budget to the district cabinet, and then we can buy the necessary equipment or facilitate the activity. However, some times the district cabinet lacks information on the exact sectors working on the programme, which will become a minor challenge. In addition, since our region is new, we are not getting the exact figure we are looking for due to the budget shortage the region is facing. Nonetheless, it is difficult to run this programme with only the government budget; the NGO working in the district has left. To do field work, we need fuel for the motors, even though our office does not have a motorbike. In general, there is a budget shortage to work extensively.

I; What about HR and other resource issues in your office?

D; In our office, we have adequate human resources; the shortage we are facing is the budget. We plan to do field work, but usually we fail to accomplish all the planned activities due to a budget shortage.

I; could you mention other resources?

D; there is shortage of fuel and stationary. Even our office did not have motorbike to conduct field visits. The budget shortage even making it difficult to pay salary for district employees, it is a huge problem of the whole region.

I; how did the professionals who work on a multi-sectorial nutrition program capacitate?

D; As we all know, everybody will not have equal capacity, and some of the staff, including our office staff, lack the necessary experience to complete the work exactly as planned. Most of the professionals working have good knowledge and experience.

I; opportunities for training, workshops, and others?

D: They need some appreciation; they will go to Kebeles on Monday and return on Friday; we only support them with a very small incentive for transportation. We know there is a shortage of budget to award them, but they need appreciation.

I; Have you had the consultant workshop on this programme?

D: Yes, previously we had a programme on HIV called "buna tetu," a tea-coffee ceremony to discuss the issue. However, currently we have stopped the programme due to a shortage of funds to conduct the study.

I; How do you involve the community to create awareness?

D; In the community, we have a mothers' network structure to share ideas and discuss nutrition and the health of themselves and their children. So we use this existing structure to involve the females and improve the awareness in the community, since if the mother had awareness, the family would eventually have good awareness regarding feeding and nutrition.

I; What are the nutrition-related programmes other than multi-sectorial in your office?

D; In addition to this programme, we coordinate with the agricultural programmes to improve the nutritional status of our clients. The programmes are inadequate, and when some community members come to our office, it is scary to watch the hurt. Begging on the street is becoming more common and is increasing. This is not only the problem of not working, but it is also due to the loss of agricultural production due to the drought.

I; Is there a designated responsible body to coordinate the programme?

D; sometimes the region will call us to coordinate those programmes. The programmes are coordinated by the agriculture office since the programme will be effective if they lead it. When we work with the livestock and fish resource office, sometimes we coordinate; other times they will take the lead. Generally, they coordinate the main work, and we focus on awareness-creation activities.

I; For example, who is responsible in your office?

D; I am responsible.

I; What are the strategic and operational plans of your office in the multi-sectorial nutrition programme?

D; We have three- and six-month plans with the agriculture and natural resource offices, and we coordinate to achieve our goals.

I; What are those plans?

D: You are working on nutrition only, but our coordination goes beyond that; we have the three- and six-month plans for those programmes too.

I; How is the community committed to supporting the activity plan of this programme?

D; it is good. Currently, the community listens to what the programme staff is teaching, and the motivation and commitment of the community have increased to alleviate the problem.

I; Did the community support the programme?

D: Yes, the support from the community is good. They mainly support the programme by learning and practicing the recommended activities.

I; Tell me about the promising work structure of this programme.

D; Since every sector is working towards the same goal, it is a promising structure. The programme encouraged the community to use fertilizer, use appropriate seed, and perform improved agricultural activities to improve production and productivity. Regarding livestock, we teach them to take the hens to start a small-scale poultry farm. We create awareness, while the livestock and fish resources will work with them to improve productivity.

I; How is the political support for this programme?

D; All the work we discussed today needs political stability and involvement. All the sectors have strategic plans to accomplish the shared goals. The political office also took the agenda seriously, and every sector office head will present its plan and achievements regarding this programme, which will be evaluated, the decisions also being taken too centrally. Generally, the commitment and support shown by the political leaders were very good.

I; What about the support from the regional government?

D; To speak frankly, the regional office supported us two or three times, which might be due to the busy schedule they have. In November, we asked for support from 200 people, who provided us with several supplies, including floor and female sanitary supplies. Moreover, they support us by providing capacity-building training.

I; What do you think of the recommended strategy to improve the implementation of a multi-sectorial nutrition programme in your district or region?

D; The strategies of the multispectral programme are very good. If we work hard and commit ourselves to the programme activities, we can eventually decrease the problem. The strategies of the programme are very good; we just have to do the activities.

I; How do you think these strategies can improve the multi-sectorial nutrition programme?

D; I am happy with all the strategies of the programme. It only needs action. I am happy that you came to ask us questions and forward any problems we faced.

I; What about specific strategies for resolving women's and child's problems?

D: obviously, the family problem decreases when the mother problem is relieved, so this programme will be effective since it is working by giving focus to the rural mothers. And we are happy with that.

I; What about working with pregnant mothers?

D; In coordination with the HEWs, we are encouraging mothers to take vaccinations, follow their pregnancy, and give birth at a health facility. In addition, we are working to improve the food consumption patterns of pregnant mothers since this is crucial for the baby too. We are also working to improve the dietary practises and nutritional status of children under two and children under five. Previously, the mothers would sell all the eggs in the house, but now they have improved and sell half and feed the rest to their children. This is working for milk and butter too.

I; Now that I'm almost done with my questions, you can express any concerns you have about this programme.

D. Since the programme is working with aware and non-aware individuals, it has to give additional focus to awareness creation. The problem in our community is due to climate change, and it will be improved if we work hard. I know you are working on research, but you have to inform others about the problem, and we have to increase our efforts to alleviate this problem in our hard-working community.

Thank you.

Interview 4 – 02 district WCNF

Data Collector: A Translator, and Transcriber

Key I: Interviewer

D: Respondant

I; Woreda/district/partner/region office name?

D; 02

I; sex?

D; male

I; how old are you?

D; I am 36 years old.

I; what is your marital status?

D; married

I; what your profession?

D; sociology

I; position

D; WCNF

I; Work experiences?

D; 12 years in total; six years in agriculture office, 2 years in conservation office and 4 years in women affairs office.

I; Tell me about the nutritional problem in your region/district/locality?

D: Regarding the nutritional problem in our area, the community has eggs, milk, cabbage, bananas, and pepper but does not practice consuming a variety of foods. After the initiation of the sekota programme, we are working on passing nutrition-related messages through the community development army to create awareness about the topic. We are working on creating awareness about appropriate feeding practices; previously, mothers' ate two times a day after feeding the father and child. They eat small portions of non-diversified food, which puts them at risk for nutritional problems during pregnancy and lactation. But no, we are teaching the mothers to eat at least three times a day and eat protein-rich foods for the health of the baby. After the Sekota programme started, we created awareness about appropriate feeding habits for pregnant and lactating mothers.

I; Okay, good, but what nutritional problems exist in this area?

D: The main problem in this area is consuming a monotonous or single type of food throughout the year. Currently, we are working with a multisectoral team to introduce cabbage, carrots, and lettuce to diversify the dietary consumption of the community. Moreover, there is a shortage of food in most households. As you know, half of the community depends on agriculture, and the other half is pastoralist. There is also a huge health problem related to nutrition. Climate change has resulted in drought production loss in our district for three consecutive years; in 2012, 2013, and 2014, the rain was very low, resulting in harvest loss for farmers. Food insecurity is the main challenge, and begging on the street is increasing.

I; Have you heard about the multi-sectorial nutrition programme?

D; I have not only heard about it, I am working on it. There are seven sectors, including our multi-sectorial nutrition programme, and we are working together. For example, the agricultural bureau provides us with cabbage, lettuce, and others for mothers. Multi-sectorial is working on pregnant and lactating women to prevent stenting due to a shortage of food, and the Water Office will make clean water easily accessible for every mother in our community. In addition, the livestock and fish production office will provide us with hens and goats for pregnant mothers to feed the child eggs and milk. Furthermore, the education office will create awareness regarding nutrition and stunting among students, and in return, the students will teach their families. As the women and child affairs office, our responsibility is to select the mothers for the service, identify the pregnant and lactating mothers, and disseminate nutrition-related messages in the community through religious leaders, elders, and the community development army. For example, the agriculture office will provide the supplies, our office will then work on improving the dietary practises, and our office will focus on behavioural change.

I: If yes, would you say something about its policy, principles, and implementation?

D; The multi-sectorial nutrition programme is a 15-year strategic programme. The programme is our national programme, and it has three phases. In the first five-year phase, the programme has been implemented in Sekota district in Wollo, Amhara region, which is one of the most affected areas in terms of malnutrition. In the second phase, this declaration selected 240 most affected districts in the whole country, including 10 districts in the Sidama region, of which our district is one. This programme is led by the prime minister at the national level, the regional president at the region level, and district administrators at the woreda level. In addition, at the woreda level, there is a string committed to which all the seven sector heads are members, and the focal members from the seven sectors are technique team members. With three phases, the Sekota Declaration is working to eliminate stunting in Ethiopia by 2030.

I: This is the policy?

D; Yes, the policy of the programme is to eliminate stunting from our country; it might be difficult since the generations continue and as the community lives along, there may be occasions. However, the work has to be done continuously.

I; What are the nutrition-related activities of your office?

D; In our office, the main nutrition-related activity we are doing is behavioural change. We are doing this to make all women, particularly pregnant and lactating mothers, practise good nutrition and become self-sufficient. During' meher' and 'belg' agriculture seasons, the women have their own plan to saw maize and haricot beans, so we support them to collect their own harvest and become self-sufficient. Moreover, there are female-headed households and widowers in the community, and we provide special support to them. If they have a shortage of seed, we will provide seeds for them, coordinating with the agriculture office. Our woreda is drought-prone, but no NGO is working to support us. Especially as our office, which works on behavioural change, needs huge support, previously we could write project proposals and invite organisations to work with us, but now only the region can do that. Even though there is a resource shortage, we are working to enable women in our community to work equally as hard as men and generate their own income. For example, there are five children in the house; the children are 'lemigeb yederesu lesera yalderesu nachew," so the male small-scale farm cannot feed the family adequately; therefore, we are working with the mother in one-to-five networks and kebele leagues to enable them to generate their own income.

I: Would you say more about each nutrition related activity in your office? List of activities? Its aim? Any plan?

D. For example, in our office, we planned for 10,000 women to work on their land during the fall season, we planned for 1000 hectares of land to be prepared for cultivation, we estimated the amount of seed needed for that, and we arranged for provision. The women cultivate intercrop production; they will see mainly maize and haricot beans with an 80:20 ratio.

I; What are the roles and responsibilities of your office to achieve the implementation of multi-sectorial nutrition?

D; Our main role in the multi-sectorial programme is disseminating nutritional messages to mothers. Secondly, our role is to identify pregnant and lactating women and ensure they get support in order to prevent malnutrition among them. Moreover, we work with the health office to create awareness regarding antenatal and postnatal care follow-up and improve the utilisation

of the services, including immunization for their children. We collaborate with the education sector to maintain the hygiene of the students by creating awareness; additionally, we teach the students about nutrition so they can also teach their mothers. Generally, our role in the multi-sectorial nutrition programme is to promote healthy nutrition among mothers.

I; Tell me the ownership of the programme.

D; There is a string team that meets once a month to discuss the plan and evaluate the activities. I am a member of the technical committee. The committee has seven members, and we meet every two weeks to assess the plan and the achievements. With our coordinator, we evaluate the activities and report to the string committee. I prepare an action plan and an annual plan; for example, I make sure that pregnant women take training on the IG programme; the community development army disseminates nutrition-related messages; I ensure this using a checklist; I identify orphans and vulnerable children and make sure they are provided with support. Generally, I and Mihret led the programme in our office.

I; Tell me the challenges of your office in relation to collaboration with other offices?

D; The first challenge we faced working with other offices was that the gender professionals working in each sector were not taking their jobs seriously. However, as a women's affairs office, the main challenge we are facing is an attitude problem towards our contribution, but there is an improvement in this regard. In addition, we are facing transportation problems in order to do fieldwork with other sectors, and we have a budget shortage on our side to conduct all the jointly planned activities. The other sectors, like agriculture and health, have adequate budgets and logistics to do the activities; they are doing their specific jobs, but since our main role is bringing behavioural change, which is crucial for their work too, we fall behind due to the budget shortage.

I; What challenges are there for the community to achieve a multi-sectorial nutrition programme?

D: The community is good, but there is a message mix-up; every sector is disseminating messages individually, which is creating confusion in the community. Due to the budget shortage, we are not going together. The programme is designed to be multi-sectorial, but the activities are fragmented, which in turn creates confusion and mix-ups in the messages. However, if we plan and execute the activities together, this confusion will not be an issue.

I: ask for the commitment of the community, for any resistance from the community.

D; in addition, the other challenge is that since the community is consuming monotonous food, stunting is prevalent. There is a shortage of food and a shortage of seed to produce locally. The programme has to start modelling certain families and establish 'serto masaya' in order to provide seeds of vegetables and other crops to those in need. In addition, supplies of poultry are needed; we teach them thoroughly, and they will accept, but they do not have the money to buy the hens. Generally, the main challenge in the community is a shortage of supplies. All the kebeles are in need of support, but we are working on a few selected kebeles. In the past year, we have worked on four kebeles, but due to a shortage of budget, we are working only on two kebeles. For their example, the sekota programme budgeted 100,000 birr for our office; from this, we used 45,000 for IG training for 30 women, 18,000 for harmful traditional practise training for 60 individuals, and another 18,000 for 1000 days of training. Not only in the offices, the financial shortage is huge in the community.

I; is there any resistance from the community?

D; The community accepted the programme well. Even those kebele that are not selected for the programme are requesting it. They are even asking us to arrange a field visit to those kebeles to share experiences from the selected kebeles. The programme is well accepted in the community; there is no attitude problem regarding the programme.

I; Tell me how the structure of this programme is organized, reporting system of the programme.

D; We have a reporting system. We have a monthly report and a quarterly report; the reports will be received from each kebele. In addition to our annual plan, which is divided into quarterly plans, our plan is simple and brief. Our plan includes IG training for pregnant and lactating women, training for community-based development armies, and activities on preventing early marriage. In addition, reports on the support provided to those women in the safety net programme and the support provided to orphans will be sent to us from the kebeles, and we will compile and send them to our coordinators and sekota programme staff, which in turn will be sent to regional bureaus.

I; Say something about the programme in relation to the budget.

Probe: ask for the financial administration, adequacy or shortage, and others.

D, mentioned above.

I; HR and other resource issues in your office

D; We have adequate staff working on the programme, but the available human resources need financial resources to conduct field activities. We have a shortage of logistical items like computers and motorbikes. We did not have a shortage in human resources.

I; How did the professionals who work on a multi-sectorial nutrition programme capacitate themselves?

D; Besides the training the region provided at the initiation of the programme, there are no capacity-building trainings given to the staff at the woreda or regional level due to a budget shortage. However, as the focal person, we usually meet and discuss ideas at an inter-sectorial level.

I; Have you had the consultant workshop on this programme?

D; We provided 100,000 ETB to conduct four trainings yearly; besides that, we did not participate in or prepare any workshops or capacity-building trainings.

I; What are the nutrition-related programmes other than multi-sectorial in your office?

D; No, we did not have any other nutrition-related programmes.

I; Is there a designated responsible body to coordinate the programme?

D: Yes, I'm responsible.

I; If yes, are you committed?

D; I am committed to all of my work; you can ask my supervisors for more.

I; What are the strategic and operational plans of your office in the multi-sectorial nutrition programme?

D; Our long-term plan is to eliminate stunting among women by 2030. Creating adequate nutrition-related awareness is also part of our long-term plan. Preventing genital mutilation and early marriage are our short-term goals that we are working on.

I; How is the community committed to supporting the activity plan of this programme?

D: The support from the community is very good; everybody, starting from the kebele administration to each household head, is committed.

I; Tell me about the promising work structure of this programme.

D; The programme structure, being multi-sectorial, is amazing. But the coordination among the sectors is poor. Even so, I recommend the Sekota structure be an independent office.

I; How is the political support for this programme?

D: They are only supporting us during mobilization work; they just open the programme with the speech; this is the support.

I; How do you think these strategies can improve the multi-sectorial nutrition programme?

D; The programme is very important, and the strategy made me immerse myself in the programme. We changed the mother at least three or four times; this is huge.

I; Now that I'm almost done with my questions, you can express any concerns you have about this programme.

D., thank you too. But the research has to be practical and recommend practical solutions for the programme.

Interview 5 – 01 district WC01

Data Collector: A , Translator, and Transcriber

Key; I: Interviewer, R: respondent

1. (I) name of district

(R) 01

2.(I)Sex:

(R) male

3(.I) Age;

(R) :36

4(.I) Marital status;

(R) married

5(.I) Professions;

(R) Business management

6.(I) Position;

(R) WC01

7. (I) Work experiences

(R) 11 years

8 (I): Tell me about the nutritional problem in your region, district, or locality.

(R) There I big problem in this area related with nutrition due to high fertility rate that is incomparable to the economy of the household to raise the children properly and scarcity of agricultural land, most people in the community fail to eat three times per day except few. Inappropriate utilization also is there among those who get food from farming.

9. Have you heard about the multi-sectorial nutrition programme?

I: If yes, would you say something about its policy, principles, and implementation?

(D): Yes, I have heard about the multi-sectorial nutrition programme, we also meet and work in collaboration with all the sectors involved in this program such as agriculture office, health office, and water and sanitation office.

10 (I): How does this problem trend look from the past to the present?

(D) There has been improvement in awareness of nutrition at present time compared to the past time as there is collaboration among different sectors to alleviate the problem. Our woreda is known internationally for being the most draught affected areas and receiving international aids for long period of time. It's in my recall that in 1994 Ethiopian calendar, there was a helicopter landed in korangoge kebeles of our woreda containing international aids for severely malnourished ones, since then the woreda has been receiving & dependent on aids. However though not totally changed, there is a good improvement being observed recently.

11 (I): What are the nutrition-related activities of your office?

I: Would you say more about each nutrition-related activity in your office? List of activities? Its aim? Any plan?

(D) As the programe is mainly concerned with women and children, in collaboration with other sectors involve in the programe like health extension workers, we assess and identify children and pregnant and lactating mothers that are candidate to safety-net programe and run the programe.

12 (I) What are the roles and responsibilities of your office to achieve the Implementation of multi-sectorial nutrition

(D). we identify and select malnourished children and mothers when we work in the community and pass on to agriculture and health office for them to take action. Otherwise we work in collaboration with all the sectors and no independent plan and action is in place for specific sector.

13 (I): Tell me the ownership of the programme.

I: Who is the coordinator of the programme and his responsibility?

(D) Focal persons in each sector are owners of the programe next to agriculture and health offices that are the main owners of the programe in general. The focal persons do not plan and act by themselves but collaborate with health and agriculture offices.

14 (I) Tell me the challenges of your office in relation to collaboration with other offices.

I: How about the procedure of the programme, such as: About the annual plan, half-year plan, quarterly plan, achievement report, meeting schedule, monitoring, and evaluation-related commitment

(D) I do not see any challenges in collaborating with sector offices involved as those identifies by us are linked to agriculture and health office where immediate actions are taken place. There is a meeting that takes place quarterly among sector offices led by health office and monthly among us

15 (I) What challenges are there for the community to achieve a multi-sectorial nutrition programme?

I: How about commitment of the community, is there any resistance from the community?

(R) There is no problem with regard to the community concerning the programe, they are very much cooperative.

16 (I): Tell me how the structure of this programme is organized.

I: the reporting system of the programme?

(D): As we do not plan by ourselves concerning the programe but cascade what is planned by health office, there is nothing that we report but the woreda health office report to regional higher officials.

17(I): Say something about the programme in relation to the budget.

Probe: How is your financial administration, adequacy or shortage?

(D) I can say nothing about the budget as our office is not the one running it but health office.

18. (I) How about human resource and other resource issues in your office?

(D): We have trained personnel regarding the programe

19 (I): How did the professionals who work on a multi-sectorial nutrition programme Capacitate?

I: Is there any opportunities for training, workshops, and others.

(D) Through training and different short-term workshops, save the children has provided extensive training to health extension workers and others where we ourselves have involved and received magnificent knowledge on the nutrition program

20 (I) have you had the consultant workshop on this programme?

I: who were participants?

(D) Yes, there are regular workshops organized by the save the children. Mostly the office head and focal person of the nutrition program had been participating.

21, How do you involve the community to create awareness?

(D) There are different forums of awareness creation that takes place in the community by professionals and political leaders.

22(I): What are the nutrition-related programmes other than multi-sectorial of your office?

I: what is a list of activities for this programme? Its aim? Any plan?

(D) save the children has been supporting school feeding program but I do not know if there are other organizations supporting the programe.

23 (I): Is there a designated responsible body to coordinate the programme?

If yes, how is she or he committed?

(D) if you mean from this specific office, the head of this office (women and children office) is the main coordinator of the program.

24 (I) What are the strategic and operational plans of your office in multi-sectorial nutrition programme

(D) we have neither strategic nor operational plan by ourselves but we support and act according to what other sectors like health office and agricultural offices plan.

26(I): Tell the presence of a promising work structure in this programme ?

(D) it is our role as a stakeholders to facilitate the program and we are trying our best to keep the programe moving.

27(I) How is the political support for this programme?

(D) There is active participation and strong support from the political people, as the politicians are part of the programe, helping on the practicability of it.

28 (I) What do you think of the recommended strategy to improve the Implementation of a multi-sectorial nutrition programme in your district or region?

I: What do you think recommended strategy regarding your sector?

(D)if we ,the stakeholders, continue with the way we are collaborating with now, we are surely going to achieve the objective.

29 (I): How do you think these strategies can improve multi-sectorial nutrition? program?

(D) more collaboration from stakeholders could improve the programe.

Interview 6 – 01 district WCNF

Data Collector, Translator, and Transcriber: A

Key; I: Interviewer: A

D: Discussant

1. (I) name of district

(D) 01 district

2.(I)Sex:

(D) male

3(.I) Age;

(D) 34

4(.I) Marital status;

(D) Married

5(.I) Professions;

(D) masters in business administration

6. (I) Position;

(D) WCNF

7. (I) Work experiences

(D) 15 years

8 (I): Tell me about the nutritional problem in your region, district ?

I: How is the nutritional problem in this locality?

(D) yes, there is problem related to nutrition in our woreda, 13 out of the 14 kebeles in our woreda are the beneficiaries of safety net programe indicating the presence of the problem which is mainly due to irregularity in climate for the agriculture in entirely dependent on rain water & on top of that there is poverty.

9 (I): Have you heard about the multi-sectorial nutrition programme?

I: If yes, would you say something about its policy, principles, and implementation?

(D) Yes, sometimes there is a meeting that take place among technical teams and the steering committee which I participate in. during our meeting we talk about issues related to child and maternal nutrition and the importance of balanced diet to prevent stunting for the stunted child is not going to be productive nor would be of aide for himself and the community as well.

I: what about policy?

(D): there are two things involved under safety-net program; the first is those who can't work receive aids directly however those who can work but has burden at home with household chores in addition to breastfeeding their under two years children and pregnant ones are allowed to come to work and leave their work place early.

(I) What does your office do related to nutrition?

(D); we encourage women involvement in different activities like economic, political, educational & social issues and help them empower themselves for nutrition is directly related to their empowerment. We provide different cereals like beans to plant in their farm for women which replaces what they get from meat and aid them with hens, sheep's and goats to rear and benefit from.

12: What are the roles and responsibilities of your office to achieve the Implementation of multi-sectorial nutrition?

(D) we participate in different meetings called up by health office and agriculture office related to nutrition, we make sure mothers are provided with balanced diet during pregnancy and after child birth via 1to 5 networking.

13 (I): Tell me the ownership of the programme.

I: who is the coordinator of the programme and his responsibilities?

(D) Because the program mainly deals with mothers and children, the primary ownership goes to health office and agriculture office is the secondary owner as food production is mainly related to it and it is the backbone to overcome hunger.

14 (I) Tell me the challenges of your office in relation to collaboration with other offices?

I: How about the annual plan, half-year plan, quarterly plan, achievement report, meeting schedule, monitoring, and evaluation-related commitment

(D); we do not have any challenge in collaboration with others offices everything is taking place smoothly. Everything is going well; all the plans, achievement reports, quarterly supervisions, meeting schedules, monitoring and evaluation related commitments are going according to plan.

15 (I) what challenges are there for the community to achieve a multi-sectorial nutrition programme?

I: how about the commitment of the community and any resistance from the community.

(D) The community acts according to what they are told to. So as we, the supervisors, are good there is no way for the community not to be good. They are good enough to cooperate and collaborate since the launching of every program.

16 (I): Tell me how the structure of this programme is organized?

I: how about the reporting system of the programme.

(D) The main owner of the program is health office, so we report our work to them and sometimes, for the most part it is preferable to get them involved in activities than mere reports, so they are involved accordingly.

17(I): Say something about the programme in relation to the budget.

I: the financial administration, adequacy or shortage, and others?

(D) There is inadequacy of budget to run the program there are situations where we fail to refill a fuel the car to supervise some areas regarding the program.

18 (I): how about human resource and other resource issues in your office?

(D) All the personnel's are well equipped and knowledgeable regarding to what they do, for this reason there is no problem concerning human resource but budget.

19 (I): How did the professionals who work on a multi-sectorial nutrition programe capacitate?

I: any opportunities for training, workshops, and others.

(D) There are different trainings, workshops and experience sharing among different sectors collaborating on the issue to fill gaps of knowledge among each other though there is budget shortage to undergo it adequately.

21 (I): How you involve the community to create awareness?

(D) We sometimes prepare forum for exemplary people to provide awareness creation in the community about the programe. Furthermore all the sectors involved are playing their own part to create awareness.

22(I): What are the nutrition-related programmes other than multi-sectorial of your office?

I: what are a list of activities for this programme. Its aim? Any plan?

(D) There is programe called safety –net that help selected people with very hard financial constrain in the kebeles and school feeding programe organized by save the children where they supply food at schools to maintain smooth teaching learning process, reduce and ultimately eliminate drop outs, however the variety and quality of food they provide is decreasing lately. Save the children has also been providing training & creating awareness for husbands to share responsibility at household. In addition to this they have been working on different women empowerment programs.

24 What are the strategic and operational plans of your office in multi-sectorial nutrition programme.

(D) Agriculture and natural resource office & health office are the offices that work on poverty reduction & have an operational plan that entails selecting households eligible for safety- net programe and supply food for those who needs immediate help. Our office also undergoes some operational and strategic plans accordingly.

25(I): How is the community committed to supporting the activity plan of this programme?

(D) As I said earlier the community acts according to what they are told to. So as we, the supervisors, are good there is no way for them not to be good. They are good enough to cooperate and collaborate. They say being aware of the problem is half way to the solution. Admitting the presence of problem inspire us and the community to plan and act accordingly. For this reason the community is more than cooperative to the programe. One of the indications for the community cooperating to the program is that they are planting and cultivating what they are being provided, as a result there is greener environments compared to the past times. The only impediment and hindrance climate change resulting in drought,

26(I): Tell the presence of a promising work structure in this programme.

(D) Working in collaboration with all the sectors involved in the program and having supportive political system is the promising thing in addition to the proficient human resource in the program. if I give you one example of program ,in the long run there would be no one going out to market to buy an egg as there would be plenty at each household as they are being provided with hens for egg supply,

27(I) How is the political support for this programme?

(D) The government is very much supportive of the programe except the budget shortage related to the current political situation in the country that impede easy undergoing of the programe.

28 (I) what do you think of the recommended strategy to improve the Implementation of a multi-sectorial nutrition programme in your district or region

I: How about a general and office/sector-contextualized recommended Strategy?

(D) there should be more awareness creation activities in the community for they are only concerned in filling their stomach than consuming balanced diet when everything have at their

disposal. Maximum effort should be put in place to create awareness in the community to replace what they are buying from market by products from their backyard (beans from backyard instead of spending the already scarce money.

29 (I): How do you think these strategies can improve multi-sectorial nutrition? program?

(D) During earlier time women and children used to eat husbands leftovers but nowadays this trend has changed and they should nourish themselves with balanced diets because they are the main focus in the programe. They should have life for themselves to give life and procreate, they should help themselves with extra meal during pregnancy and lactating and this has to be tol for them during different forums in the taken place in the community. There should be budget and logistic improvement for the programe to run optimally.

Interview 7 – 03 district

Data Collector: Ayantu, Translator, and Transcriber

Key; I: Interviewer

D: Respondent

1. (I) Worada/district/partner/region office name:

(D) 03 district

2.(I)Sex:

(D) female

3(.I) Age;

(D) 35

4(.I) Marital status;

(D) married

5(.I) Professions;

(D) BSc management

6.(I) Position;

(D) WC01

7.(I) Work experiences

(D) 14 years

Section 2: Multi-sectorial nutrition programme implementation-related challenges in this office/partner

8 (I): Tell me about the nutritional problem in your region, district, or locality.

I: the nature of the nutritional problem in the local context.

(D) Mothers have proclivity to sell whatever they produce at household level including eggs instead of utilizing at home. There is scarcity of food at household level due to draught as a result of absent rainfall on top of these because of local conflict with neighboring region, there is instability leading to non-production resulting in food shortage.

9. Have you heard about the multi-sectorial nutrition programme?

I: If yes, would you say something about its policy, principles, and

implementation?

(D): Yes, we meet and work in collaboration with all the sectors involved.

10 (I): How does this problem trend look from the past to the present?

(D)There has been improvement in awareness of nutrition at present time compared to the past time as there is collaboration among different sectors to alleviate the problem. Our woreda is known internationally for being the most draught affected areas and receiving international aids for long period of time. It's in my recall that in 1994 Ethiopian calendar, there was a helicopter landed in korangoge kebeles of our woreda containing international aids for severely malnourished ones, since then the woreda has been receiving & dependent on aids. However though not 100 %, there is unbelievable improvement being observed recently.

11 (I): What are the nutrition-related activities of your office?

I: Would you say more about each nutrition-related activity in your office?

List of activities? Its aim? Any plan?

(D) As the program is mainly concerned with women and children, in collaboration with other sectors involve in the program like health extension workers, we assess and identify children and pregnant and lactating mothers that are candidate to safety-net programe and run the programe.

12 (I) What are the roles and responsibilities of your office to achieve the

Implementation of multi-sectorial nutrition

(D). we identify and select malnourished children and mothers when we work in the community and pass on to agriculture and health office for them to take action. Otherwise we work in collaboration with all the sectors and no independent plan and action is in place for specific sector.

I: the coordinator of the programme and this body's responsibility.

(D) Focal persons in each sector are owners of the programe next to agriculture and health offices that are the main owners of the programe in general. The focal persons do not plan and act by themselves but collaborate with health and agriculture offices.

14 (I) Tell me the challenges of your office in relation to collaboration with other

offices.

I: detail about the procedure of the programme, such as:

About the annual plan, half-year plan, quarterly plan, achievement report,

meeting schedule, monitoring, and evaluation-related commitment

(D) I do not see any challenges in collaborating with sector offices involved as those identifies by us are linked to agriculture and health office where immediate actions are taken place. There is a meeting that takes place quarterly among sector offices led by health office and monthly among us

15 (I) What challenges are there for the community to achieve a multi-sectorial

nutrition programme?

I: the commitment of the community and for any resistance from the

community.

(D) There is no problem with regard to the community concerning the programe, they are very much cooperative.

16 (I): Tell me how the structure of this programme is organized.

I: the reporting system of the programme.

(D): As we do not plan by ourselves concerning the program but cascade what is planned by health office, there is nothing that we report but the woreda health office report to regional higher officials.

17(I): Say something about the programme in relation to the budget.

I: the financial administration, adequacy or shortage, and others.

(D) I can say nothing about the budget as our office is not the one running it but health office.

18. (I) HR and other resource issues in your office

(D): We have trained personnel regarding the programe

19 (I): How did the professionals who work on a multi-sectorial nutrition programme

Capacitate?

I: opportunities for training, workshops, and others.

(D) Through training and different short-term workshops, save the children has provided extensive training to health extension workers and others where we ourselves involved and received magnificent knowledge on the issue

20 (I) Have you had the consultant workshop on this programme?

I: If yes, probe for details like who were participants?

(D) Yes, there are regular workshops organized by the save the children.

21, How do you involve the community to create awareness?

(D) There are different forums of awareness creation that takes place in the community by professionals and political leaders.

22(I): What are the nutrition-related programmes other than multi-sectorial of your

office?

I: ask for a list of activities for this programme. Its aim? Any plan?

(D) save the children has been supporting school feeding program but I do not know if there are other organizations supporting the programe.

23 (I): Is there a designated responsible body to coordinate the programme?

If yes, how is she or he committed?

(D) if you mean from this specific office, the head of this office is the main coordinator of the program.

24 (I) What are the strategic and operational plans of your office in multi-sectorial

nutrition programme

(D) we have neither strategic nor operational plan by ourselves but we support and act according to what other sectors like health office and agricultural offices plan.

26(I): Tell the presence of a promising work structure in this programme.

(D) it is our role as a stakeholders to facilitate the program and we are trying our best to keep the programe moving.

27(I) How is the political support for this programme?

(D) There is active participation and strong support from the political people, as the politicians are part of the programe, helping on the practicability of it.

28 (I) What do you think of the recommended strategy to improve the

Implementation of a multi-sectorial nutrition programme in your district or region

I: a general and office/sector-contextualised recommended

strategy?

(D) if we, the stakeholders, continue with the way we are collaborating with now, we are surely going to achieve the objective.

29 (I): How do you think these strategies can improve multi-sectorial nutrition?

program?

(D) more collaboration from stakeholders could improve the programe.

Interview 8 – 03 district WCNF

Data Collector: A , Translator, and Transcriber

Key; I: Interviewer

D: Discussant

1.(I) Woreda/district/partner/region office name:

(D) 03

2.(I)Sex:

(D) male

3(.I) Age;

(D) 30

4(.I) Marital status;

(D) married

5(.I) Professions;

(D) Civics and ethical education.

6.(I)Position;

(D) WCNF

7. (I) Work experiences

(D) 13 years

8 (I) Tell me about the nutritional problem in your region/district/locality?

I: the nature of the nutritional problem in the local context.

Among districts, in Sidama regional states our woreda is highly affected by climate changes and by being low lands which increases scarcity of food and mostly affected from nutritional problems.

9 (I) Have you heard about the multi-sectorial nutrition program?

I: If yes, would you say something about its policy, principles, and implementation?

9(I) How this problem trend looks from past to the present?

(D) Our district is low-land area (‘‘kola’’) /scarcity of water resources due to this ,the most of years there is nutritional problem because of the climate change and lack of water resources in the district.

10 (I) Have you heard about the multi-sectorial nutrition program?

I: If yes, would you say something about its policy, principles, and implementation?

I; Yes, I heard about multi-sectorial nutrition program ,which support preventing stunting for under two years children and availing different types of foods for pregnant and lactating mother.

11 (I) What are the nutrition related activities of your office?

I: Would you say more about each nutrition related activity in your office?

List of activities? Its aim? Any plan?

(D) Our sector responsibility creating aware especially under two children, pregnant, lactating mother including their husbands about the balanced diet and accordingly, our plan we evaluate the program weather achieved or not.

12(I) What are the roles and responsibilities of your office to achieve the Implementation of multi-sectorial of nutrition?

(D) Strengthening and encouraging women's and creating awaress about saving the money and building economy in the house hold level rather than depending on their husbands as well training women's in order to build capacity.

13(I) Tell me the ownership of the program

I: what are responsibility of the coordinator of the program?

(D) Stakeholders are responsible and ownership for the program and to overcome nutritional problems at house hold level rather than individuals and targeting prosperity at the house hold level for improving nutritional status. All multi-sectors work hand in hands by integrating and collaborating to tackle nutritional problems without assigning individual tasks. To summarize, all multi-sectors for this program ownerships regardless of sectors which led by woreda health office.

14(I)Tell me the challenges of your office in relation to collaboration with other offices.

I: procedure of program?

About the annual plan, half year plan, quarterly plan, achievement report, meeting schedule, monitoring and evaluation related commitment

(D) Our challenges are lack of motor bikes for transportation, scarcity of budget in our district, logistics for stationary. According to our plan we evaluate twice in month and finalize the report in three month report without interruptions of the program by participating woreda administer and councils of woreda. All multi-sectors have focal person for sekota multi-sectorial nutritional program which leads by woreda health office, this makes effective for integration and collaborations to tackle challenges.

15 (I) What challenges are there for the community to achieve a multi-sectorial nutrition program?

I: commitment of the community, for any resistance from the community.

D: Accordingly our district, we select among 19 kebeles ,we selected two kebeles and supporting , encouraging and working to be model for others .Criteria for selection those who have children under two ,pregnant and lactating women.In the past years, there were challenges in order to select very poor community and including data base management. Currently no challenges arise due to supervisions of stake holders with in time. But, all community are committed to achieve multi-sectorial nutritional program without any resistance.

16 (I) Tell me how the structure of this program is organized?

I: the reporting system of the program

(D) multi-sectorial nutrition program has its own string committee in the kebeles and collaborated with hygiene and sanitation as well as district level accordingly ,which leads by kebeles chairperson that makes organized achievement from top-down stakeholders and weekly and monthly report under gone from low-to- high levels of responsibilities of multi-sectorial accordingly.

17(I) Say something about the program in relation to the budget?

I: financial admin, adequacy or shortage, and others

(D) Under sekota multi-sectorial nutritional program targets many aspects at the house hold level but due to scarcity of budget we couldn’t achieve regardless of our plan.

18(I) HR and other resource issues in your office?

(D) We have not enough skilled human resource, because of scarcity of budget we couldn’t hire other professionals in the district, lack of computer, smart phone also our challenges.

19 (I) How did the professionals who work on a multi-sectorial nutrition program capacitate?

I: opportunities for training, workshop and others

(D) No trainings, supportive supervision and encouragement through rewards in the district.

20 (I) Have you had the consultant workshop on this program?

I: if yes, details like who were participants?

(D) Yes, Participating by community in order to creating ownerships, integration and collaborations for preventing stunting for under two years children.

21 (I) How you involve the community to create awareness?

(D) We involved the community after creating awareness by using local language through the radio by their kebeles, religion and neighbor leaders and creating awareness how to feed different types of feed for under two children, pregnant and lactating women.

22 What are the nutrition related programs other than multi-sectorial of your office?

I: list of activities this program? Its aim? Any plan?

(D) No other program in the woreda.

23 (I) s there designated responsible body to coordinate the program?

If yes, how she/he is committed?

(D) we should integrate and collaborate multi-sectorial nutritional program plan ,according the given tasks and leads by string committee leaders ,woreda administration and committed for the activities.

24(I) What are strategic and operational plans of your office in multi-sectorial nutrition program

(D) All activities regarding to multi-sectorial nutritional program are based on operational plan because, this program aimed for prevention stunting, pregnant and lactating women in the district.

25(I) How community are committed to support the activity plan of this program

(D) For creating awareness and supporting nutritional programs for the community ,kebeles leaders, health extension workers, health development army are very committed to support by any aspects and multi-sectorial nutritional program are committed , but regardless of lack of budget .

26(I) Tell the presence of promising work structure of this program

(D) Those who pregnant and lactating mother don't awarded how to feed different kinds of food ,But currently , after multi-sectorial nutritional program launched :community are ready and practice such programs and know the importance of integrations and collaborations of multi-sectorial nutritional programs rather than giving for single sector.

27(I) How is the political support of this program:

(D) In our district political support are committed for any aspects and leaded by woreda administer.

28(I) What do you think on the recommended strategy to improve the implementation of multi-sectorial nutrition program in your district/region?

I: A general and office/sector contextualized recommended strategy?

(D) My recommendations are increasing budget providing cost based analysis for water related logistics supplies in the district in order to improve and implement for the multi-sector nutritional program.

29(I) How do you think these strategies can improve the multi-sectorial nutrition program?

(D) Firstly, creating awareness for the community that the importance of multi-sectorial nutritional program, increasing annually budget and to sustain nutritional program in the house hold level.

Interview 9 – 04 district WC01

Data Collector: A , Translator, and Transcriber

Key; I: Interviewer

D: Respondent

1. (I) Worada/district/partner/region office name:

(D) 04

2. (I) Sex:

(D) female

3. (I) Age;

(D) 42

4(.I) marital status;

(D) Married

5(.I) Professions;

(D) Second degree in Business administration

6. (I) Position;

(D) WC01

7. (I) Work experiences

(D) Overall I have 15 years' experience with 6 years as sector office management in women's league, finance and trade and industry office; in the current position I have only five month experience.

8 (I). Tell me about the nutritional problem in your region/district/locality?

I: the nature of the nutritional problem in the local context.

(D): yes, there is a huge nutritional problem due to the current climate change. When there is a nutritional problem, women and children are the first to suffer other family members are lately exposed. In the past year due to the climatic change the expected rain did not fall at the beginning of the year and still the seasonal rain did not at the time and the community is suffering from food shortage. The farmers are also afraid to cultivate their land due to the loss in the previous year because of the shortage of rain. In addition, the people in our vicinity is densely populated which is also additional reason for the food insecurity.

9 (I). How this problem trend looks from past to the present?

(D): The problem increased compared to the previous year, the problem has significantly increased due to the shortage of rain resulting in poor agricultural production, making it difficult to even survive. Even there is high shortage of fodder for the livestock too. The district is known for its high production of maize even if it is a drought-prone district with high temperatures. Due to the fact that in the past two years the community has not gotten any results from agriculture, it is becoming difficult to fulfil their basic needs. Specially, the lower 18 kebeles suffered from high agricultural production loss. Previously the price of one quintal maize is 1000 or 2000 but now it is 6000/7000 birr which makes it even difficult to purchase for the community.

10 (I). Have you heard about the multi-sectorial nutrition program?

(D): Yes, sometimes there is a training for the focal persons in each office and also the steering committee which I participate in. the program focused on working on child and maternal nutrition and the importance of balanced diet to prevent malnutrition and food security in general. The program is conducted in coordination with six sectors including health office, agriculture and livestock development office. We have 25 kebeles in our district however due to the existing budget shortage in the program we are working only two kebeles. We are providing training to women regarding trading and selling skills and appropriate dietary practice among mothers and children. Generally we are working to reduce poverty.

(I) I: If yes, would you say something about its policy, principles, and implementation?

(D); the seqota declaration started at Amhara region, the area that is highly impacted with food insecurity and malnutrition. The program developed and believed that if we decrease the nutrition condition at this area we can change in all districts in the country. The program currently working to improve the nutritional status of children and mothers who are pregnant and breast feeding.

11. (I). What are the nutrition related activities of your office?

(D): The nutrition-related work we conduct relates to food and nutrition, raising knowledge about nutrition, and eating a balanced diet, particularly for mothers and children. Mothers are the primary individuals responsible for family feeding habits. Therefore, it is important to educate them on the importance of balanced nutrition for their families. Additionally, our work also involves promoting access to affordable and nutritious food options in underserved communities. We also encourage women's involvement in different activities like economic, political, educational, and social issues and help them empower themselves and their families. By providing them with the necessary knowledge and resources, we aim to create a healthier and more equitable society where everyone has the opportunity to thrive. Through our efforts, we hope to inspire positive change and make a lasting impact on the lives of those we serve. We are working with restricted budget by engaging the health extension workers, one to five network, development group and religious leaders.

(I: Would you say more about each nutrition related activity in your office? List of activities? Its aim? Any plan?

Some of the most effective ways to empower women are to strengthen and encourage them, raise awareness about the importance of budgeting and building household economies so that women don't have to rely on their husbands, and train them to become more capable. Women's empowerment can aid in eradicating poverty and advancing gender equality. It is critical to understand that supporting women's empowerment is both a moral and an economic necessity.

(I): what are the activities your office working particularly in multi-sectorial nutrition program?

(D): Our work with mothers starts at the beginning of the pregnancy and continues until the end. The other sectors support the women by providing hens, goats, and livestock; we create awareness about the appropriate usage of these resources. To improve the health of the mother and newborn baby, we teach them to get vaccinations during pregnancy and for the infant. Additionally, we also provide education on harmful practices and hygiene practices to ensure a healthy life for the mother and the child. Our goal is to empower women with the knowledge and resources necessary to make informed decisions about their health and the health of their families. We are working with the health extension workers to achieve these goals.

13. (I). Tell me the ownership of the program

I: responsibility?

(D): at the woreda level the steering committee is led by the head of district health office while in our office I am the one leading the activities. In addition, there is a focal person in our office, there is also a program coordinator whom working with the steering committee. We work coordinately with the woreda program coordinator during activities like providing training.

14. (I) Tell me the challenges of your office in relation to collaboration with other offices.

I: Detail about the procedure of program such as; About the annual plan, half year plan, quarterly plan, achievement report, meeting schedule, monitoring and evaluation related commitment.

(D); There are no issues with our collaboration with other offices; everything is going as planned. The plans, achievement reports, quarterly supervisions, meeting schedules, monitoring commitments, and evaluation commitments are all going as expected. Our team has also been successful in keeping in touch with our sister offices, and the programme coordinator makes sure that everyone is aware of the status of the project and any emerging problems. This has further contributed to the success of our collaboration efforts by enabling us to quickly and effectively address any issues.

15. (I). What challenges are there for the community to achieve a multi-sectorial nutrition program?

Probe: ask for the commitment of the community, for any resistance from the community.

(D): previously we are worked at four kebeles but now we are working only in two kebeles with twelve families. The community is very happy with program. The only concern was from those kebeles we are not working on, they want the program to start in their kebele too.

16 (I): Tell me how the structure of this programme is organized.

I: reporting system of the programme.

(D): The woreda health office report to regional office. We never plan by ourselves as I mentioned earlier we run want is planned by others.

17(I): Say something about the programme in relation to the budget.

Probe: ask for the financial administration, adequacy or shortage, and others.

(D) I can say nothing about the budget here, we already have finished 90% of it. It is very embarrassing. I guess at least 5 million ETB might have been allocated to the programme in our woreda but they have docked and given us 128860 ETB which is very ridiculous for a programme that deals with something related to mothers, children and nutrition.

18. (I) HR and other resource issues in your office

(D): We have trained personnel regarding the programme, no problem regarding human resource but budget and vehicle (which we wish to use to travel to kebeles hard to reach without vehicle)

19 (I): How did the professionals who work on a multi-sectorial nutrition programme Capacitate?

I: opportunities for training, workshops, and others.

(D) There is no formal training available for the workers in the area but they try to get some knowledge from each other through experience sharing.

22(I): What are the nutrition-related programmes other than multi-sectorial of your office?

I: list of activities for this programme. Its aim? Any plan?

(D) There is a programme called ESD that works on females who are under the age of 18. They train on them on saving. Women and give them goats to rear. They also support disabled children and those who need special attention supplying pen and exercise book and 500 birr every year. There also was an NGO named center of concern that used to work on harmful tradition practices that has been phased our already.

21, How do you involve the community to create awareness?

(D) We distribute concerned staffs in every stakeholders to the community, kebele, with checklists and do the assessment and everything in the checklist and evaluate their activities upon their arrival back to office.

22. (I): Is there a designated responsible body to coordinate the programme?

If yes, how is she or he committed?

(D) As I said earlier the woreda administrators is leading us then health office then heads of each stakeholders are responsible.

24 What are the strategic and operational plans of your office in multi-sectorial nutrition programme.

(D) our operational plan is preventing maternal and child death but our strategic plan is alleviating and eradicating poverty through long run

25(I): Tell the presence of a promising work structure in this programme.

(D) Working in collaboration with all the sectors involved in the program and having supportive political system is the promising thing, it is our role as the managers of stakeholders to facilitate the program and we are trying our best to get the programme going. We have been sharing experiences among kebeles of best performers and found it very interesting.

27(I) how is the political support for this programme?

(D) There is active participation and strong support from the political sect as, we the leaders of the programme, are politicians already and competing to finish given task before due time and supporting each other.

28 (I) what do you think of the recommended strategy to improve the Implementation of a multi-sectorial nutrition programme in your district or region

I: a general and office/sector-contextualized recommended strategy?

(D) we are responsible only for our duties & are not able to verify who else is doing good and bad, but if there is unlimited effort from every sector, it would be great.
