# Supplementary material for: Exploring the challenges and opportunities of multisectoral nutrition programme in Ethiopia: A qualitative study on combating undernutrition during pregnancy
Source: PLoS One. 2025 Jul 3;20(7):e0311336. doi: 10.1371/journal.pone.0311336 (PMC12225801; doi:10.1371/journal.pone.0311336)
Supplement: Attachment — Submitted filename: Letter with point-by-point responses to reviewers comments.docx [file pone.0311336.s009.docx]

**April 1, 2025**

**To: The Editorial Committee**

PLOS ONE

On behalf of authors of the manuscript entitled “*Exploring the Challenges and Opportunities of Multisectoral Nutrition Programme in Ethiopia: A Qualitative Study on Combating Undernutrition during Pregnancy*”, I would like to pass our sincere thanks and appreciation to the reviewers and the editor of PLOS ONE Journal for giving us invaluable comments and feedback on our manuscript.

Appended with this letter are subsequent pages that hold point-by-point responses to comments forwarded by the reviewers and Editorial Board. The revisions made on the original manuscript are track changed to ease visibility. Furthermore, the manuscript is proofread by a native English speaker and substantial changes are made.

Sincerely,

Nana Chea

Corresponding author

**Reviewer 1**

| **No** | **Comment** | **Response** |
| --- | --- | --- |
| 1 | I congratulate the authors on the study and thank them for the opportunity to evaluate it. | - We thank you very much for your time and thorough review of our works and for the appreciation that encourages us to do more and to contribute more to the scientific world. |
| 2 | In the introduction, I suggest showing more of the originality of this work: What is different? Why publish it? | - On the background section of this study, from line 83 to line 89 we indicated the knowledge gap of this study under. However, still now we have improved a concept that shows an originality of work, knowledge gap (difference) and why doing or publishing it is important in the introduction section of the manuscript. - It is track changed for clear visibility |
| 3 | I suggest improving the tables, presenting them in a more didactic and scientific way. | - We have also much improved presentation of table. - It is track changed for clear visibility |
| 4 | How was this age division made? | - Thank you very much for this comment. Now we have revised the age division of the study participants with acceptable references. We categorised the age of the participants in this study according to the usual age groupings for adults in the National Demographic and Health Survey. - It is track changed for clear visibility |

**Reviewer 2**

| **No** | **Comment** | **Response** |
| --- | --- | --- |
| 1 | The authors highlight challenges seen in other countries on multisectoral nutrition programmes but there was no background information about Ethiopia. How did you identify that this was a challenge in Ethiopia? | Thank you for that observation and comment. The Multisectoral Nutrition Programme in Ethiopia (‘SEQOTA’) was mentioned in the background section from line number 62 to 68. That is, the country has launched the historic Seqota Declaration, a multisectoral collaboration to combat stunting and wasting among children and pregnant women in the northern part of the country and is currently in its second phase (expansion phase) of spreading throughout the country. It aims to end the cycle of poverty by supporting healthy growth and nutrition in the first 1,000 days of life, with a long-lasting impact on subsequent generations, and is in line with the United Nations Sustainable Development Goals. However, we agree with you that mentioning the previously known challenges in Ethiopia was overlooked. Thank you for your comment. It is now well incorporated and it is tracked for clear visibility. |
| 2 | Line 100 - 108: What is the relevance of this information? | Thank you for your critical observation.   - The details, particularly on the population and health facilities of the study area, were mentioned because we thought it would be useful for readers to understand the study area. However, from your comment we understand and agree that this information is unnecessary detail. So, it is update and only very important one included. it is tracked for clear visibility |
| 3 | Line 125: I had to try and figure what these stands for. Spell out IDIs and FGDs in the first instance for everyone to follow | Thank you for this comment.   - We agree that all acronyms and abbreviations should be fully spelled in the first instance. However, IDIs and FGDs were not. Thank you for this comment, and it is now spelled out in the first instance. At the same time, we checked for others for similar mistake not to happen and it is track changed for clear visibility. |
| 4 | Line 127: You say you had a present sample size. So how did you use data saturation principle when you had already estimated 20? And why did you end up with 36 (line 128) when you had estimated 20? Line 133: Why did you end up with four and not ten? | Thank you for your critical review and observing this point.   - There has been a dilemma in deciding the sample size for the qualitative study for a long time. According to some authors, different study types require different sample sizes. For example, the minimum recommended sample size for a phenomenological study design is 5-20 interviews according to (Sandelowski M, et al, 1999). - There are also recommended sample sizes for other study designs such as ethnographic study design. But your remarks made us realize that the difference between the estimated and actual sample sizes is significant, taking into account the satiation principle. - Finally, we accepted your comments and corrected accordingly. Thank you for this comment. it is also track changed for clear visibility. |
| 5 | Line 135: If you were considering this, then the present sample size does not work. What is the minimum for a qualitative study? That is what you use if you are using the data saturation principle. | Thank you for this comment.   - We understand that this comment is also in line with the above comment. Except that applying the data saturation principle or redundancy of the response of the participant, we agree that the estimating sample size in qualitative study may not work. it is also track changed for clear visibility. |
| 6 | Write in the correct order of doing things | Thank you for observing this. We have accepted the comment and highlighted with track change. |
| 7 | It is still unclear which theoretical framework was used for this study. On what are you basing your work? | In line 91 we stated that the theoretical framework for this study was phenomenological. This is because the aim of this study was to understand and describe the experiences of people working on and around a multisectoral nutrition programme. However, based on this comment, we have added the description to it and it is clearly track changed for clarity. |
| 8 | Line 153 - 157: How was objectivity achieved during the coding process? How many authors conducted coding and how were these compared? | - Thank you for this important comment. - In order to achieve objectivity during the coding process, two investigators openly coded the generated transcripts using an inductive approach. The two researchers checked the consistency of coding on a daily basis during peer debriefing. In case of disagreement, a third researcher was consulted to reach a common understanding of the coding. The two investigators then assessed the revised set of codes for textual congruence and linkage using axial coding. - Subsequently, similar codes were systematically categorised and categories were labelled. Finally, the labelled categories were transformed into candidate themes and final non-repetitive themes. - Based on your comment, this description is well incorporated - Finally, it is track changed for clear visibility. |
| 9 | The paper has several grammatical and spelling errors. I urge the authors to thoroughly read through and correct these. | - Based on your comment, we thoroughly revised the paper using English language experts or proofread by a native English speaker and substantial changes are made. |

**Reviewer 3**

| **No** | **Comment** | **Response** |
| --- | --- | --- |
| 1 | This article examines a qualitative study to understand facilitators, barriers, and implications that address undernutrition in pregnant women participating in a multisectoral nutrition programme in Ethiopia. The authors provide sound evidence of a careful and considerate community-based study that utilized interviews and focus groups. | - We thank you very much for your time and thorough review of our works and for the appreciation that encourages us to do more and to contribute more to the scientific world. |
| 2 | However, there were many grammatical errors that made it hard to read the manuscript. I would suggest a major revision for editing. | - Based on your comment, we thoroughly revised and proofread by a native English speaker and substantial changes are made. It is track changed for clear visibility. |
| 3 | Some of the themes that were presented appear to have overlap and redundancy, specifically 'Lack of operational structure,' and 'Shortage of programme budget and logistics.' Overall, well done study. | - Thank you so much for the critical observation of these points. we thoroughly revised these points based on your comments. Thank you once, more again for critical review! |

**Editor’s Comment**

| **No** | **Comment** | **Response** |
| --- | --- | --- |
| 1 | Please ensure that your manuscript meets PLOS ONE's style requirements, including those for file naming. | Thank you very much for reminding us to be sure that our manuscript is as per PLOS ONE’s style requirement as requiring the English language copy editing. We have revised our manuscript as per PLOS ONE’s style requirement. It is track changed for clear visibility. |
| 2 | Please include a complete copy of PLOS’ questionnaire on inclusivity in global research in your revised manuscript. Our policy for research in this area aims to improve transparency in the reporting of research performed outside of researchers’ own country or community. The policy applies to researchers who have travelled to a different country to conduct research, research with Indigenous populations or their lands, and research on cultural artefacts. The questionnaire can also be requested at the journal’s discretion for any other submissions, even if these conditions are not met. Please find more information on the policy and a link to download a blank copy of the questionnaire here: <https://journals.plos.org/plosone/s/best-practices-in-research-reporting>. Please upload a completed version of your questionnaire as Supporting Information when you resubmit your manuscript. | We included a complete copy of PLOS’ questionnaire on inclusivity in global research in our revised manuscript. |
| 3 | In the online submission form, you indicated that “The data that support the findings of this study are available on request from the corresponding author via [cheanana2007@gmail.com](mailto:cheanana2007@gmail.com)”  All PLOS journals now require all data underlying the findings described in their manuscript to be freely available to other researchers, either 1. In a public repository, 2. Within the manuscript itself, or 3. Uploaded as supplementary information. | Thank you, yes in the online submission form, we indicated that “The data that support the findings of this study are available on request from the corresponding author via [cheanana2007@gmail.com](mailto:cheanana2007@gmail.com)”. Accordingly, we agreed and all data underlying the findings have uploaded now as supplementary information. Moreover, data that support the findings of this study are available on request from institutional representatives who did not collaborate in the study, and are not listed as authors on the manuscript, who would be able to hold the data and respond to external requests for data access via his institutional email (ashagre.beyene@sphi.gov.et). In addition, we will deposit the research data in renowned, open-access repositories that are recognized for their long-term preservation and easy access. These repositories often have specific guidelines and standards for research data, which will help ensure that our findings remain widely accessible and citable. |
| 4 | When completing the data availability statement of the submission form, you indicated that you will make your data available on acceptance. We strongly recommend all authors decide on a data sharing plan before acceptance, as the process can be lengthy and hold up publication timelines. Please note that, though access restrictions are acceptable now, your entire data will need to be made freely accessible if your manuscript is accepted for publication. This policy applies to all data except where public deposition would breach compliance with the protocol approved by your research ethics board. If you are unable to adhere to our open data policy, please kindly revise your statement to explain your reasoning and we will seek the editor's input on an exemption. Please be assured that, once you have provided your new statement, the assessment of your exemption will not hold up the peer review process. | Thank you. All authors decided on a data sharing |
